# Minimalistic peptidic scaffolds harbouring an artificial carbene-containing amino acid modulate reductase activity†

**DOI:** 10.1039/d1cc03158a

**Published:** 2021-08-06

**Authors:** Karst Lenzen, Matteo Planchestainer, Isabelle Feller, David Roura Padrosa, Francesca Paradisi, Martin Albrecht

**Affiliations:** Department of Chemistry, Biochemistry & Pharmaceutical Sciences, University of Bern Freiestrasse 3 3012 Bern Switzerland Francesca.paradisi@dcb.unibe.ch martin.albrecht@dcb.unibe.ch

## Abstract

Inspired by the boom of new artificial metalloenzymes, we developed an Fmoc-protected histidinium salt (Hum) as N-heterocyclic carbene precursor. Hum was placed *via* solid-phase peptide synthesis into short 7-mer peptides. Upon iridation, the metallo-peptidic construct displayed activity in catalytic hydrogenation that outperforms small molecule analogues and which is dependent on the peptide sequence, a typical feature of metalloenzymes.

## Abstract 2

A non-natural histidinium amino acid has been developed and used for solid-phase peptide synthesis to construct a peptide iridium carbene conjugate as artificial mini-peptide for hydrogenation catalysis.

## Graphical abstract

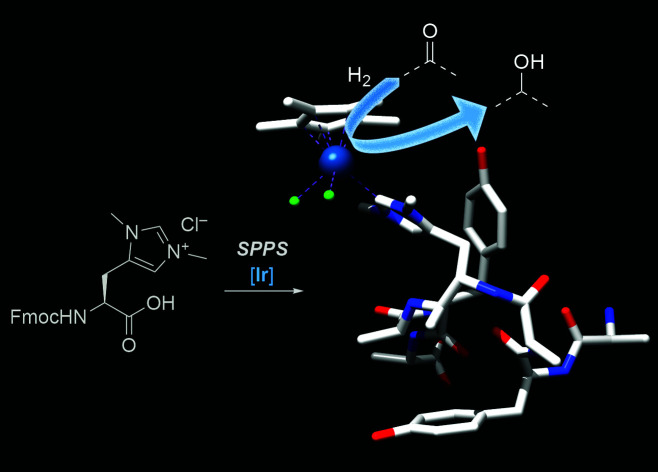

The implementation of abiotic reactivity is considered to be one of the most powerful strategies to broaden the toolbox of biocatalysis.^1^ Non-natural transformations have been evoked, for example, by directed evolution of natural metalloenzymes, *e.g.* silylation with P450,^2^ by site directed mutagenesis using (non-)natural amino acids,^3^ or by the incorporation of a synthetic co-factor with a specific abiotic transition metal center.^4^ This latter approach also allowed for incorporating N-heterocyclic carbene (NHC) metal complexes into a protein scaffold to produce evolvable artificial metalloenzymes that catalyze olefin metathesis^5^ and hydrogenation.^6^

The use of N-heterocyclic carbene complexes to generate minimalistic peptidic scaffolds displaying pseudo-enzymatic activity: on one handside, NHCs have been standing out as facilitator ligands in homogeneous catalysis^7^ and, on the other, the side chain of histidine (His) provides the imidazole skeleton of NHCs and may therefore potentially bind a metal center *via* classical N- or carbenic C-coordination.^8^ Previous work in our group^9^ and others^10^ has shown that histidylidene complexes are accessible, though incorporation into oligopeptides required the introduction of an external NHC ligand.^11^

Considering the potential existence of carbenes in biological systems,^2,3,8^ and the possibility to incorporate carbene-precursors in amino acids, we got intrigued to broaden the scope by incorporating a carbene precursor into peptide sequences and test the effect of different amino acid scaffolds in catalysis. Therefore, the histidinium salt **Fmoc-Hum-OH** (Scheme 1, abbreviated one letter code Ḧ) was designed with a Fmoc protection group which allows for use in standard solid-phase peptide synthesis (SPPS). Here, we show a simple series of palindromic heptapeptides with which we probe the effectiveness of coupling Hum in SPPS. Iridium was chosen as a metal center for binding the carbene, as Ir–NHC complexes have shown high activity in a range of catalytic applications,^12^ moreover, the Ir–C_NHC_ bond is exceptionally stable under acidic and basic conditions.^13^ This approach furnished metallo-peptides with pseudo reductase activity, demonstrated in the hydrogenation of acetophenone as model reaction.

**Scheme 1:**
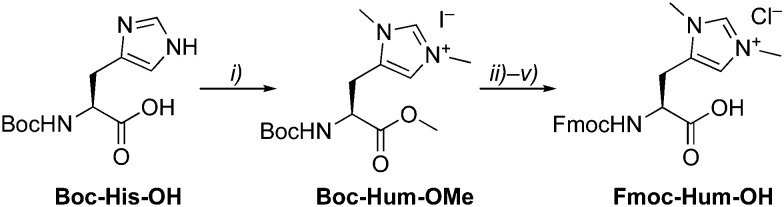
Synthesis of Fmoc-protected Hum (Ḧ). Reagents and conditions: (i) MeI, MeCN, 40 C; (ii) LiOH, MeOH/H_2_O, r.t.; (iii) PAc-Br, KF, DMF, r.t.; (iv) HCl, dioxane, then immediately FmocCl, Na_2_CO_3_, MeCN, r.t.; (v) Zn, HOAc, then HCl, dioxane, r.t.

The artificial amino acid Hum as NHC precursor was prepared by methylation of **Boc-His-OH**, which yielded **Boc-Hum-OMe** in good yields (Scheme 1).^14^ Sequential protecting group modifications gave **Fmoc-Hum-OH** over 4 steps in 47% overall yield. A direct Boc/Fmoc exchange after ester hydrolysis *via* the unprotected Hum gave only traces of the desired product in a complex mixture. **Fmoc-Hum-OH** was analyzed by NMR spectroscopy, MS, and its purity confirmed by microanalysis. Diagnostic NMR signals include the two singlets due to chemically distinct N–CH_3_ groups (*δ*_H_ 3.51, 3.48) and two aromatic resonances at *δ*_H_ 6.87 and 8.31 for H_*δ*_ and H_*ε*_, respectively. Derivatization of **Fmoc-Hum-OH***via***Hum–OMe** with (*S*)-Mosher's acid indicated at least 95% retention of enantiopurity (ESI†) and negligible racemization during the Boc deprotection step of the phenacyl (PAc) protected ester group.^15^

**Fmoc-Hum-OH** has the appropriate functionalization for application in SPPS and has been used to prepare a set of palindromic heptapeptides **AXAḦAXA** with varying amino acids X in the 2- and 6-position and Hum (Ḧ) in the central position (Scheme 2). To test the compatibility of the Hum towards coordination of metals in the presence of different functional groups, X was permuted with all 20 natural amino acids. This afforded 20 heptapeptides that were unfunctionalized neutral (X = Gly), hydrophobic (X = Ala, Val, Pro, Ile, Leu, Met), aromatic (X = Phe, Tyr, Trp), positively charged (X = His, Arg, Lys), negatively charged (X = Asp, Glu) or polar uncharged (X = Ser, Asn, Tyr, Gln, Cys). While coupling of the first three amino acids on the Wang resin followed standard procedures,^16^ coupling of **Fmoc-Hum-OH** required longer reaction times (5 *vs.* 1–2 h) to reach completion (TNBS control). Also, coupling of the subsequent Ala was performed twice to reach optimal results. Subsequent introduction of X and A proceeded again according to standard protocols.

**Scheme 2:**
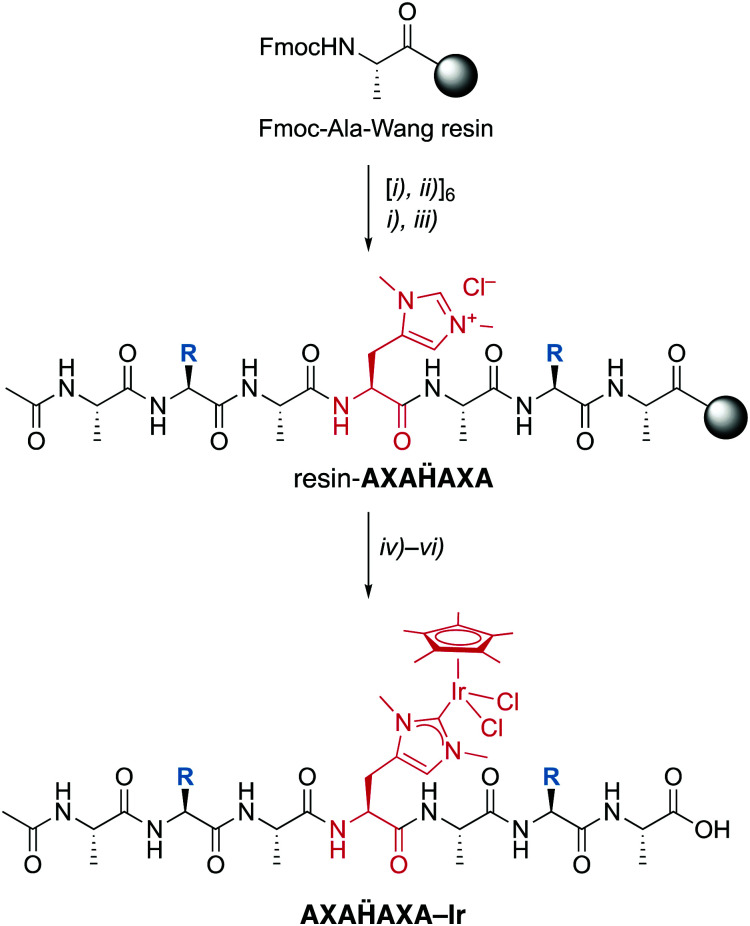
Solid-phase peptide synthesis (SPPS) of the palindromic AXAḦAXA heptapeptide and iridation to form a mini-metalloenzyme with a NHC iridium active site. Reagents and conditions: (i) piperidine in DMF; (ii) **Fmoc-Aaa-OH**, oxyma, collidine, DIC in NMP; (iii) AcOAc in CH_2_Cl_2_,; (iv) Ag_2_O, Me_4_NCl in CH_2_Cl2/MeCN; (v) [IrCl_2_Cp*]_2_ in CH_2_Cl_2_/MeCN; (vi) TFA in H_2_O. X = permutation of all 20 natural amino acids.

TFA-mediated cleavage of the heptapeptide followed by revers-phase HPLC purification yielded the apo-peptides sub-gram quantities (about 20 mg per 50 mg Wang resin with a 0.4–0.8 mmol g^−1^ pre-loading). Identity and purity were analyzed by high-resolution ESI-MS, LC–MS and NMR spectroscopy (†). For example, for the sequence **ASAḦASA** with two Ser and Hum and alternating Ala, ^1^H–^1^H COSY identified the Hum C_α_H-proton as a distinct triplet at *δ*_H_ 4.61, which is well-separated from the C_α_H-protons of the other amino acids (*δ*_H_ 4.18–4.35). The Hum H_*δ*_ and H_*ε*_ appeared as singlets at *δ*_H_ 7.17 and 8.52 ppm, respectively. The presence of just one set of signals in the ^1^H and ^13^C NMR spectra indicate the presence of just one isomer, suggesting that either the d-Hum epimer is not distinguishable by NMR analysis (*cf.* Mosher acid analysis) or that only the l-enantiomer is incorporated into the peptide.

Installation of the catalytic iridium center was accomplished on the resin-bound heptapeptide by treatment with Ag_2_O to form the putative carbene silver intermediate, followed by transmetalation with [IrCl_2_Cp*]_2_.§§Attempts to metallate the apo-peptides in the liquid-phase and detached from the resin have failed so far, irrespective of whether the heptapeptide was used or **Fmoc-Hum** without additional amino acids. After incubation for 24 h, the metallopeptide was cleaved from the resin with TFA, a procedure that is applicable because the Ir–C_NHC_ bond is remarkably stable under acidic conditions. While this procedure was successful for most palindromic sequences, those with X = Val, Trp, or His gave lower yields, the latter presumably because of interference of the heteroaromatic side chain with the metal center. Moreover, the procedure failed when Cys or Met were used as amino acids, which was attributed to the strong affinity of Ag to sulfur ligands and therefore a preferred reactivity of Ag_2_O with these side chains rather than the Hum.

Analysis by LC–MS of crude samples after resin cleavage revealed only partial metalation, which was attributed to partial hydrolytic Ir–C bond scission in the resin cleavage step due to the presence of TFA rather than to an incomplete metalation. Revers phase HPLC purification yielded the pure peptidic scaffolds **AXAḦAXA–Ir** (LC–MS). Analysis by HR-MS revealed a most significant signal for [**AXAḦAXA–Ir** –2Cl]^2+^ for all peptides except for the Lys-containing heptamer, for which *z* = 3 due to the basic side chain (†). The NMR spectra of these systems were consistently very broad, though they clearly revealed the Cp* proton signals around 1.6 ppm in the correct integral ratio. Depending on the amino acid sequence, this signal is split in two or more different signals, which however coalesce to a single resonance at 50 °C. Therefore, the different Cp* signals were attributed to distinct rotamers that show restricted interconversion due to the metal fragment bound to Hum, rather than epimers.

Since NHC iridium complexes are established hydrogenation catalysts, we investigated the activity of this minimalistic peptidic scaffolds in catalytic hydrogenation in citrate buffer at pH 3. All **AXAḦAXA–Ir** constructs showed activity in the hydrogenation of acetophenone as model substrate, albeit without any enantiomeric excess (ee < 3%). Reminiscent to metalloenzymes, the activity of the heptapeptide shows considerable dependence on the type of amino acids in close proximity to the active site (Table 1, entries 1–18). For example, there is an order of magnitude difference between X = R (TOF = 3 h^−1^) and X = Y (TOF = 30 h^−1^; entry 11 *vs.* 8).¶¶Chiral GC analysis indicated that the benzyl alcohol was obtained as a racemate in all catalytic runs, which can be rationalized by the fact that the catalytic site is extremely exposed in all modelled structures. Some general trends from this initial activity screen can be deduced. For example, aromatic side chains (X = F, Y) have a positive effect on the catalytic activity, possibly because of suitable interactions with the substrate. Potentially coordinating side chains reduce the activity (X = H, K) or induce an induction period (X = E, but not D, probably because the additional CH_2_ group in E enables metal binding). Bulkiness of the side chain has some inhibiting effect (S *vs.* T, N *vs.* Q), yet this effect was not observed in hydrophobic side chains. These variations indicate that these mini scaffolds can be tailored despite their small size. Moreover, the oligopeptide backbone is highly relevant for the catalytic activity as the iridium-complex **1** bound to protected **Hum** or a simple NHC iridium analogue (**2**) showed much lower activity and reached a modest <20% conversion after 24 h (*cf* >90% conversion with selected mini scaffolds with X = A, F, G, S, Y) under identical reaction conditions (entries 20 and 21, Fig. 1, Fig. S1, ESI†). The metal precursor [IrCl_2_Cp*]_2_ does not show any detectable activity (entry 19).

**Table 1:** Catalytic hydrogenation of acetophenone^a^

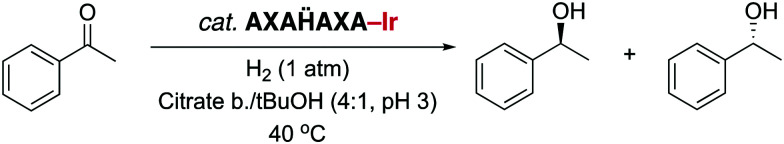				
Entry	Category	**X** b	TOF_max_^c^ (h^−1^)	Conversion^d^ (%)
1	Unfunctionalized	**G**	20	95
2		**A**	17	91
3		**V**	18	86
4	Hydrophobic	**P**	18	77
5		**I**	16	83
6		**L**	13	82
7		**F**	24	94
8	Aromatic	**Y**	30	96
9		**W**	12	60
10		**H**	n.d.	<2
11	+Charge	**R**	3	40
12		**K**	9	81
13	−Charge	**D**	17	80
14		**E**	17	78
15	Polar uncharged	**S**	17	94
16		**N**	19	88
17		**T**	7	82
18		**Q**	10	70
19		[IrCl2Cp*]2	n.d.	<2
20	Reference	**1**	2	18
21		**2**	1	19

a General reaction conditions: acetophenone (10 μmol), [Ir] (0.1 μmol, 1 mol%), citrate buffer pH 3/*t*BuOH (1 mL, 4 : 1 v/v), 40 °C, H_2_ atmosphere.

b X = M, C not evaluated since metalation did not proceed.

c TOF_max_ determined from the rate at the steepest section for each entry, n.d. not determined.

d Conversion determined by GC, all ee < 3%.

**Fig. 1:**
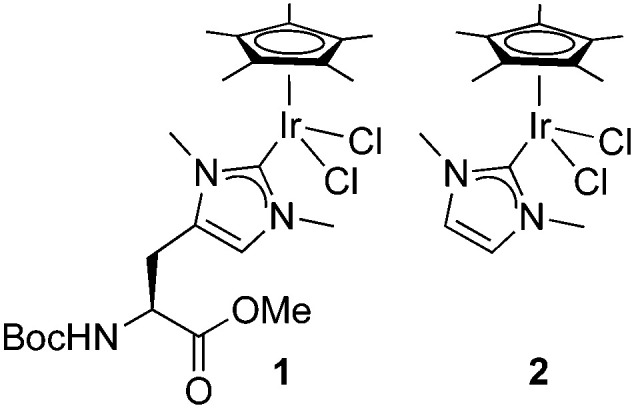
Schematic of small molecule catalysts **1** and **2**.

To shed some light on the structural implications imparted by the variations in the amino acid sequence, molecular dynamics simulations of all 18 catalytically active heptapeptides were performed (†). The peptides were modelled initially with His rather than the Hum–Ir in central position. The Hum–Ir was then constructed as a constrained entity based on reported crystal structure data using the UCSF Chimera software. The conformational changes of the metal-free heptapeptides in 20 ns intervals indicate that despite their short size, most heptapeptides adopt stable secondary structures (Fig. S2–S4 and see also CD spectra in S5, ESI†).^17^ While neither the root mean square deviation of the conformational changes of the heptapeptide nor the number of clusters from the simulations correlate with the catalytic activity, the histidine exposure revealed a positive correlation with the TOF_max_ (Fig. S6 and Table S1, ESI†). For example, AYAHAYA with two Tyr adopts secondary structures in which the central His is well-exposed for solvent and substrate accessibility. Closer inspection of the structures indicates a conformationally stable interaction between one phenol side chain and the central His unit, also when bound to Ir in the AYAḦAYA-Ir variant (Fig. 2). Similar His/Tyr interactions have been noted previously.^18^ The correlation between His exposure and catalytic activity for subsets of the heptapeptides (Fig. S6, ESI†) constitutes a key concept of metalloenzyme engineering and offers an attractive approach for further optimization of the catalytic performance, especially upon increasing the length of the peptide sequence.

**Fig. 2:**
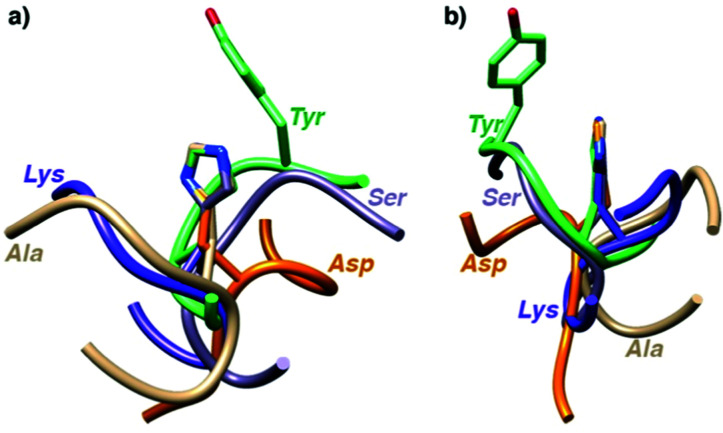
Superimposition of most stable conformations deduced from molecular dynamics simulation of representative heptapeptides AXAHAXA containing a central His and X = Tyr, Ser, Gly, Val, and Arg from two different angles (a and b). The phenol residue of Tyr remains in close proximity of the His (see ESI† for time-deconvoluted movies of conformational changes for two metallopeptides **AXAḦAXA–Ir** with X = Tyr and Lys).

In summary, a versatile pre-carbene amino acid was developed, and was incorporated into oligopeptides to generate minimalistic protein-like assemblies containing a metal–carbene active site. The introduction of an Fmoc-protecting group on the N-terminus of the amino acid allowed for the use of SPPS as synthetic approach for *de novo* peptide synthesis. Metalation was performed successfully with an Ir-precursor on the resin-bound peptides, followed by cleavage of the organometallic bioconjugate from the resin, which generated active scaffolds for hydrogenation reactions. This system shows characteristics which mimics natural enzymes including a direct dependence of the catalytic activity on proximal amino acid side chains, which provides opportunities for evolution. While the heptapeptide sequence is short to impose unique secondary structures, molecular dynamics identified for some of the peptide sequences clear energy minima. The established potential of SPPS to prepare also longer structures and the availability of mixed de-novo/ligation strategies offers approaches to implement structural elements and to tailor further the selectivity of this bio-inspired catalyst. A particularly appealing aspect of the concept disclosed here is the opportunity to combine an enzymatic scaffold with carbene complexes and their vast array of catalytic applications. This combination considerably expands the toolbox of biocatalysis and allows to design systems for abiotic transformations.

Conceptualization (F. P., M. A.), data curation (K. L., M. P., I. F., D. R.) data analysis, validation, visualization, and writing (K. L., M. P., D. R. P., F. P., M. A.).

The authors gratefully acknowledge generous financial support from the ERC (CoG 615653) and the Swiss National Science Foundation (20020_182663 and 200021_192274).

## Conflicts of interest

There are no conflicts to declare.

## Supplementary Material

CC-057-D1CC03158A-s001

CC-057-D1CC03158A-s002

CC-057-D1CC03158A-s003

## References

[cit1] Natoli S. N., Hartwig J. F. (2019). Acc. Chem. Res..

[cit2] Kan S. B. J., Lewis R. D., Chen K., Arnold F. H. (2016). Science.

[cit3] Green A. P., Hayashi T., Mittl P. R. E., Hilvert D. (2016). J. Am. Chem. Soc..

[cit4] Schwizer F., Okamoto Y., Heinisch T., Gu Y., Pellizzoni M. M., Lebrun V., Reuter R., Köhler V., Lewis J. C., Ward T. R. (2018). Chem. Rev..

[cit5] Mayer C., Gillingham D. G., Ward T. R., Hilvert D. (2011). Chem. Commun..

[cit6] Basauri-Molina M., Riemersma C. F., Würdemann M. A., Kleijn H., Klein Gebbink R. J. M. (2015). Chem. Commun..

[cit7] Nelson D. J., Nolan S. P. (2013). Chem. Soc. Rev..

[cit8] Planchestainer M., Ségaud N., Shanmugam M., McMaster J., Paradisi F., Albrecht M. (2018). Angew. Chem., Int. Ed..

[cit9] Monney A., Alberico E., Ortin Y., Müller-Bunz H., Gladiali S., Albrecht M. (2012). Dalton Trans..

[cit10] Hannig F., Kehr G., Fröhlich R., Erker G. (2005). J. Organomet. Chem..

[cit11] Xu G., Gilbertson S. R. (2005). Org. Lett..

[cit12] Prinz M., Grosche M., Herdtweck E., Herrmann W. A. (2000). Organometallics.

[cit13] Petronilho A., Woods J. A., Müller-Bunz H., Bernhard S., Albrecht M. (2014). Chem. – Eur. J..

[cit14] Schmitt F., Donnelly K., Muenzner J. K., Rehm T., Novohradsky V., Brabec V., Kasparkova J., Albrecht M., Schobert R., Mueller T. (2016). J. Inorg. Biochem..

[cit15] Kuroda H., Kubo S., Chino N., Kimura T., Sakakibara S. (1992). Int. J. Pept. Protein Res..

[cit16] Abd-Elgaliel W. R., Gallazzi F., Lever S. Z. (2007). J. Pept. Sci..

[cit17] MD simulations were performed on the AXAHAXA sequence with a natural His as the central amino acid (see ESI† for details). Replacing H with the Ḧ–Ir unit and rigidly fixed atomic positions of the ligands and metal center for four variants AXAḦAXA-Ir with X = K, H, W and Y did not reveal any apparent changes in the behavior of the secondary structure or solvent exposure (Fig. S7)

[cit18] Albada H. B., Wieberneit F., Dijkgraaf I., Harvey J. H., Whistler J. L., Stoll R., Metzler-Nolte N., Fish R. H. (2012). J. Am. Chem. Soc..

