# Supplementary material for: Minimalistic peptidic scaffolds harbouring an artificial carbene-containing amino acid modulate reductase activity
Source: Chem Commun (Camb). 2021 Aug 6;57(72):9068–71. doi: 10.1039/d1cc03158a (PMC8427656; doi:10.1039/d1cc03158a)

Electronic Supplementary Material (ESI) for ChemComm.
This journal is © The Royal Society of Chemistry 2021

Electronic Supporting Information

Belonging to

Minimalistic peptidic scaffolds harbouring an artificial carbene-containing amino acid modulate reductase activity

Karst Lenzen,[a] Matteo Planchestainer,[a] Isabelle Feller,[a] David Roura Padrosa,[a] Francesca Paradisi *[a] and Martin Albrecht *[a]

Department of Chemistry, Biochemistry & Pharmaceutical Sciences, University of Bern, Freiestr. 3, 3012 Bern, Switzerland

E-mail: Francesca.paradisi@dcb.unibe.ch, martin.albrecht@dcb.unibe.ch

Table of Contents:

1. Experimental Procedures S2
   1.1. Synthesis of Fmoc-Hum-OH S3
   1.2. General SPPS procedure S6
   1.3. General procedure for the preparation of AXAӒAXA–Ir S12
   1.4. Catalytic experiments S18
2. Conformational Analysis by Molecular Dynamics Simulations and CD Spectroscopy S19
3. References S26
4. Spectra and MS Data S27

1. Experimental Procedures

General considerations. Boc-Hum-OMe,[S1] complex **1**,[S2] complex **2**,[S3] and (S)-(–)-Mosher's acid chloride[S4] were prepared according to literature procedures. All Fmoc-protected amino acids were obtained from Iris Biotech GmbH. All other reagents were commercially available and used as received. NMR spectra were recorded at 298 K (unless stated otherwise) on Bruker spectrometers operating at 300 or 400 MHz (^{1}H NMR) and 75 or 100 MHz (^{13}C{^{1}H} NMR), respectively. Chemical shifts (δ in ppm and coupling constants *J* in Hz) were referenced to residual solvent signals (^{1}H and ^{13}C). The purity of the bulk samples of the complexes has been established by NMR spectroscopy and by elemental analysis, which were performed at the Microanalytic Laboratory, University of Bern, using a Thermo Scientific Flash 2000 CHNS-O elemental analyser. HR-MS was carried out with a Thermo Scientific LTQ Orbitrap XL (ESI-TOF) spectrometer. Preparative RP-HPLC was performed on an Agilent 1260 Infinity II preparative LC system using an Agilent 5 Prep-C18 100 × 30.0 mm column (SN: 552624). LC-MS data were collected on a LCQ Fleet Ion Trap mass spectrometer (Thermo Scientific, San Jose, CA, USA).

CD spectra were measured using a Jasco J-715 spectrometer at room temperature equipped with a PS-150J power supply. All experiments were measured using a Hellma High Precision cell 0.2 cm cuvette. A stock solution (1 mg mL^{-1}) of each peptide was freshly prepared in miliQ water. For the measurement, the peptides were diluted to a concentration from 0.04 to 0.1 mg mL^{-1} in 10 mM phosphate buffer pH 8. The range of measurement was 185–260 nm with a scan rate of 20 nm/min, pitch 05 nm, response 16 sec and band 1 nm. The nitrogen flow was always kept above 8.5 L min^{-1}. A blank was recorded under the same conditions. Between measurements, the cuvettes were washed twice with 1 M HCl and twice with the buffer before adding the sample. Data was treated using the CAPITO webservice at https://capito.uni-jena.de.

1.1. Synthesis of Fmoc-Hum-OH

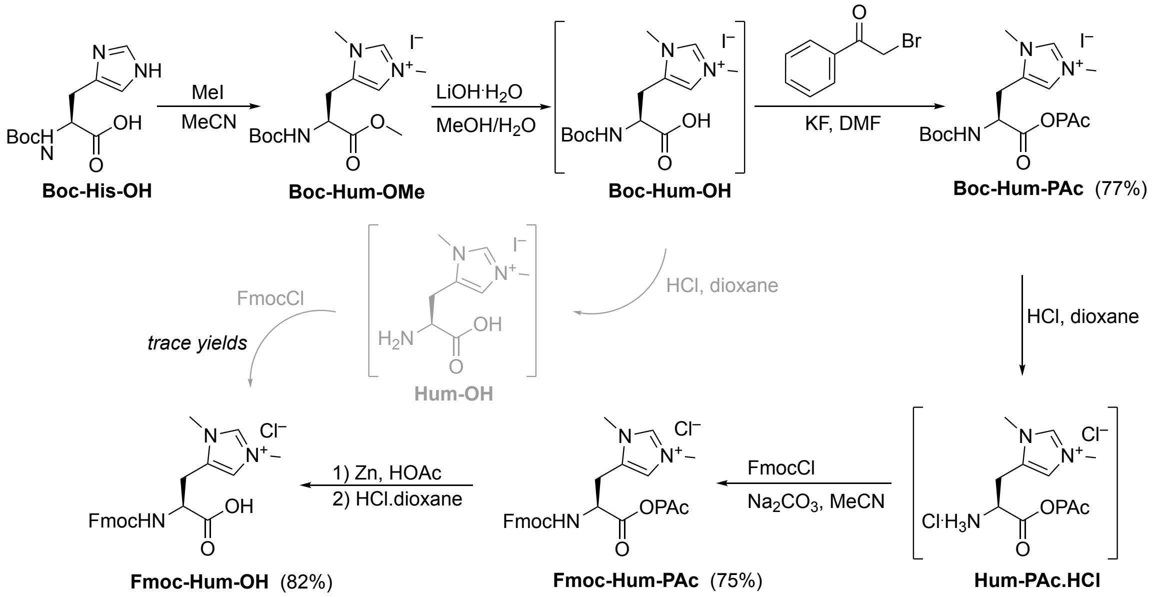

Synthesis of Boc-Hum-OMe. L-Histidine methyl ester dihydrochloride (5.0 g, 20 mmol), Na_2CO_3 (8.3 g, 78 mmol) were dissolved in MeCN (200 mL). MeI (12.5 mL, 200 mmol) was added and stirred 24 h at 40 ºC. The mixture was dried and dissolved in CH_2Cl_2 (100 mL). The mixture was filtered and evaporated to dryness to yield **Boc-Hum-OMe** (8.25 g, 97%) as a white solid. The spectral data are consistent with literature data.[1]

Synthesis of Boc-Hum-OPAc. Boc-Hum-OMe (8.25 g, 19.4 mmol) was dissolved in MeOH/H_2O (2:1, 50 mL) and LiOH.H_2O (855 mg, 20.4 mmol) was added. The mixture was stirred for 3 h at RT, neutralized with 4 M HCl and evaporated to dryness, yielding **Boc-Hum-OH** as a colorless oil, which was immediately used without further purification. ^{1}H NMR (D_2O, 300 MHz) δ 8.58 (s, 1H, NCHN), 7.23 (m, 1H, NCHC), 4.19 (dd, $^3J_{HH}$ = 8.8, 4.8 Hz, 1H, C*H*CH$_2$), 3.80, 3.80 (2 × s, 3H, NCH$_3$), 3.19 (dd, $^2J_{HH}$ = 15.5 Hz, $^3J_{HH}$ = 4.7 Hz, 1H, CHC*H$_2$*), 2.97 (dd, $^2J_{HH}$ = 15.5 Hz, $^3J_{HH}$ = 8.8 Hz, 1H, CHC*H$_2$*), 1.36 (s, 9H, C(CH$_3$)$_3$). 2-Bromoacetophenone (4.05 g, 20.4 mmol) and KF (2.82 g, 48.5 mmol) were added to **Boc-Hum-OH** and dissolved in DMF (20 mL) and stirred for 4 h at rt. CH_2Cl_2 (200 mL) was added and the mixture was filtered through Celite. The filtrate was washed with brine (2x 100 mL) and H_2O (2x 100 mL). The organic phase was dried over Na_2SO_4, filtered and concentrated to 4 mL. Et_2O (50 mL) was added to induce precipitation. The precipitate was separated by centrifugation. The precipitation from CH_2Cl_2 and Et_2O was repeated thrice and the precipitate was subsequently dried in vacuo to yield **Boc-Hum-OPAc** as a light yellow solid (8.74 g, 77%, yield corrected for traces of DMF (~1 eq) which were unproblematic for subsequent transformations). ^{1}H NMR ($CDCl_3$, 400 MHz) δ 9.89 (s, 1H, C$_ε$H), 7.89–7.86 (m, 2H, H$_{Ph}$), 7.62–7.57 (m, 1H, H$_{Ph}$), 7.49–7.45 (m, 3H, H$_{Ph}$ + C$_δ$H), 5.66 (d, $^3J_{HH}$ = 6.4 Hz, 1H, NH), 5.60, 5.44 (2 × d, $^2J_{HH}$ = 16.5 Hz, 1H, CH$_2$O), 4.69 (q, $^3J_{HH}$ = 6.5 Hz, 1H, C$_α$H),

3.97, 3.92 (2 × s, 3H, NCH_3), 3.37, 3.30 (2 × dd, $^2J_{HH}$ = 15.8 Hz, $^3J_{HH}$ = 6.0 Hz, 1H, $C_\beta H_2$), 1.38 (s, 9H, $C(CH_3)_3$), $^{13}C\{^1H\}$ NMR ($CDCl_3$, 100 MHz) δ 191.6 (Ph–CO), 169.8 (CH*C*OO), 155.3 (NHCOO), 137.4 ($C_\varepsilon H$), 134.4 (CH_{Ph}), 133.6 (C_{Ph}), 131.1 (C_γ), 129.1, 127.9 (2 × CH_{Ph}), 122.3 ($C_\delta H$), 80.9 (*C*$(CH_3)_3$), 67.6 (OCH_2), 52.4 ($C_\alpha H$), 36.8, 34.3 (2 × NCH_3), 28.3 ($C(CH_3)_3$), 26.6 ($C_\beta H_2$). HRMS (ESI^+): calcd for $[M – I]^+$ 402.2023, found 402.2011.

Synthesis of Fmoc-Hum-OPAc. Boc-Hum-OPAc (4.0 g, 7.6 mmol) was dissolved in HCl in dioxane (25 mL, 4 M, 0.10 mol) and stirred for 2 h at rt. A biphasic system formed and the top layer was pipetted off. The oily residue was washed with Et_2O (4 × 30 mL) and dried to yield **Hum-OPAc** as an orange solid, which was immediately used without removal of residual dioxane. ^1H NMR (CD_3CN, 400 MHz) δ 9.13 (NH_3Cl), 8.58 (s, 1H, $C_\varepsilon H$), 7.97–7.95 (m, 2H, H_{Ph}), 7.72 (s, 1H, $C_\delta H$), 7.68 (tt, $^3J_{HH}$ = 7.1 Hz, $^4J_{HH}$ = 1.3 Hz, 1H, H_{Ph}), 7.56–7.52 (m, 2H, H_{Ph}), 5.71, 5.51 (2 × d, $^2J_{HH}$ = 16.8 Hz, 1H, CH_2O), 4.66–4.60 (m, 1H, $C_\alpha H$), 3.90, 3.82 (2 × s, 3H, NCH_3), 3.79–3.71, 3.64–3.58 (2 × m, 1H, $C_\beta H_2$), $^{13}C\{^1H\}$ NMR (CD_3CN, 100 MHz) δ 192.8 (Ph–CO), 168.7 (CH*C*OO), 155.3 (NHCOO), 137.9 ($C_\varepsilon H$), 135.4, 134.7, 130.0, 129.8, 129.0 (4 × C_{Ph} + C_γ), 125.1 ($C_\delta H$), 69.3 (OCH_2), 52.4 ($C_\alpha H$), 37.1, 35.1 (2 × NCH_3), 24.7 ($C_\beta H_2$). HRMS (ESI^+): calcd for $[M – H – 2Cl]^+$ 302.1505, found 302.1486. **Hum-OPAc** was dissolved in MeCN (60 mL) and FmocCl (3.9 g, 15.2 mmol) and Na_2CO_3 (0.81 g, 7.6 mmol) were added. The mixture was stirred 16 h at room temperature and evaporated to dryness. The residue was dissolved in CH_2Cl_2 (50 mL) and filtered. The filtrate was concentrated and further purified by precipitation (3 × CH_2Cl_2/Et_2O). The residue was dissolved in CH_2Cl_2 (50 mL) and washed with brine (3 × 50 mL). The organic layer was dried over Na_2SO_4, filtered and evaporated to dryness, yielding **Fmoc-Hum-OPAc** as a light orange solid (3.2 g, 75%). ^1H NMR ($CDCl_3$, 400 MHz) δ 9.75 (s, 1H, $C_\varepsilon H$), 7.88 (d, $^3J_{HH}$ = 7.0 Hz, 2H, H_{Ph}), 7.75–7.74 (m, 2H, H_{Ph}), 7.64–7.60 (m, 3H, H_{Ph}), 7.49 (t, $^3J_{HH}$ = 7.8 Hz, 2H, H_{Ph}), 7.41–7.37 (m, 3H, H_{Ph}), 7.33–7.29 (m, 3H, H_{Ph} + $C_\delta H$), 6.24 (broad d, $^3J_{HH}$ = 7.3 Hz, 1H, NH), 5.58, 5.37 (2 × d, $^2J_{HH}$ = 16.4 Hz, 1H, $CH_2C(O)Ph$), 4.78–4.77 (m, 1H, $C_\alpha H$), 4.45–4.37 (m, 2H, O*CH₂*CH), 4.19 (t, $^3J_{HH}$ = 6.6 Hz, 1H, OCH_2*CH*), 3.91, 3.87 (2 × s, 3H, NCH_3), 3.41–3.31 (2 × m, 1H, $C_\beta H_2$), $^{13}C\{^1H\}$ NMR ($CDCl_3$, 100 MHz) δ 191.6 (Ph–CO), 169.7 (CH*C*OO), 156.1 (NHCOO), 143.7, 143.6, 141.4 (3 × C_{Ph}), 137.6 ($C_\varepsilon H$), 134.6, 133.6, 131.1, 129.2, 128.0, 128.0, 127.4, 127.3, 125.3, 122.3, 120.2 (11 × (C_{Ph} + C_γ + $C_\delta H$)), 67.6 (*C*$H_2C(O)Ph$), 67.4 (CH*C*H_2O), 52.8 ($C_\alpha H$), 47.2 (OCH_2*C*H), 37.0, 34.5 (2 × NCH_3), 26.4 ($C_\beta H_2$). HRMS (ESI^+): calcd for $[M – Cl]^+$ 524.2180, found 524.2166.

Synthesis of Fmoc-Hum-OH. Fmoc-Hum-OPAc (3.2 g, 4.6 mmol) was dissolved in 90% HOAc (25 mL) and Zn powder (10.0 g, 153 mmol) was added. The mixture was stirred vigorously for 4 h at room temperature and subsequently filtered through Celite. The filtrate was dried in vacuo, dissolved in CH_2Cl_2 (10 mL) and filtered. The filtrate was concentrated and precipitated with Et_2O (20 mL). The precipitation was repeated twice and the precipitate was dried. HCl in dioxane (5 mL, 4 M, 20 mmol) was added and the mixture was stirred for 1 h at RT and subsequently precipitated with Et_2O (100 mL)

and centrifuged. The residue was washed with Et_2O (2 × 50 mL) and CH_2Cl_2 (2 × 25 mL) and evaporated to dryness yielding **Fmoc-Hum-OH** (1.66 g, 82%) as a white solid. An analytically pure sample was obtained by reverse-phase HPLC column chromatography. ^{1}H NMR (CD_3CN, 400 MHz) δ 8.76 (s, 1H, $C_εH$), 7.81 (d, $^3J_{HH}$ = 7.5 Hz, 2H, H_{Ph}), 7.64 (t, $^3J_{HH}$ = 7.3 Hz, 2H, H_{Ph}), 7.40 (t, $^3J_{HH}$ = 7.5 Hz, 3H, H_{Ph}), 7.34–7.29 (m, 3H, H_{Ph} + $C_δH$), 6.15 (broad d, $^3J_{HH}$ = 8.5 Hz, 1H, NH), 4.53 (dd, $^3J_{HH}$ = 4.8, 9.1 Hz, 1H, $C_αH$), 4.44–4.33 (m, 2H OCH_2CH), 4.20 (t, $^3J_{HH}$ = 6.7 Hz, 1H, OCH_2C*H*), 3.79, 3.77 (2 × s, 3H, NCH_3), 3.34–3.28 (m, 1H, $C_βH_2$), 3.08 (dd, $^2J_{HH}$ = 15.9 Hz, $^3J_{HH}$ = 9.1 Hz, 1H, $C_βH_2$). $^{13}C\{^1H\}$ NMR (CD_3CN, 100 MHz) δ 171.7 (CH*C*OO), 144.9, 142.2 (2 × C_{Ph}), 137.3 ($C_εH$), 132.7 ($C_γ$), 128.8 128.2, 126.1 (3 × CH_{Ph}), 122.7 ($C_δH$), 121.0 (CH_{Ph}), 67.6 (OCH_2), 53.0 ($C_αH$), 48.0 (OCH_2C*H*), 36.9, 34.5 (2 × NCH_3), 26.4 ($C_βH_2$). HRMS (ESI$^+$): calcd for $[M - Cl]^+$ 406.1761, found 406.1752. Calcd for $C_{23}H_{24}ClN_3O_4·CF_3COOH$: C, 54.01; H, 4.53; N, 7.56. Found: C, 54.43; H, 4.10; N, 7.06.

Synthesis of Hum-OH. Boc-Hum-OMe (747 mg, 1.76 mmol) was dissolved in MeOH/H_2O (2:1, 6 mL) and $LiOH.H_2O$ (77 mg, 1.84 mmol) was added. The mixture was stirred for 3 h at RT, neutralized with 4 M HCl and evaporated to dryness. HCl in dioxane (15 mL, 4 M, 60 mmol) was added and the mixture was stirred for 16 h at RT. Et_2O was added to induce precipitation and the supernatant was removed. The residue was dried to yield crude **Hum-OH** (546 mg, >100%), as a light orange and very hygroscopic powder). ^{1}H NMR (D_2O, 400 MHz) δ 8.71 (s, 1H, $C_εH$), 7.45 (s, 1H, $C_δH$), 4.31 (dd, $^3J_{HH}$ = 6.9, 6.9 Hz, 1H, $C_αH$), 3.87, 3.85 (2 × s, 3H, NCH_3), 3.45, 3.37 (2 × dd, $^2J_{HH}$ = 16.3 Hz, $^3J_{HH}$ = 6.9 Hz, 1H, $C_βH_2$). $^{13}C\{^1H\}$ NMR (D_2O, 100 MHz) 175.4 (COO), 142.0 ($C_εH$), 133.7 ($C_γ$), 127.3 ($C_δH$), 56.3 ($C_αH$), 40.5, 38.1 (2 × NCH_3), 28.7 ($C_βH_2$). HRMS (ESI$^+$): calcd for $[M - Cl]^+$ 184.1086, found 184.1092.

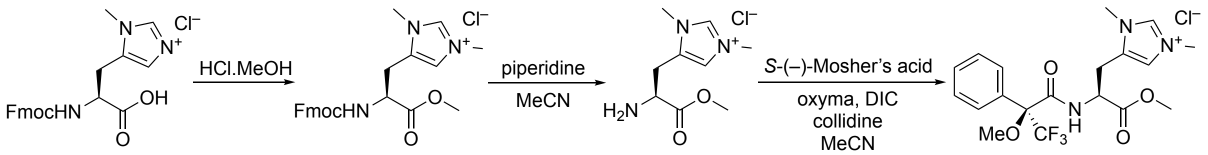

Synthesis of Mosher-Hum-OMe. Fmoc-Hum-OH (50 mg, 0.094 mmol) was dissolved in a methanolic HCl solution (5 mL, 0.5 M in MeOH, 2.5 mmol) and stirred for 16 h at RT. The volatiles were evaporated and the residue was dried in vacuo for 3 h to yield **Fmoc-Hum-OMe**. A 20% solution of piperidine in MeCN (5 mL) was added and the mixture was stirred for 2 h. Purification was performed by RP-HPLC to yield, after drying, **Hum-OMe**. The residue was dissolved in MeCN (5 mL) and oxyma (20 mg 0.14 mmol), collidine (18 µL, 0.14 mmol), *N,N'*-diisopropylcarbodiimide (DIC; 22 µL, 0.14 mmol) and Mosher's acid (30 mg, 0.13 mmol) were added and stirred for 5 h at RT. The mixture was purified by RP-HPLC and dried to yield a 95:5 epimeric mixture of **Mosher-Hum-OMe**, together with traces of oxyma, which was not further purified. *Major epimer (***L-Hum**): ^{1}H NMR (CD_3CN, 300 MHz)

δ 8.23 (s, 1H, $C_{\varepsilon}H$), 7.72 (d, $^3J_{HH}$ = 9.4 Hz, 1H, NH), 7.48–7.41 (m, 5H, CH_{Ph}), 6.88 (s, 1H, $C_{\delta}H$), 4.89–4.81 (m, 1H, $C_{\alpha}H$), 3.74, 3.65, 3.58 (3 × s, 3H, NCH_3 + $COOCH_3$), 3.52 (q, $^4J_{HF}$ = 1.8 Hz, 3H, OCH_3), 3.26 (dd, $^2J_{HH}$ = 16.0 Hz, $^3J_{HH}$ = 5.0 Hz, 1H, $C_{\beta}H_2$), 3.03 (dd, $^2J_{HH}$ = 16.0 Hz, $^3J_{HH}$ = 10.1 Hz, 1H, $C_{\beta}H_2$). ^{19}F NMR (CD_3CN, 282 Hz) δ –69.6 (s, CF_3). *Minor epimer (***D-Hum***)*: ^{1}H NMR (CD_3CN, 300 MHz) δ 8.32 (s, 1H, $C_{\varepsilon}H$), 7.05 (s, 1H, $C_{\delta}H$), too dilute for full analysis. ^{19}F NMR (CD_3CN, 282 Hz) δ –69.9 (s, CF_3).

1.2. General SPPS procedure

Amino acid coupling and synthesis **resin-AXAḦAXA***:* A functionalized Wang resin (Fmoc-Ala-Wang resin (100 mg, 0.4–0.8 mmol/g) was placed in a 10 mL polypropylene syringe, equipped with frit and a stopper. The resin was swollen for 5 minutes in CH_2Cl_2 (5 mL). The CH_2Cl_2 was removed and 20% piperidine in DMF (7 mL) was added to the resin and shaken for 20 min. Shaking was performed by attaching the closed syringe to a rotating axis. After filtration, the resin was washed with NMP (2 × 5 mL), MeOH (2 × 5 mL) and CH_2Cl_2 (2 × 5 mL) and swollen for an additional 3 min in CH_2Cl_2 (5 mL) and filtered. A coupling cocktail was prepared by dissolving Fmoc-AA-OH (0.20 mmol), oxyma (28 mg, 0.20 mmol), collidine (26 µL, 0.20 mmol) and DIC (31 µL, 0.20 mmol) in NMP (5 mL) and was subsequently added to the resin. After addition of CH_2Cl_2 (3 mL), the mixture was shaken for 2 h, except for the coupling with **6** and the two Fmoc-AA-OH following **6**, which were shaken for 5–6 h. After filtration the resin was washed with NMP (2 × 5 mL), MeOH (2 × 5 mL) and CH_2Cl_2 (2 × 5 mL) and swollen for an additional 3 min in CH_2Cl_2 (5 mL). The completion of the reaction was checked using the TNBS (2,4,6-trinitrobenzene sulfonic acid) test, by taking a sample from the suspension in CH_2Cl_2 (10 µL) and adding it to a 1.5 mL microcentrifuge tube. Subsequently, 1% TNBS in DMF (25 µL) and 10% DIPEA in DMF (50 µL) were added and the mixture was mixed for 30 s. MeOH (0.5 mL) was added and the color of the beads was checked (colorless is negative, orange-red is positive). When negative, 20% piperidine in DMF (7 mL) was added to the resin and the coupling procedure was repeated for a subsequent amino acid coupling. After the final piperidine-mediated Fmoc-deprotection, the peptide was treated with a solution of acetic acid anhydride / CH_2Cl_2 (1:1 v/v, 7 mL) for 10 min and filtered. This procedure was repeated once, and the resin was subsequently washed with NMP (2 × 5 mL), MeOH (2 × 5 mL) and CH_2Cl_2 (2 × 5 mL) to yield **resin-AXAḦAXA**.

Peptide cleavage and preparation of apo-peptides **AXAḦAXA***:* A solution of TFA/H_2O/TIS (7 mL, 94:5:1, v/v/v) was added to the resin-bound peptide **resin-AXAḦAXA** and the mixture was shaken for 4 h. For the cleavage of the Cys-containing peptide **resin-ACAḦACA**, the solution was TFA/H_2O/DTT/TIS (7 mL, 94:2.5:2.5:1, v/v/w/v). The supernatant was collected by filtration and cold *t*BuOMe (40 mL) was added and the mixture was left for 30 min at –20 °C. The cold mixture was centrifuged, the solids were collected and further purified by gradient preparative RP-HPLC column

chromatography (all solvents are acidified with 0.1% TFA; 5 min at 100% H_2O, 10 min gradient to 50% H_2O/50% MeCN, 5 min gradient to 100% MeCN, 5 min at 100% MeCN) and lyophilized to yield the peptide **AXAḦAXA** as a white powder. Peptide integrity was assessed by HR-MS and purity by LC-MS (pp. S25).

Synthesis of AGAḦAGA. Following the SPPS general procedure, using half the quantities stated for the Fmoc-Ala-Wang resin and the coupling cocktail. White powder (8 mg, 54±16%). ^{1}H NMR (H_2O:D_2O, 9:1, 300 MHz) δ 8.48 (s, 1H, $C_\varepsilon H_{\text{ Hum}}$), 8.40–8.0 (m, 7H, 7 × CONH), 7.14 (m, 1H, $C_\delta H_{\text{ Hum}}$), 4.61–4.56 (m, 1H, $C_\alpha H_{\text{ Hum}}$), 4.32–4.11 (m, 4H, 4 × $C_\alpha H_{\text{ Ala}}$), 3.84, 3.79 (2 × s, 2H, $CH_{2\text{ Gly}}$), 3.70–3.68 (m, 6H, 2 × NCH_3), 3.18–3.13, 3.03–2.94 (2 × m, 1H, $CH_{2\text{ Hum}}$), 1.91 (s, 3H, $CH_{3\text{ acetyl}}$), 1.32–1.19 (m, 12H, 4 × $CH_{3\text{ Ala}}$). ^{13}C{^{1}H} NMR (H_2O:D_2O, 9:1, 75 MHz) δ 176.0, 176.0, 175.1, 175.0, 174.4, 174.4, 171.2, 171.1, 170.8 (9 × C=O), 136.6 ($C_{\gamma\text{ Hum}}$), 130.6 ($C_\delta H_{\text{ Hum}}$), 121.8 ($C_{\varepsilon\text{ Hum}}$), 51.1, 51.1, 49.9, 49.9, 49.8, 49.6, 48.5, 48.5 (8 × $C_\alpha H_{\text{ Ala}}$), 42.2, 42.1 (2 × $CH_{2\text{ Gly}}$), 36.5, 33.13 (2 × s, NCH_3), 24.8 ($CH_{2\text{ Hum}}$), 21.6 ($CH_{3\text{ acetyl}}$), 16.4, 16.3, 16.3, 16.1 (4 × $CH_{3\text{ Ala}}$). HRMS (ESI$^+$): calcd for $[M - Cl]^+$ 624.3105, found 624.3067

Synthesis of ASAḦASA. Following the SPPS general procedure, using half the quantities stated for the Fmoc-Ala-Wang resin and the coupling cocktail. White powder (20 mg, 85±15%). ^{1}H NMR (H_2O:D_2O, 9:1, 300 MHz) δ 8.52 (s, 1H, $C_\varepsilon H_{\text{ Hum}}$), 8.39 (d, $^3J_{HH}$ = 7.7 Hz, 1H, CONH), 8.30–8.22 (m, 5H, 5 × CONH), 8.14 (d, $^3J_{HH}$ = 5.8 Hz, 1H, CONH), 7.17 (s, $C_\delta H_{\text{ Hum}}$, 1H), 4.65–4.58 (m, 1H, $C_\alpha H_{\text{ Hum}}$) 4.38–4.16 (m, 6H, 6 × $C_\alpha H$), 3.82–3.76 (m, 4H, 2 × CH_2OH), 3.75, 3.72 (2 × s, 3H, NCH_3), 3.16 (dd, $^2J_{HH}$ = 16.0 Hz, $^3J_{HH}$ = 9.0 Hz, 1H, $C_\beta H_{2\text{ Hum}}$), 3.01 (dd, $^2J_{HH}$ = 16.0 Hz, $^3J_{HH}$ = 6.0 Hz, 1H, $C_\beta H_{2\text{ Hum}}$), 1.94 (s, 3H, $CH_{3\text{ acetyl}}$), 1.35–1.26 (m, 12H, $CH_{3\text{ Ala}}$), not resolved (2 × OH_{Ser}). ^{13}C{^{1}H} NMR (H_2O:D_2O, 9:1, 75 MHz) δ 176.3, 175.8, 175.0, 174.8, 174.5, 171.7, 171.3, 171.2 (8 × C=O), 136.7 ($C_\varepsilon H_{\text{ Hum}}$), 130.6 ($C_{\gamma\text{ Hum}}$), 121.8 ($C_\delta H_{\text{ Hum}}$), 61.2, 61.1 (2 × CH_2OH), 55.6, 55.6, 51.4, 50.2, 50.0, 50.0, 48.9 (7 × $C_\alpha H$), 35.7, 33.3 (2 × NCH_3), 25.0 ($C_\beta H_{2\text{ Hum}}$), 21.8 ($CH_{3\text{ acetyl}}$), 16.7, 16.5, 16.5, 16.4 (4 × $CH_{3\text{ Ala}}$). HRMS (ESI$^+$): calcd for $[M - Cl]^+$ 684.3317, found 684.3296.

Synthesis of ADAḦADA. Following the SPPS general procedure, using half the quantities stated for the Fmoc-Ala-Wang resin and the coupling cocktail. White powder (22 mg, 85±15%). ^{1}H NMR (H_2O:D_2O, 9:1, 300 MHz) δ 8.55 (s, 1H, $C_\varepsilon H_{\text{ Hum}}$), 8.4–8.0 (m, 7H, 7 × CONH), 7.19 (s, 1H, $C_\delta H_{\text{ Hum}}$), 4.66–4.60 (m, 3H, $C_\alpha H_{\text{ Hum}}$ + 2 × $C_\alpha H_{\text{ Asp}}$), 4.34–4.11 (m, 4H, 4 × $C_\alpha H_{\text{ Ala}}$), 3.77, 3.74 (2 × s, 3H, NCH_3), 3.19 (dd, $^2J_{HH}$ = 15.9 Hz, $^3J_{HH}$ = 9.2 Hz, 1H, $C_\beta H_{2\text{ Hum}}$), 3.04 (2 × dd, $^2J_{HH}$ = 15.9 Hz, $^3J_{HH}$ = 5.9 Hz, 1H, $C_\beta H_{2\text{ Hum}}$), 2.94–2.86, 2.81–2.70 (2 × m, 2H, 2 × $C_\beta H_{2\text{ Asp}}$), 1.96 (s, 3H, $CH_{3\text{ acetyl}}$), 1.37–1.26 (m, 12H, 4 × $CH_{3\text{ Ala}}$). ^{13}C{^{1}H} NMR (H_2O:D_2O, 9:1, 75 MHz) δ 175.9, 175.4, 174.8, 174.4, 174.4, 174.0, 173.7, 172.0, 171.7, 171.1 (8 × C=O + 2 × $COO_{\text{ Asp}}$), 136.6 ($C_\varepsilon H_{\text{ Hum}}$), 130.6 ($C_{\gamma\text{ Hum}}$), 121.7 ($C_\delta H_{\text{ Hum}}$), 51.2, 50.1, 49.9, 49.9, 49.8, 49.7, 48.6 (7 × $C_\alpha H$), 35.5 (NCH_3), 35.2, 34.9 (2 × $C_\beta H_{2\text{ Asp}}$), 33.1 (NCH_3), 24.7

($C_\beta H_{2\ Hum}$), 21.5 ($CH_{3\ acetyl}$), 16.3, 16.2, 16.1, 16.0 (4 × $CH_{3\ Ala}$). HRMS (ESI$^+$): calcd for $[M - Cl]^+$ 684.3317, found 684.3299. HRMS (ESI$^+$): calcd for $[M - Cl]^+$ 740.3215, found 740.3192.

Synthesis of AEAḦAEA. Following the SPPS general procedure, using half the quantities stated for the Fmoc-Ala-Wang resin and the coupling cocktail. White powder (17 mg, 76±24%). ^{1}H NMR (H_2O:D_2O, 9:1, 300 MHz) δ 8.57 (s, 1H, $C_\varepsilon H_{\ Hum}$), 8.4–8.0 (m, 7H, 7 × CONH), 7.23 (s, 1H, $C_\delta H_{\ Hum}$), 4.65 (dd, $^3J_{HH}$ = 9.0/6.0 Hz, 1H, $C_\alpha H_{\ Hum}$), 4.36–4.15 (m, 4H, 4 × $C_\alpha H_{\ Ala}$), 3.80, 3.77 (2 × s, 3H, NCH_3), 3.21 (dd, $^2J_{HH}$ = 15.9 Hz, $^3J_{HH}$ = 9.0 Hz, 1H, $C_\beta H_{2\ Hum}$), 3.06 (dd, $^2J_{HH}$ = 15.9 Hz, $^3J_{HH}$ = 6.0 Hz, 1H, $C_\beta H_{2\ Hum}$), 2.47 (dt, $^2J_{HH}$ = 14.0 Hz, $^3J_{HH}$ = 7.3 Hz, 4H, 2 × $C_\gamma H_{2\ Glu}$), 2.15–2.04 (m, 2H, 2 × $C_\beta H_{2\ Asp}$), 1.98 (s, 3H, $CH_{3\ acetyl}$), 1.98–1.88 (m, 2H, 2 × $C_\beta H_{2\ Glu}$), 1.39 (d, $^3J_{HH}$ = 7.3 Hz, 3H, $CH_{3\ Ala}$), 1.35–1.29 (m, 9H, 3 × $CH_{3\ Ala}$). $^{13}C\{^1H\}$ NMR (H_2O:D_2O, 9:1, 75 MHz) δ 176.9, 176.9, 176.1, 175.5, 174.8, 174.5, 174.3, 173.0, 172.8, 170.9 (8 × C=O + 2 × $COO_{\ Glu}$), 136.6 ($C_\varepsilon H_{\ Hum}$), 130.5 ($C_{\gamma\ Hum}$), 121.8 ($C_\delta H_{\ Hum}$), 52.6, 52.6, 51.1, 49.9, 49.7, 49.7, 48.6 (7 × C_αH), 35.5, 33.1 (2 × NCH_3), 29.7, 29.6 (2 × $C_\gamma H_{2\ Glu}$), 26.1, 25.8 (2 × $C_\beta H_{2\ Asp}$), 24.9 ($C_\beta H_{2\ Hum}$), 21.5 ($CH_{3\ acetyl}$), 16.4, 16.3, 16.3, 16.0 (4 × $CH_{3\ Ala}$). HRMS (ESI$^+$): calcd for $[M - Cl]^+$ 768.3528, found 768.3505.

Synthesis of AKAḦAKA. Following the SPPS general procedure, using half the quantities stated for the Fmoc-Ala-Wang resin and the coupling cocktail. White powder (28 mg, 83±17%). ^{1}H NMR (H_2O:D_2O, 9:1, 300 MHz) δ 8.58 (s, 1H, $C_\varepsilon H_{\ Hum}$), not resolved (m, 11H, 7 × CONH + 2 × NH_2), 7.23 (s, 1H, $C_\delta H_{\ Hum}$), 4.63 (dd, $^3J_{HH}$ = 5.9/9.1 Hz, 1H, $C_\alpha H_{\ Hum}$), 4.36–4.14 (m, 6H, 6 × C_αH), 3.80, 3.76 (2 × s, 3H, NCH_3), 3.20 (dd, $^2J_{HH}$ = 16.1 Hz, $^3J_{HH}$ = 9.2 Hz, 1H, $C_\beta H_{2\ Hum}$), 3.06 (dd, $^2J_{HH}$ = 16.1 Hz, $^3J_{HH}$ = 5.9 Hz, 1H, $C_\beta H_{2\ Hum}$), 2.98–2.94 (m, 4H, 2 × $C_\varepsilon H_{2\ Lys}$), 1.97 (s, 3H, $CH_{3\ acetyl}$), 1.84–1.60 (m, 8H, 2 × $C_\beta H_{2\ Lys}$ + 2 × $C_\delta H_{2\ Lys}$), 1.49–1.29 (m, 16H, 4 × $CH_{3\ Ala}$ + 2 × $C_\gamma H_{2\ Lys}$). $^{13}C\{^1H\}$ NMR (H_2O:D_2O, 9:1, 75 MHz) δ 176.2, 175.6, 174.9, 174.5, 174.2, 173.6, 173.4, 171.0 (8 × C=O), 136.6 ($C_\varepsilon H_{\ Hum}$), 130.5 ($C_{\gamma\ Hum}$), 121.8 ($C_\delta H_{\ Hum}$), 53.4, 53.1, 51.2, 49.9, 49.6, 49.6, 48.5 (7 × C_αH), 39.1, 39.1 (2 × $C_\varepsilon H_{2\ Lys}$), 35.5, 33.1 (2 × NCH_3), 30.3, 30.1 (2 × $C_\delta H_{2\ Lys}$), 26.3, 26.1 (2 × $C_\beta H_{2\ Lys}$), 24.9 ($C_\beta H_{2\ Hum}$), 22.0, 21.9 (2 × $C_\gamma H_{2\ Lys}$), 21.4 ($CH_{3\ acetyl}$), 16.6, 16.3, 16.3, 16.0 (4 × $CH_{3\ Ala}$). HRMS (ESI$^+$): calcd for $[M - Cl]^+$ 766.4575, found 766.4550.

Synthesis of AHAḦAHA. Following the SPPS general procedure, using half the quantities stated for the Fmoc-Ala-Wang resin and the coupling cocktail. White powder (18 mg, 73±7%). ^{1}H NMR (H_2O:D_2O, 9:1, 300 MHz) δ 8.52 (m, 3H, $C_\varepsilon H_{\ Hum}$ and 2 × $C_\varepsilon H_{\ His}$), 8.4–8.1 (m, 7H, 7 × CONH), 7.21–7.12 (m, 3H, $C_\delta H_{\ Hum}$ and 2 × $C_\delta H_{\ His}$), 4.60–4.59 (m, 3H, $C_\alpha H_{\ Hum}$ and 2× $C_\alpha H_{\ His}$), 4.22–3.97 (m, 4H, 4 × $C_\alpha H_{\ Ala}$), 3.71, 3.67 (2 × s, 3H, NCH_3), 3.24–3.29 (m, 6H, $C_\beta H_{2\ Hum}$ and 2× $C_\beta H_{2\ His}$), 1.94 (s, 3H, $CH_{3\ acetyl}$), 1.35–1.26 (m, 12H, $CH_{3\ Ala}$), no assigned (2H, 2 × $NH_{\ His}$). $^{13}C\{^1H\}$ NMR (H_2O:D_2O, 9:1, 75 MHz) δ 176.0, 175.4, 174.8, 174.4, 174.2, 171.4, 171.1, 171.0 (8 × C=O), 163.1, 162.6 (2x $C_\varepsilon H_{\ His}$), 136.6 ($C_\varepsilon H_{\ Hum}$), 136.6, 133.4 (2 × $C_{\gamma\ His}$), 130.6 ($C_{\gamma\ Hum}$), 128.5, 128.0 (2 × $C_\delta H_{\ His}$), 121.8 ($C_\delta H_{\ Hum}$),

35.5, 33.1 (2 × NCH_3), 26.0, 25.9, 25.8 (3 × $C_\beta H_2$), 21.4 ($CH_{3\ acetyl}$), 16.4, 16.4, 16.1, 16.0 (4 × $CH_{3\ Ala}$). HRMS (ESI^+): calcd for $[M – Cl]^+$ 784.3854, found 784.3830.

Synthesis of AYAȞAYA. Following the SPPS general procedure, using half the quantities stated for the Fmoc-Ala-Wang resin and the coupling cocktail. White powder (21 mg, 79±13%). ^1H NMR (H_2O:D_2O, 9:1, 300 MHz) δ 8.46-8.45 (m, 1H, $C_\varepsilon H_{\ Hum}$), 8.30–7.95 (m, 7H, 7 × CONH), 7.06–6.96, 6.73–6.67 (2 × m, 4H, $C_{Arom}H_{\ Tyr}$), 6.91 (s, $C_\delta H_{\ Hum}$, 1H), 4.49–4.38 (m, 3H, $C_\alpha H_{\ Hum}$ and 2 × $C_\alpha H_{\ Tyr}$), 4.24–3.98 (m, 4H, 4 × $C_\alpha H_{\ Ala}$), 3.64–3.62 (2 × s, 3H, NCH_3), 3.20–2.98 (6H, $C_\beta H_{2\ Hum}$ 2 × $C_\beta H_{2\ Tyr}$), 1.94 (s, 3H, $CH_{3\ acetyl}$), 1.35–1.26 (m, 12H, $CH_{3\ Ala}$), not resolved (2 × OH_{Tyr}). $^{13}C\{^1H\}$ NMR (H_2O:D_2O, 9:1, 75 MHz) δ not assigned (8 × C=O), 137.3 ($C_\varepsilon H_{\ Hum}$), 135–115 (12 × C_{Arom}, assigned by ^1H-^{13}C HSQC) 130.7 ($C_{\gamma\ Hum}$), 121.8 ($C_\delta H_{\ Hum}$), 54–49 (7 × C_α assigned by ^1H-^{13}C HSQC), 35.2, 33.4 (2 × NCH_3), ~35 (2 × $C_{\beta\ Tyr}$ assigned by ^1H-^{13}C HSQC), 25.0 ($C_\beta H_{2\ Hum}$), 21.8 ($CH_{3\ acetyl}$), 16.7, 16.5, 16.5, 16.4 (4 × $CH_{3\ Ala}$). HRMS (ESI^+): calcd for $[M – Cl]^+$ 836.3943, found 836.3916.

Synthesis of ACAȞACA. Following the SPPS general procedure, using half the quantities stated for the Fmoc-Ala-Wang resin and the coupling cocktail. White powder (12 mg, 59±11%). HRMS (ESI^+): calcd for $[M – Cl]^+$ 716.2860, found 716.2837.

Synthesis of AMAȞAMA. Following the SPPS general procedure, using half the quantities stated for the Fmoc-Ala-Wang resin and the coupling cocktail. White powder (9 mg, 36±9%). HRMS (ESI^+): calcd for $[M – Cl]^+$ 772.3486, found 772.3466.

Synthesis of AAAȞAAA. Following the SPPS general procedure, using half the quantities stated for the Fmoc-Ala-Wang resin and the coupling cocktail, **20-apo** was prepared as a white powder (16 mg, 71±10%). ^1H NMR (H_2O:D_2O, 9:1, 300 MHz) δ 8.52 (s, 1H, $C_\varepsilon H_{\ Hum}$), 8.3–8.0 (m, 7H, 7 × CONH), 7.23 (d, $^4J_{HH}$ = 1.3 Hz, $C_\delta H_{\ Hum}$, 1H), 4.66–4.61 (m, 1H, $C_\alpha H_{\ Hum}$), 4.35–4.15 (m, 6H, 6 × $C_\alpha H$), 3.80, 3,77 (2 × s, 3H, NCH_3), 3.21 (dd, $^2J_{HH}$ = 16.0 Hz, $^3J_{HH}$ = 8.9 Hz, 1H, $C_\beta H_{2\ Hum}$), 3.06 (dd, $^2J_{HH}$ = 16.0 Hz, $^3J_{HH}$ = 6.0 Hz, 1H, $C_\beta H_{2\ Hum}$), 1.98 (s, 3H, $CH_{3\ acetyl}$), 1.35–1.26 (m, 18H, $CH_{3\ Ala}$). $^{13}C\{^1H\}$ NMR (H_2O:D_2O, 9:1, 75 MHz) δ 176.2, 175.3, 174.9, 174.7, 174.5, 174.2, 170.9 (7 × C=O), 136.6 ($C_\varepsilon H_{\ Hum}$), 130.5 ($C_{\gamma\ Hum}$), 121.8 ($C_\delta H_{\ Hum}$), 51.2, 49.8, 49.6, 49.5, 49.4, 49.4, 48.5 (7 × $C_\alpha H$), 35.5, 33.1 (2 × NCH_3), 24.9 ($C_\beta H_{2\ Hum}$), 21.5 ($CH_{3\ acetyl}$), 16.5, 16.3, 16.3, 16.2, 16.2, 16.0 (6 × $CH_{3\ Ala}$). HRMS (ESI^+): calcd for $[M – Cl]^+$ 652.3418, found 652.3418.

Synthesis of AVAȞAVA. Following the SPPS general procedure, using half the quantities stated for the Fmoc-Ala-Wang resin and the coupling cocktail. White powder (21 mg, 85±11%). ^1H NMR (H_2O:D_2O, 9:1, 300 MHz) δ 8.59 (s, 1H, $C_\varepsilon H_{\ Hum}$), 8.4–8.0 (m, 7H, 7 × CONH), 7.23 (d, $^4J_{HH}$ = 1.4 Hz, $C_\delta H_{\ Hum}$, 1H), 4.67–4.59 (m, 1H, $C_\alpha H_{\ Hum}$), 4.33–4.17 (m, 4H, 4 × $C_\alpha H$), 4.06–4.04 (m, 2H, 2 × $C_\alpha H$), 3.83–3.77 (m, 6H, 2 × NCH_3), 3.19 (dd, $^2J_{HH}$ = 15.9 Hz, $^3J_{HH}$ = 6.1 Hz, 1H, $C_\beta H_{2\ Hum}$), 3.05 (dd, $^2J_{HH}$ =

16.0 Hz, ${}^{3}J_{HH}$ = 8.9 Hz, 1H, $C_\beta H_{2\ Hum}$), 2.07–1.99 (m, 2H, 2 × $C_\beta H_{Val}$), 1.97 (s, 3H, $CH_{3\ acetyl}$), 1.39 (d, ${}^{3}J_{HH}$ = 7.3 Hz, 3H, $CH_{3\ Ala}$), 1.34–1.29 (m, 9H, $CH_{3\ Ala}$), 0.95–0.87 (m, 12H, $CH_{3\ Val}$). ${}^{13}C\{{}^{1}H\}$ NMR (H_2O:D_2O, 9:1, 75 MHz) δ 176.2, 175.3, 174.7, 174.5, 174.0, 173.0, 173.0, 170.8 (8 × C=O), 136.5 ($C_\varepsilon H_{\ Hum}$), 130.5 ($C_{\gamma\ Hum}$), 121.8 ($C_\delta H_{\ Hum}$), 59.2, 59.0 (2 × $C_\alpha H_{Val}$), 51.1, 49.6, 49.6, 49.5, 48.6 (5 × $C_\alpha H$), 35.6, 33.1 (2 × NCH_3), 30.2, 30.1 (2 × $C_\beta H_{\ Val}$), 25.0 ($C_\beta H_{2\ Hum}$), 21.4 ($CH_{3\ acetyl}$), 18.2, 18.2, 17.6, 17.5, 16.5, 16.4, 16.3, 16.0 (8 × $CH_{3\ Ala+Val}$). HRMS (ESI^+): calcd for $[M - Cl]^+$ 708.4044, found 708.4028.

Synthesis of APAḦAPA. Following the SPPS general procedure, using half the quantities stated for the Fmoc-Ala-Wang resin and the coupling cocktail. White powder (19 mg, 67±9%). ${}^{1}H$ NMR (complete assignment not possible due to mixture) (H_2O:D_2O, 9:1, 300 MHz) δ 8.56 (s, 1H, $C_\varepsilon H_{\ Hum}$), 8.5–8.0 (m, 7H, 7 × CONH), 7.24 (s, $C_\delta H_{\ Hum}$, 1H), 4.61–4.50 (m), 4.42–4.17 (m), 3.83–3,75 (m), 3.68–3.56 (m), 3.21–3.04 (m), 2.30–2.21 (m), 2.05–1.82 (m), 1.44–1.14 (m, 18H, $CH_{3\ Ala}$). ${}^{13}C\{{}^{1}H\}$ NMR (assignment not possible due to mixture) (H_2O:D_2O, 9:1, 75 MHz) δ 201.1, 189.9, 174.3, 173.8, 173.7, 172.7, 157.5, 60.1, 47.6, 40.4, 29.2, 24.6, 21.3, 16.4, 15.9, 15.3, 15.2. HRMS (ESI^+): calcd for $[M - Cl]^+$ 704.3731, found 704.3735.

Synthesis of ALAḦALA. Following the SPPS general procedure, using half the quantities stated for the Fmoc-Ala-Wang resin and the coupling cocktail. White powder (27 mg, 87±14%). ${}^{1}H$ NMR (H_2O:D_2O, 9:1, 300 MHz) δ 8.59 (s, 1H, $C_\varepsilon H_{\ Hum}$), 8.3–8.0 (m, 7H, 7 × CONH), 7.23 (d, ${}^{4}J_{HH}$ = 1.2 Hz, $C_\delta H_{\ Hum}$, 1H), 4.66–4.61 (m, 1H, $C_\alpha H_{\ Hum}$), 4.36–4.14 (m, 6H, 6 × $C_\alpha H$), 3.81, 3.77 (2 × s, 3H, NCH_3), 3.20 (dd, ${}^{2}J_{HH}$ = 15.9 Hz, ${}^{3}J_{HH}$ = 5.9 Hz, 1H, $C_\beta H_{2\ Hum}$), 3.05 (dd, ${}^{2}J_{HH}$ = 16.0 Hz, ${}^{3}J_{HH}$ = 9.0 Hz, 1H, $C_\beta H_{2\ Hum}$), 1.97 (s, 3H, $CH_{3\ acetyl}$), 1.69–1.51 (m, 6H, 2 × ($C_\beta H_{2\ Leu}$ + $C_\gamma H_{Leu}$)), 1.40–1.29 (m, 12H, $CH_{3\ Ala}$), 0.92–0.84 (m, 12H, $CH_{3\ Leu}$). ${}^{13}C\{{}^{1}H\}$ NMR (H_2O:D_2O, 9:1, 75 MHz) δ 176.1, 175.5, 174.8, 174.5, 174.4, 174.2, 174.2, 170.9 (8 × C=O), 136.6 ($C_\varepsilon H_{\ Hum}$), 130.5 ($C_{\gamma\ Hum}$), 121.7 ($C_\delta H_{\ Hum}$), 52.1, 52.0, 51.2, 49.9, 49.7, 49.6, 48.5 (7 × $C_\alpha H$), 39.6, 39.5 (2 × $C_\beta H_{2\ Leu}$), 35.6, 33.1 (2 × NCH_3), 24.9 ($C_\beta H_{2\ Hum}$), 24.2, 24.2 (2 × $C_\gamma H_{Leu}$), 22.1, 22.0, 21.5, 20.8, 20.7, 20.6 ($CH_{3\ acetyl}$ + $C_\beta H_{2\ Leu}$ + $C_\gamma H_{Leu}$ + $CH_{3\ Leu}$), 16.5, 16.3, 16.0 (3 × $CH_{3\ Ala}$). HRMS (ESI^+): calcd for $[M - Cl]^+$ 736.4357, found 736.4346.

Synthesis of AIAḦAIA. Following the SPPS general procedure, using half the quantities stated for the Fmoc-Ala-Wang resin and the coupling cocktail. White powder (32 mg, 92±8%). ${}^{1}H$ NMR (H_2O:D_2O, 9:1, 300 MHz) δ 8.59 (s, 1H, $C_\varepsilon H_{\ Hum}$), 8.3–8.0 (m, 7H, 7 × CONH), 7.23 (d, ${}^{4}J_{HH}$ = 1.4 Hz, $C_\delta H_{\ Hum}$, 1H), 4.65 (dd, ${}^{3}J_{HH}$ = 6.1 Hz, ${}^{3}J_{HH}$ = 8.9 Hz, 1H, $C_\alpha H_{\ Hum}$), 4.35–4.20 (m, 4H, 4 × $C_\alpha H_{Ala}$), 4.12–4.08 (m, 2H, 2 × $C_\alpha H_{Ile}$), 3.81, 3.77 (2 × s, 3H, NCH_3), 3.19 (dd, ${}^{2}J_{HH}$ = 15.9 Hz, ${}^{3}J_{HH}$ = 5.9 Hz, 1H, $C_\beta H_{2\ Hum}$), 3.04 (dd, ${}^{2}J_{HH}$ = 15.9 Hz, ${}^{3}J_{HH}$ = 8.9 Hz, 1H, $C_\beta H_{2\ Hum}$), 1.97 (s, 3H, $CH_{3\ acetyl}$), 1.86–1.75 (m, 2H, 2 × $C_\beta H_{Ile}$), 1.52–1.11 (m, 14H, 4 × $CH_{3\ Ala}$ + 2 × $C_\gamma H_{2\ Ile}$), 0.91 (d, ${}^{3}J_{HH}$ = 6.8 Hz, 3H, $CH_{3\ Ile}$), 0.86–0.80 (m, 9H, $CH_{3\ Ile}$). ${}^{13}C\{{}^{1}H\}$ NMR (H_2O:D_2O, 9:1, 75 MHz) δ 176.1, 175.3, 174.6, 174.5, 174.0,

173.1, 173.1, 170.8 (8 × C=O), 136.5 ($C_\varepsilon H_{Hum}$), 130.5 ($C_{\gamma\,Hum}$), 121.8 ($C_\delta H_{Hum}$), 58.1, 57.9 (2 × $C_\alpha H_{Ile}$), 51.1, 49.6, 49.6, 48.6 (4 × $C_\alpha H$), 36.1, 36.0 (2 × $C_\beta H_{Ile}$), 35.6, 33.1 (2 × NCH_3), 25.0 ($C_\beta H_{2\,Hum}$), 24.4, 24.3 (2 × $C_\gamma H_{2\,Ile}$), 21.4 ($CH_{3\,acetyl}$), 16.5, 16.4, 16.3, 16.0, 14.6, 14.5 (6 × $CH_{3\,Ala+Ile}$), 10.0, 9.9 (2 × $C_\delta H_{3\,Ile}$). HRMS (ESI^+): calcd for $[M - Cl]^+$ 736.4357, found 736.4335.

Synthesis of AFAȞAFA. Following the SPPS general procedure, using half the quantities stated for the Fmoc-Ala-Wang resin and the coupling cocktail. White powder (29 mg, 89±11%). ^{1}H NMR (H_2O:D_2O, 9:1, 300 MHz) δ 8.46-8.45 (m, 1H, $C_\varepsilon H_{Hum}$), 8.3–7.98 (m, 7H, 7 × CONH), 7.27–7.10 (m, 10H, $C_{Arm}H_{Phe}$), 6.97 (s, $C_\delta H_{Hum}$, 1H), 4.53–4.44 (m, 3H, $C_\alpha H_{Hum}$ and 2 × $C_\alpha H_{Phe}$), 4.24–3.98 (m, 4H, 4 × $C_\alpha H_{Ala}$), 3.64–3.62 (2 × s, 3H, NCH_3), 3.20–2.98 (6H, $C_\beta H_{2\,Hum}$ 2 × $C_\beta H_{2\,Phe}$), 1.84 (s, 3H, $CH_{3\,acetyl}$), 1.28–1.05 (m, 12H, $CH_{3\,Ala}$). ^{13}C{^{1}H} NMR (H_2O:D_2O, 9:1, 75 MHz) δ 176.0, 175.1, 174.6, 174.2, 174.1, 172.7, 172.4, 170.8 (8 × C=O), 136.8 ($C_\varepsilon H_{Hum}$), 130–128 (12 × C_{Arom}, assigned by ^{1}H-^{13}C HSQC), 127.1 ($C_{\gamma\,Hum}$), 121.8 ($C_\delta H_{Hum}$), 54–49 (7 × C_α assigned by ^{1}H-^{13}C HSQC), 36.4, 36.9 (2 × $C_{\beta\,Phe}$), 35.1, 33.0 (2 × NCH_3), 25.0 ($C_\beta H_{2\,Hum}$), 21.8 ($CH_{3\,acetyl}$), 16.5, 16.3, 16.2, 16.0 (4 × $CH_{3\,Ala}$). HRMS (ESI^+): calcd for $[M - Cl]^+$ 804.4044, found 804.4063.

Synthesis of AWAȞAWA. Following the SPPS general procedure, using half the quantities stated for the Fmoc-Ala-Wang resin and the coupling cocktail. White powder (9 mg, 29±7%). ^{1}H NMR (complete assignment not possible due to mixture) (H_2O:D_2O, 9:1, 300 MHz) δ 8.31 ($C_\varepsilon H_{Hum}$), 8.29 ($C_\varepsilon H_{Hum}$), 8.3–7.98 (m, 7H, 7 × CONH), 7.68–7.66, 7.59–7.56, 7.48–7.45, 7.39–7.37, 7.24–7.12, 6.74, 6.72 ($C_\delta H_{Hum}$ + CH_{Trp}), 4.62–4.56 (m), 4.42–4.37 (m), 4.18–3.97 (m), 3.68–3.57 (m), 3.32–3.18 (m), 2.77–2.71 (m), 1.83 (s, 3H, $CH_{3\,acetyl}$), 1.31–1.09 (m, $CH_{3\,Ala}$). ^{13}C{^{1}H} NMR (H_2O:D_2O, 9:1, 75 MHz) not resolved. HRMS (ESI^+): calcd for $[M - Cl]^+$ 882.4262, found 882.4248.

Synthesis of ATAȞATA. Following the SPPS general procedure, using half the quantities stated for the Fmoc-Ala-Wang resin and the coupling cocktail. White powder (23 mg, 81±10%). ^{1}H NMR (H_2O:D_2O, 9:1, 300 MHz) δ 8.50 (m, 1H, $C_\varepsilon H_{Hum}$), 8.4–8.0 (m, 7H, 7 × CONH), 7.14 (s, $C_\delta H_{Hum}$, 1H), 4.60–4.55 (m, 1H, $C_\alpha H_{Hum}$) 4.29–4.03 (m, 6H, 6 × $C_\alpha H$ and 2H, 2 × $C_\beta H_{Thr}$), 3.73, 3.69 (2 × s, 3H, NCH_3), 3.04 (dd, $^2J_{HH}$ = 16.0 Hz, $^3J_{HH}$ = 9.0 Hz, 1H, $C_\beta H_{2\,Hum}$), 3.01 (dd, $^2J_{HH}$ = 15.9 Hz, $^3J_{HH}$ = 6.1 Hz, 1H, $C_\beta H_{2\,Hum}$), 1.90 (s, 3H, $CH_{3\,acetyl}$), 1.34–1.08 (m, 12H, $CH_{3\,Ala}$ and 3H, $C_\gamma H_{3\,Thr}$), not resolved (2 × OH_{Thr}). ^{13}C{^{1}H} NMR (H_2O:D_2O, 9:1, 75 MHz) δ 176.2, 175.8, 174.8, 174.7, 174.3, 171.5, 171.3, 171.1 (8 × C=O), 136.6 ($C_\varepsilon H_{Hum}$), 130.5 ($C_{\gamma\,Hum}$), 121.8 ($C_\delta H_{Hum}$), 67.0, 67.1 (2 × $C_\gamma H_{3\,Thr}$), 59.0, 58.7 (2 × CHOH), 55.6, 55.6, 51.4, 50.2, 50.0, 50.0, 48.9 (7 × $C_\alpha H$), 35.5, 33.1 (2 × NCH_3), 25.0 ($C_\beta H_{2\,Hum}$), 21.5 ($CH_{3\,acetyl}$), 18.8, 18.7, 16.3, 16.1 (4 × $CH_{3\,Ala}$). (ESI^+): calcd for $[M - Cl]^+$ 712.3630, found 712.3614.

Synthesis of ANAȞANA. Following the SPPS general procedure, using half the quantities stated for the Fmoc-Ala-Wang resin and the coupling cocktail. White powder (25 mg, 79±9%). ^{1}H NMR

(H_2O:D_2O, 9:1, 300 MHz) δ 8.43 (m, 1H, $C_εH$ $_{Hum}$), 8.4–8.0 (m, 7H, 7 × CONH), 7.07 (m, 1H, $C_δH$ $_{Hum}$), 4.50–4.46 (m, 3H, $C_αH$ $_{Hum}$ + 2 × $C_αH$ $_{Asn}$), 4.21–3.98 (m, 4H, 4 × $C_αH$ $_{Ala}$), 3.65, 3.62 (2 × s, 3H, NCH_3), 3.08 (dd, $^2J_{HH}$ = 15.9 Hz, $^3J_{HH}$ = 9.4 Hz, 1H, $C_βH_2$ $_{Hum}$), 2.93 (dd, $^2J_{HH}$ = 15.9 Hz, $^3J_{HH}$ = 5.7 Hz, 1H, $C_βH_2$ $_{Hum}$), 2.68–2.50 (m, 4H, 2 × $C_βH_2$ $_{Asn}$), 1.84 (s, 3H, CH_3 $_{acetyl}$), 1.25–1.14 (m, 12H, 4 × CH_3 $_{Ala}$), not resolved (NH_2 $_{Asn}$). $^{13}C\{^1H\}$ NMR (H_2O:D_2O, 9:1, 75 MHz) δ 175.9, 175.4, 175.2, 174.8, 174.4, 174.4, 174.2, 172.2, 171.8, 171.1 (8 × C=O + 2 × CON $_{Asn}$), 136.5 ($C_εH$ $_{Hum}$), 130.6 ($C_γ$ $_{Hum}$), 121.8 ($C_δH$ $_{Hum}$), 51.1, 50.2, 50.1, 49.9, 49.8, 49.8, 48.6 (7 × $C_αH$), 36.0 (NCH_3), 35.7, 35.4 (2 × $C_βH_2$ $_{Asp}$), 33.1 (NCH_3), 24.6 ($C_βH_2$ $_{Hum}$), 21.4 (CH_3 $_{acetyl}$), 16.3, 16.2, 16.1, 16.0 (4 × CH_3 $_{Ala}$). HRMS (ESI^+): calcd for $[M – Cl]^+$ 738.3535, found 738.3513.

Synthesis of AQAḦAQA. Following the SPPS general procedure, using half the quantities stated for the Fmoc-Ala-Wang resin and the coupling cocktail. White powder (21 mg, 70±9%). ^1H NMR (H_2O:D_2O, 9:1, 300 MHz) δ 8.47 (m, 1H, $C_εH$ $_{Hum}$), 8.4–8.0 (m, 7H, 7 × CONH), 7.13 (s, 1H, $C_δH$ $_{Hum}$), 4.55 (dd, $^3J_{HH}$ = 9.1/5.0 Hz, 1H, $C_αH$ $_{Hum}$), 4.25–4.05 (m, 6H, 6 × $C_αH$), 3.70, 3.67 (2 × s, 3H, NCH_3), 3.14–2.69 (m, 2H, $C_βH_2$ $_{Hum}$), 2.30–2.21 (m, 4H, 2 × $C_γH_2$ $_{Gln}$), 2.15–2.04 (m, 4H, 2 × $C_βH_2$ $_{Gln}$), 1.88 (s, 3H, CH_3 $_{acetyl}$), 2.05–1.75 (m, 2H, 2 × $C_βH_2$ $_{Asp}$), 1.31–1.19 (m, 12H, 3 × CH_3 $_{Ala}$). $^{13}C\{^1H\}$ NMR (H_2O:D_2O, 9:1, 75 MHz) δ 177.8, 177.8, 176.1, 175.6, 174.8, 174.5, 174.3, 173.0, 172.8, 171.0 (8 × C=O + 2 × CON $_{Gln}$), 136.6 ($C_εH$ $_{Hum}$), 130.5 ($C_γ$ $_{Hum}$), 121.8 ($C_δH$ $_{Hum}$), 52.8, 52.7, 51.1, 49.9, 49.7, 49.7, 48.6 (7 × $C_αH$), 35.5, 33.1 (2 × NCH_3), 30.9, 30.8 (2 × $C_γH_2$ $_{Glu}$), 26.8, 26.4 (2 × $C_βH_2$ $_{Asp}$), 24.9 ($C_βH_2$ $_{Hum}$), 21.5 (CH_3 $_{acetyl}$), 16.4, 16.3, 16.3, 15.9 (4 × CH_3 $_{Ala}$). HRMS (ESI^+): calcd for $[M – Cl]^+$ 766.3834, found 766.3862.

Synthesis of ARAḦARA. Following the SPPS general procedure, using half the quantities stated for the Fmoc-Ala-Wang resin and the coupling cocktail. White powder (25 mg, 74±8%). ^1H NMR (H_2O:D_2O, 9:1, 300 MHz) δ 8.48 (s, 1H, $C_εH$ $_{Hum}$), not resolved (m, 12H, 7 × CONH + CN_3H_5 $_{Arg}$), 7.16 (s, 1H, $C_δH$ $_{Hum}$), 4.59–4.51 (m, 1H, $C_αH$ $_{Hum}$), 4.42–4.06 (m, 6H, 6 × $C_αH$), 3.71, 3.68 (2 × s, 3H, NCH_3), 3.14–2.98 (m 6H, $C_βH_2$ $_{Hum}$ + 2 × $C_γH_2$ $_{Arg}$), 1.89 (s, 3H, CH_3 $_{acetyl}$), 1.81–1.47 (m, 8H, 2 × $C_βH_2$ $_{Arg}$ + 2 × $C_δH_2$ $_{Arg}$), 1.30–1.19 (m, 12H, 4 × CH_3 $_{Ala}$). $^{13}C\{^1H\}$ NMR (H_2O:D_2O, 9:1, 75 MHz) δ 176.2, 175.6, 174.9, 174.5, 174.2, 173.3, 173.1, 171.0 (8 × C=O), 136.6 ($C_εH$ $_{Hum}$), 130.6 ($C_γ$ $_{Hum}$), 121.7 ($C_δH$ $_{Hum}$), 53.1, 52.9, 51.2, 49.9, 49.7, 49.6, 48.5 (7 × $C_αH$), 40.5, 40.4 (2 × $C_δH_2$ $_{Arg}$), 35.5, 33.1 (2 × NCH_3), 28.1, 27.8 (2 × $C_βH_2$ $_{Arg}$), 24.8 ($C_βH_2$ $_{Hum}$), 24.2, 24.3 (2 × $C_γH_2$ $_{Lys}$), 21.5 (CH_3 $_{acetyl}$), 16.5, 16.3, 16.3, 16.0 (4 × CH_3 $_{Ala}$). HRMS (ESI^+): calcd for $[M – Cl]^{2+}$ 411.7383, found 411.7374.

1.3. General procedure for the preparation of AXAḦAXA–Ir

The resin-bound peptide **resin-AXAḦAXA** was shaken for 24 h with Ag_2O (18.5 mg, 0.080 mmol) and Me_4NCl (8.8 mg, 0.080 mmol) in CH_2Cl_2:MeCN (7 mL, 1:1, v/v). Then $[IrCl_2Cp^*]_2$ (31.9 mg, 0.040

mmol) was added and the mixture was shaken for an additional 24 h. After filtration, the resin was washed with NMP (2 × 5 mL), MeOH (2 × 5 mL) and CH_2Cl_2 (2 × 5 mL). Subsequent cleavage of the peptide from the resin and purification by prep-HPLC was performed as described above for the preparation of **AXAḦAXA** and afforded **AXAḦAXA–Ir** as light orange powder (apart from X = C, M). Peptide integrity was assessed by HR-MS and purity by NMR spectroscopy and LC-MS (pp. S25).

Synthesis of AGAḦAGA–Ir. Light orange powder (8.0 mg, 15±5%). ^{1}H NMR (H_2O:D_2O, 9:1, 300 MHz) δ not resolved (m, 7H, 7 × CONH), 7.16–7.10 (m, 1H, $C_{\delta}H_{Hum}$), 4.72–4.64 (m, 1H, $C_{\alpha}H_{Hum}$), 4.38–4.20 (m, 4H, 4 × $C_{\alpha}H$), 3.91, 3.88 (2 × s, 2H, $CH_{2\,Gly}$), 3.67–3.64 (m, 6H, 2 × NCH_3), 3.30–3.22, 3.06–2.97 (2 × m, 1H, $CH_{2\,Hum}$), 1.99 (s, 3H, $CH_{3\,acetyl}$), 1.63–1.60 (m, 15H, Cp*), 1.40–1.29 (m, 12H, 4 × $CH_{3\,Ala}$). $^{13}C\{^{1}H\}$ NMR (H_2O:D_2O, 9:1, 75 MHz) δ 176.1, 175.1, 174.9, 174.4, 174.4, 174.3, 171.5, 171.3, 170.8 (9 × C=O), 131.2 ($C_{\gamma\,Hum}$), 123.4 ($C_{\delta}H_{Hu}$), 91.5 (C_{Cp}), 51.2, 51.2, 50.1, 50.0, 49.9, 49.9, 49.8, 48.5 (8 × $C_{\alpha}H_{Ala}$), 42.3, 42.1 (2 × $CH_{2\,Gly}$), ~37 (broad s, NCH_3, assigned by ^{1}H-^{13}C HSQC), 25.8 ($CH_{2\,Hum}$), 21.6 ($CH_{3\,acetyl}$), 16.4, 16.4, 16.3, 16.1 (4 × $CH_{3\,Ala}$), 8.3, 8.1 (2 × Cp–CH_3), not resolved (C–Ir). HRMS (ESI^+): calcd for $[M - Cl - HCl]^+$ 950.3752, found 950.3720

Synthesis of ASAḦASA–Ir. Light orange powder (35 mg, 25±8%). ^{1}H NMR (H_2O:D_2O, 9:1, 300 MHz) δ not resolved (m, 7H, 7 × CONH), 7.13–7.06 (m, 1H, $C_{\delta}H_{Hum}$), 4.62–4.57 (m, 1H, $C_{\alpha}H_{Hum}$), 4.39–4.16 (m, 6H, 6 × $C_{\alpha}H$), 3.88–3.81 (m, 4H, 2 × $C_{\beta}H_{2\,Ser}$), 3.67–3.59 (m, 6H, 2 × NCH_3), 3.25–3.16, 3.06–2.93 (2 × m, 1H, $CH_{2\,Hum}$), 1.96 (s, 3H, $CH_{3\,acetyl}$), 1.60–1.57 (m, 15H, Cp*), 1.38–1.27 (m, 12H, 4 × $CH_{3\,Ala}$), not resolved (2 × OH_{Ser}). $^{13}C\{^{1}H\}$ NMR (H_2O:D_2O, 9:1, 75 MHz) δ 176.0, 175.7, 175.7, 174.8, 174.7, 174.7, 174.6, 174.4, 171.7, 171.7, 171.5, 171.5, 171.1 (13 × C=O), 131.0 ($C_{\gamma\,Hum}$), 123.3 ($C_{\delta}H_{Hu}$), 91.5 (C_{Cp}), 60.9, 60.8, 60.3 (3 × $C_{\beta}H_2$), 55.4 ($C_{\alpha}H_{Ser}$), 51.3 ($C_{\alpha}H_{Hum}$), 50.1, 49.9, 49.8, 48.6 (4 × $C_{\alpha}H_{Ala}$), 36.7, 34.2 (2 × broad s, NCH_3), 25.9 ($CH_{2\,Hum}$), 21.5 ($CH_{3\,acetyl}$), 16.5, 16.4, 16.3, 16.1 (4 × $CH_{3\,Ala}$), 8.2, 8.1 (2 × Cp–CH_3), not resolved (C–Ir). HRMS (ESI^+): calcd for $[M - 2Cl]^{2+}$ 505.7016, found 505.7010.

Synthesis of ADAḦADA–Ir. Light orange powder (19 mg, 32±11%). ^{1}H NMR (H_2O:D_2O, 9:1, 300 MHz) δ not resolved (m, 7H, 7 × CONH), 7.15–7.04 (m, 1H, $C_{\delta}H_{Hum}$), 4.71–4.58 (m, 3H, $C_{\alpha}H_{Hum+Asp}$), 4.33–4.15 (m, 4H, 4 × $C_{\alpha}H_{Ala}$), 3.65–3.59 (m, 6H, 2 × NCH_3), 3.26–3.20, 3.10–3.01 (2 × m, 1H, $CH_{2\,Hum}$), 2.96–2.88, 2.83–2.73 (2 × m, 1H, $C_{\beta}H_{2\,Asp}$), 1.98 (s, 3H, $CH_{3\,acetyl}$), 1.62–1.58 (m, 15H, Cp*), 1.39–1.30 (m, 12H, 4 × $CH_{3\,Ala}$). $^{13}C\{^{1}H\}$ NMR (H_2O:D_2O, 9:1, 75 MHz) δ 175.9, 175.5, 175.5, 174.4, 174.1, 173.8, 172.1, 171.7 (8 × C=O), 123.5 ($C_{\delta}H_{Hum}$), 91.5, 88.4 (2 × C_{Cp}), 50.3, 49.9, 49.8, 48.6 (4 × $C_{\alpha}H$), 36.6 (NCH_3), 35.2, 35.0 ($C_{\beta}H_{2\,Asp}$), 34.4 (NCH_3), 26.1, 25.8 (2 × $CH_{2\,Hum}$), 21.5, 21.5 (2 × $CH_{3\,acetyl}$), 16.4, 16.3, 16.1, 16.0 (4 × $CH_{3\,Ala}$), 8.6, 8.2 (2 × Cp–CH_3), not resolved (C–Ir). HRMS (ESI^+): calcd for $[M - 2Cl]^{2+}$ 555.7284, found 555.7286.

Synthesis of AEAḦAEA–Ir. Light orange powder (16 mg, 32±11%). ^{1}H NMR (H_2O:D_2O, 9:1, 300 MHz) δ not resolved (m, 7H, 7 × CONH), 7.15–7.10 (m, 1H, $C_\delta H_{Hum}$), 4.66–4.59 (m, 1H, $C_\alpha H_{Hum}$), 4.36–4.29 (m, 6H, 6 × $C_\alpha H_{Ala+Glu}$), 3.66–3.60 (m, 6H, 2 × NCH_3), 3.26–3.18, 3.10–2.98 (2 × m, 1H, $CH_{2\ Hum}$), 2.52–2.43 (m, 4H, $C_\gamma H_{2\ Glu}$), 2.18–2.02 (m, 2H, $C_\beta H_{2\ Glu}$), 1.98 (s, 3H, $CH_{3\ acetyl}$), 1.98–1.87 (m, 1H, $C_\beta H_{2\ Glu}$), 1.63–1.60 (m, 15H, Cp*), 1.40–1.31 (m, 12H, 4 × $CH_{3\ Ala}$). ^{13}C{^{1}H} NMR (H_2O:D_2O, 9:1, 75 MHz) δ 176.9, 176.9, 176.1, 175.8, 175.7, 174.8, 174.7, 174.6, 174.5, 174.4, 174.2, 173.1, 173.1, 172.8 (12 × C=O), 123.0 ($C_\delta H_{Hum}$), 91.5, 88.5 (2 × C_{Cp}), 52.8, 52.6, 50.1, 49.9, 49.7, 49.6, 48.6 (7 × C_αH), 36.8, 34.3 (2 × NCH_3), 29.8, 29.7 ($C_\gamma H_{2\ Glu}$), 26.1, 25.9, 25.7, 25.7 (2 × $CH_{2\ Hum}$ + 2 × $C_\beta H_{2\ Glu}$), 21.5 ($CH_{3\ acetyl}$), 16.4, 16.3, 16.0 (3 × $CH_{3\ Ala}$), 8.6, 8.3 (2 × Cp–CH_3), not resolved (C–Ir). HRMS (ESI$^+$): calcd for $[M-2Cl]^{2+}$ 533.6965, found 533.6961.

Synthesis of AKAḦAKA–Ir. Light orange powder (16 mg, 26±9%). ^{1}H NMR (H_2O:D_2O, 9:1, 300 MHz) δ not resolved (m, 7H, 7 × CONH), 7.13–7.09 (m, 1H, $C_\delta H_{Hum}$), 4.62–4.57 (m, 1H, $C_\alpha H_{Hum}$), 4.34–4.17 (m, 6H, 6 × $C_\alpha H_{Ala+Lys}$), 3.67–3.62 (m, 6H, 2 × NCH_3), 3.26–3.18 (m, 1H, $CH_{2\ Hum}$), 3.08–2.94 (m, 5H, $CH_{2\ Hum}$ + $C_\varepsilon H_{2\ Lys}$), 1.98 (s, 3H, $CH_{3\ acetyl}$), 1.98–1.87 (m, 1H, $C_\beta H_{2\ Glu}$), 1.64–1.31 (m, 39H, Cp* + 2 × $C_\beta H_2$ + 2 × $C_\gamma H_2$ + 2 × $C_\delta H_2$). ^{13}C{^{1}H} NMR (H_2O:D_2O, 9:1, 75 MHz) δ 176.3, 176.2, 174.8, 174.3, 173.9, 173.7, 173.6 (7 × C=O), 123.0 ($C_\delta H_{Hum}$), 88.5 (C_{Cp}), 53.4, 53.3, 53.1, 50.1, 49.7, 49.5, 48.6 (7 × C_αH), 39.1 ($C_\varepsilon H_2$), 36.8, 34.7 (2 × NCH_3), 30.4, 30.0, 26.3, 26.1, 22.1, 21.9 (6 × $CH_{2\ Lys}$), 21.5 ($CH_{3\ acetyl}$), 16.6, 16.5, 16.4, 16.3, 16.0 (5 × $CH_{3\ Ala}$), 8.5, 8.3 (2 × Cp–CH_3), not resolved (C–Ir). HRMS (ESI$^+$): calcd for $[M-2Cl+H]^{3+}$ 364.8459, found 364.8465.

Synthesis of AHAḦAHA–Ir. Light orange powder (13 mg, 20±7%). ^{1}H NMR (H_2O:D_2O, 9:1, 300 MHz) δ 8.5, 8.2 (2 ×, s, 1H, $C_\varepsilon H_{His}$), 8.4–8.1 (m, 7H, 7 × CONH), 7.2–7.0 (m, 3H, $C_\delta H_{Hum}$ and 2 × $C_\delta H_{His}$), 4.65–4.55 (m, 3H, $C_\alpha H_{Hum}$ and 2× $C_\alpha H_{His}$), 4.3–4.1 (m, 4H, 4 × $C_\alpha H_{Ala}$), 3.7, 3.6 (2 × broad s, 3H, NCH_3), 3.2–3.0 (m, 6H, $C_\beta H_{2\ Hum}$ and 2× $C_\beta H_{2\ His}$), 1.90 (s, 3H, $CH_{3\ acetyl}$), 1.63–1.56 (m, 15H, Cp*), 1.35–1.26 (m, 12H, $CH_{3\ Ala}$), no assigned (2H, 2 × NH_{His}). ^{13}C{^{1}H} NMR (H_2O:D_2O, 9:1, 75 MHz) δ 176.0, 174.7, 174.6, 174.0, 172.4, 171.3, 171.0, 169.3 (8 × C=O), 163.1, 162.6 (2x $C_\varepsilon H_{His}$), 136.6 ($C_\varepsilon H_{Hum}$), 137.1, 133.4 (2 × $C_{\gamma\ His}$), not resolved ($C_{\gamma\ Hum}$), 128.0 (2 × $C_\delta H_{His}$), 121.8 ($C_\delta H_{Hum}$), 89.7 (Cp*), 52.3, 51.8, 50.6, 49.8, 49.7, 48.6, 48.6 (7 × C_αH), 36.2 (2 × NCH_3), 26.3, (3 × $C_\beta H_2$), 21.4 ($CH_{3\ acetyl}$), 17.7, 16.4, 16.0, 16.0 (4 × $CH_{3\ Ala}$), 8.41 (Cp–CH_3), not resolved (C–Ir). HRMS (ESI$^+$): calcd for $[M-2Cl]^{2+}$ 547.7121, found 547.7122.

Synthesis of AYAḦAYA–Ir. Light orange powder (32 mg, 27±9%). ^{1}H NMR (H_2O:D_2O, 9:1, 300 MHz) δ 8.26–7.81 (m, 7H, 7 × CONH), 7.04–6.90, 6.73–6.57 (2 × m, 4H and 5H, $C_{Arm}H_{Tyr}$ and $C_\delta H_{Hum}$), 4.49–4.35 (m, 3H, $C_\alpha H_{Hum}$ and 2 × $C_\alpha H_{Tyr}$), 4.26–3.99 (m, 4H, 4 × $C_\alpha H_{Ala}$), 3.56–3.51 (2 × d, 3H, J= 8.0 and 12.1 Hz, NCH_3), 3.07–2.71 (6H, $C_\beta H_{2\ Hum}$ 2 × $C_\beta H_{2\ Tyr}$), 1.86 (s, 3H, $CH_{3\ acetyl}$), 1.64–1.31 (d, 15H, J=11.8 Hz, Cp*), 1.31–1.09 (m, 12H, $CH_{3\ Ala}$), not resolved (2 × OH_{Tyr}). ^{13}C{^{1}H} NMR (H_2O:D_2O, 9:1, 75 MHz) δ 175.1, 174.1, 174.4 172.2, 172.2, 171.1, 171.0, 170.1 (8 × C=O), 130–114 (12 × C_{Arom},

assigned by ^{1}H-^{13}C HSQC), 130.7–130.5 ($C_{\gamma\ Hum}$), 119 ($C_{\delta}H_{\ Hum}$), ~91 (C_{Cp}), 54–48 (7 × C_{α} assigned by ^{1}H-^{13}C HSQC), 37.5 (2 × NCH_3), ~37 (2 × $C_{\beta\ Tyr}$ assigned by ^{1}H-^{13}C HSQC), 25.0 ($C_{\beta}H_{2\ Hum}$), 21.8 ($CH_{3\ acetyl}$), 16.7, 16.5, 16.5, 16.4 (4 × $CH_{3\ Ala}$), 8.1, 8.0 (2 × Cp–CH_3), not resolved (C–Ir). HRMS (ESI^{+}): calcd for $[M-2Cl]^{2+}$ 581.7329, found 581.7380.

Synthesis of AAAḦAAA–Ir. Light orange powder (30 mg, 31±9%). ^{1}H NMR (H_2O:D_2O, 9:1, 300 MHz) δ not resolved (m, 7H, 7 × CONH), 7.13–7.10 (m, 1H, $C_{\delta}H_{\ Hum}$), 4.62–4.55 (m, 1H, $C_{\alpha}H_{\ Hum}$), 4.32–4.15 (m, 6H, 6 × $C_{\alpha}H$), 3.74–3.57 (m, 6H, 2 × NCH_3), 3.26–3.20, 3.06–3.03 (2 × m, 1H, $CH_{2\ Hum}$), 1.99 (s, 3H, $CH_{3\ acetyl}$), 1.64–1.57 (m, 15H, Cp*), 1.40–1.32 (m, 18H, 6 × $CH_{3\ Ala}$). ^{13}C{^{1}H} NMR (H_2O:D_2O, 9:1, 75 MHz) δ 175.5, 175.5, 174.9, 174.8, 171.5, 162.7 (6 × C=O), 131.0 ($C_{\gamma\ Hum}$), 122.3 ($C_{\delta}H_{\ Hum}$ (assigned by ^{1}H-^{13}C HSQC)), 88.5 (C_{Cp}), 50.0, 49.8, 49.6, 49.5, 49.5, 49.4, 48.6 (5 × $C_{\alpha}H$), 39.7, 39.4 (2 × $C_{\beta}H_{2\ Leu}$), 36.7, 34.3 (2 × NCH_3), 25.9 ($CH_{2\ Hum}$), 21.5 ($CH_{3\ acetyl}$), 16.6, 16.4, 16.3, 16.3, 16.1, 16.0 (6 × $CH_{3\ Ala}$ (strongly overlapping peaks)), 8.5, 8.5 (2 × Cp–CH_3), not resolved (C–Ir). HRMS (ESI^{+}): calcd for $[M-2Cl]^{2+}$ 489.7076, found 489.7072

Synthesis of AVAḦAVA–Ir. Light orange powder (4 mg, 4±1%). ^{1}H NMR (H_2O:D_2O, 9:1, 300 MHz) δ not resolved (m, 7H, 7 × CONH), 7.23–7.03 (m, 1H, $C_{\delta}H_{\ Hum}$), 4.23–4.14 (m, 4H, 4 × $C_{\alpha}H_{\ Hum}$), 4.02–3.98 (m, 2H, 2 × $C_{\alpha}H$), 3.64–3.40 (m, 6H, 2 × NCH_3), 3.29–2.83 (m, 2H, $CH_{2\ Hum}$), 2.03–1.89 (m, 2H, 2 × $C_{\beta}H_{\ Val}$), 1.89 (s, 3H, $CH_{3\ acetyl}$), 1.53–1.50 (m, 15H, Cp*), 1.39–1.22 (m, 12H, 4 × $CH_{3\ Ala}$), 0.92–0.79 (m, 12H, 4 × $CH_{3\ Val}$). ^{13}C{^{1}H} NMR (H_2O:D_2O, 9:1, 75 MHz) δ not resolved. HRMS (ESI^{+}): calcd for $[M-2Cl]^{2+}$ 517.7377, found 517.98

Synthesis of APAḦAPA–Ir. Light orange powder (28 mg, 36±10%). ^{1}H NMR (H_2O:D_2O, 9:1, 300 MHz) δ not resolved (m, 7H, 7 × CONH), 7.17–7.13 (m, 1H, $C_{\delta}H_{\ Hum}$), 4.63–4.47 (m, 3H, $C_{\alpha}H$), 4.40–4.26 (m, 3H, 3 × $C_{\alpha}H$), 4.20–4.11 (m, 1H, $C_{\alpha}H$), 3.83–3.73 (m, 2H, 2 × $NCH_{2\ Pro}$), 3.67–3.54 (m, 8H, $NCH_{2\ Pro}$ + 2 × NCH_3), 3.21–3.14, 3.06–2.94 (2 × m, 1H, $CH_{2\ Hum}$), 2.32–2.21 (m, 2H, 2 × $C_{\beta}H_{2\ Pro}$), 2.01–1.83 (m, 9H, 2 × $C_{\beta}H_{2\ Pro}$ + 2 × $C_{\gamma}H_{2\ Pro}$ + $CH_{3\ acetyl}$), 1.62–1.62 (m, 15H, Cp*), 1.38 (d, $^{3}J_{HH}$ = 7.3 Hz, 3H, 2 × $CH_{3\ Ala}$), 1.32–1.27 (m, 9H, 3 × $CH_{3\ Ala}$). ^{13}C{^{1}H} NMR (H_2O:D_2O, 9:1, 75 MHz) δ 176.3, 174.9, 174.8, 173.9, 173.7, 173.7, 172.5, 171.1, 163.1, 162.6 (10 × C=O), 131.0 ($C_{\gamma\ Hum}$), 123.3 ($C_{\delta}H_{\ Hum}$), 94.2, 91.5 (2 × C_{Cp}), 60.1 ($C_{\alpha}H$), 49.7, 48.5, 47.7, 47.6 (4 × $C_{\alpha}H$), 36.4, 35.1 (2 × NCH_3 (not well resolved, assigned by ^{1}H-^{13}C HSQC)), 29.2, 29.2 (2 × $C_{\beta}H_{2\ Pro}$), 26.1 ($CH_{2\ Hum}$), 24.6, 24.6 (2 × $C_{\gamma}H_{2\ Pro}$), 21.3 ($CH_{3\ acetyl}$), 20.7, 20.6 (2 × $CH_{3\ Leu}$), 16.5, 16.0, 15.8, 15.3, 15.2 (5 × $CH_{3\ Ala}$), 8.2, 8.1 (2 × $C_{\delta}H_{3\ Ile}$), 8.5, 8.3 (2 × Cp–CH_3), not resolved (C–Ir). HRMS (ESI^{+}): calcd for $[M-2Cl]^{2+}$ 515.7223, found 515.7237

Synthesis of ALAḦALA–Ir. Light orange powder (21 mg, 39±13%).^{1}H NMR (H_2O:D_2O, 9:1, 300 MHz) δ not resolved (m, 7H, 7 × CONH), 7.13–7.10 (m, 1H, $C_{\delta}H_{\ Hum}$), 4.64–4.55 (m, 1H, $C_{\alpha}H_{\ Hum}$), 4.33–4.18 (m, 6H, 6 × $C_{\alpha}H$), 3.67–3.60 (m, 6H, 2 × NCH_3), 3.23–3.18, 3.10–2.96 (2 × m, 1H, $CH_{2\ Hum}$),

1.98 (s, 3H, $CH_{3\ acetyl}$), 1.64–1.53 (m, 21H, Cp* + 2 × ($C_\beta H_{2\ Leu}$ + $C_\gamma H_{Leu}$)), 1.40–1.31 (m, 12H, 4 × $CH_{3\ Ala}$), 0.92–0.84 (m, 12H, 4 × $CH_{3\ Leu}$). $^{13}C\{^1H\}$ NMR (H_2O:D_2O, 9:1, 75 MHz) δ 175.6, 174.5, 174.4, 174.2, 163.2, 162.7 (6 × C=O), 131.0 ($C_{\gamma\ Hum}$), 123.3 ($C_\delta H_{Hum}$), 88.5 (C_{Cp}), 52.1, 50.0, 49.8, 49.8, 48.6 (5 × $C_\alpha H$), 39.7, 39.4 (2 × $C_\beta H_{2\ Leu}$), 36.8, 34.2 (2 × broad s, NCH_3), 25.9 ($CH_{2\ Hum}$), 24.2 ($C_\beta H_{2\ Leu}$), 22.1 ($CH_{3\ Leu}$), 21.5 ($CH_{3\ acetyl}$), 20.7, 20.6 (2 × $CH_{3\ Leu}$), 16.5, 16.2, 16.1 (3 × $CH_{3\ Ala}$), 8.5, 8.3 (2 × Cp–CH_3), not resolved (C–Ir). HRMS (ESI^+): calcd for $[M – 2Cl]^{2+}$ 531.7536, found 531.7539

Synthesis of AIAӒAIA–Ir. Light orange powder (40 mg, 74±11%). ^1H NMR (H_2O:D_2O, 9:1, 300 MHz) δ not resolved (m, 7H, 7 × CONH), 7.11–7.09 (m, 1H, $C_\delta H_{Hum}$), 4.62–4.57 (m, 1H, $C_\alpha H_{Hum}$), 4.36–4.10 (m, 6H, 6 × $C_\alpha H$), 3.83–3.55 (m, 6H, 2 × NCH_3), 3.23–3.18, 3.06–2.98 (2 × m, 1H, $CH_{2\ Hum}$), 1.97 (s, 3H, $CH_{3\ acetyl}$), 1.82–1.81 (m, 2H, 2 × $C_\beta H_{Ile}$), 1.64–1.57 (m, 15H, Cp*), 1.38–1.08 (m, 16H, 4 × $CH_{3\ Ala}$ + 2 × $C_\gamma H_{2\ Ile}$), 0.93–0.81 (m, 12H, 4 × $CH_{3\ Ile}$). $^{13}C\{^1H\}$ NMR (H_2O:D_2O, 9:1, 75 MHz) δ 175.4, 174.7, 174.0, 173.2, 163.1, 162.7 (6 × C=O), 131.0 ($C_{\gamma\ Hum}$), 123.3 ($C_\delta H_{Hum}$), 88.5 (C_{Cp}), 58.1, 58.1 (2 × $C_\alpha H_{Ile}$), 51.8, 49.7, 48.6 (3 × $C_\alpha H$), 36.9, 36.2, 36.0, 34.3 (2 × NCH_3 + 2 × $C_\beta H_{Ile}$), 26.0 ($CH_{2\ Hum}$), 24.4 ($C_\gamma H_{2\ Ile}$), 21.4 ($CH_{3\ acetyl}$), 20.7, 20.6 (2 × $CH_{3\ Leu}$), 16.5, 16.3, 16.0, 14.7, 14.7, 14.6 (6 × $CH_{3\ Ala+Ile}$), 10.1, 10.0 (2 × $C_\delta H_{3\ Ile}$), 8.5, 8.3 (2 × Cp–CH_3), not resolved (C–Ir). HRMS (ESI^+): calcd for $[M – 2Cl]^{2+}$ 531.7536, found 531.7534

Synthesis of AFAӒAFA–Ir. Light orange powder (19 mg, 17±9%). ^1H NMR (H_2O:D_2O, 9:1, 300 MHz) δ 8.5–7.9 (m, 7H, 7 × CONH), 7.24–7.13 (m, 10H, $C_{Arm}H_{Phe}$), 6.92 (d, $C_\delta H_{Hum}$, 1H), 4.54–4.41 (m, 3H, $C_\alpha H_{Hum}$ and 2 × $C_\alpha H_{Phe}$), 4.3–4.0 (m, 4H, 4 × $C_\alpha H_{Ala}$), 3.57–3.37 (2 × s, 3H, NCH_3), 3.1–2.8 (6H, $C_\beta H_{2\ Hum}$ 2 × $C_\beta H_{2\ Phe}$), 1.86 (s, 3H, $CH_{3\ acetyl}$), 1.54–1.47 (m, 15H, Cp*), 1.3–1.0 (m, 12H, $CH_{3\ Ala}$). $^{13}C\{^1H\}$ NMR (H_2O:D_2O, 9:1, 75 MHz) δ not resolved (8 × C=O), not resolved ($C_\varepsilon H_{Hum}$), ~128 (12 × C_{Arom}, assigned by ^1H-^{13}C HSQC), not resolved ($C_{\gamma\ Hum}$), ~89 (2 × C_{Cp}), not resolved ($C_\delta H_{Hum}$), 54–50 (7 × C_α assigned by ^1H-^{13}C HSQC), ~36 (2 × $C_{\beta\ Phe}$), ~36 (2 × NCH_3), 25.0 ($C_\beta H_{2\ Hum}$), 22.0 ($CH_{3\ acetyl}$), ~16.0 (4 × $CH_{3\ Ala}$), not resolved (C–Ir). HRMS (ESI^+): calcd for $[M – 2Cl]^{2+}$ 565.7329, found 565.7390

Synthesis of AWAӒAWA–Ir. Light orange powder (5 mg, 4±1%). ^1H NMR (H_2O:D_2O, 9:1, 300 MHz) (not all peaks assigned, due to unresolved 2D NMRs) δ 7.68–7.66, 7.62–7.59, 7.56–7.54, 7.47–7.43, 7.38–7.36, 7.26–7.05, 6.85, 6.77 (m, 6H, $C_\delta H_{Hum}$ + CH_{Trp}), 4.59–4.42 (m), 4.32–4.21 (m), 4.32–4.21 (m), 4.16–4.06 (m), 3.57, 3.55, 3.50, 3.43 (4 × s, 6H, 4 × NCH_3 (peaks of isomers do not overlap)), 3.34–3.10 (m, 4H, 2 × $C_\beta CH_{2\ Trp}$), 3.00–2.93, 2.85–2.77 (2 × m, 1H, $CH_{2\ Hum}$), 1.85 (s, 3H, $CH_{3\ acetyl}$), 1.64, 1.59 (2 × s, 15H, 2 × Cp* (peaks of isomers do not overlap)), 1.35–1.21 (m, 9H, 3 × $CH_{3\ Ala}$), 1.10, 1.01 (2 × d, $^3J_{HH}$ = 7.3 Hz, 3H, 2 × $CH_{3\ Ala}$ (peaks of isomers do not overlap)). $^{13}C\{^1H\}$ NMR (H_2O:D_2O, 9:1, 75 MHz) δ not resolved. HRMS (ESI^+): calcd for $[M – 2Cl]^{2+}$ 604.7488, found 604.7503

Synthesis of ATAӒATA–Ir. Light orange powder (28 mg, 33±8%). ^1H NMR (H_2O:D_2O, 9:1, 300 MHz) δ 8.6–8.0 (m, 7H, 7 × CONH), 7.14–7.06 (m, $C_\delta H_{Hum}$, 1H), 4.56–4.52 (m, 1H, $C_\alpha H_{Hum}$) 4.24–

4.04 (m, 6H, 6 × $C_\alpha H$ and 2H, 2 × $C_\beta H_{Thr}$), 3.73, 3.69 (m, 3H, NCH_3), 3.2–3.0 (m, 2H, 2 × $C_\beta H_{2\,Hum}$), 1.87 (s, 3H, $CH_{3\,acetyl}$), 1.6–1.4 (m, 12H, $CH_{3\,Ala}$ and 3H, $C_\gamma H_{3\,Thr}$), 1.3–1.1 (m, 15H, Cp*), not resolved (2 × OH_{Thr}). $^{13}C\{^1H\}$ NMR (H_2O:D_2O, 9:1, 75 MHz) δ not resolved (8 × C=O), not resolved ($C_\varepsilon H_{Hum}$), not resolved ($C_{\gamma\,Hum}$), ~91 (2 × C_{Cp}), not resolved ($C_\delta H_{Hum}$ assigned by ^1H-^{13}C HSQC), 67–66 (2 × $C_\gamma H_3$ $_{Thr}$ assigned by ^1H-^{13}C HSQC), 59–57 (2 × CHOH assigned by ^1H-^{13}C HSQC), 51–49 (7 × $C_\alpha H$), 36.6, 35.4 (2 × NCH_3), not resolved ($C_\beta H_{2\,Hum}$), 21.4 ($CH_{3\,acetyl}$), ~18.1, ~16.1 (4 × $CH_{3\,Ala}$) 8.7, 8.1 (2 × Cp–CH_3), not resolved (C–Ir). HRMS (ESI$^+$): calcd for $[M-2Cl]^{2+}$ 519.7172, found 519.7165

Synthesis of ANAӦHANA–Ir. Light orange powder (14 mg, 17±5%). ^1H NMR (H_2O:D_2O, 9:1, 300 MHz) δ not resolved (m, 7H, 7 × CONH), 7.06–7.01 (m, 1H, $C_\delta H_{Hum}$), 4.6–4.5 (m, 3H, $C_\alpha H_{Hum+Asn}$), 4.3–4.1 (m, 4H, 4 × $C_\alpha H_{Ala}$), 3.65–3.50 (m, 6H, 2 × NCH_3), 3.2–3.0 (m, 2H, $CH_{2\,Hum}$), 2.7–2.5 (m, 2H, $C_\beta H_{2\,Asn}$), 1.98 (s, 3H, $CH_{3\,acetyl}$), 1.6–1.5 (m, 15H, Cp*), 1.3–1.2 (m, 12H, 4 × $CH_{3\,Ala}$). $^{13}C\{^1H\}$ NMR (H_2O:D_2O, 9:1, 75 MHz) δ 176–170 (8 × C=O), ~123 ($C_\delta H_{Hum}$), ~89 (C_{Cp}), 50–48 (4 × $C_\alpha H$), 36.1 (NCH_3 assigned by ^1H-^{13}C HSQC), 35.2, 35.0 ($C_\beta H_{2\,Asn}$), 34.4 (NCH_3), 26.1, 25.8 (2 × $CH_{2\,Hum}$), 21.3 ($CH_{3\,acetyl}$), ~16.2 (4 × $CH_{3\,Ala}$), 8.4 (Cp–CH_3), not resolved (C–Ir). HRMS (ESI$^+$): calcd for $[M-2Cl]^{2+}$ 532.7124, found 532.7148

Synthesis of AQAӦHAQA–Ir. Light orange powder (25 mg, 28±11%). ^1H NMR (H_2O:D_2O, 9:1, 300 MHz) δ 8.6–8.2 (m, 7H, 7 × CONH), 7.1–6.9 (m, 1H, $C_\delta H_{Hum}$), 4.55–4.50 (m, 1H, $C_\alpha H_{Hum}$), 4.3–4.1 (m, 6H, 6 × $C_\alpha H_{Ala+Gln}$), 3.6–3.4 (m, 6H, 2 × NCH_3), 3.2–2.9 (m, 2H, $CH_{2\,Hum}$), 2.52–2.43 (m, 4H, $C_\gamma H_2$ $_{Gln}$), 2.18–2.02 (m, 1H, $C_\beta H_{2\,Gln}$), 1.97 (s, 3H, $CH_{3\,acetyl}$), 2.0–1.8 (m, 2H, $C_\beta H_{2\,Gln}$), 1.48–1.45 (m, 15H, Cp*), 1.35–1.25 (m, 12H, 4 × $CH_{3\,Ala}$). $^{13}C\{^1H\}$ NMR (H_2O:D_2O, 9:1, 75 MHz) δ not resolved (8 × C=O + 2 × CON), not resolved ($C_\delta H_{Hum}$), not resolved (C_{Cp}), 52–50 (7 × $C_\alpha H$ assigned by ^1H-^{13}C HSQC), ~36.2 (NCH_3 assigned by ^1H-^{13}C HSQC), 31.0, 30.9 ($C_\gamma H_{2\,Gln}$), not resolved (2 × $CH_{2\,Hum}$ + 2 × $C_\beta H_{2\,Gln}$), 21.5 ($CH_{3\,acetyl}$), 16.4–16.0 ($CH_{3\,Ala}$), 8.6, 8.5 (2 × Cp–CH_3), not resolved (C–Ir). HRMS (ESI$^+$): calcd for $[M-2Cl]^{2+}$ 546.7281, found 546.7274

Synthesis of ARAӦHARA–Ir. Light orange powder (31 mg, 27±12%). ^1H NMR (H_2O:D_2O, 9:1, 300 MHz) δ not resolved (m, 12H, 7 × CONH + $CN_3H_{5\,Arg}$), 7.0 (s, 1H, $C_\delta H_{Hum}$), 4.6–4.4 (m, 1H, $C_\alpha H_{Hum}$), 4.3–4.0 (m, 6H, 6 × $C_\alpha H$), 3.6–3.5 (m, 6H, 2 × NCH_3), 3.2–3.0 (m 6H, $C_\beta H_{2\,Hum}$ + 2 × $C_\gamma H_{2\,Arg}$), 1.88 (s, 3H, $CH_{3\,acetyl}$), 1.80–1.4 (m, 23H, 2 × $C_\beta H_{2\,Arg}$ + 2 × $C_\delta H_{2\,Arg}$ + Cp*) 1.4–1.3 (m, 12H, 4 × $CH_{3\,Ala}$). $^{13}C\{^1H\}$ NMR (H_2O:D_2O, 9:1, 75 MHz) δ 175.7, 175.6, 174.7, 174.5, 174.2, 173.4, 173.0, 171.5 (8 × C=O), not resolved ($C_\varepsilon H_{Hum}$), 131.2 ($C_{\gamma\,Hum}$), 118.1 ($C_\delta H_{Hum}$), 88.5 (Cp*), 53–48 (7 × $C_\alpha H$ assigned by ^1H-^{13}C HSQC), 40.4 (2 × $C_\delta H_{2\,Arg}$), 34.3, 34.2 (2 × NCH_3 assigned by ^1H-^{13}C HSQC), 28.1, 27.8 (2 × $C_\beta H_{2\,Arg}$), 25.7 ($C_\beta H_{2\,Hum}$), 24.3, 24.2 (2 × $C_\gamma H_{2\,Lys}$), 21.5 ($CH_{3\,acetyl}$), ~16 (4 × $CH_{3\,Ala}$) not resolved (C–Ir). HRMS (ESI$^+$): calcd for $[M-2Cl]^{3+}$ 383.5172, found 383.5154

1.4. Catalytic experiments

General procedure for catalytic hydrogenation of acetophenone. Stock solutions of acetophenone (10 mM in 50 mM citrate buffer pH 3, containing 20% *t*BuOH as co-solvent) and of the iridium compound (10 mM in 20 mM citrate buffer pH 3, containing 20% *t*BuOH as co-solvent) were prepared. Acetophenone (1.0 mL stock solution, 10 µmol) was added to a 9 mL microwave vial and sealed with a septum. The solution was pre-heated to 40 °C and H_2 was bubbled through the solution for 10 min. The iridium compound (10 µL stock solution, 0.10 µmol) was added to the mixture and kept at 40 °C. Aliquots (0.01 mL) were taken at specific times, diluted with iPrOH (1 mL) and analyzed by GC.

Enantiomeric excess was determined by extracting the reaction mixture after 24 h with Et_2O (3 × 1 mL). The organic layers were combined, dried over Na_2SO_4 and evaporated to dryness. (S)-(–)-Mosher's acid chloride (0.10 mL, 0.13 M in dry $CDCl_3$, 13 µmol) and dry pyridine (3 µL) were added to the residue and sonicated for 1 h at 35 °C. $CDCl_3$ (0.4 mL) was added and the sample was analyzed by ^1H NMR spectroscopy. Alternatively, aliquots (0.01 mL) were taken at specific times, diluted with *i*PrOH (1 mL) and analyzed by chiral GC. Both methods showed racemic mixtures of *R*- and *S*-phenethyl alcohol.

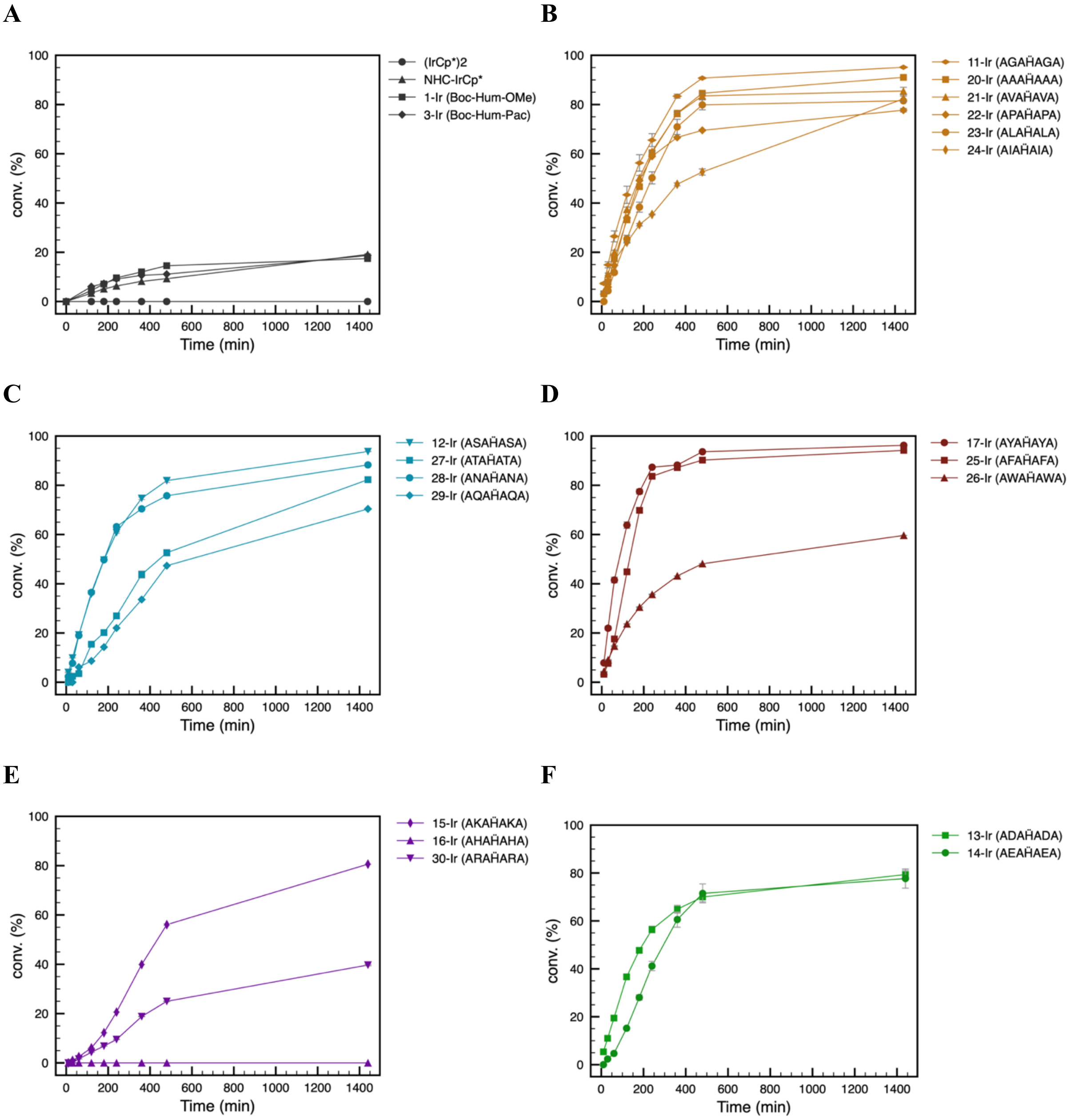

Figure S1. Conversion profiles for the hydrogenation of acetophenone to 1-phenylethanol using the Ir-functionalized peptide. Panels show groups of catalytic runs including controls (A), as well as Ir-functionalized heptapapetides **ARAḦARA–Ir** with hydrophobic (B), polar (C), aromatic (D), positive charged (E), negative charged (F) amino acids X.

2. Conformational Analysis by Molecular Dynamics Simulations and CD Spectroscopy

All peptides, either with a central histidine or with the pre-carbene amino acid, were constructed using TLEAP, part of the AmberTools21 package.[S5] Thus, all peptides started from their linear form to avoid any undesired bias in the structure imposed from the starting point. To create the unnatural amino acid, Ḧ, the central histidine was modified using UCSF Chimera.[S6] Coordinates and bond angles for the metal

centre were extracted from a crystal structure. All peptides were subsequently solvated with a 5Å box and minimized before performing the molecular dynamics simulations for 20ns. All simulations were performed using OpenMM with the Amber14 and TIP3P forcefields. Simulations were kept at 300K. Simulations were visualized using either PyMOL or UCSF Chimera tools.

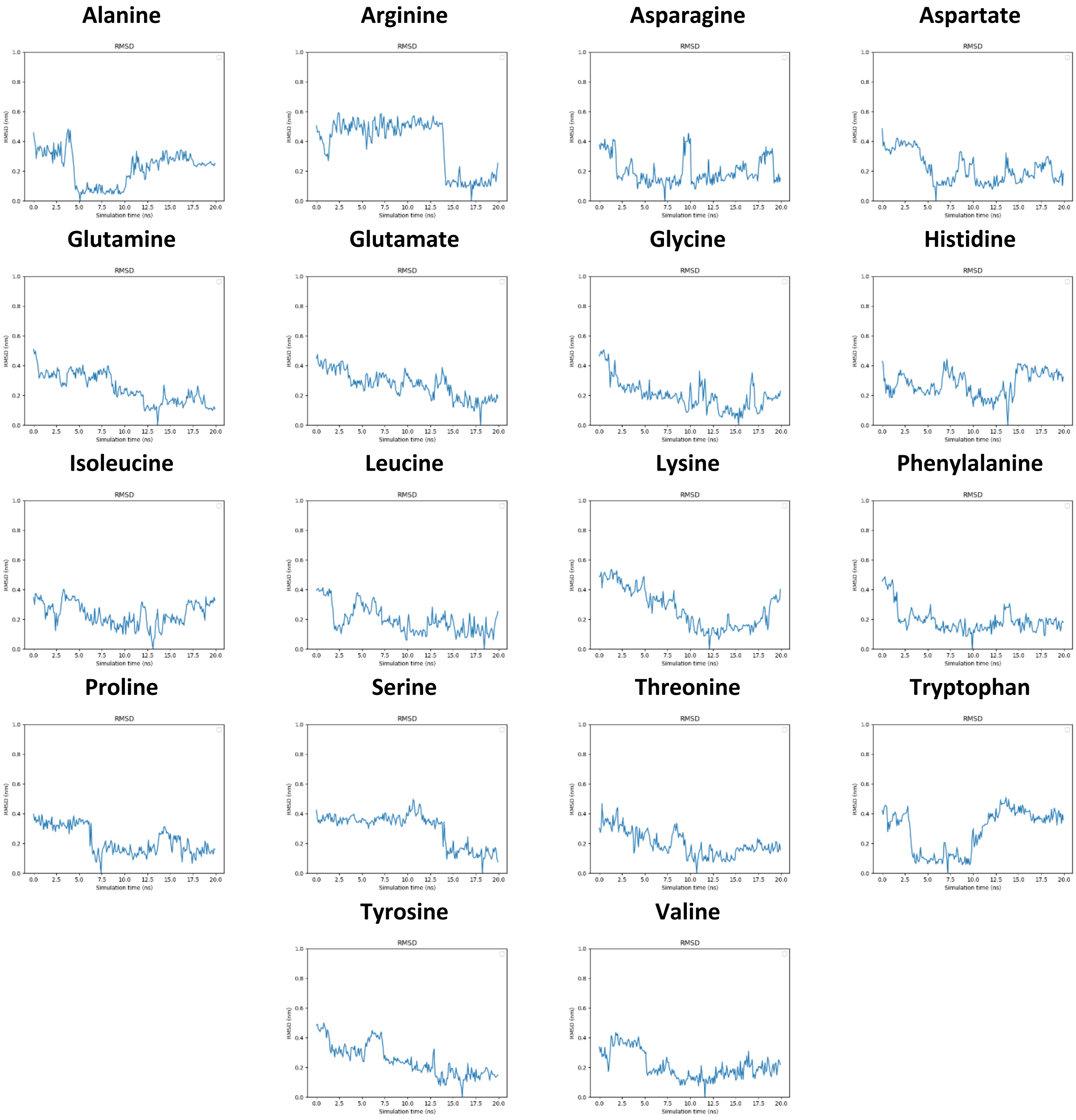

Figure S2. Root mean square deviation (RMSD) of the AXAHAXA peptides with variable amino acid X during the 20 ns simulations to the local minimum. Simulations were run using the openMM software.[S7] The RSMD distance was calculated using the MDTraj analysis package.[S8]

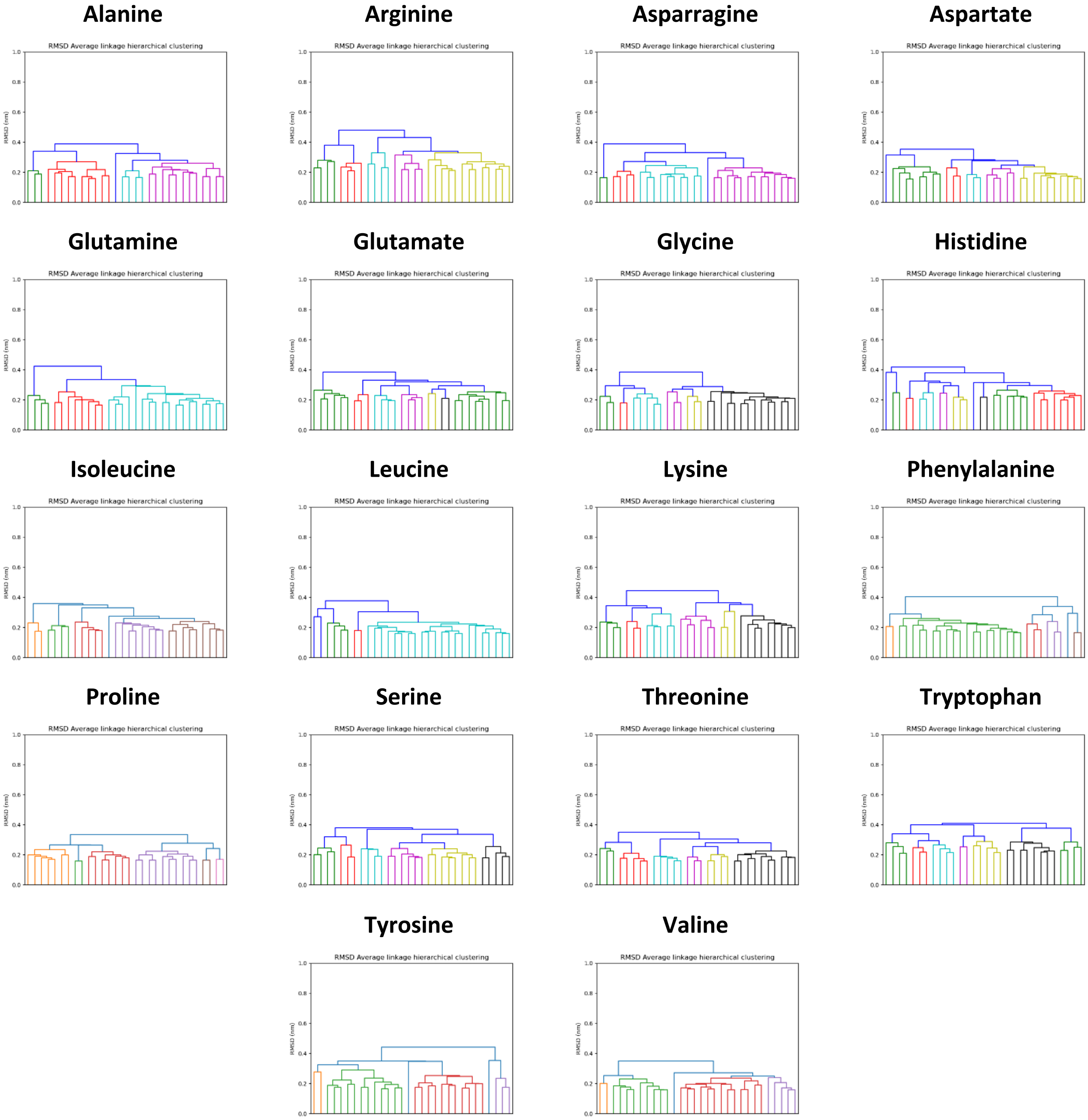

Figure S3. Hierarchical clustering of the different folding states of the AXAHAXA peptides during the MD simulations. Only relevant structures, with more than one occurrence during the simulations are shown. Different clusters are indicated with different color and their linkage in the RMSD distance (nm) shown with the blue lines. The clustering was calculated using the MDTraj analysis package.[S8]

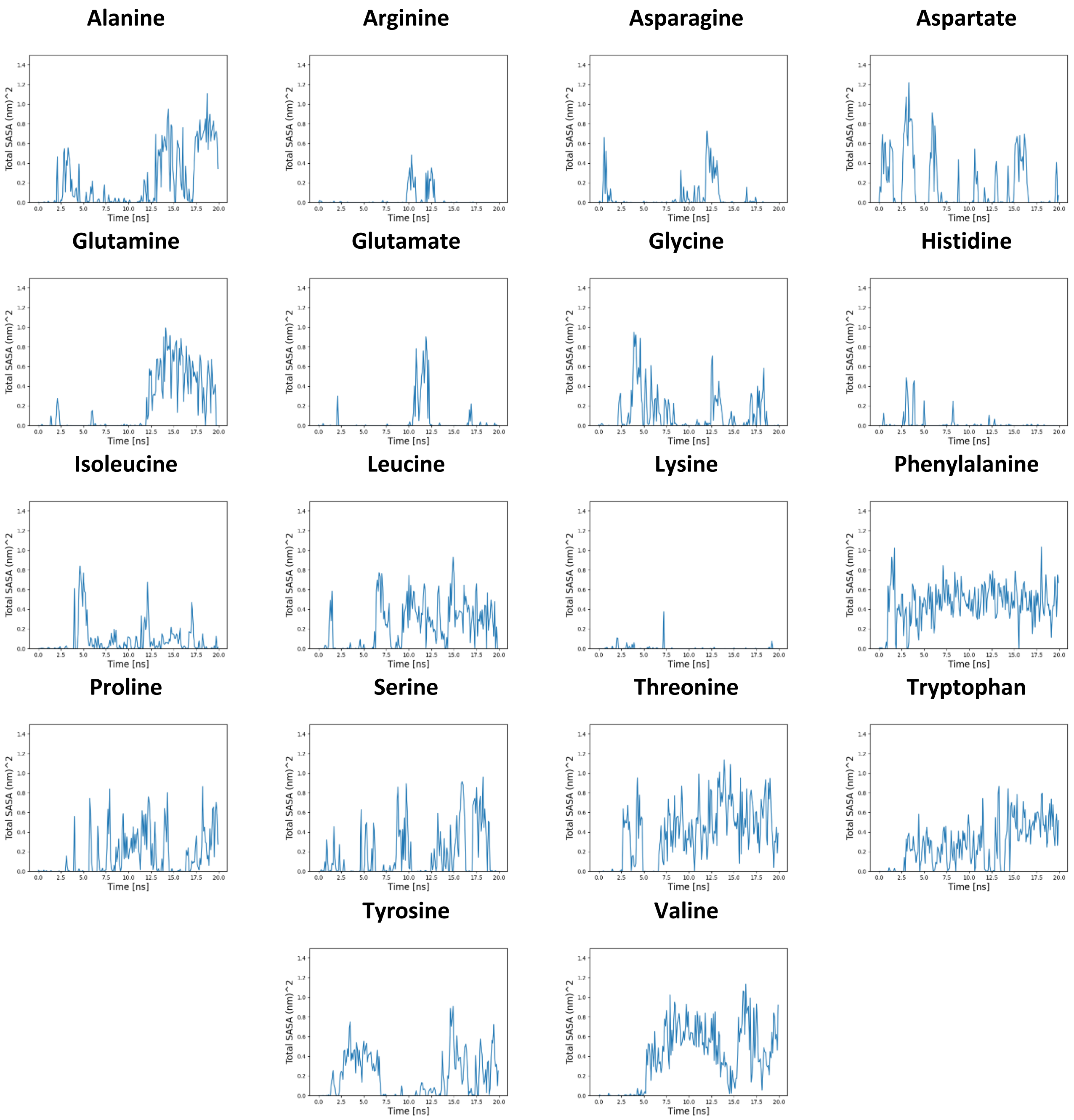

Figure S4. Evolution of the solvent-accessible surface area (SASA) of the central histidine for the 18 tested variants during the 20 ns simulations. SASA was calculated using the MDTraj analysis package.[S8]

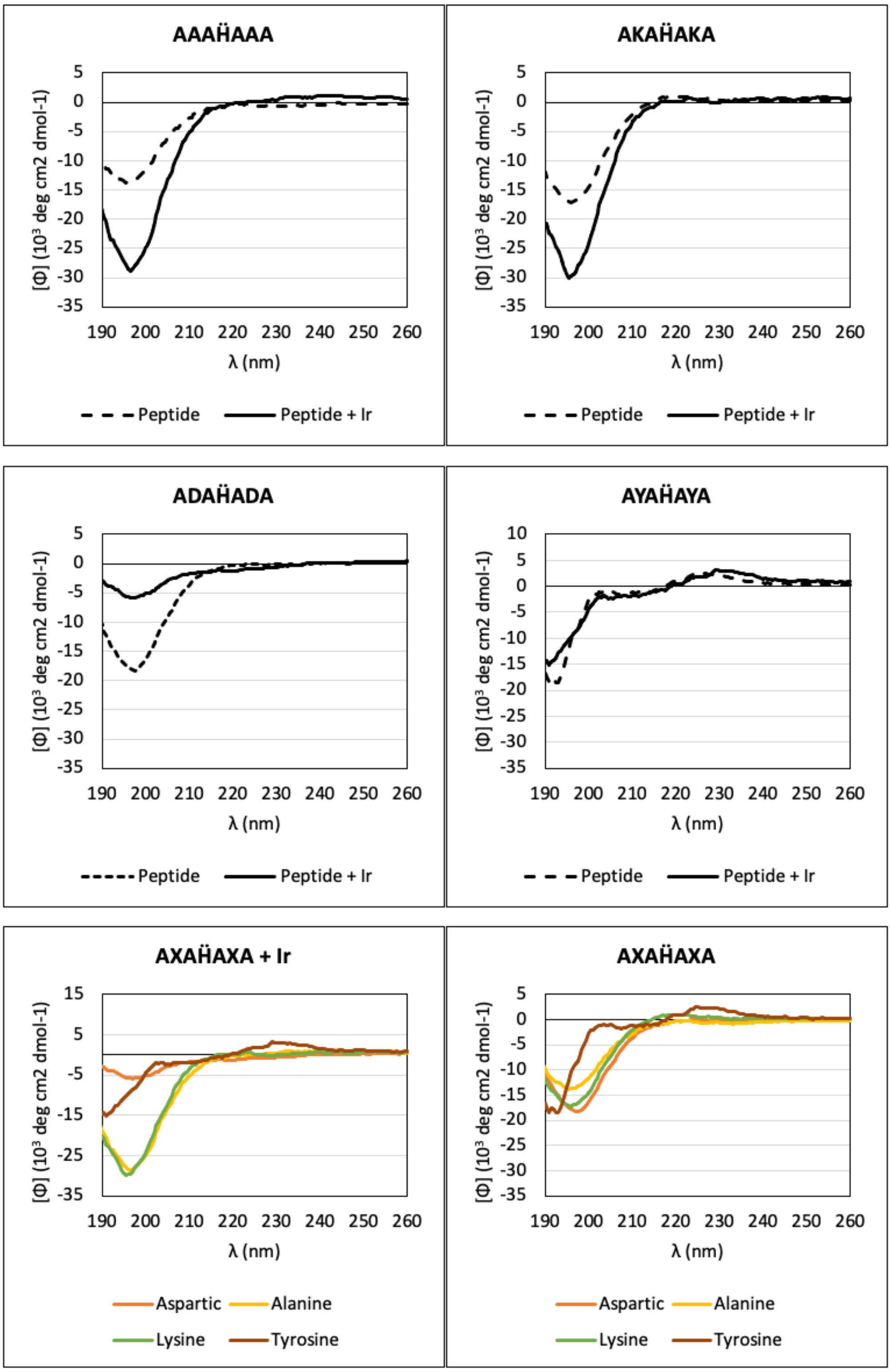

Figure S5: The CD spectra of all measured peptides indicate the absence of any specific secondary structure and the typical features associated with random coil structures, consistent with the results from molecular dynamics simulations. The spectra do not change significantly upon metalation of the peptides. The varying signal intensities may be attributed to either a slightly different folding, still as a random coil, and to the spectroscopic effect of the metal.

Table S1. Correlation of the different measured parameters.[a]

	TOF (h^{-1})	RMSD<0.1 nm (Time, ns)	His exposure > 0.1 (Time, ns)	pK_a His	Nº of clusters
**TOF (h^{-1})**	1.00				
**RMSD<0.1 nm (Time, ns)**	-0.01	1.00			
**His exposure > 0.1 (Time, ns)**	**0.41**	0.29	1.00		
**pK_a His**	0.12	0.27	-0.24	1.00	
**Nº of clusters**	-0.31	-0.18	-0.30	-0.11	1.00

[a] Both RMSD and Histidine exposure were measured as the amount of time the structure was at either less than 0.1 nm RMSD of the local minimum or with an exposure of more than 0.1 nm^2. The pK_a of the histidine was approximated by using the PROPKA software[S9] with the structure corresponding to the energy minimum.

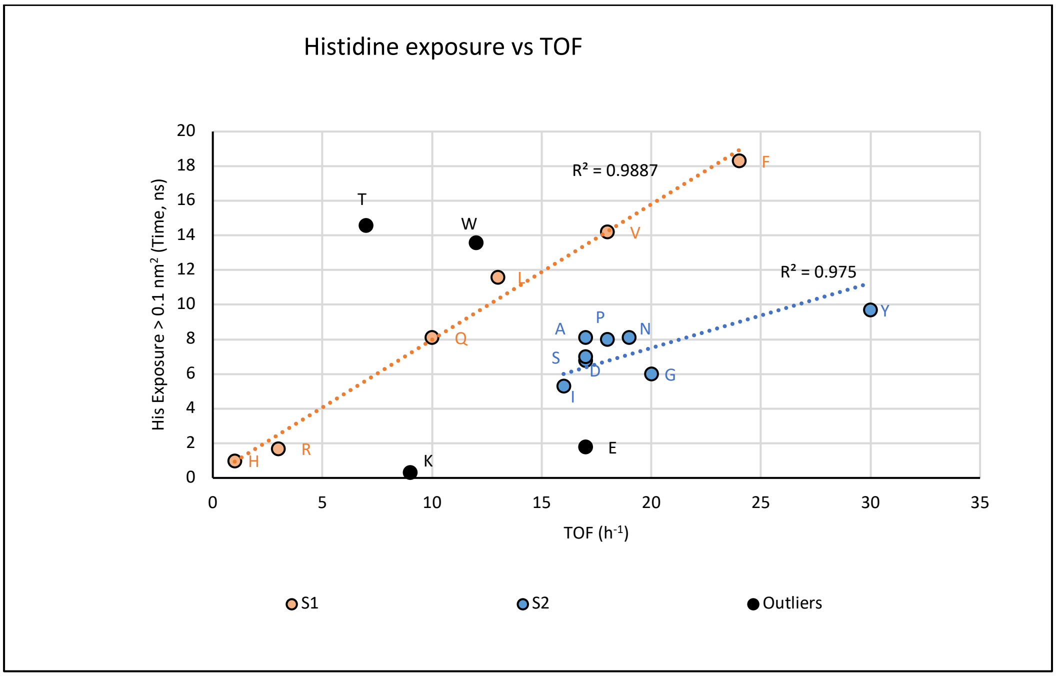

Figure S6. Correlation between the time of histidine exposure, measured in ns, and the maximum turnover frequency for the 18 AXAHAXA heptapeptides.

AKAḦAKA-Ir

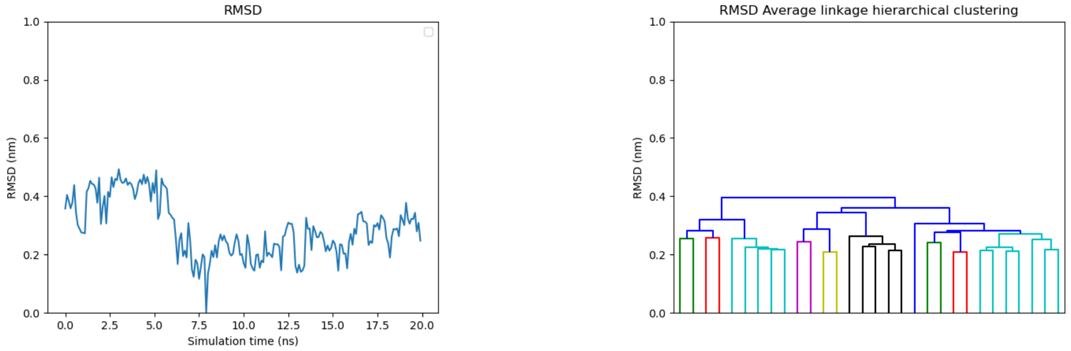

AHAḦAHA-Ir

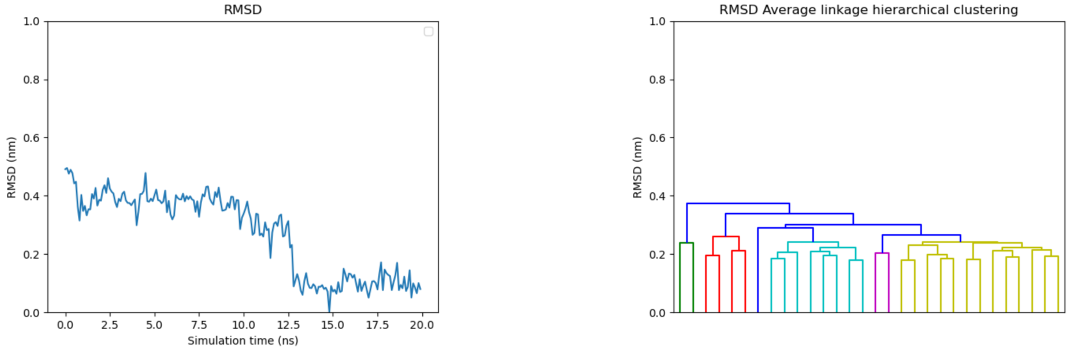

AWAḦAWA-Ir

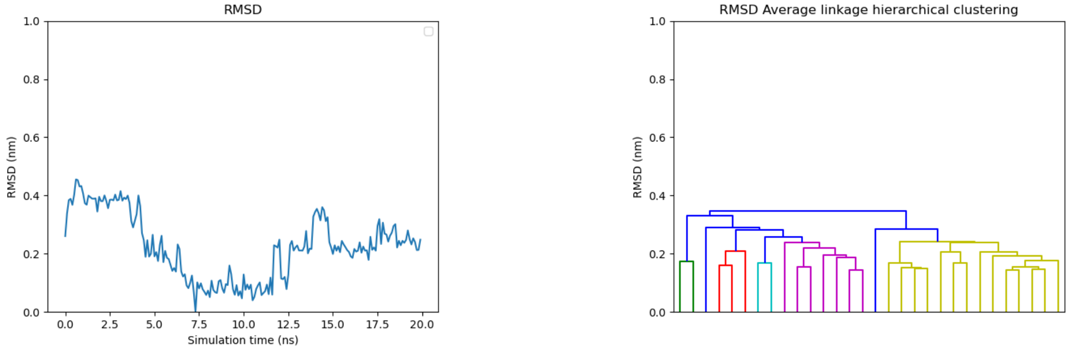

AYAḦAYA - Ir

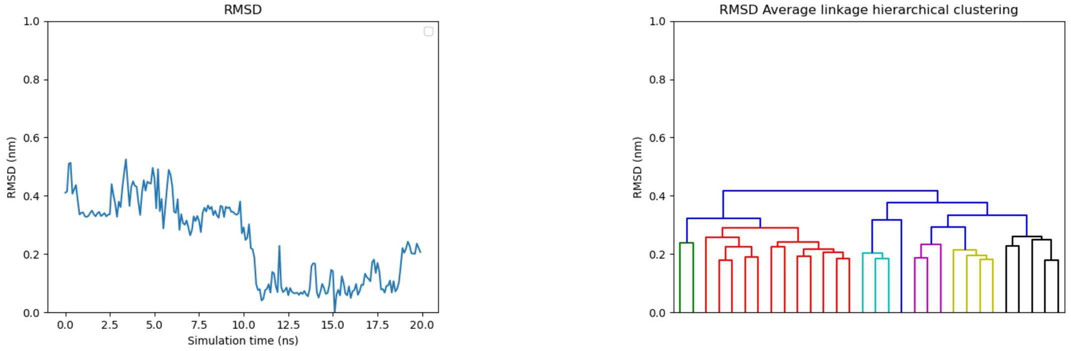

Figure S7: Summary of the results obtained running the simulations with **Ḧ–Ir** replacing the central histidine. Simulations were run in OpenMM[S5] and forcefield modification file, as well as topology and coordinate files were created using TLEAP, included in the AmberTools software package.[S5] The **Ḧ–Ir** geometry was defined from a related crystal structure.[S10]

3. References

(S1) F. Schmitt, K. Donnelly, J. K. Muenzner, T. Rehm, V. Novohradsky, V. Brabec, J. Kasparkova, M. Albrecht, R. Schobert and T. Mueller, *J. Inorg. Biochem.,* 2016, **163**, 221–228.

(S2) A. Monney, E. Alberico, Y. Ortin, H. Müller-Bunz, S. Gladiali and M. Albrecht, *Dalton Trans.* 2012, **41**, 8813–8821.

(S3) D. G. H.; Hetterscheid and J. N. H. Reek, *Chem. Commun.,* 2011, **47**, 2712–2714.

(S4) D. E. Ward and C. K. Rhee, *Tetrahedron Lett.,* 1991, **32**, 7165–7166.

(S5) D.A. Case, H.M. Aktulga, K. Belfon, I.Y. Ben-Shalom, S.R. Brozell, D.S. Cerutti, T.E. Cheatham, III, V.W.D. Cruzeiro, T.A. Darden, R.E. Duke, G. Giambasu, M.K. Gilson, H. Gohlke, A.W. Goetz, R. Harris, S. Izadi, S.A. Izmailov, C. Jin, K. Kasavajhala, M.C. Kaymak, E. King, A. Kovalenko, T. Kurtzman, T.S. Lee, S. LeGrand, P. Li, C. Lin, J. Liu, T. Luchko, R. Luo, M. Machado, V. Man, M. Manathunga, K.M. Merz, Y. Miao, O. Mikhailovskii, G. Monard, H. Nguyen, K.A. O'Hearn, A. Onufriev, F. Pan, S. Pantano, R. Qi, A. Rahnamoun, D.R. Roe, A. Roitberg, C. Sagui, S. Schott-Verdugo, J. Shen, C.L. Simmerling, N.R. Skrynnikov, J. Smith, J. Swails, R.C. Walker, J. Wang, H. Wei, R.M. Wolf, X. Wu, Y. Xue, D.M. York, S. Zhao, and P.A. Kollman (2021), Amber 2021, University of California, San Francisco.

(S6) E. F. Pettersen, T. D. Goddard, C. C. Huang, G. S. Couch, D. M. Greenblatt, E. C. Meng and T. E. Ferrin, J. *Comput. Chem*., 2004, **25**, 1605–1612

(S7) P. Eastman, J. Swails, J. D. Chodera, R. T. McGibbon, Y. Zhao, K. A. Beauchamp, L. P. Wang, A. C. Simmonett, M. P. Harrigan, C. D. Stern, R. P. Wiewiora, B. R. Brooks and V. S. Pande, *PLoS Comput. Biol.*, 2017, **13**, 1–17.

(S8) R. T. McGibbon, K. A. Beauchamp, M. P. Harrigan, C. Klein, J. M. Swails, C. X. Hernández, C. R. Schwantes, L.-P. Wang, T. J. Lane and V. S. Pande, *Biophys. J.*, 2015, **109**, 1528–1532.

(S9) M. H. M. Olsson, C. R. Søndergaard, M. Rostkowski and J. H. Jensen, *J. Chem. Theory Comput.*, 2011, **7**, 525–537.

(S10) R. Corberan, M. Sanau and E. Peris, *J. Am. Chem. Soc.*, 2006, **128**, 12, 3974–3979.

4. LC-MS and HR-MS Data of all apo-peptides AXAӇAXA and all metallopeptides AXAӇAXA–Ir

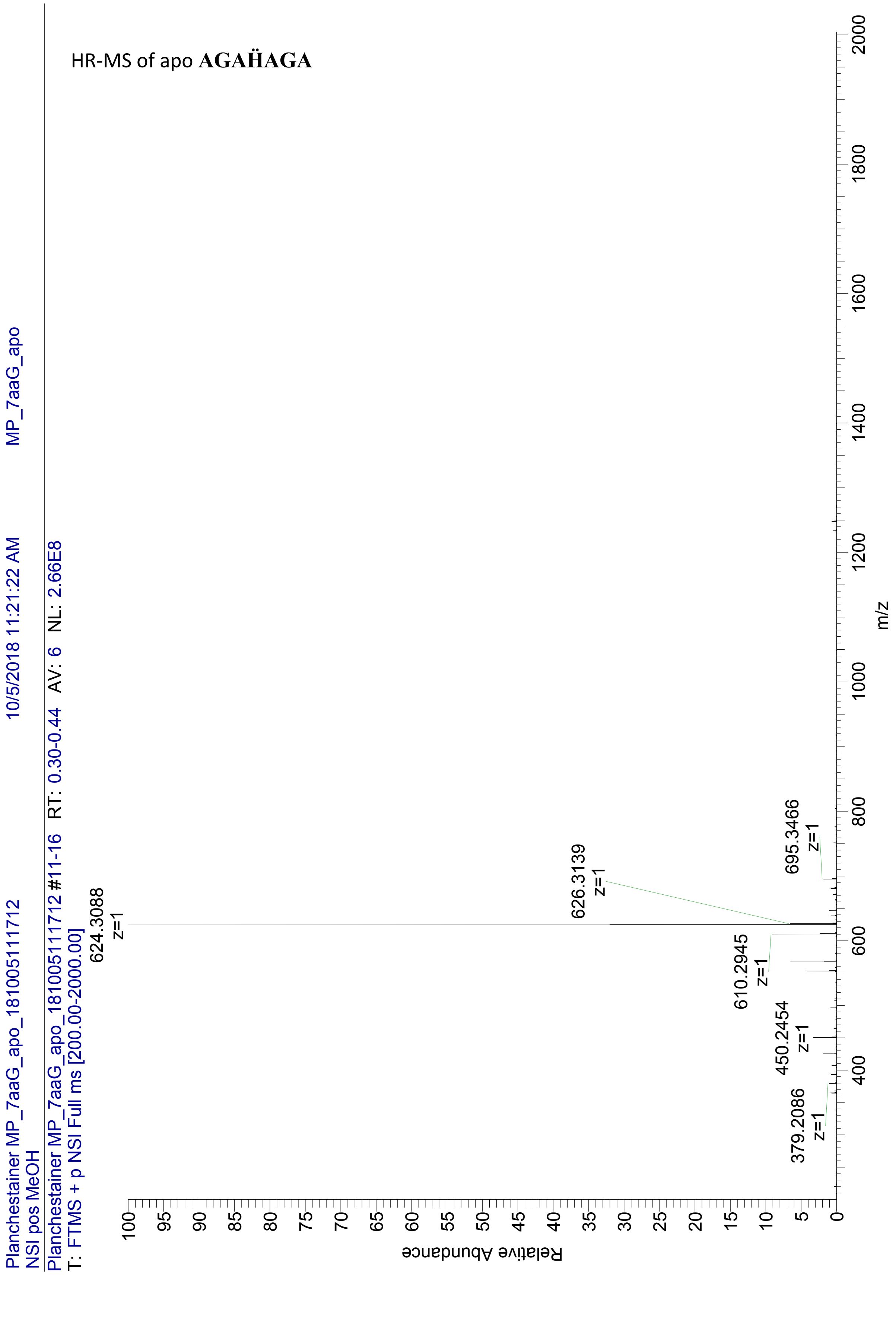

HR-MS of apo **AGAӇAGA**

4. LC-MS and HR-MS Data of all apo-peptides AXAḦAXA and all metallopeptides AXAḦAXA–Ir

HR-MS of apo **AGAḦAGA**

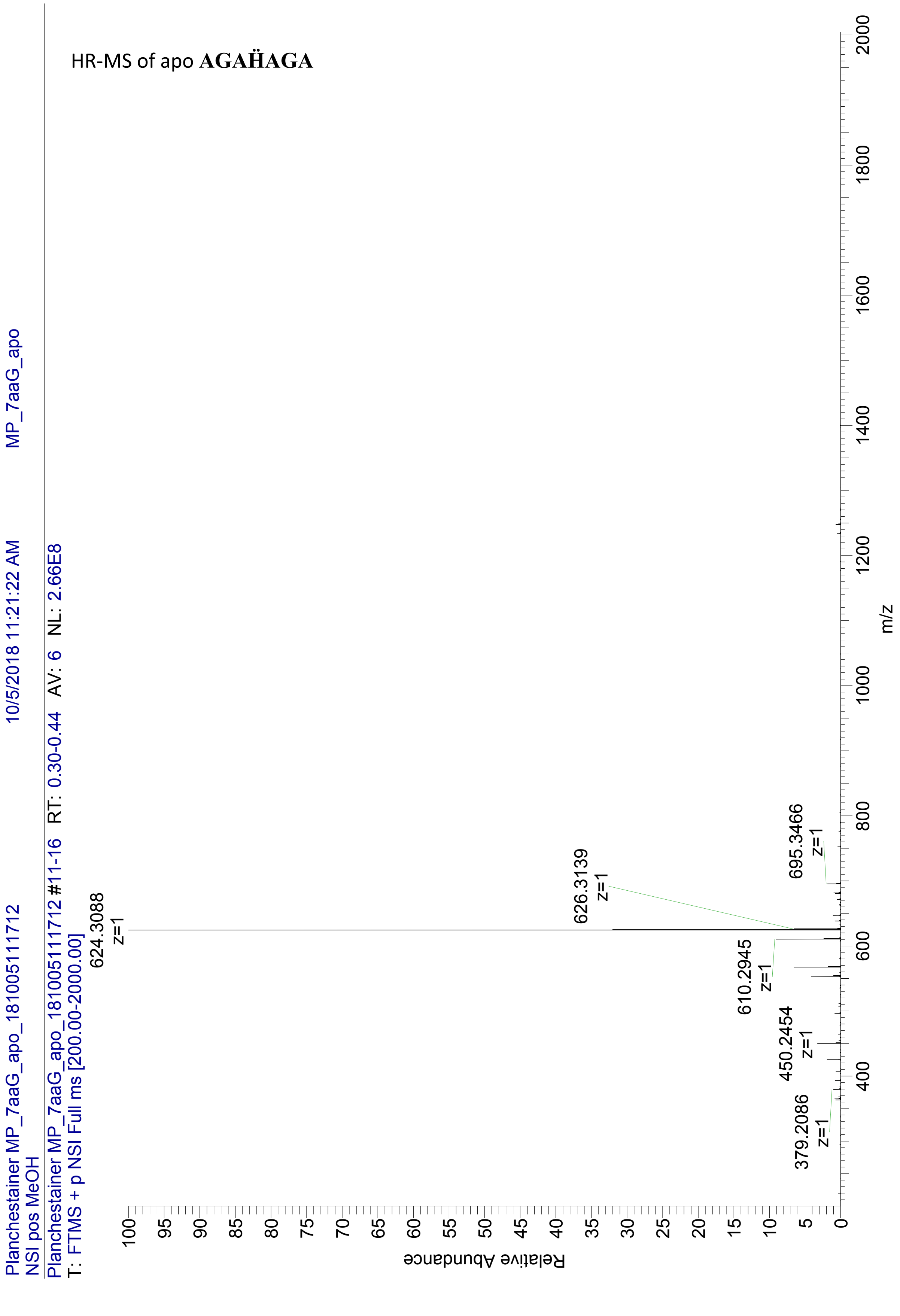

HR-MS 11-Ir (AGAḦAGA)

Planchestainer MP 7aaG_F3_181015084006 10/15/2018 12:39:00 PM Planchestainer MP 7aaG_F3
NSI pos MeOH_H2O

Planchestainer MP 7aaG_F3_181015084006 #1 RT: 0.02 AV: 1 NL: 1.20E8
T: FTMS + p NSI Full ms [150.00-2000.00]

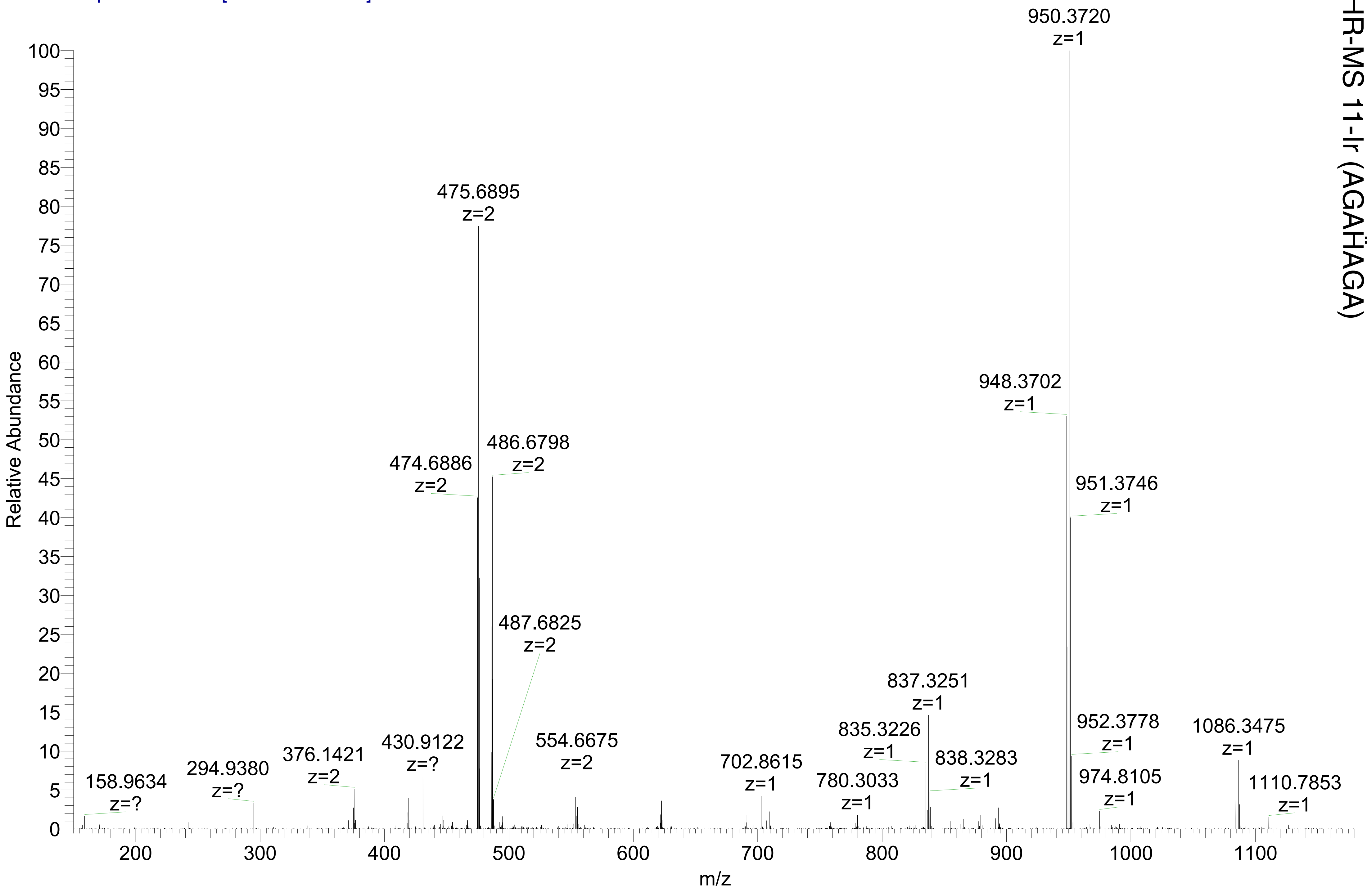

F:\Xcalibur\Data\Zamolo\MATTEO\KL-779-Gapo.raw 14.11.2018 10:16:02

RT :0.00-10.00 TIC MS KL-779-Gapo

LC-MS 11-apo (AGAḦAGA)

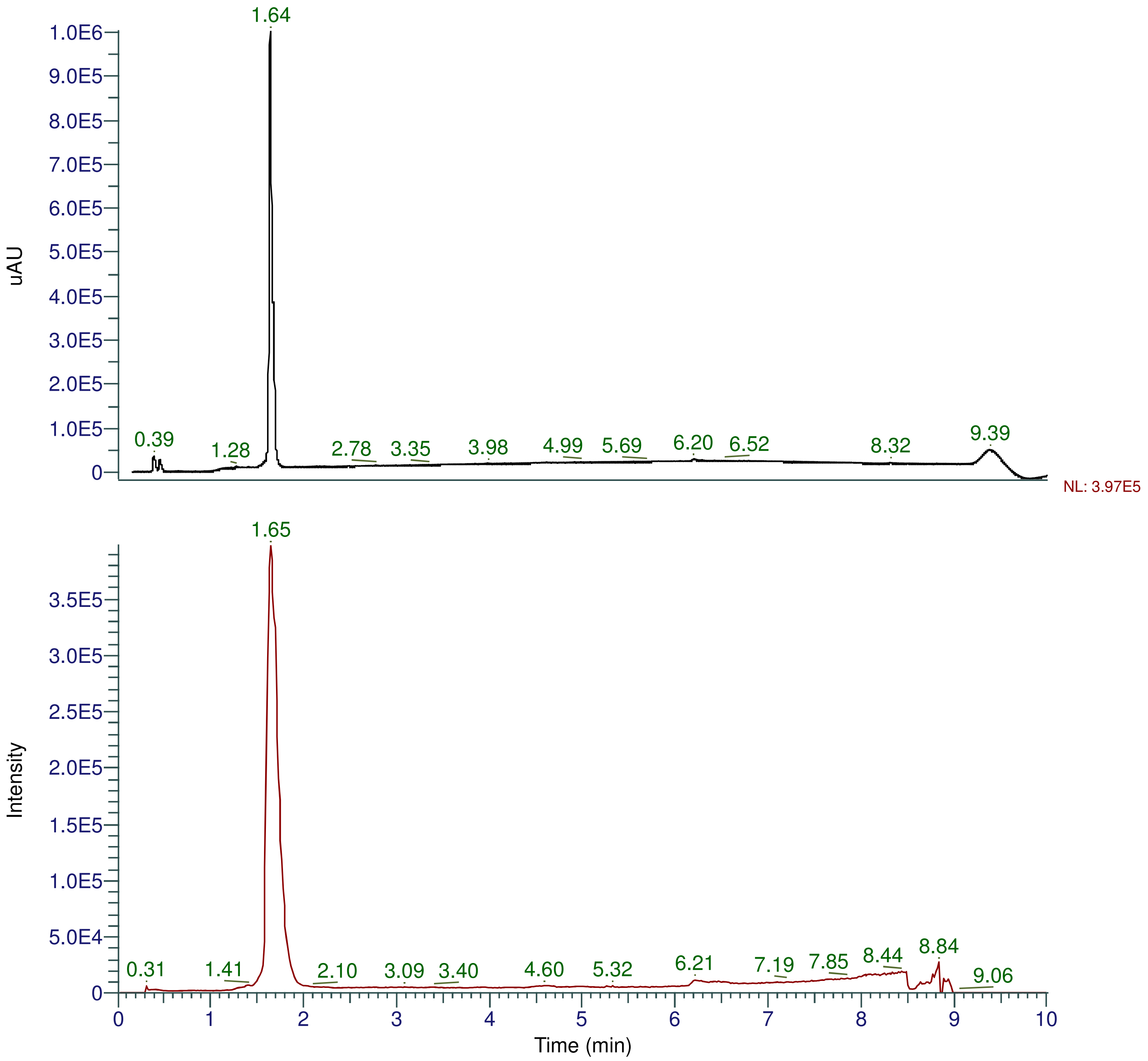

KL-779-Gapo #91 RT: 1.66 AV: 1 NL: 1.64E+005
T: ITMS + c ESI Full ms [150.00-2000.00]

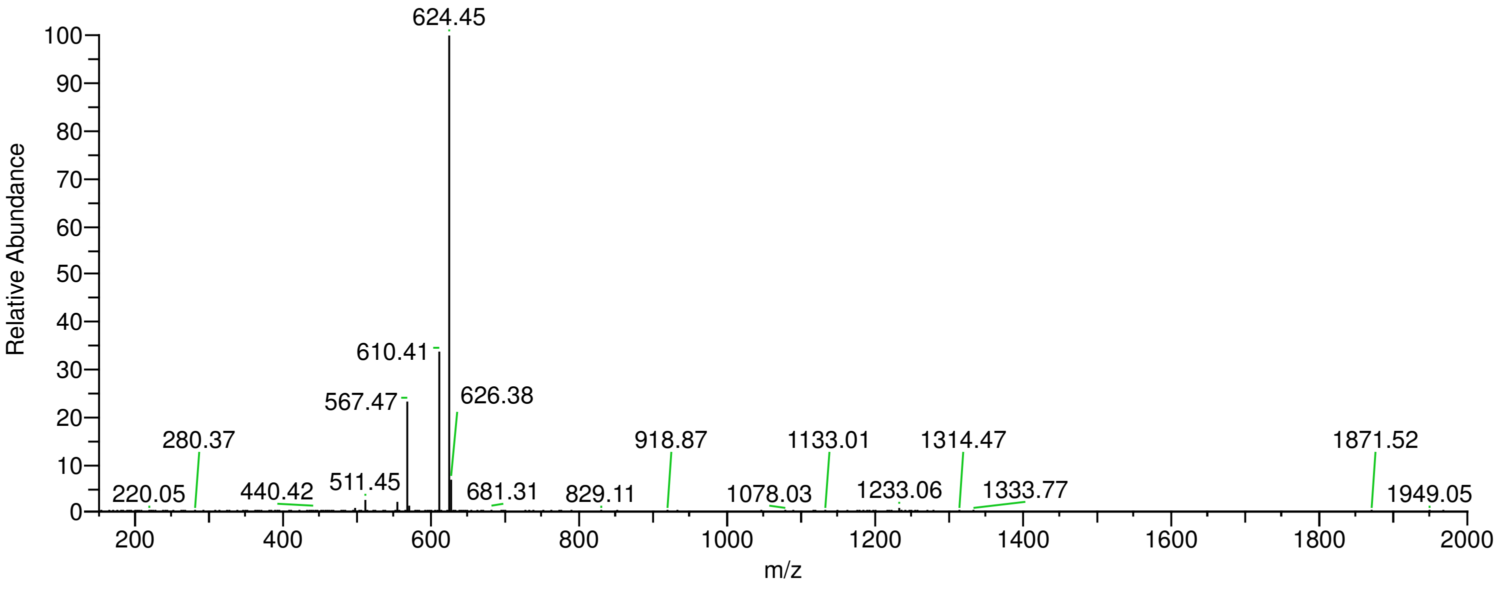

F:\Xcalibur\Data\Zamolo\MATTEO\KL-779-GIr1.raw 14.11.2018 10:18:33

RT :0.00-10.00 TIC MS KL-779-GIr1

LC-MS 11-Ir (AGAḦAGA)

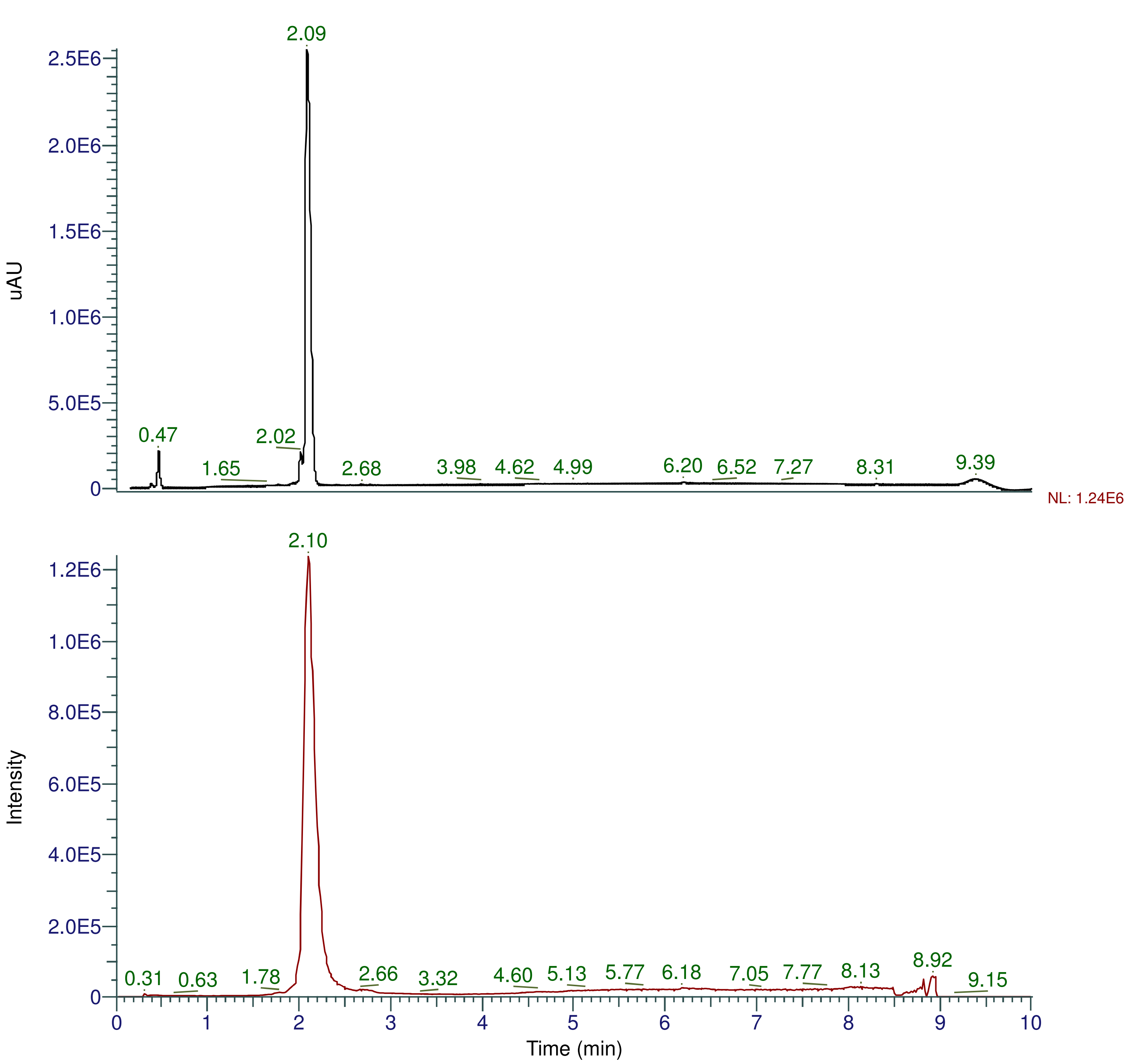

KL-779-GIr1 #117 RT: 2.10 AV: 1 NL: 3.57E+005
T: ITMS + c ESI Full ms [150.00-2000.00]

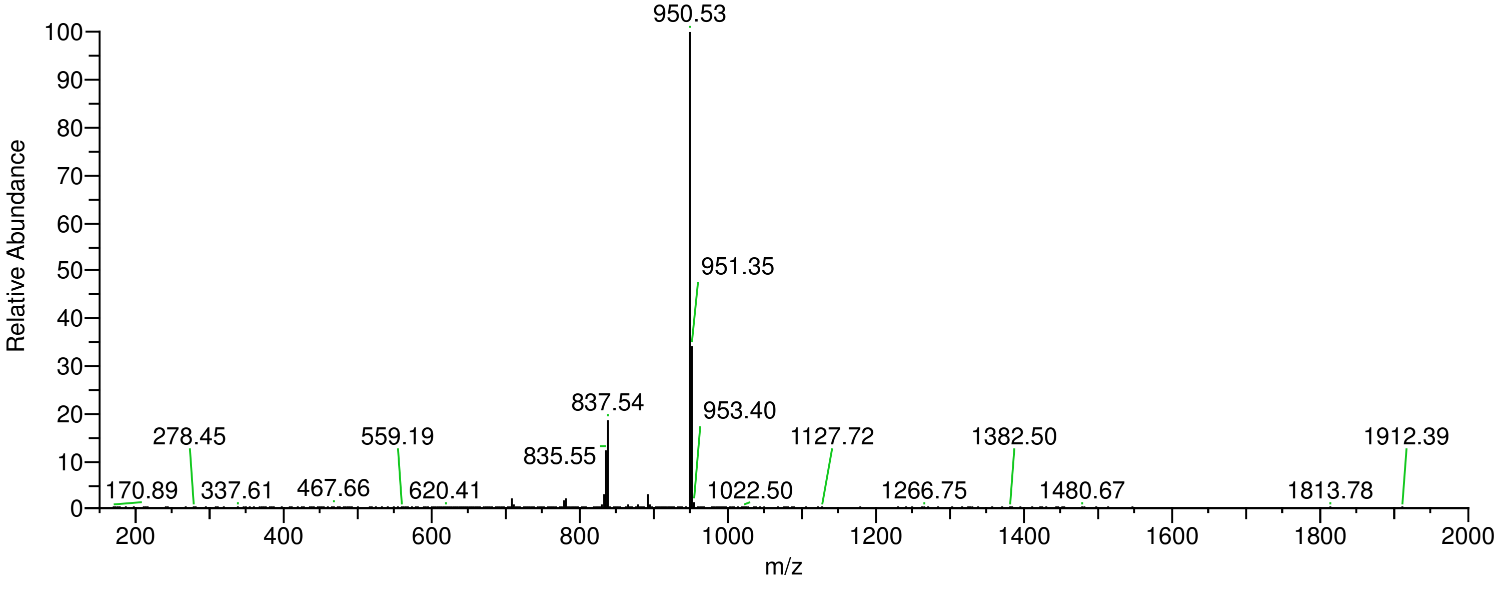

Matteo 7aaHumS_apo F2_180808090125 8/8/2018 10:15:49 AM Matteo 7aaHumS_apo F2
NSI pos ACN_H2O

Matteo 7aaHumS_apo F2_180808090125 #1-9 RT: 0.00-0.26 AV: 9 NL: 1.12E7
T: FTMS + p NSI Full ms [150.00-2000.00]

HR-MS 12-apo (ASAḦASA)

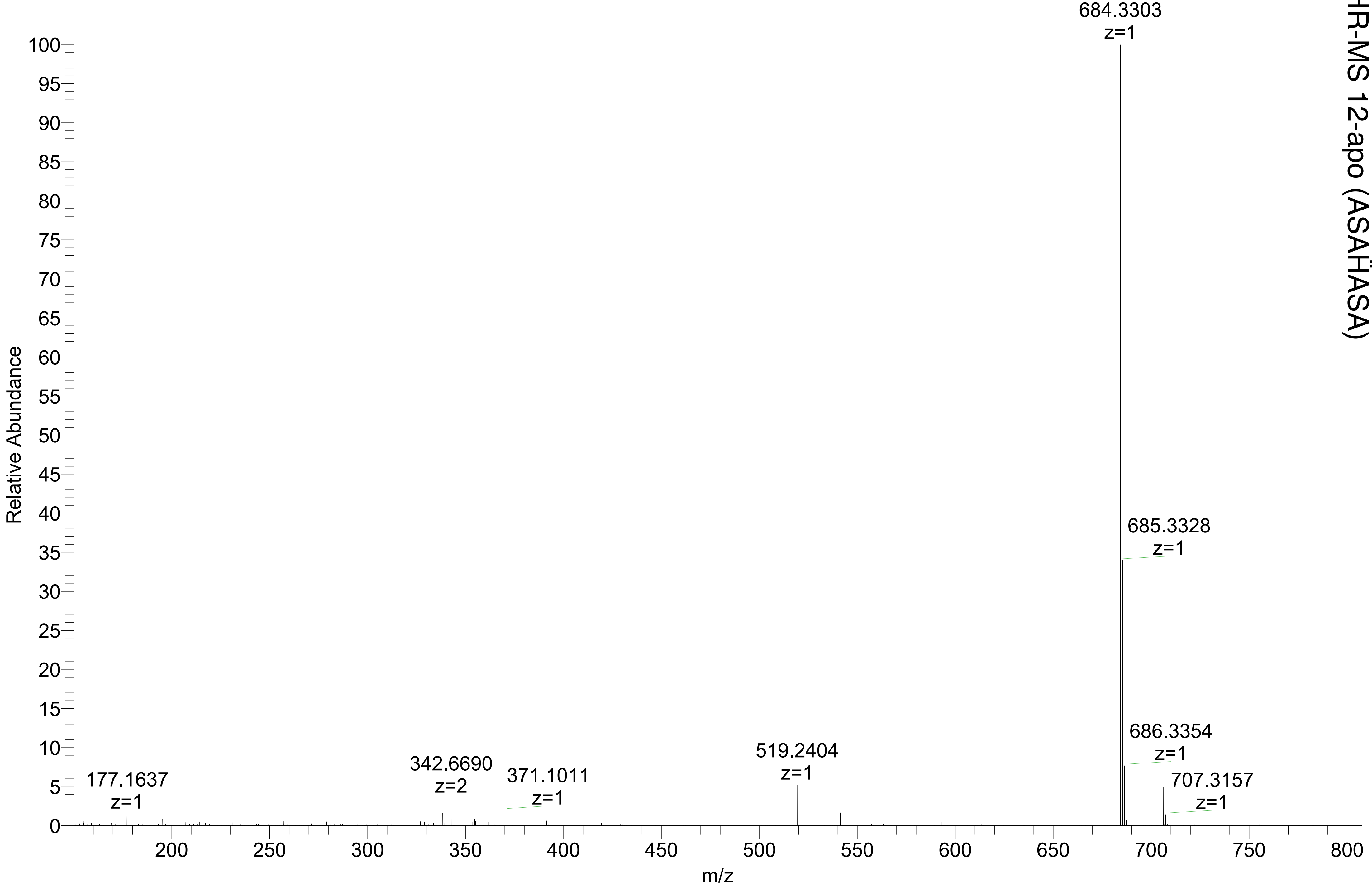

HR-MS 12-Ir (ASAḦASA)

Matteo 7aaHumS_Ir1 F2_180807100701 8/8/2018 8:33:56 AM Matteo 7aaHumS_Ir1 F2
NSI pos ACN_H2O

Matteo 7aaHumS_Ir1 F2_180807100701 #1-4 RT: 0.02-0.10 AV: 4 NL: 9.20E7
T: FTMS + p NSI Full ms [150.00-2000.00]

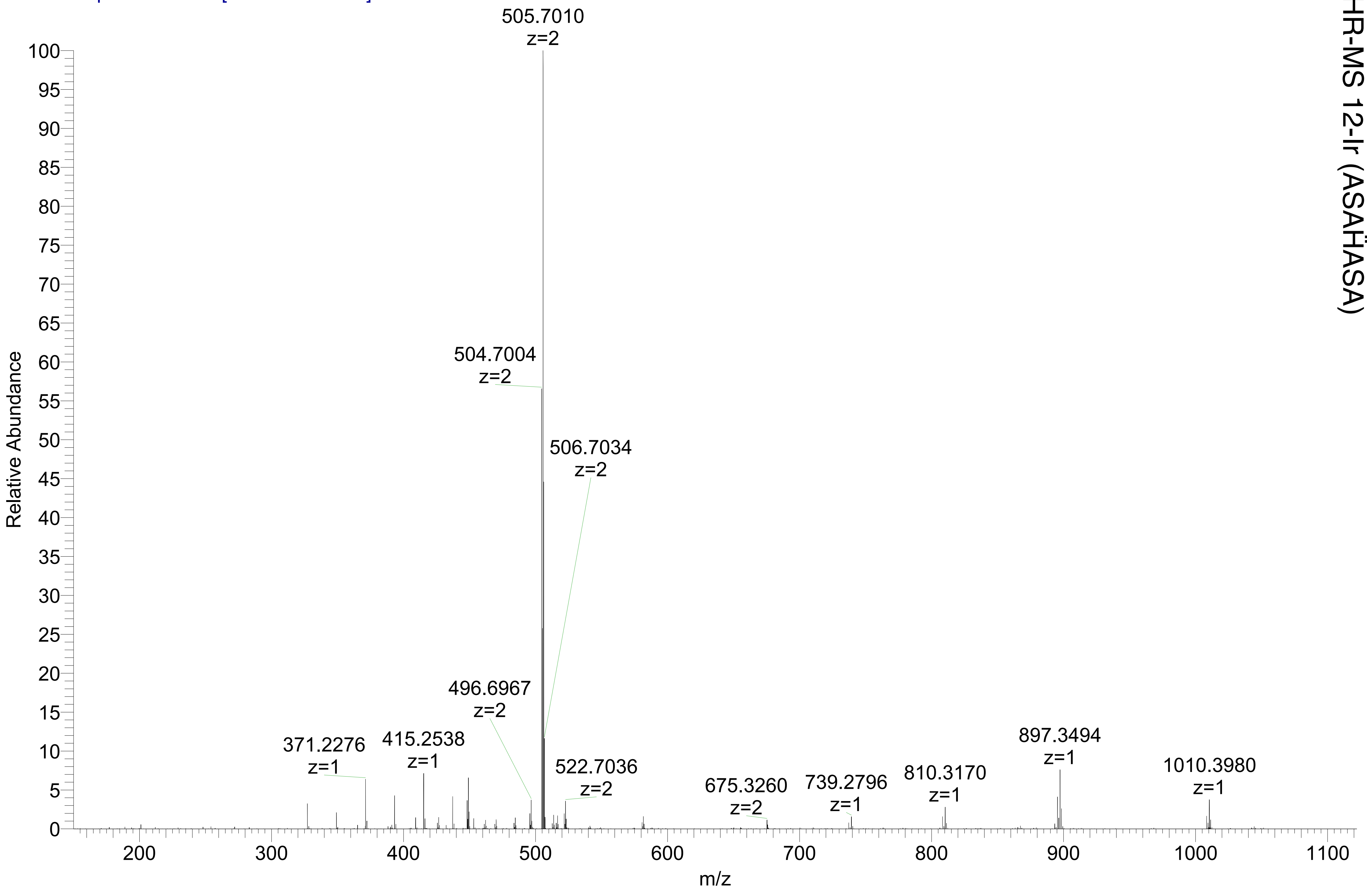

F:\Xcalibur\Data\Zamolo\MATTEO\KL779S-APO.raw 14.11.2018 10:18:01

RT :0.00-10.00 TIC MS KL779S-APO

LC-MS 12-apo (ASAḦASA)

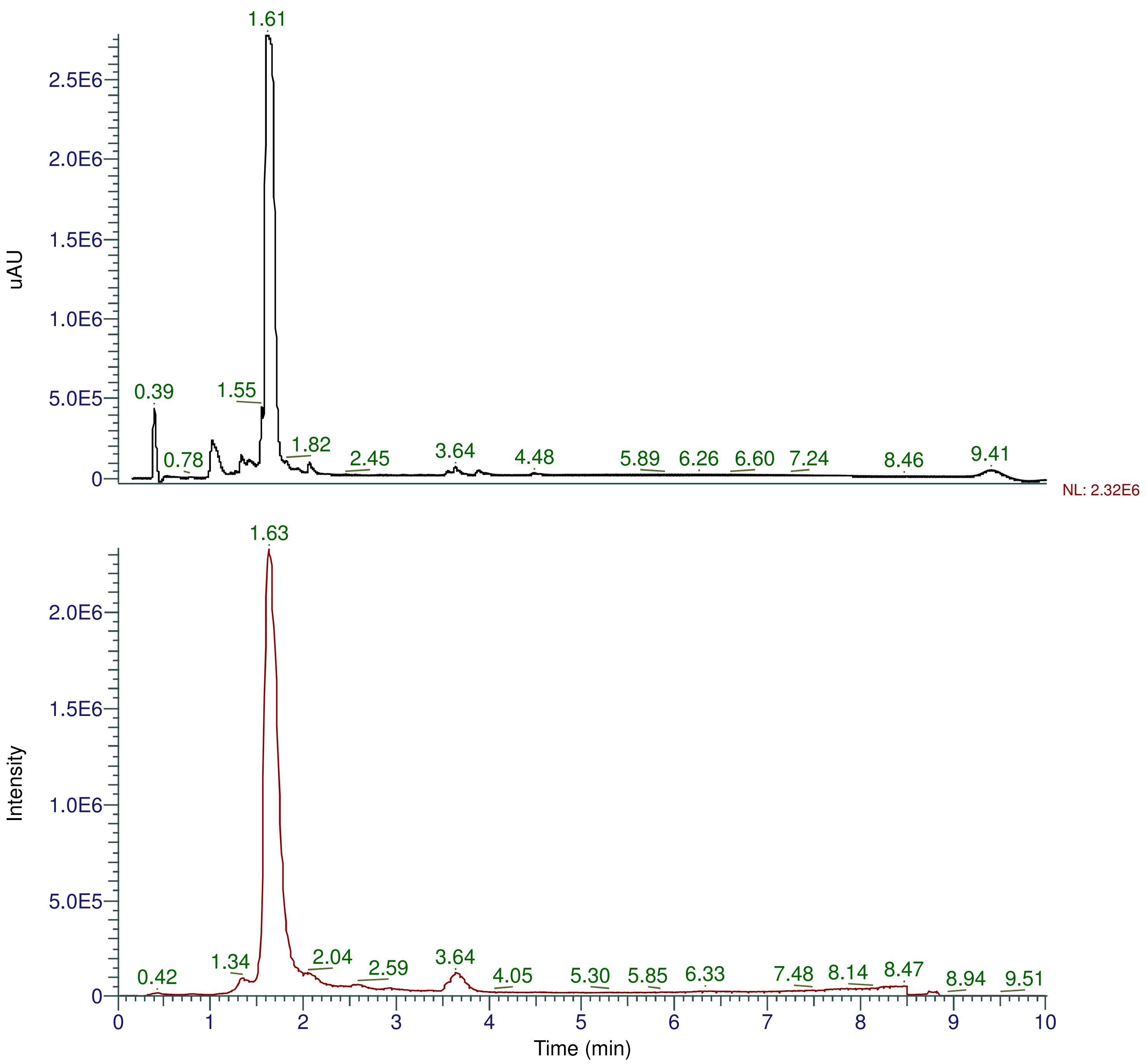

KL779S-APO #100 RT: 1.65 AV: 1 NL: 1.49E+006

T: ITMS + c ESI Full ms [150.00-2000.00]

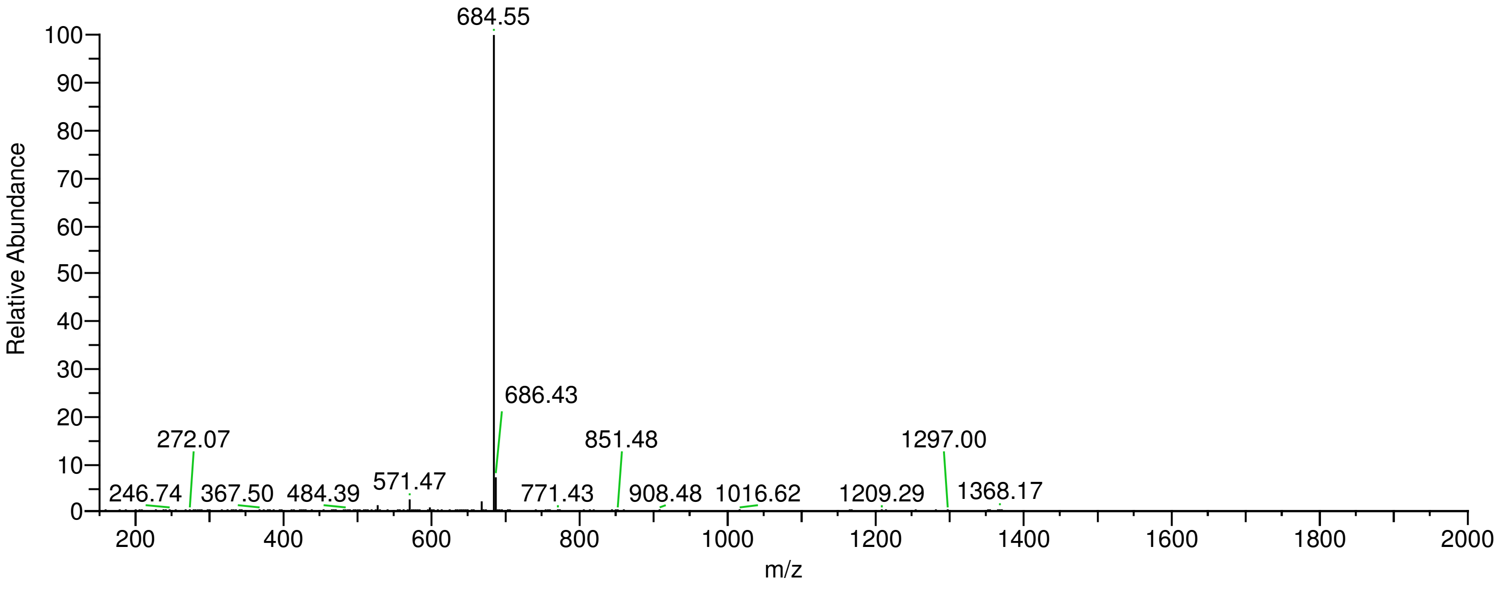

F:\Xcalibur\Data\Meier\KL779S_20180822163809.raw 14.11.2018 10:22:53

RT :0.00-10.00 TIC MS KL779S_20180822163809

LC-MS 12-Ir (ASAḦASA)

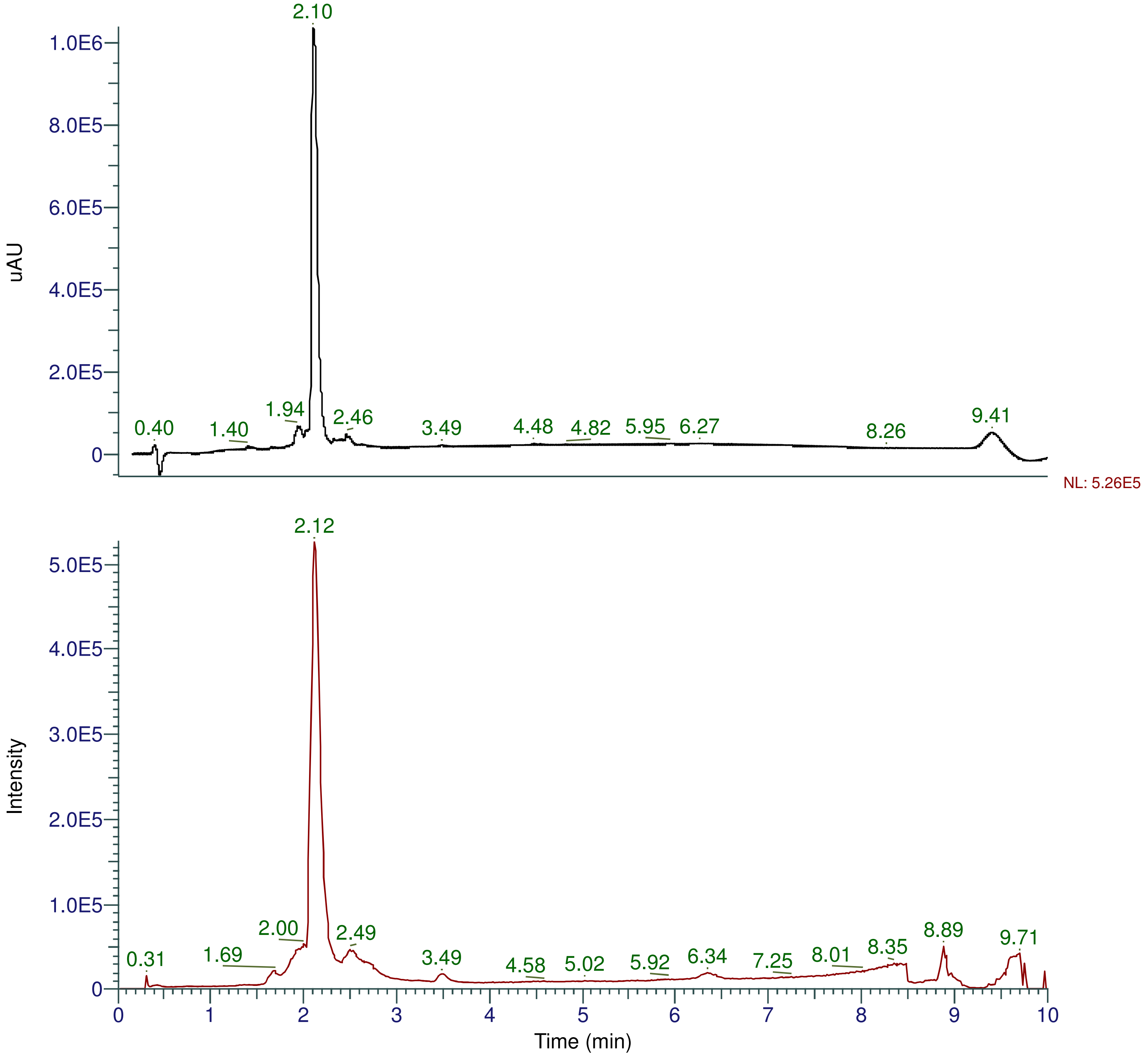

KL779S_20180822163809 #120 RT: 2.12 AV: 1 NL: 1.10E+005
T: ITMS + c ESI Full ms [150.00-2000.00]

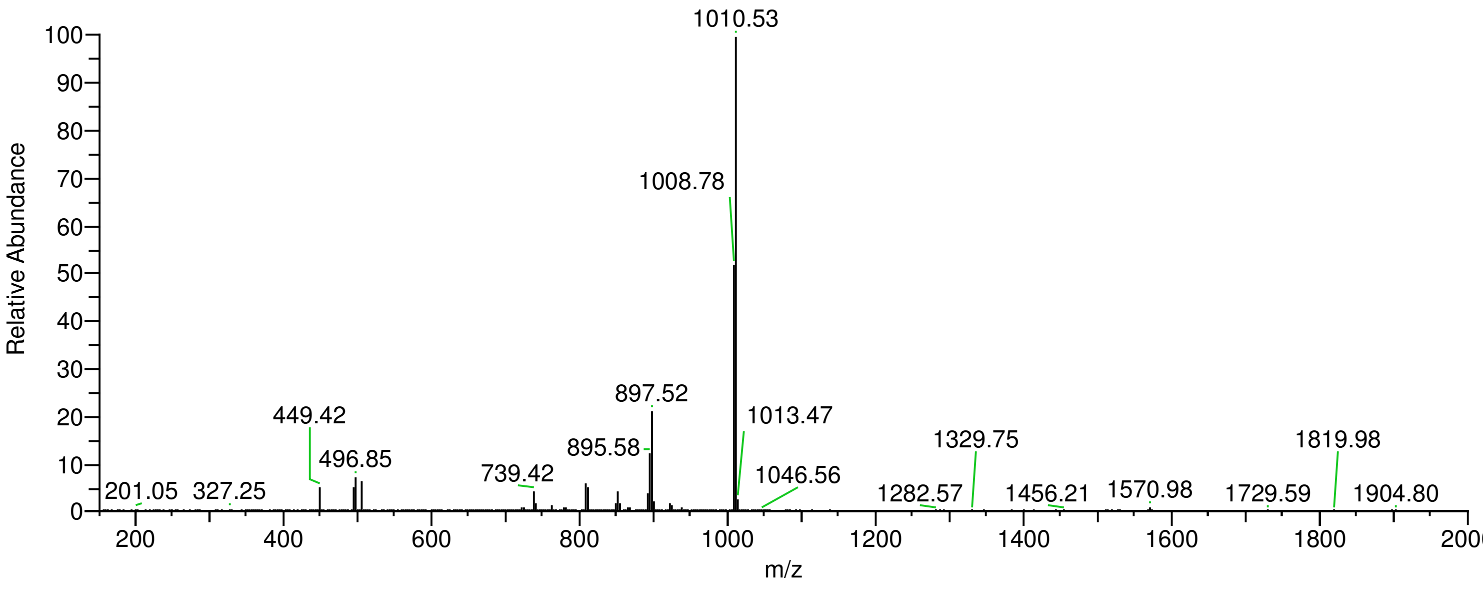

Planchestainer MP30718_7aaHumD_180802... 8/3/2018 11:42:31 AM MP30718_7aaHumD
NSI pos ACN/H2O

Planchestainer MP30718_7aaHumD_180802135018 #1-4 RT: 0.02-0.12 AV: 4 NL: 1.21E6
T: FTMS + p NSI Full ms [100.00-2000.00]

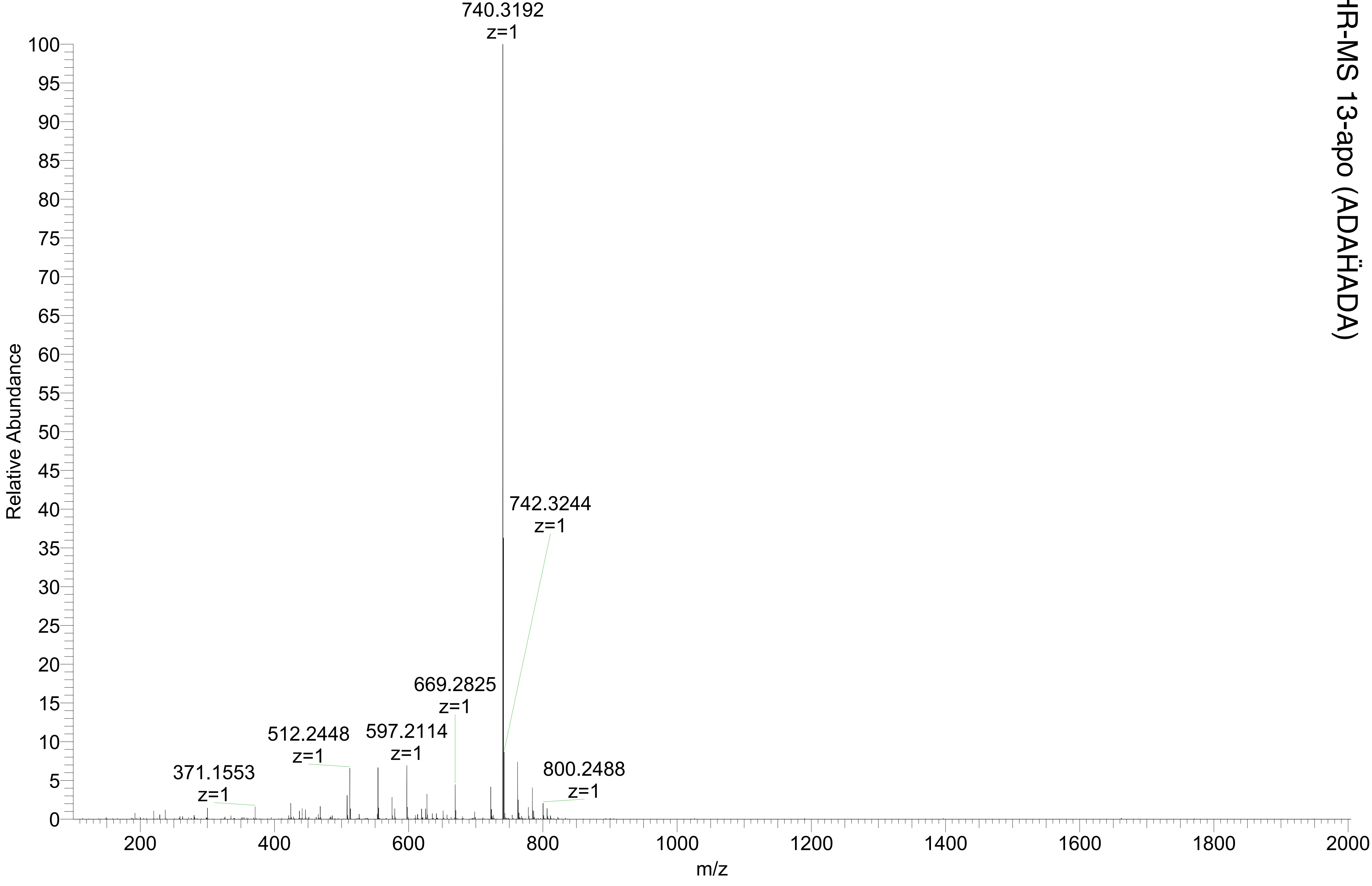

HR-MS 13-apo (ADAḦADA)

HR-MS 13-Ir (ADAḦADA)

Matteo 7aaHumD Ir F1_180808090125 8/8/2018 10:23:00 AM Matteo 7aaHumD Ir F1
NSI pos ACN_H2O

Matteo 7aaHumD Ir F1_180808090125 #1-6 RT: 0.00-0.14 AV: 6 NL: 1.72E8
T: FTMS + p NSI Full ms [150.00-2000.00]

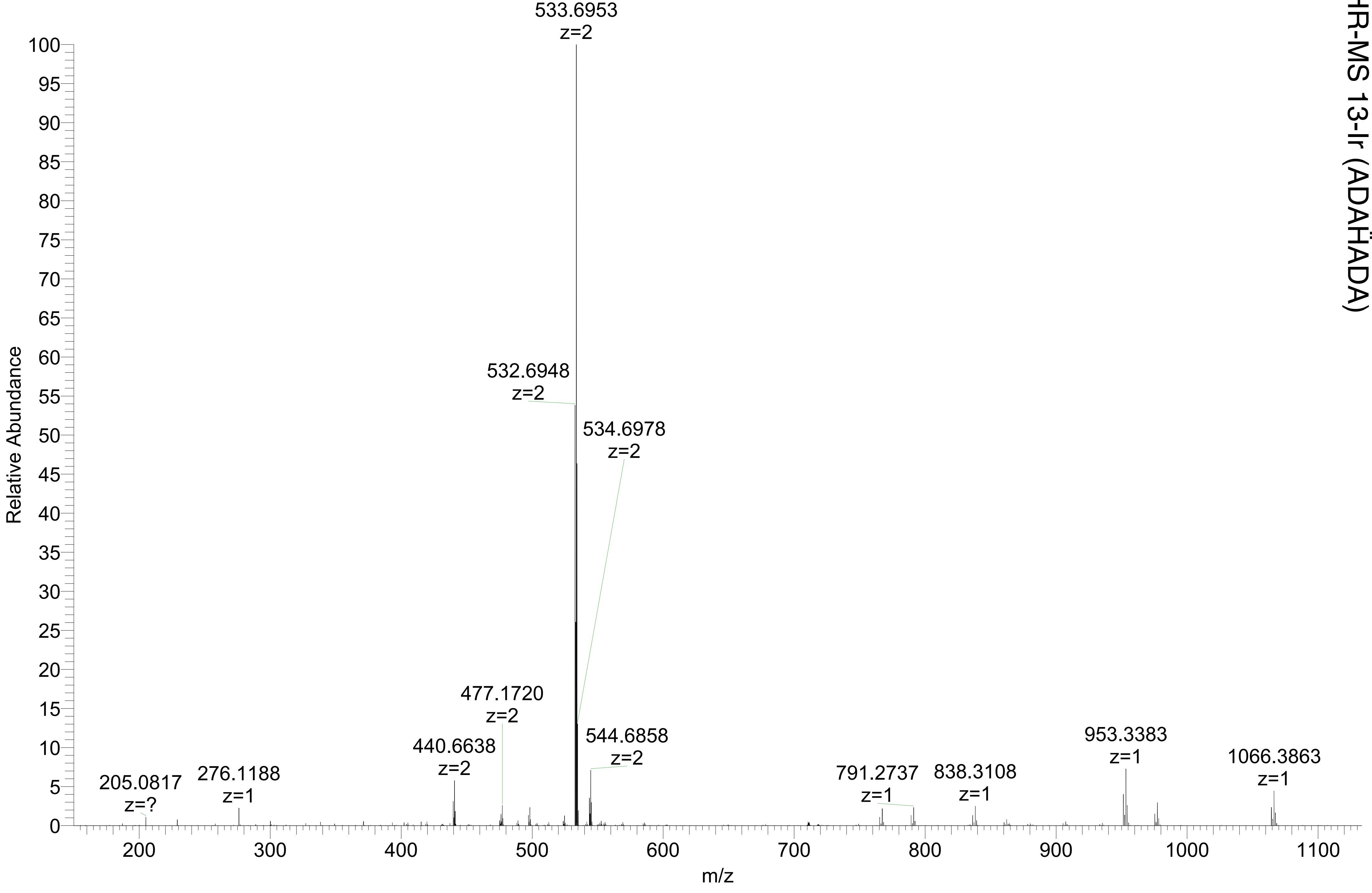

F:\Xcalibur\Data\Zamolo\MATTEO\KL-779-Dapo.raw 14.11.2018 10:11:01

RT :0.00-10.00 TIC MS KL-779-Dapo

LC-MS 13-apo (ADAḦADA)

NL: 2.08E6

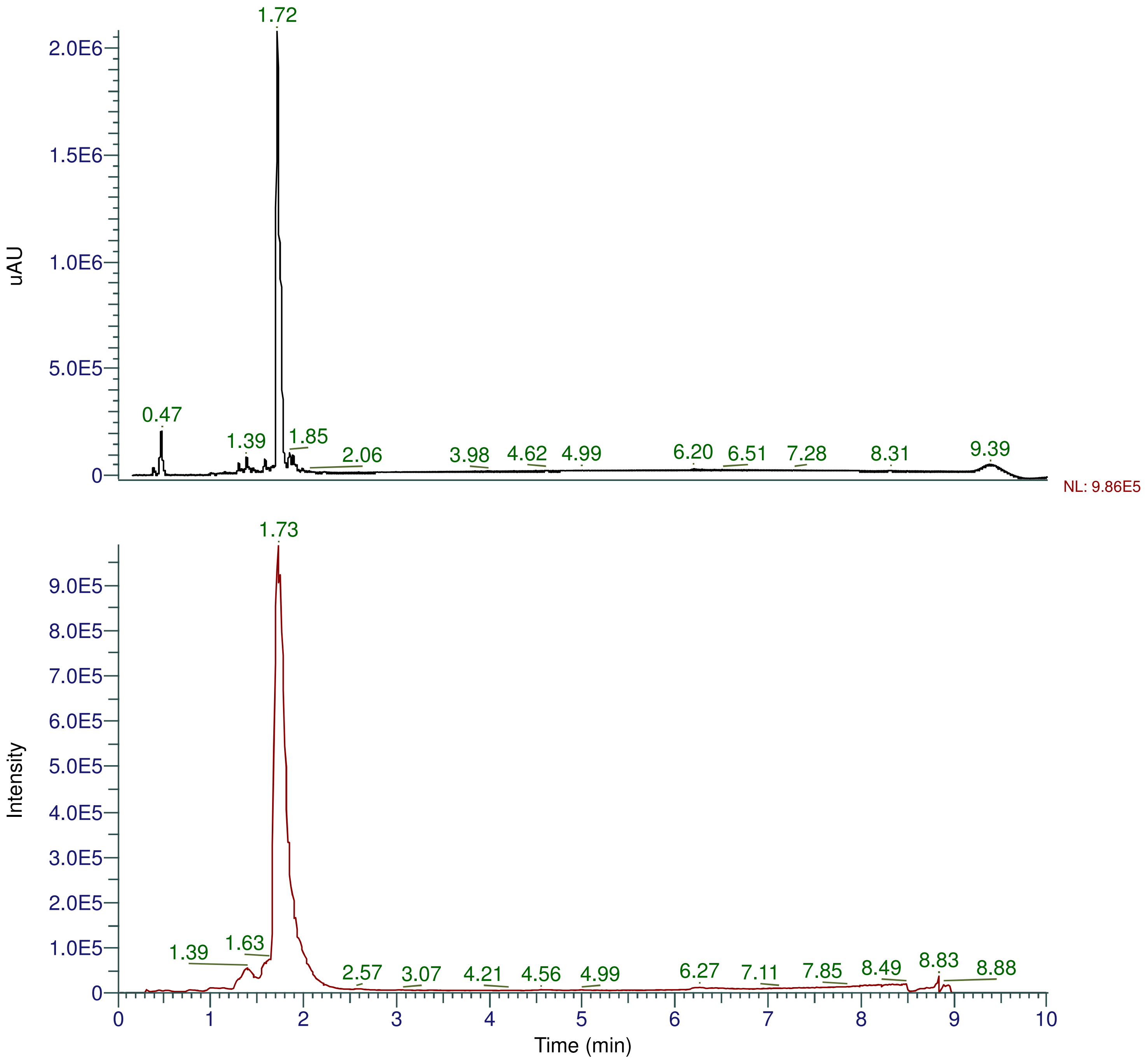

KL-779-Dapo #100 RT: 1.70 AV: 1 NL: 5.14E+005

T: ITMS + c ESI Full ms [150.00-2000.00]

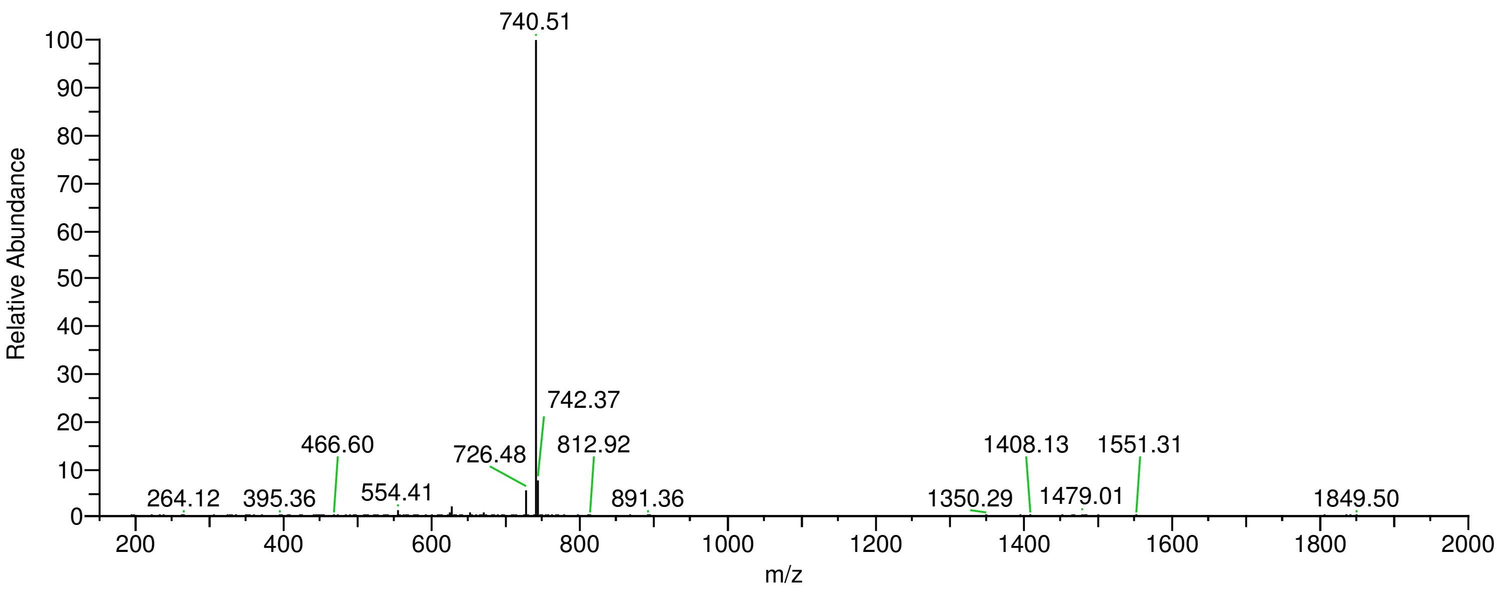

F:\Xcalibur\Data\Meier\KL779D.raw 14.11.2018 10:22:08

RT :0.00-10.00 TIC MS KL779D

LC-MS 13-Ir (ADAḦADA)

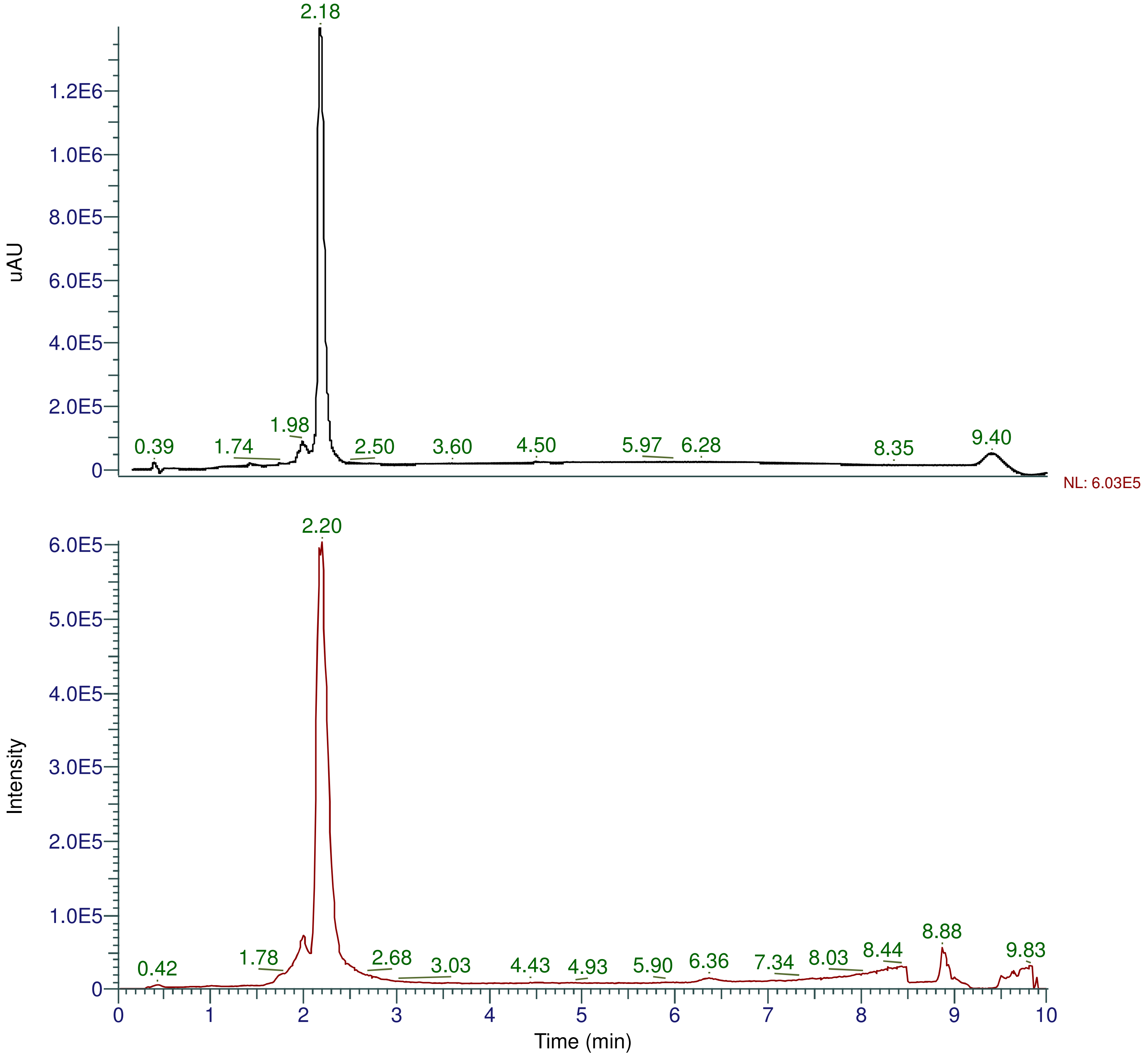

KL779D #126 RT: 2.20 AV: 1 NL: 1.15E+005
T: ITMS + c ESI Full ms [150.00-2000.00]

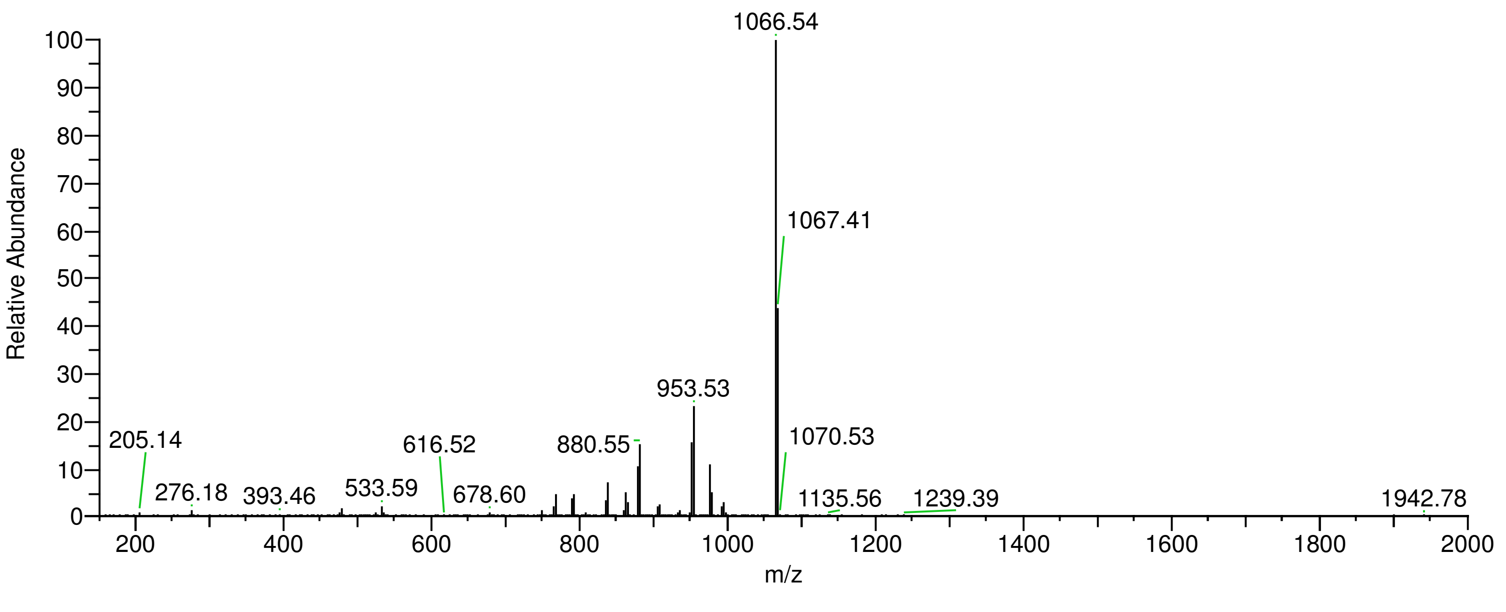

Feller IF 005_7aaE_01_F2_190321141043 3/22/2019 11:10:07 AM Feller IF 005_7aaE_01_F2
NSI pos ACN_H2O

Feller IF 005_7aaE_01_F2_190321141043 #1-6 RT: 0.01-0.15 AV: 6 NL: 6.30E7
T: FTMS + p NSI Full ms [200.00-2000.00]

HR-MS 14-apo (AEAḦAEA)

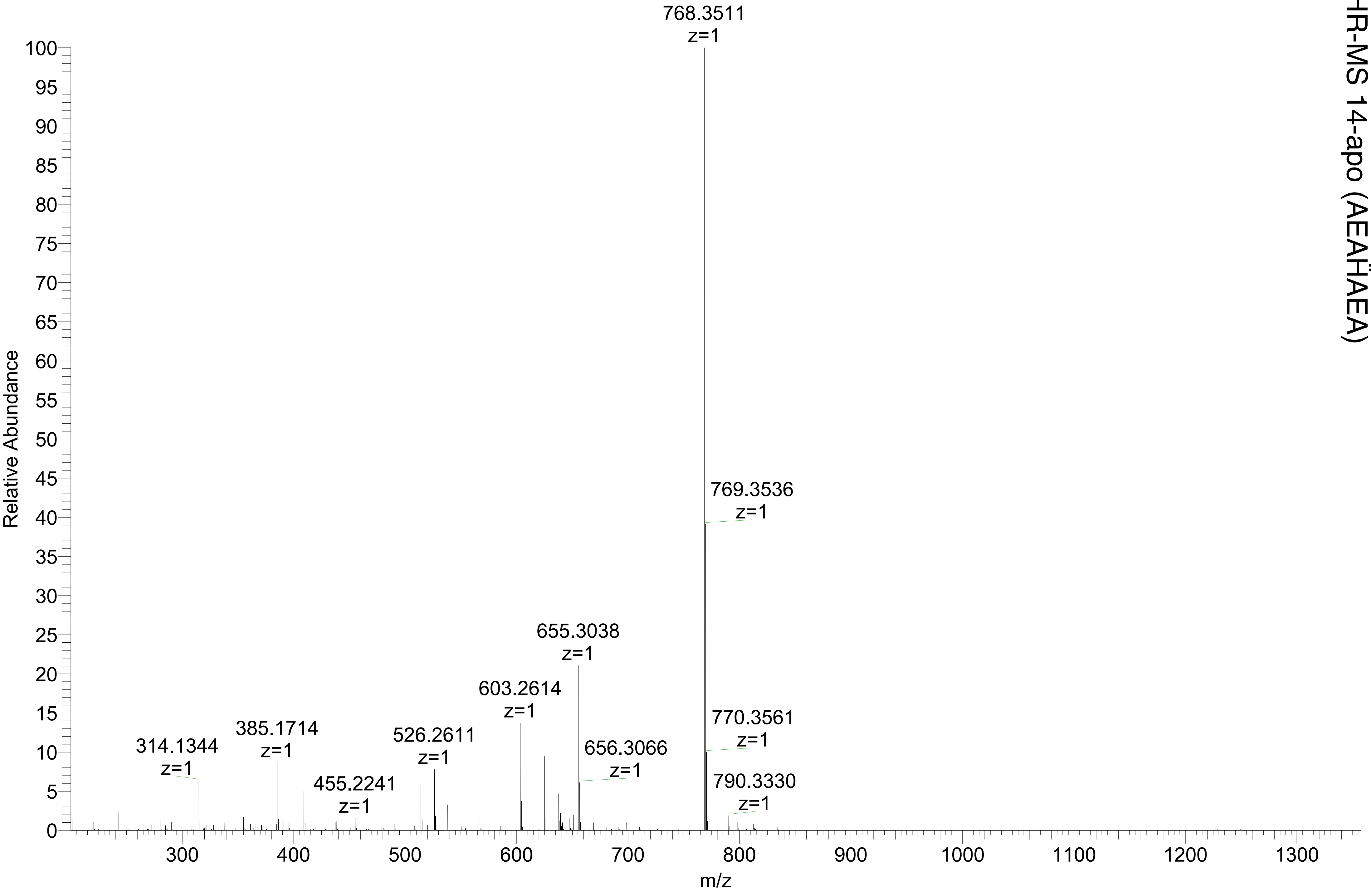

HR-MS 14-Ir (AEAḦAEA)

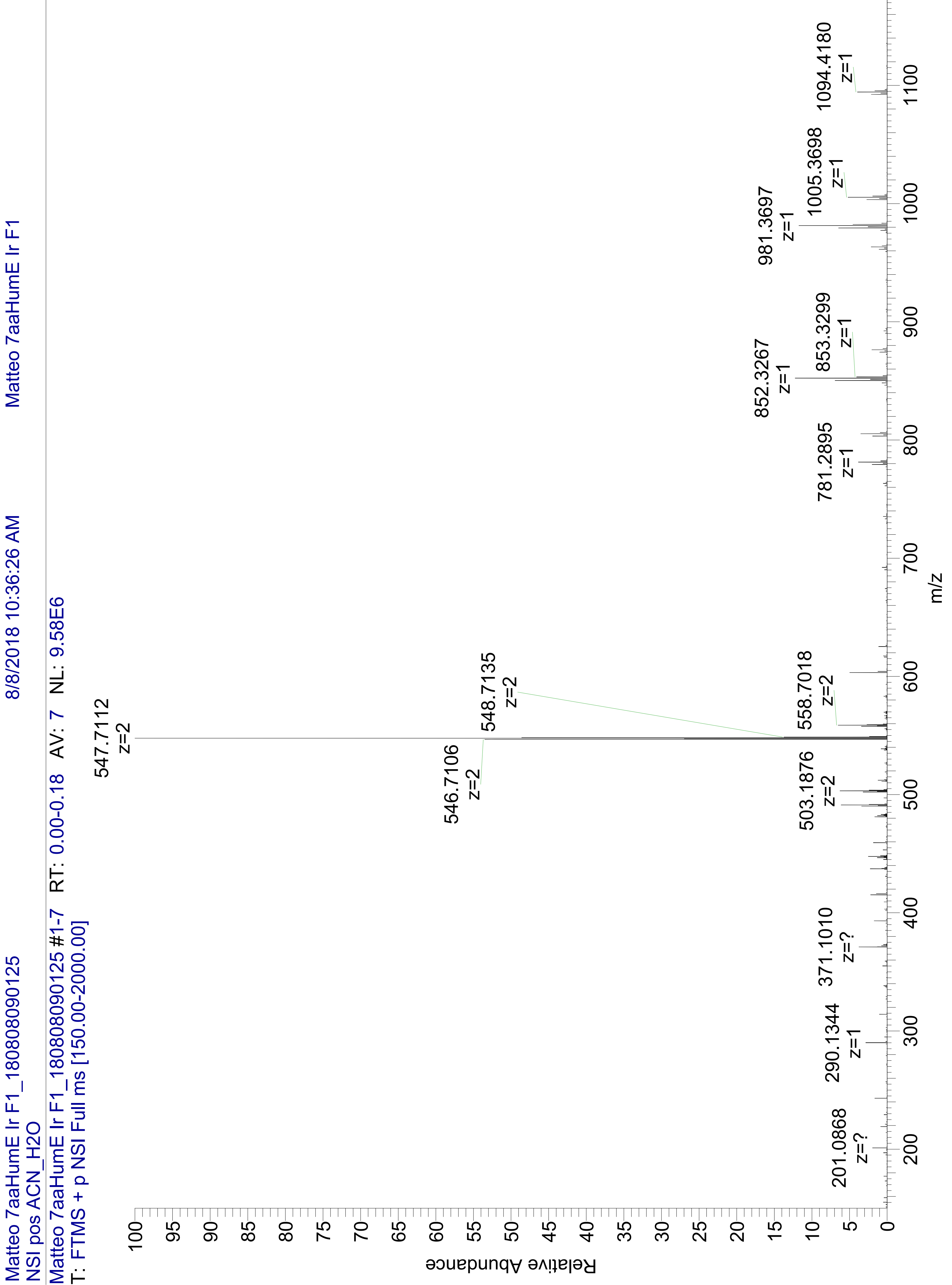

F:\Xcalibur\Data\Zamolo\MATTEO\KL-779-Eapo.raw 14.11.2018 10:15:17

RT :0.00-10.00 TIC MS KL-779-Eapo

LC-MS 14-apo (AEAḦAEA)

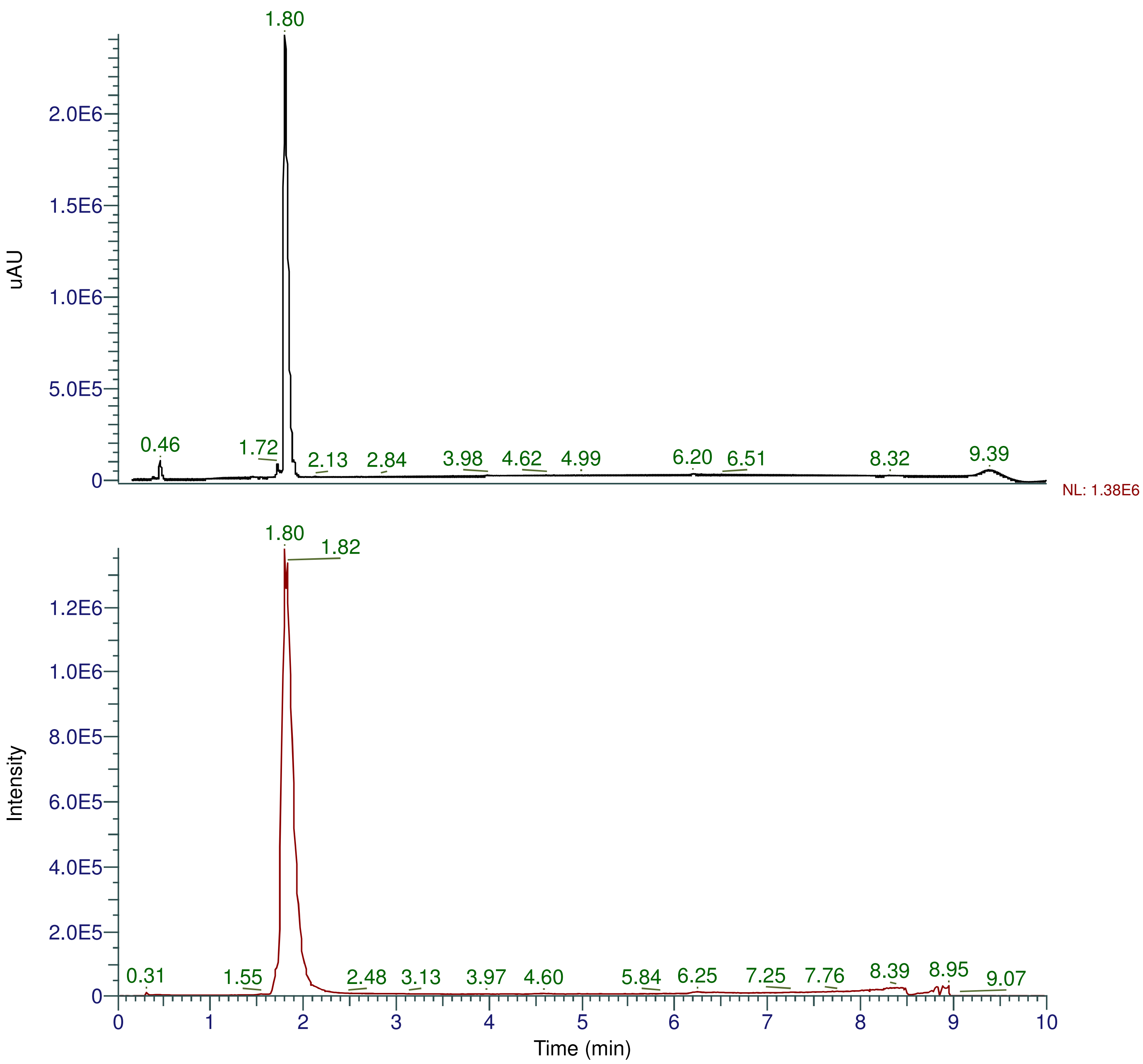

KL-779-Eapo #100 RT: 1.81 AV: 1 NL: 7.49E+005

T: ITMS + c ESI Full ms [150.00-2000.00]

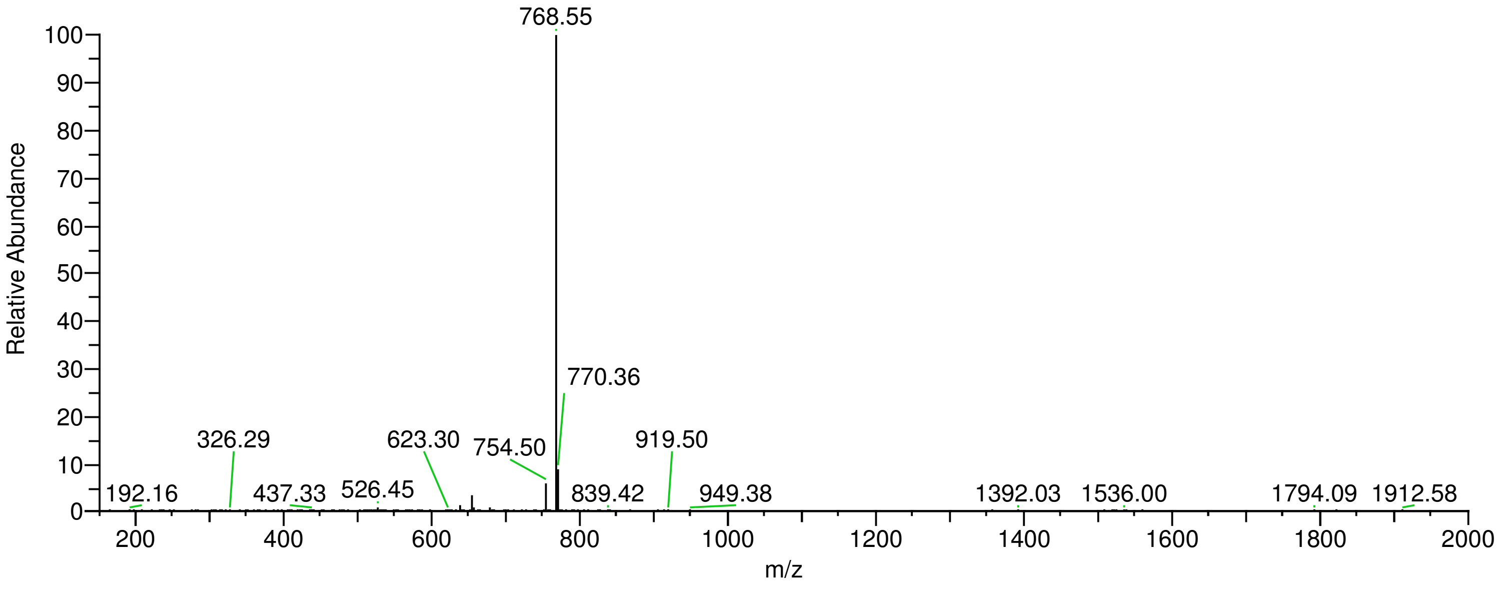

F:\Xcalibur\Data\Meier\KL779E_20180822171215.raw 14.11.2018 10:21:43

RT :0.00-10.00 TIC MS KL779E_20180822171215

LC-MS 14-Ir (AEAḦAEA)

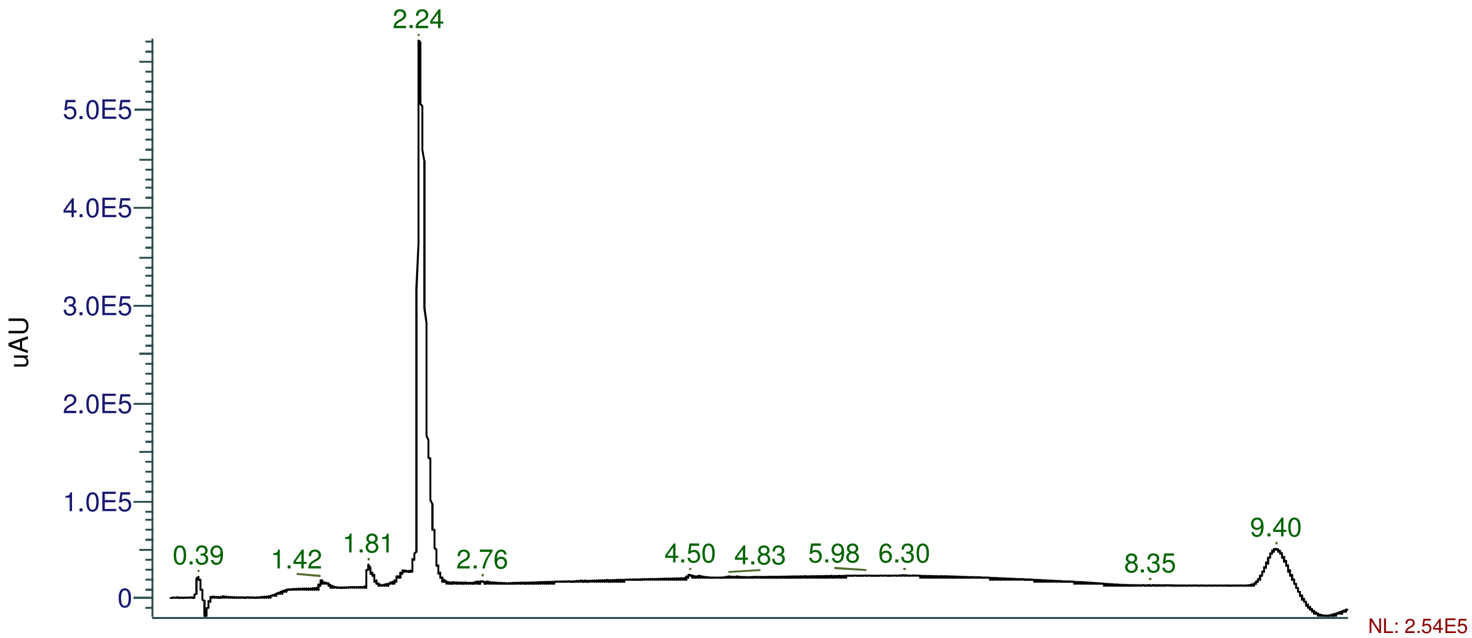

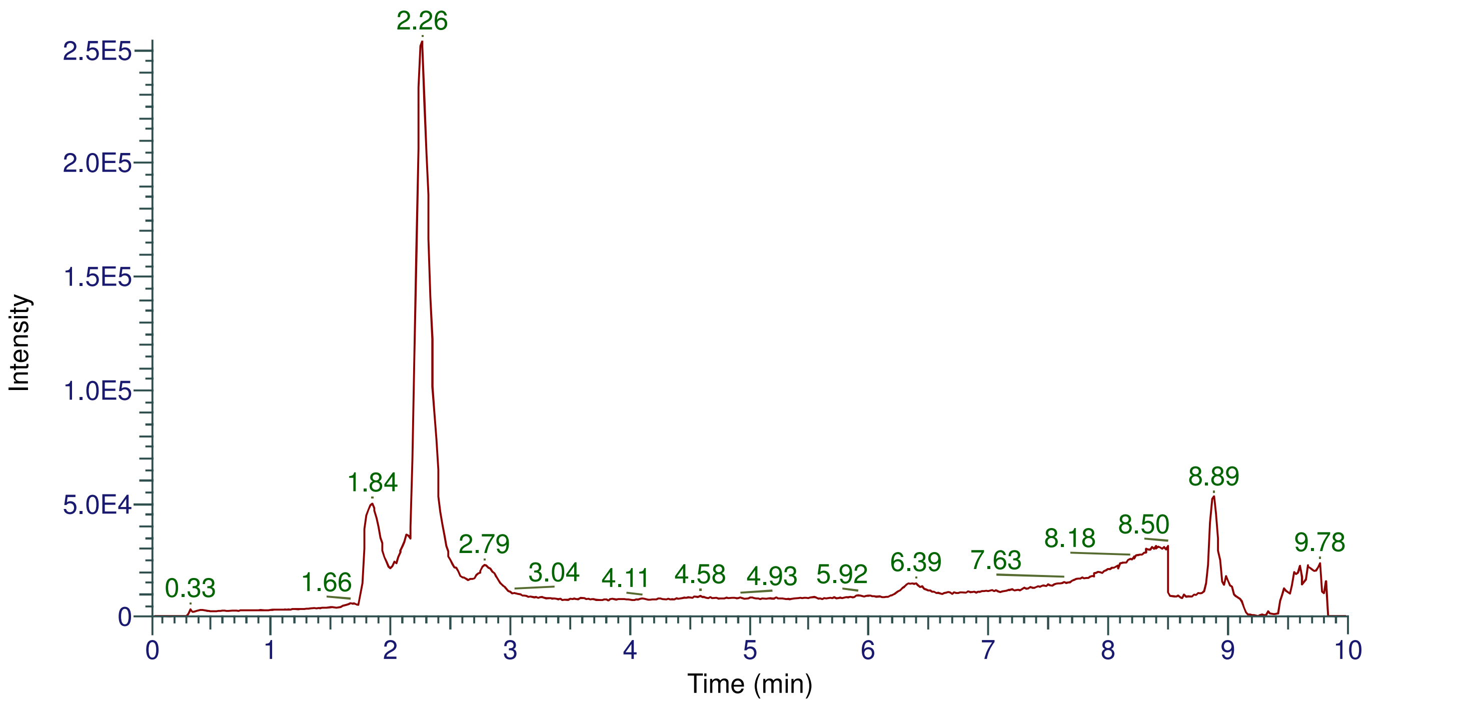

KL779E_20180822171215 #129 RT: 2.26 AV: 1 NL: 3.62E+004
T: ITMS + c ESI Full ms [150.00-2000.00]

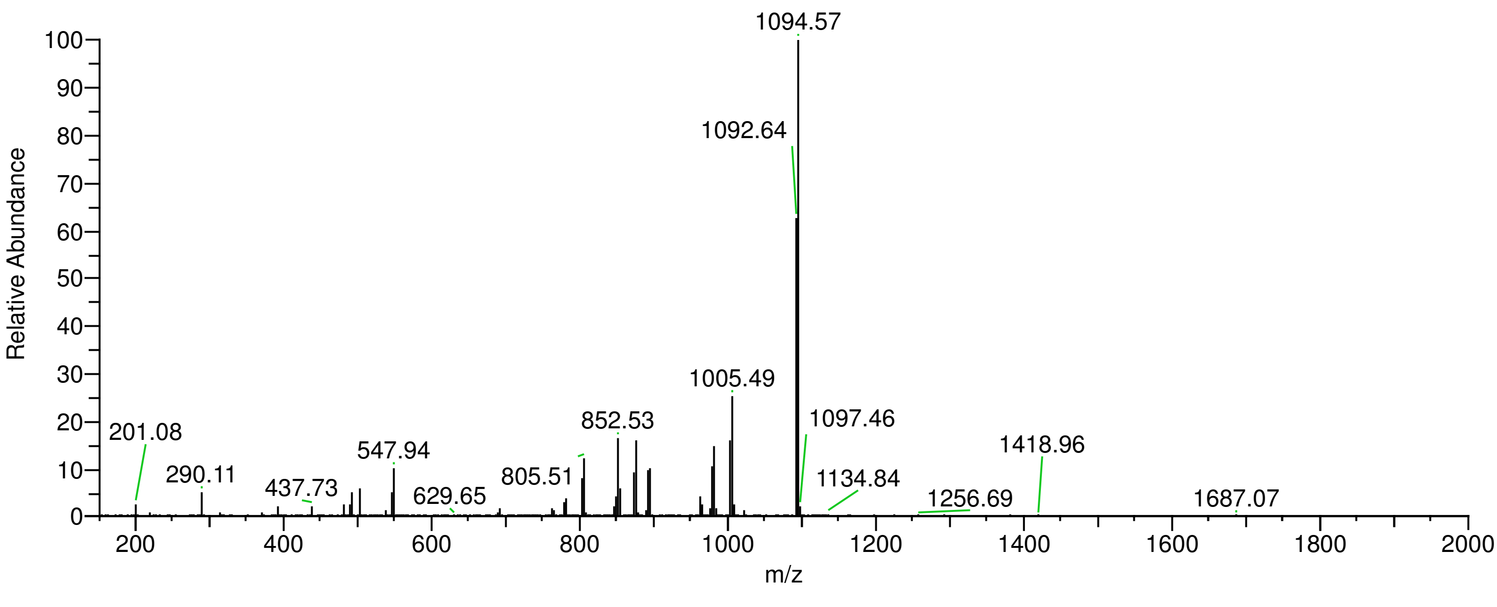

HR-MS 15-apo (AKAḦAKA)

Matteo MP 7aaK_apo_pure_190111124157 1/11/2019 1:18:19 PM Matteo MP 7aaK_apo_pure
NSI pos ACN_H2O

Matteo MP 7aaK_apo_pure_190111124157 #1-9 RT: 0.01-0.22 AV: 9 NL: 5.89E8
T: FTMS + p NSI Full ms [150.00-2000.00]

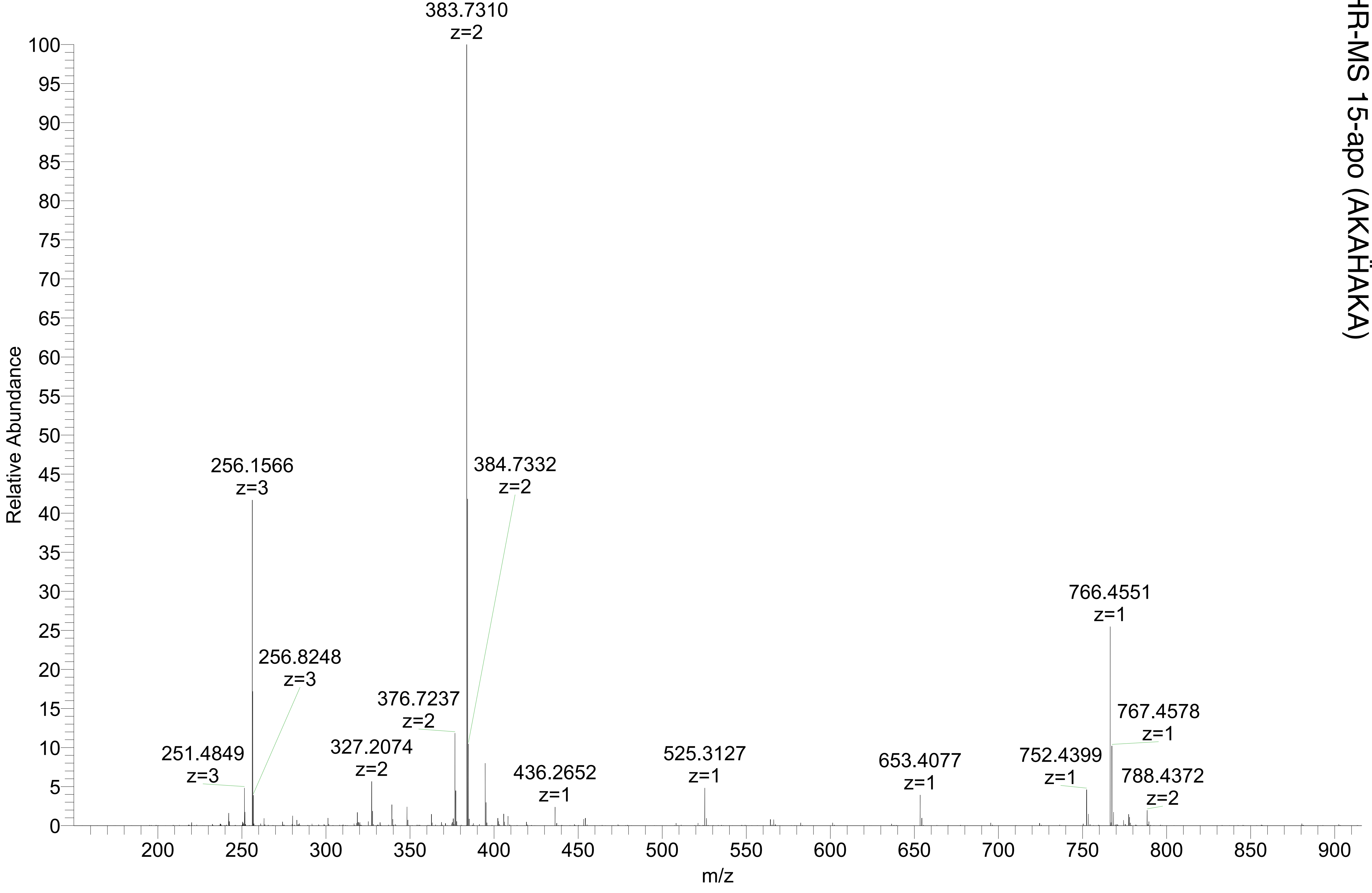

HR-MS 15-Ir (AKAḦAKA)

Matteo 7aaHumK Ir F1_180808133151 8/9/2018 11:10:43 AM Matteo 7aaHumK Ir F1
NSI pos ACN_H2O

Matteo 7aaHumK Ir F1_180808133151 #1-4 RT: 0.02-0.10 AV: 4 NL: 3.27E7
T: FTMS + p NSI Full ms [150.00-2000.00]

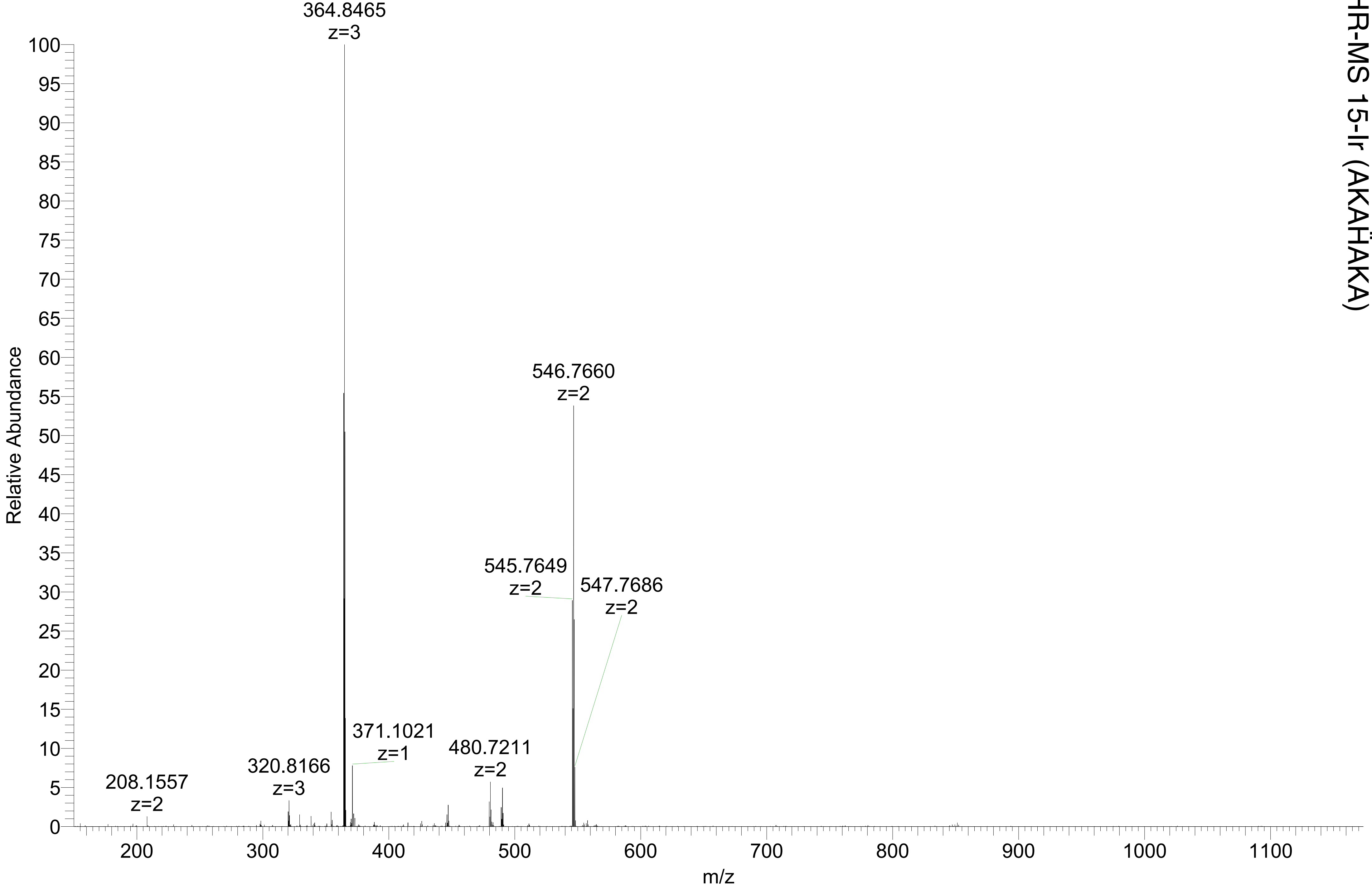

F:\Xcalibur\Data\Zamolo\MATTEO\KL-779-Kapo.raw 14.11.2018 10:19:01

RT :0.00-10.00 TIC MS KL-779-Kapo

LC-MS 15-apo (AKAḦAKA)

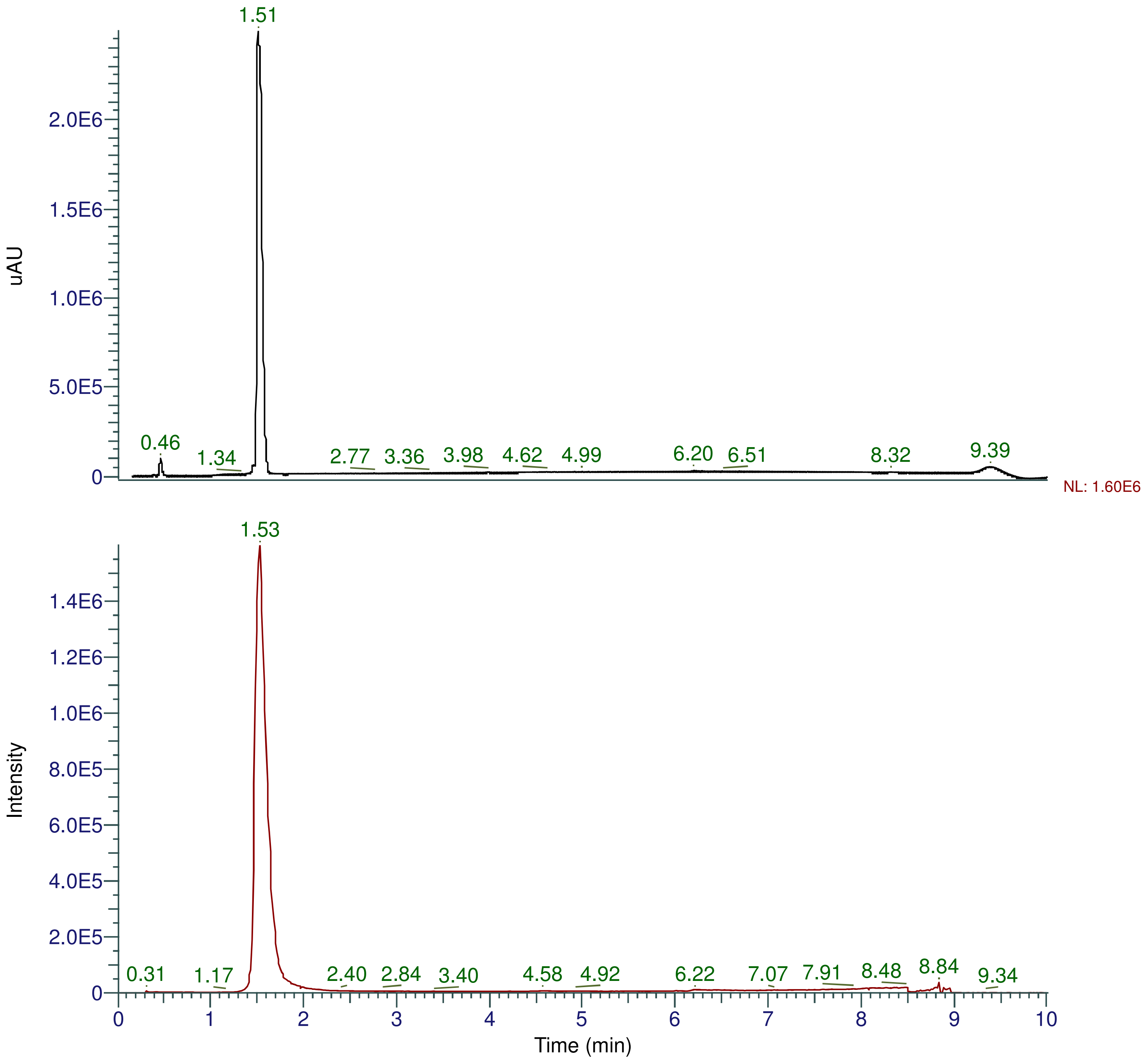

KL-779-Kapo #85 RT: 1.53 AV: 1 NL: 8.28E+005

T: ITMS + c ESI Full ms [150.00-2000.00]

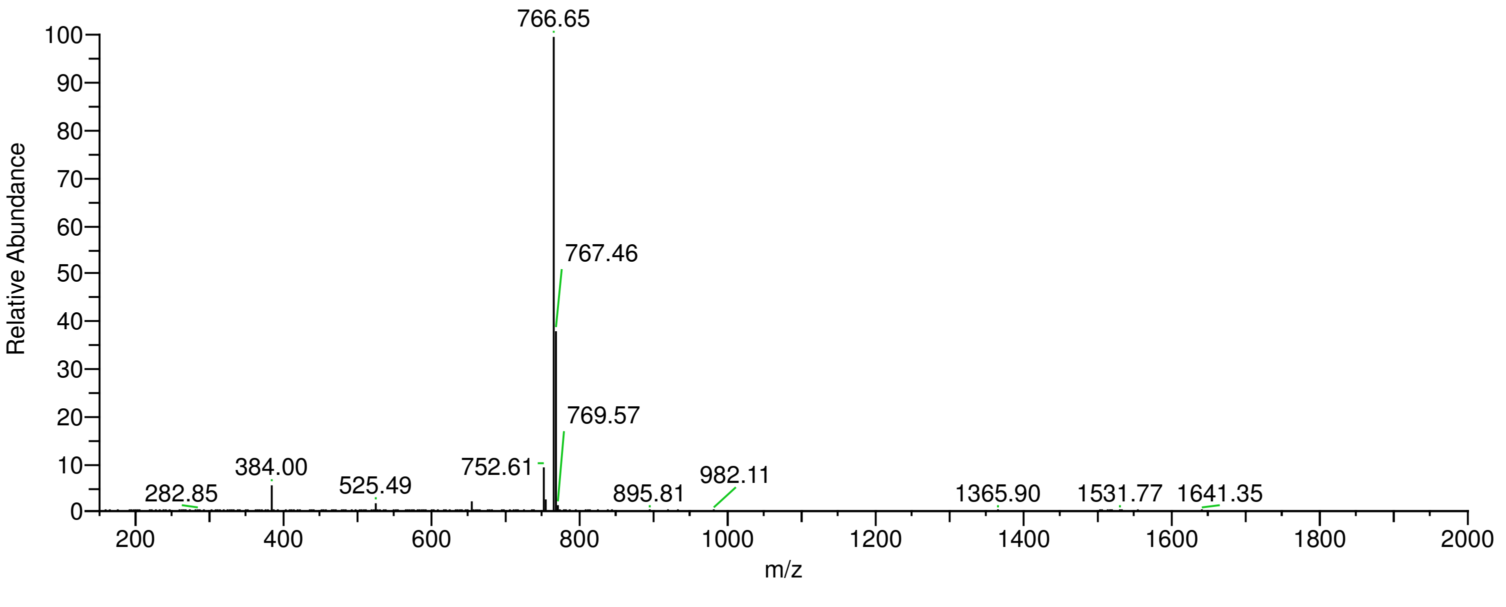

F:\Xcalibur\Data\Meier\KL779K_20180822164931.raw 14.11.2018 10:22:34

RT :0.00-10.00 TIC MS KL779K_20180822164931

LC-MS 15-Ir (AKAḦAKA)

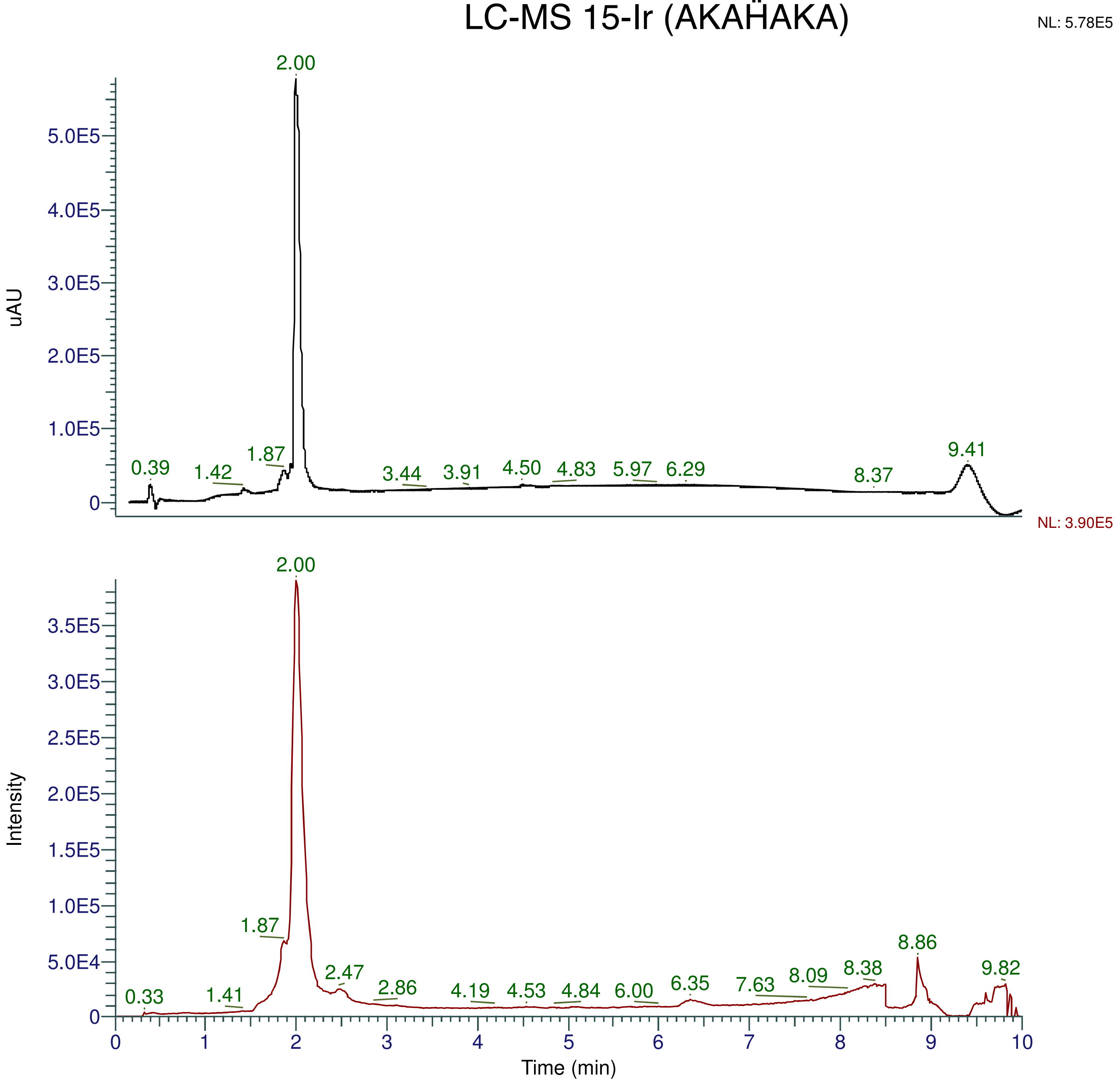

KL779K_20180822164931 #114 RT: 2.01 AV: 1 NL: 5.74E+004

T: ITMS + c ESI Full ms [150.00-2000.00]

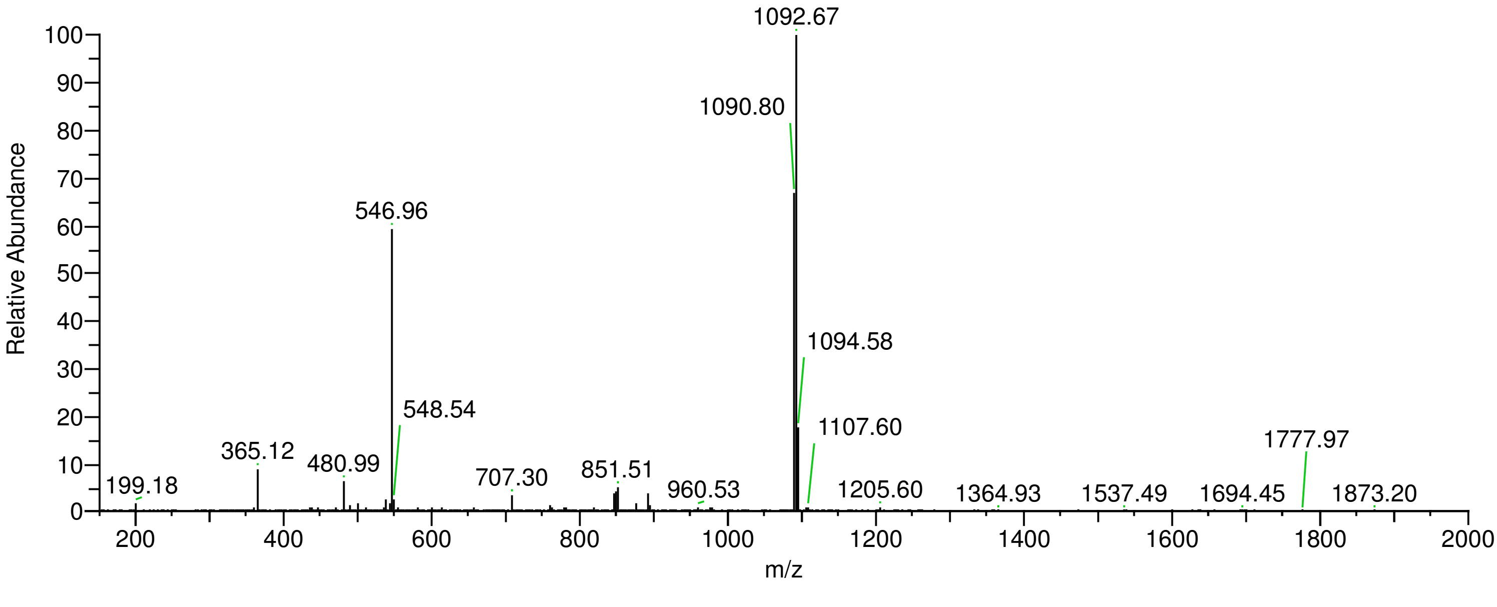

HR-MS 16-apo (AHAẌAHA)

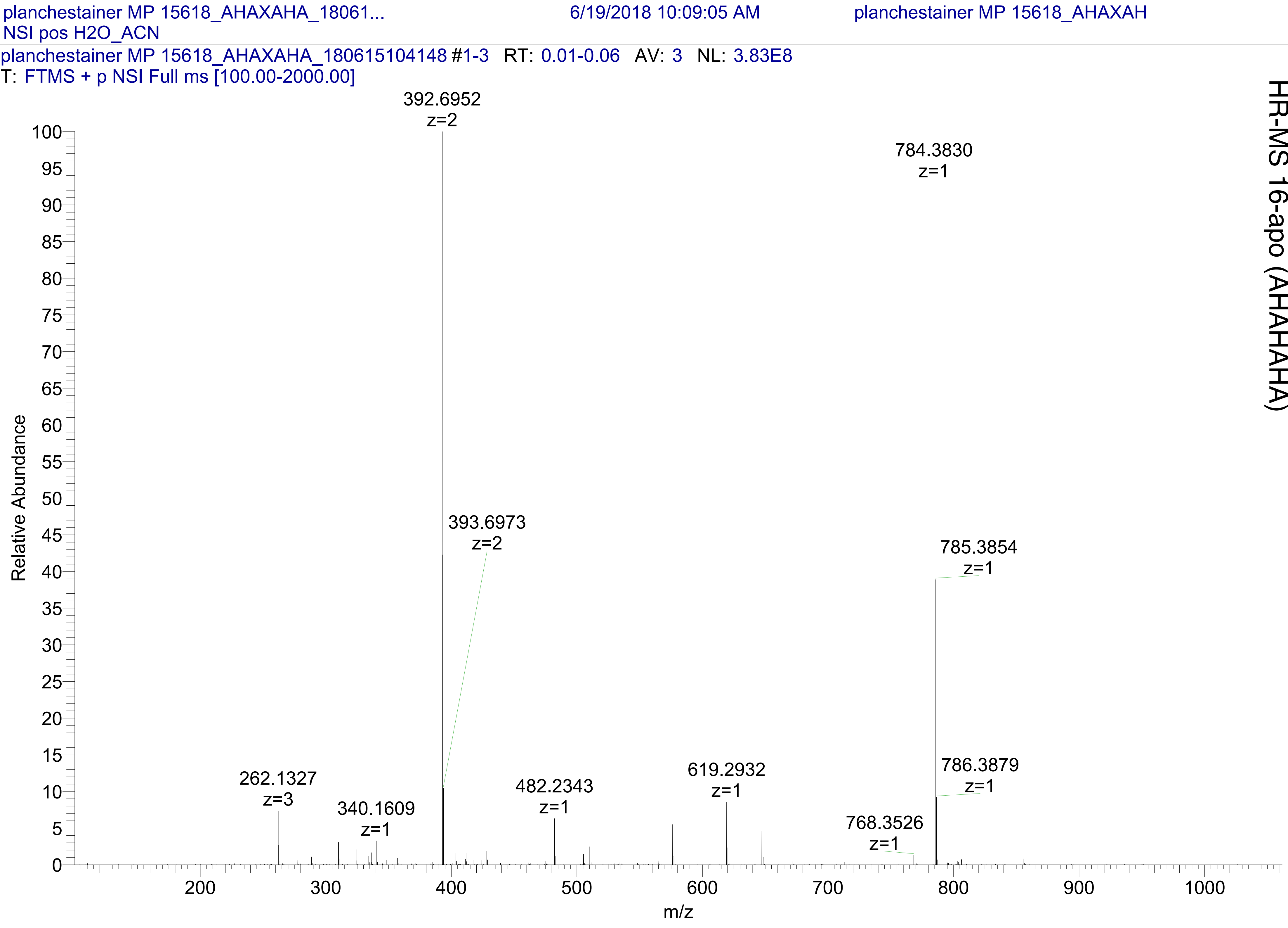

HR-MS 16-Ir (AHAḦAHA)

Matteo 7aaHumH Ir F2_180808133151 8/9/2018 11:14:28 AM Matteo 7aaHumH Ir F2
NSI pos ACN_H2O

Matteo 7aaHumH Ir F2_180808133151 #1-3 RT: 0.01-0.07 AV: 3 NL: 6.22E6
T: FTMS + p NSI Full ms [150.00-2000.00]

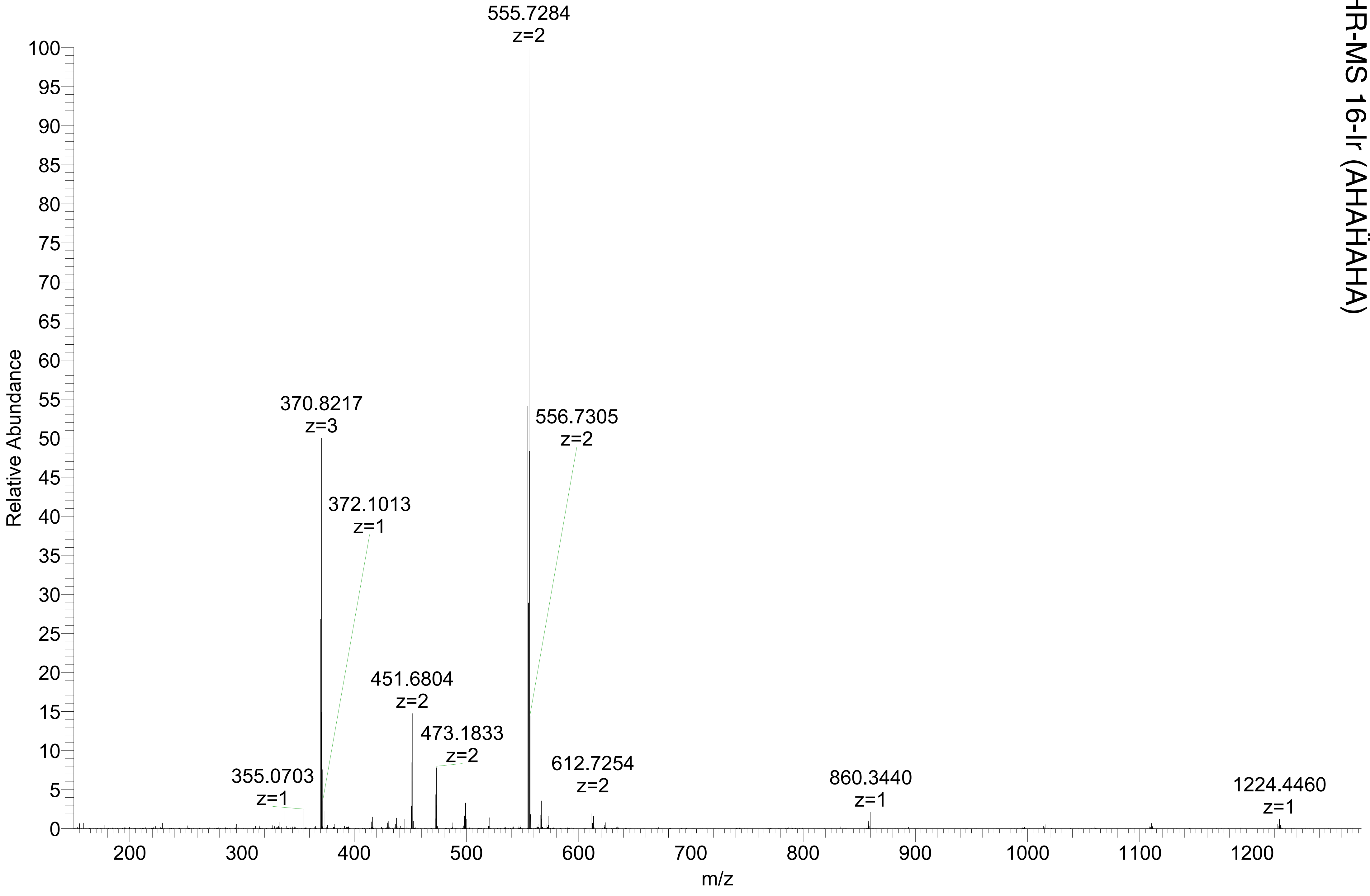

F:\Xcalibur\Data\Zamolo\MATTEO\MP_7aaH-apo2.raw 25.04.2019 17:41:42

RT :0.00-10.00 GNL: 2.73E6 TIC MS MP_7aaH-apo2

LC-MS 16-apo (AHAḦAHA)

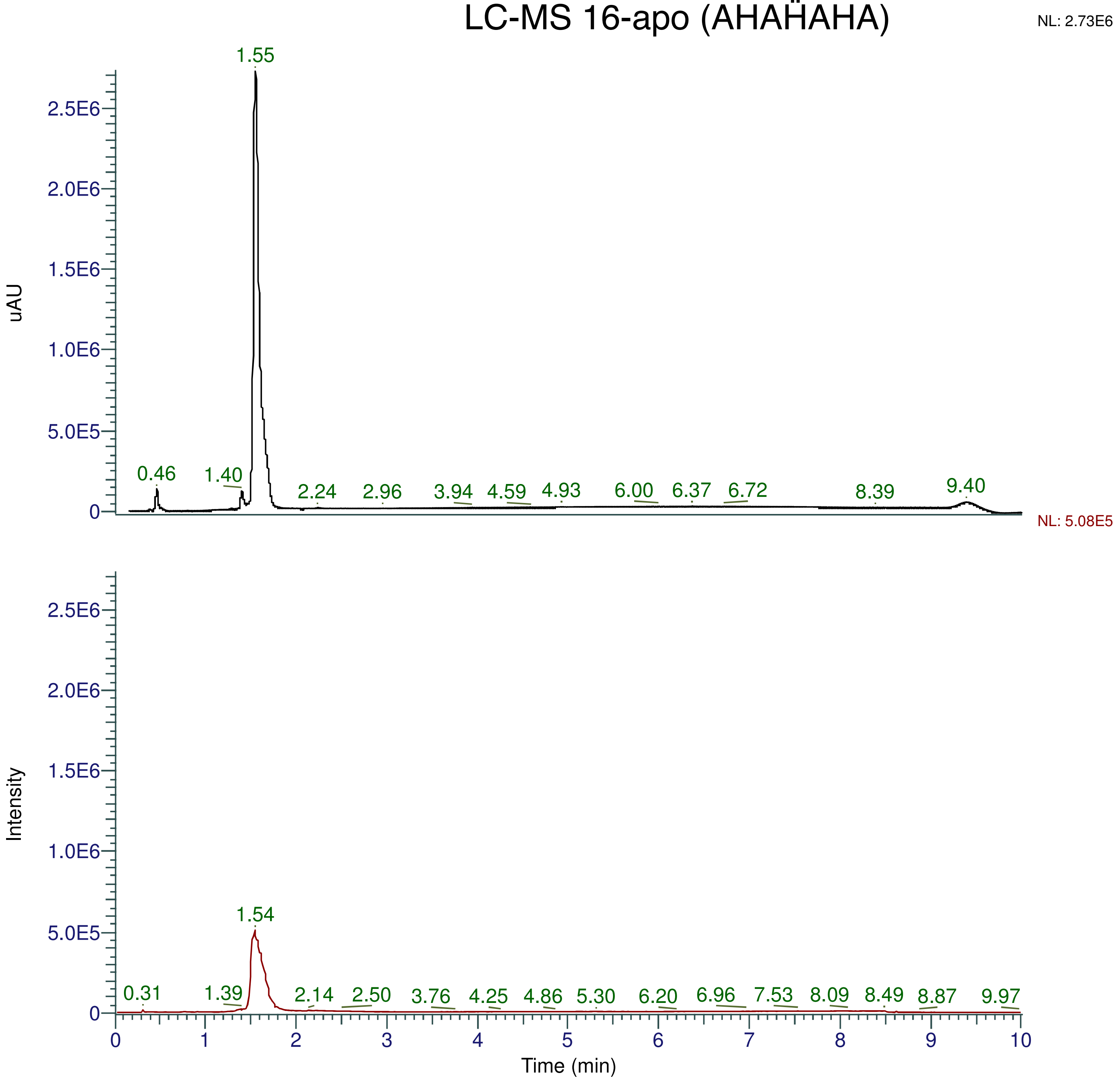

MP_7aaH-apo2 #87 RT: 1.56 AV: 1 NL: 1.90E+005
T: ITMS + c ESI Full ms [150.00-2000.00]

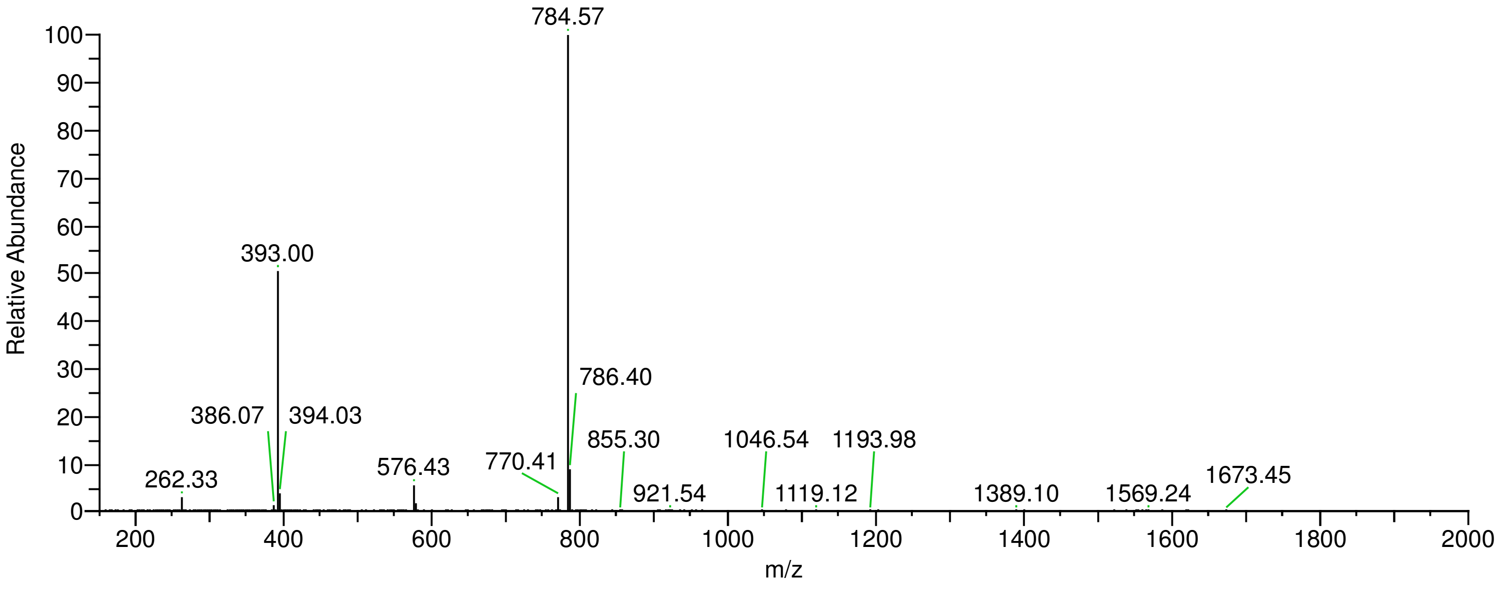

F:\Xcalibur\Data\Meier\KL779H_20180822172338.raw 14.11.2018 10:21:04

RT :0.00-10.00 TIC MS KL779H_20180822172338

LC-MS 16-Ir (AHAḦAHA)

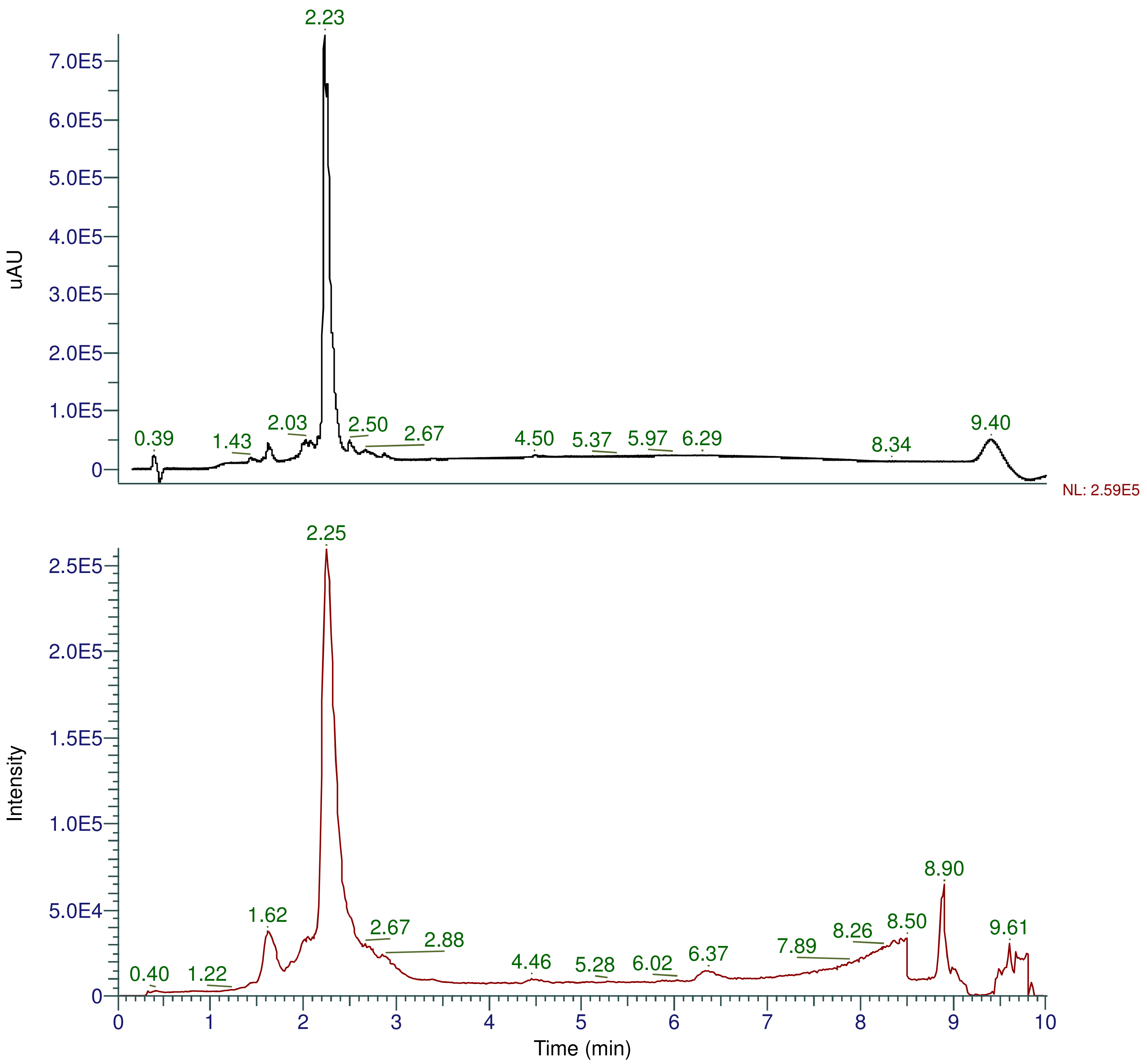

KL779H_20180822172338 #131 RT: 2.26 AV: 1 NL: 5.81E+004
T: ITMS + c ESI Full ms [150.00-2000.00]

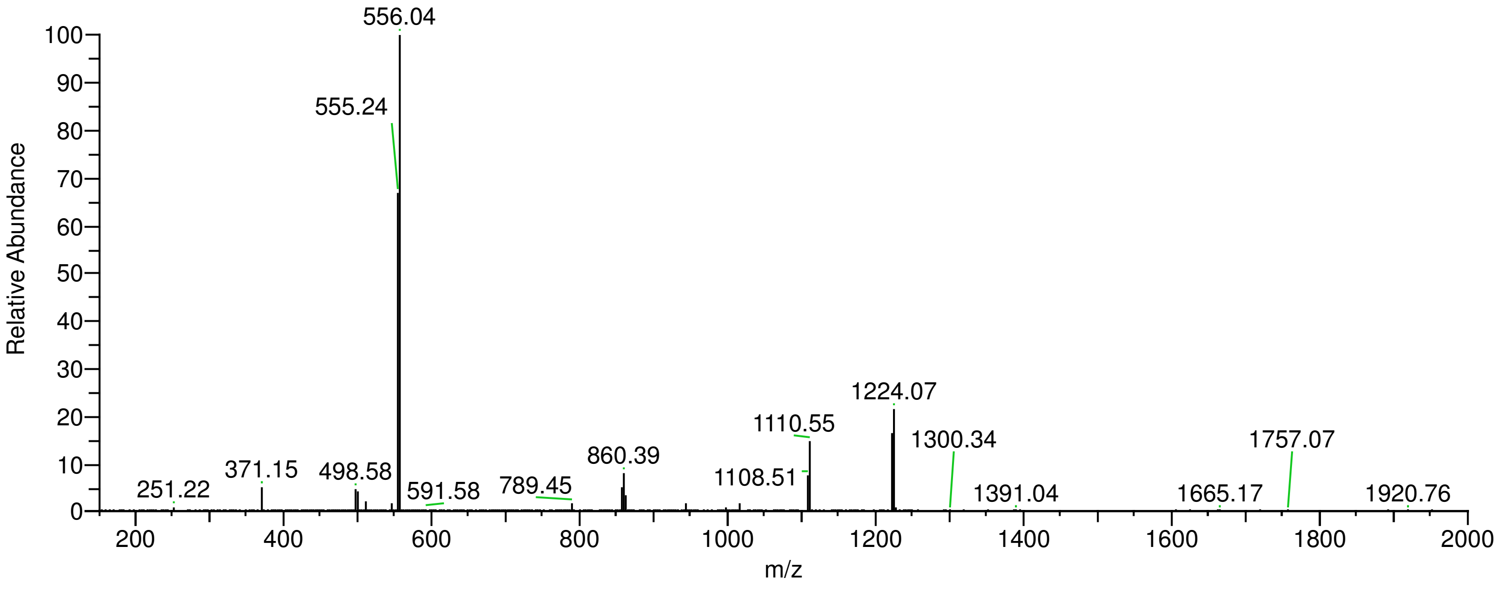

D:\Xcalibur\...\Matteo MP7aaY_apo2 4/29/2019 9:07:38 AM Matteo MP7aaY_apo2
NSI pos ACN_H2O

Matteo MP7aaY_apo2 #1-3 RT: 0.02-0.08 AV: 3 NL: 3.12E7
T: FTMS + p NSI Full ms [150.00-2000.00]

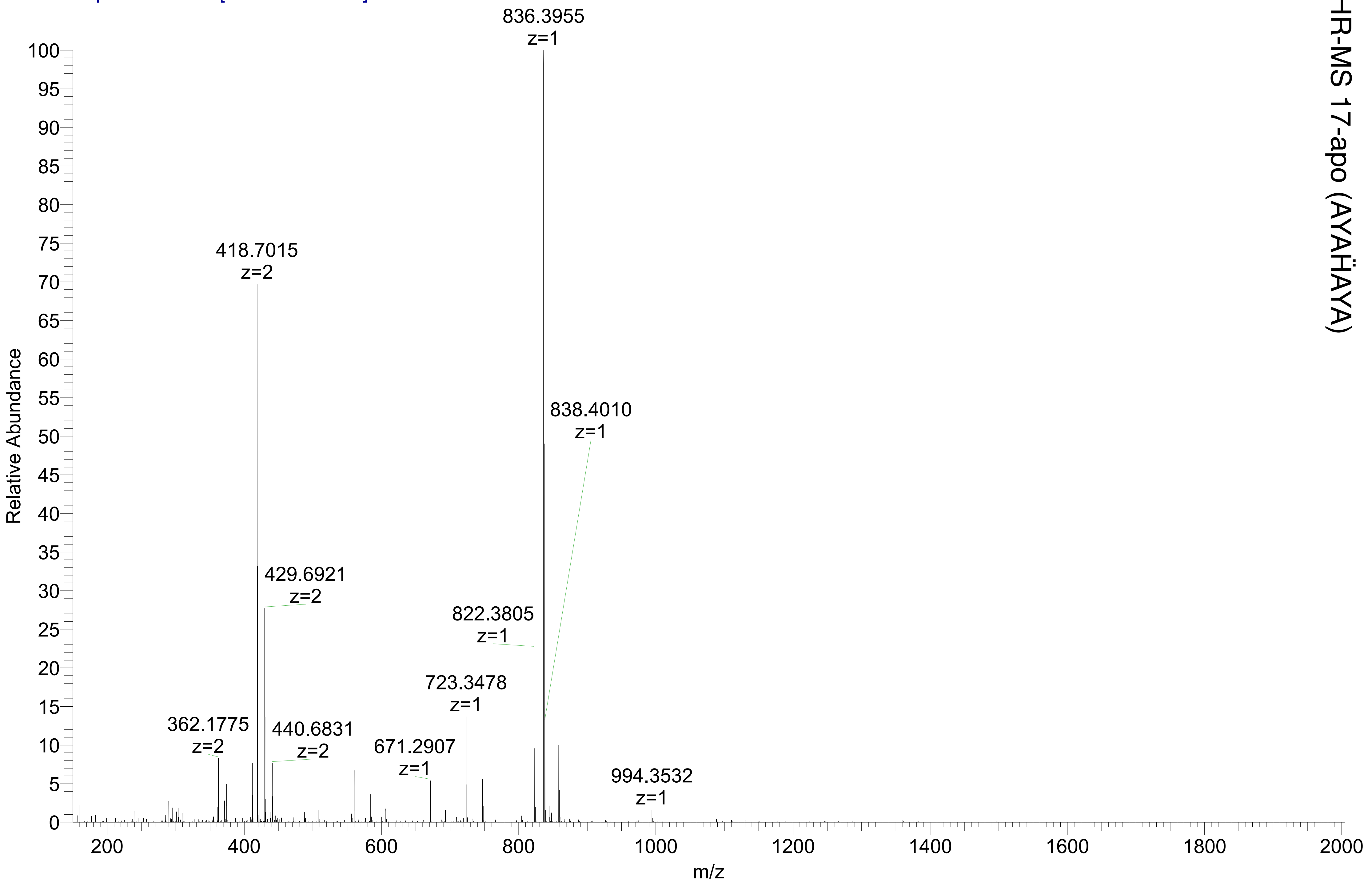

HR-MS 17-apo (AYAḦAYA)

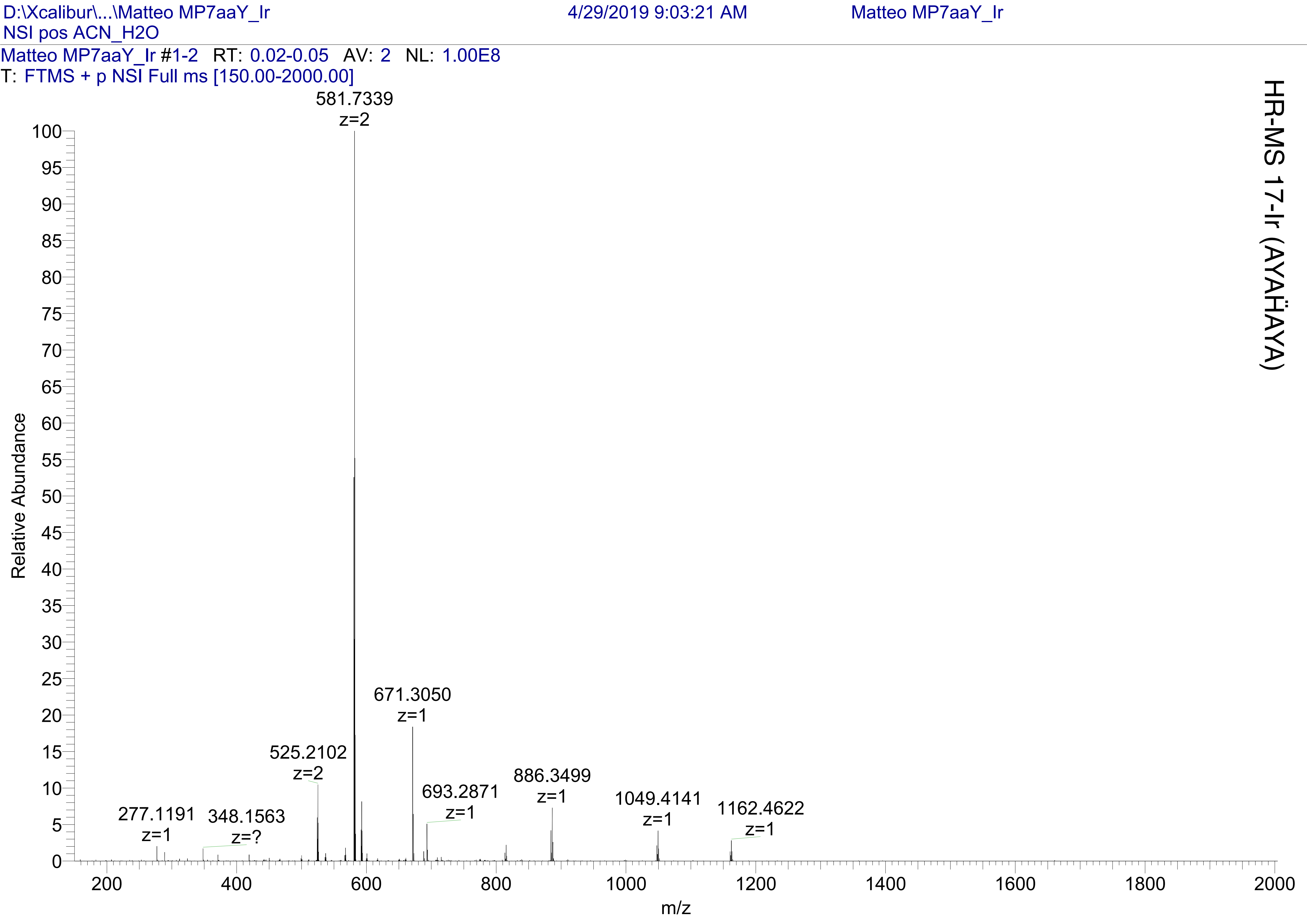

D:\Xcalibur\...\Matteo MP7aaY_Ir
4/29/2019 9:03:21 AM
Matteo MP7aaY_Ir
NSI pos ACN_H2O
Matteo MP7aaY_Ir #1-2 RT: 0.02-0.05 AV: 2 NL: 1.00E8
T: FTMS + p NSI Full ms [150.00-2000.00]
HR-MS 17-Ir (AYAḦAYA)
581.7339
z=2
671.3050
z=1
525.2102
z=2
693.2871
z=1
886.3499
z=1
1049.4141
z=1
1162.4622
z=1
277.1191
z=1
348.1563
z=?
Relative Abundance
m/z

F:\Xcalibur\Data\Zamolo\MATTEO\MP_7aaY-apo.raw 06.05.2019 08:49:52

RT :0.00-10.00 GNL: 7.38E5 TIC MS MP_7aaY-apo

LC-MS 17-apo (AYAḦAYA)

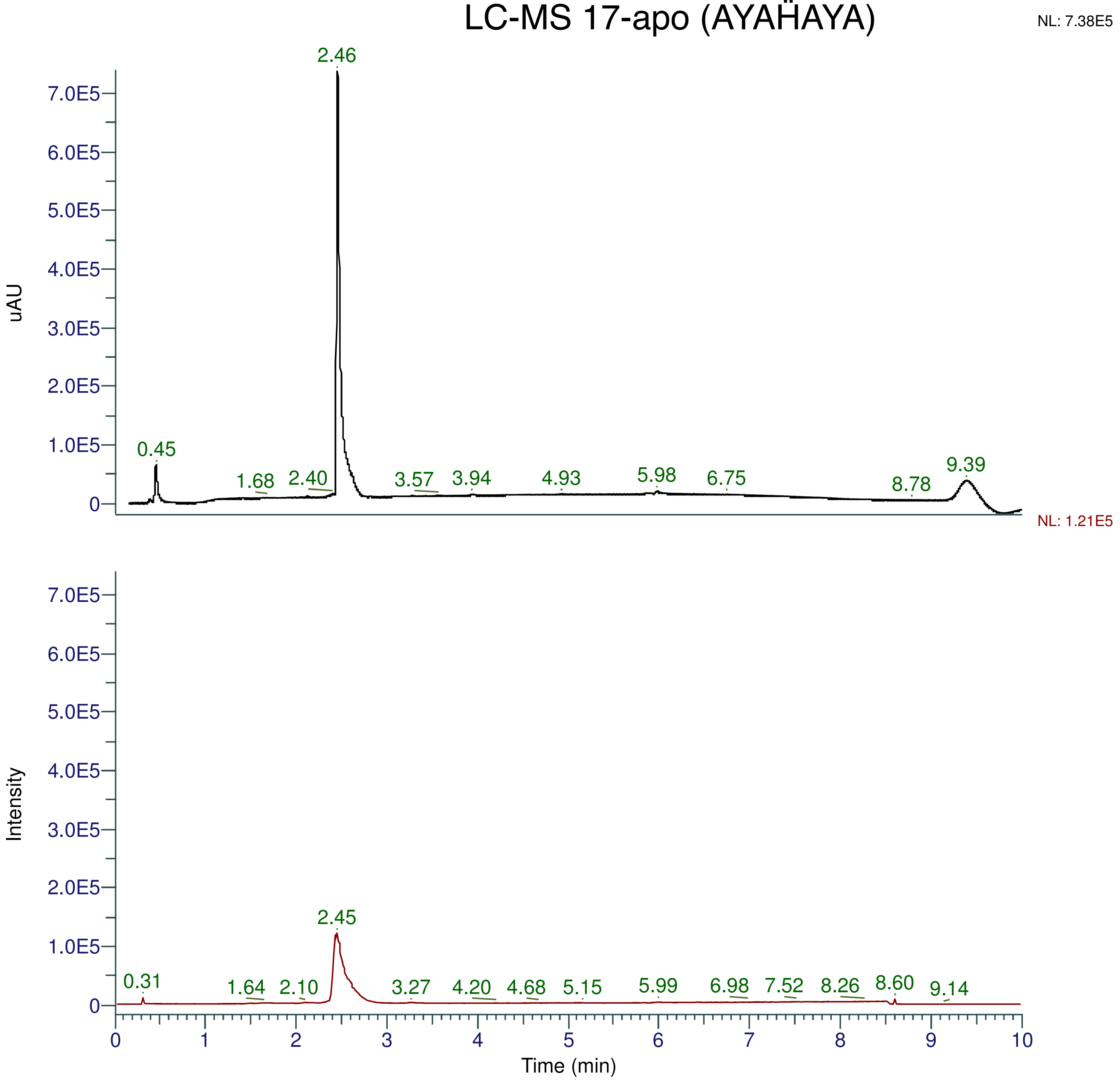

MP_7aaY-apo #130 RT: 2.44 AV: 1 NL: 6.72E+004

T: ITMS + c ESI Full ms [150.00-2000.00]

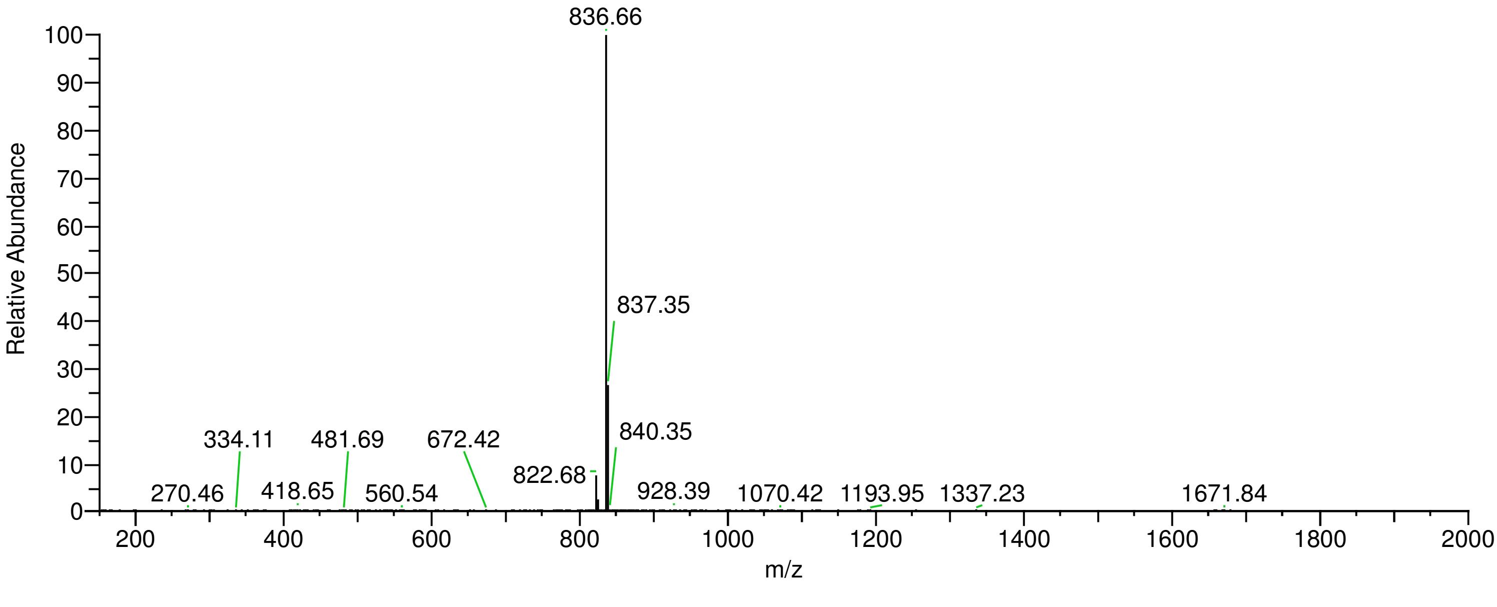

RT :0.00-10.00 GNL: 8.08E5 TIC MS MP_7aaY-Ir

LC-MS 17-Ir (AYAḦAYA)

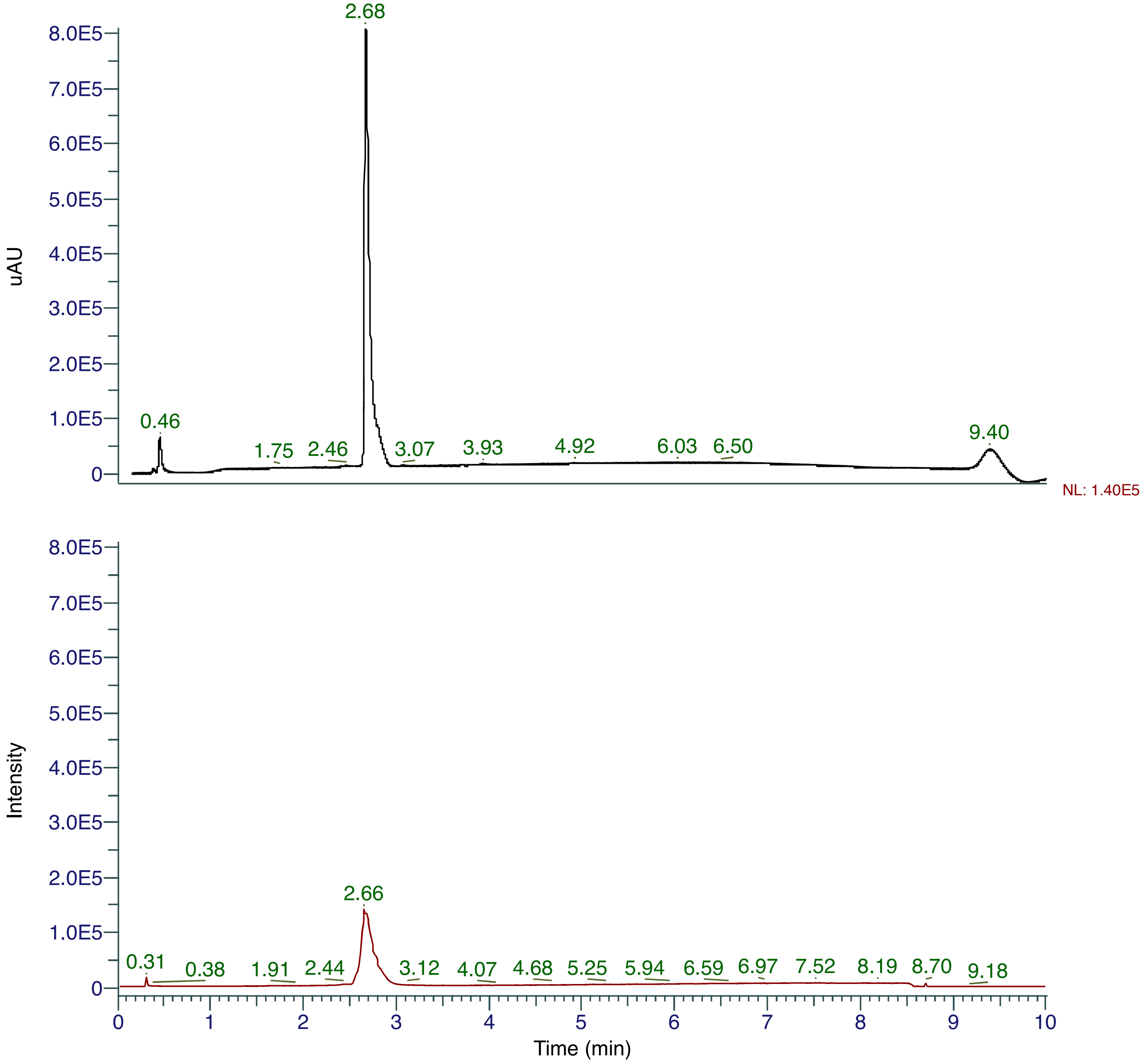

MP_7aaY-Ir #146 RT: 2.71 AV: 1 NL: 2.25E+004
T: ITMS + c ESI Full ms [150.00-2000.00]

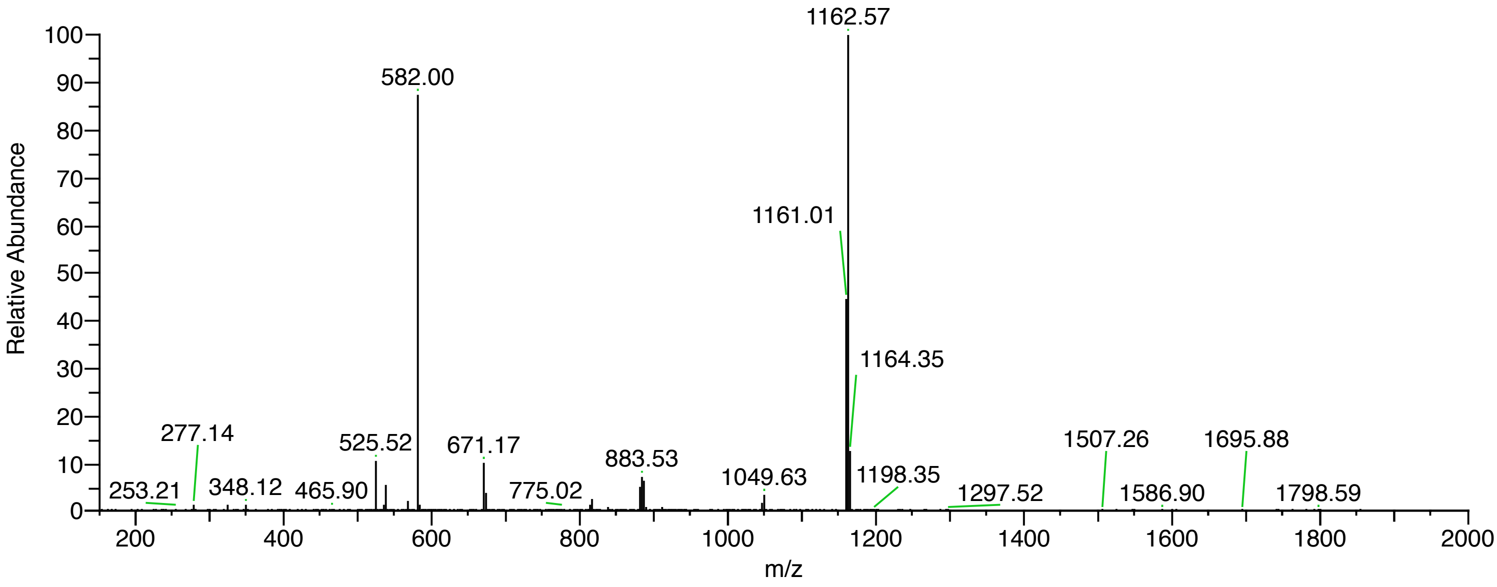

HR-MS 18-apo (ACAẌACA)

planchestainer MP 15618_ACAXACA_18061... 6/19/2018 10:17:03 AM planchestainer MP 15618_ACAXACA
NSI pos H2O_ACN

planchestainer MP 15618_ACAXACA_180615104148 #1-4 RT: 0.00-0.08 AV: 4 NL: 1.77E9
T: FTMS + p NSI Full ms [100.00-2000.00]

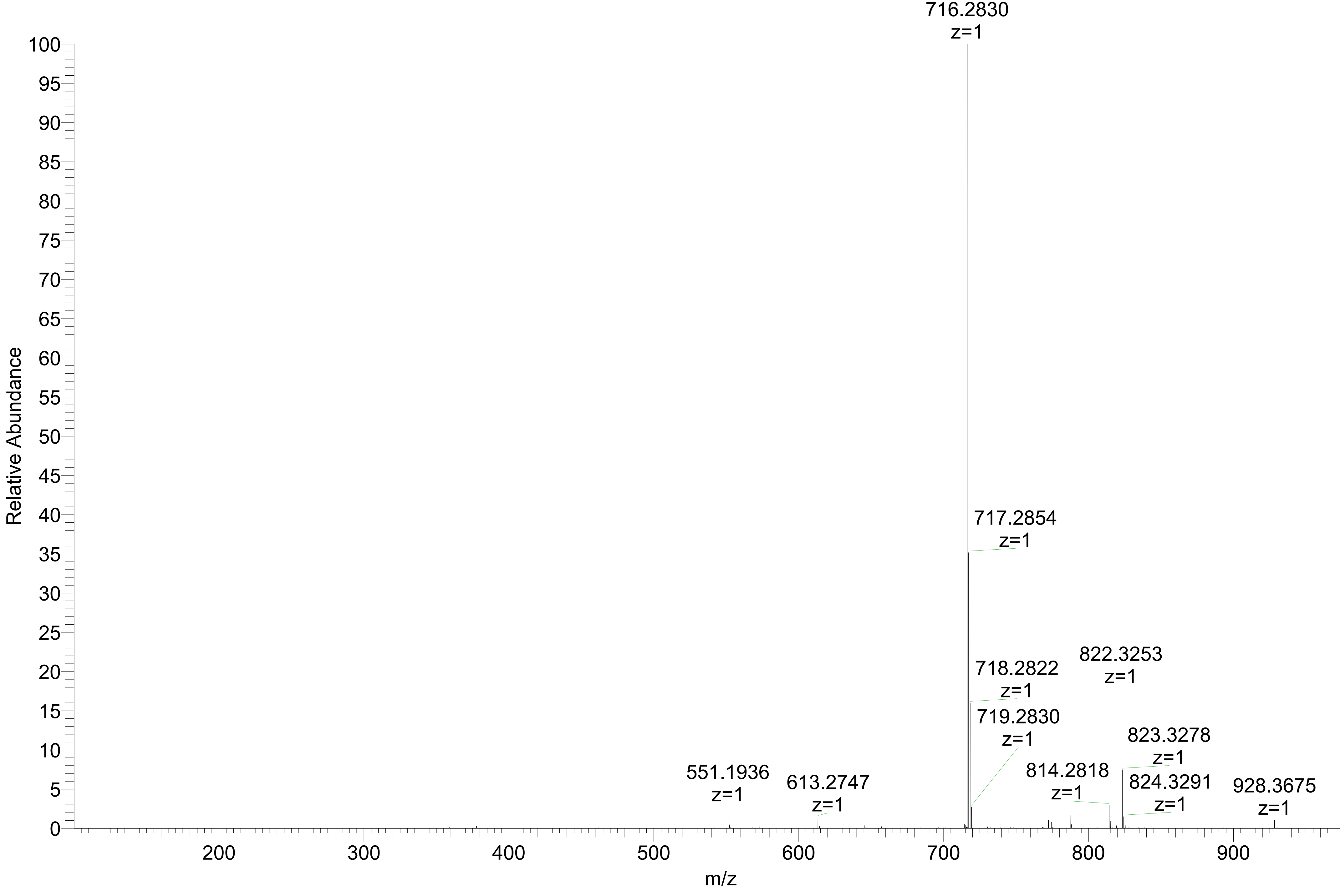

F:\Xcalibur\Data\Zamolo\MATTEO\KL779C-APO.raw 14.11.2018 10:19:33

RT :0.00-10.00 TIC MS KL779C-APO

LC-MS 18-apo (ACAḦACA)

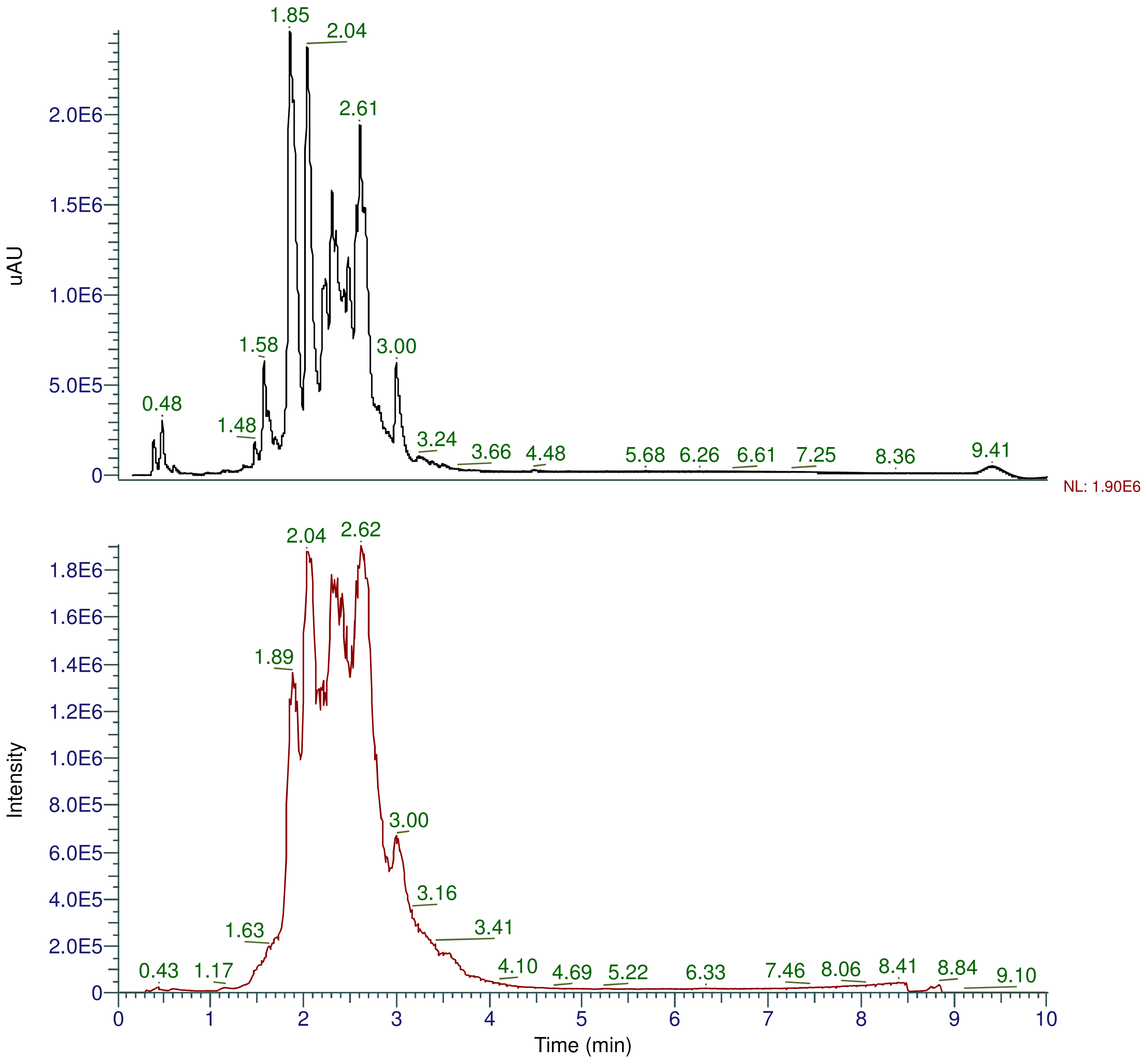

KL779C-APO #3 RT: 0.04 AV: 1 NL: 6.46E+000

T: ITMS + c ESI Full ms [150.00-2000.00]

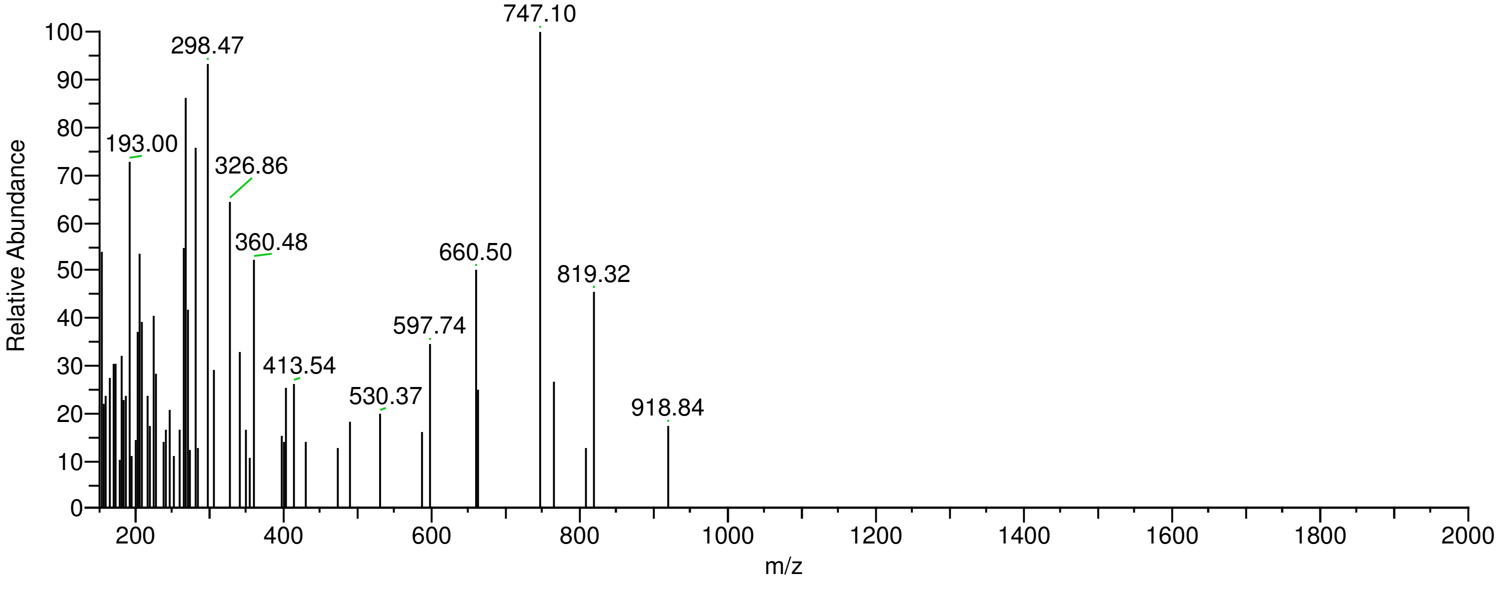

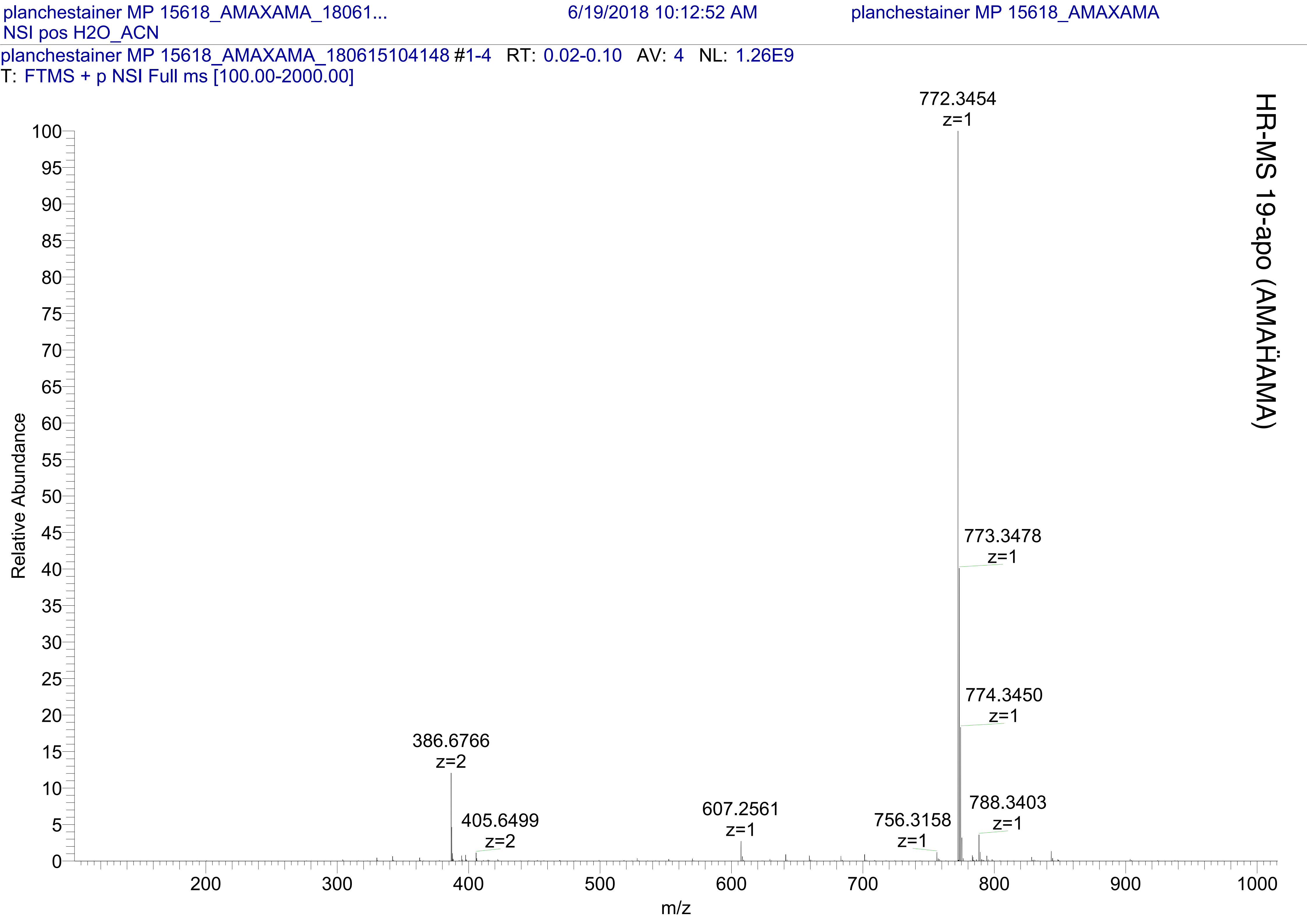
planchestainer MP 15618_AMAXAMA_18061...
6/19/2018 10:12:52 AM
planchestainer MP 15618_AMAXAMA
NSI pos H2O_ACN
planchestainer MP 15618_AMAXAMA_180615104148 #1-4 RT: 0.02-0.10 AV: 4 NL: 1.26E9
T: FTMS + p NSI Full ms [100.00-2000.00]
HR-MS 19-apo (AMAЙAMA)
772.3454 z=1
773.3478 z=1
774.3450 z=1
788.3403 z=1
756.3158 z=1
607.2561 z=1
405.6499 z=2
386.6766 z=2
Relative Abundance
m/z

F:\Xcalibur\Data\Zamolo\MATTEO\MP7aaM-apo_pure.raw 10.01.2019 11:02:50

RT :0.00-10.00 TIC MS MP7aaM-apo_pure

LC-MS 19-apo (AMAḦAMA)

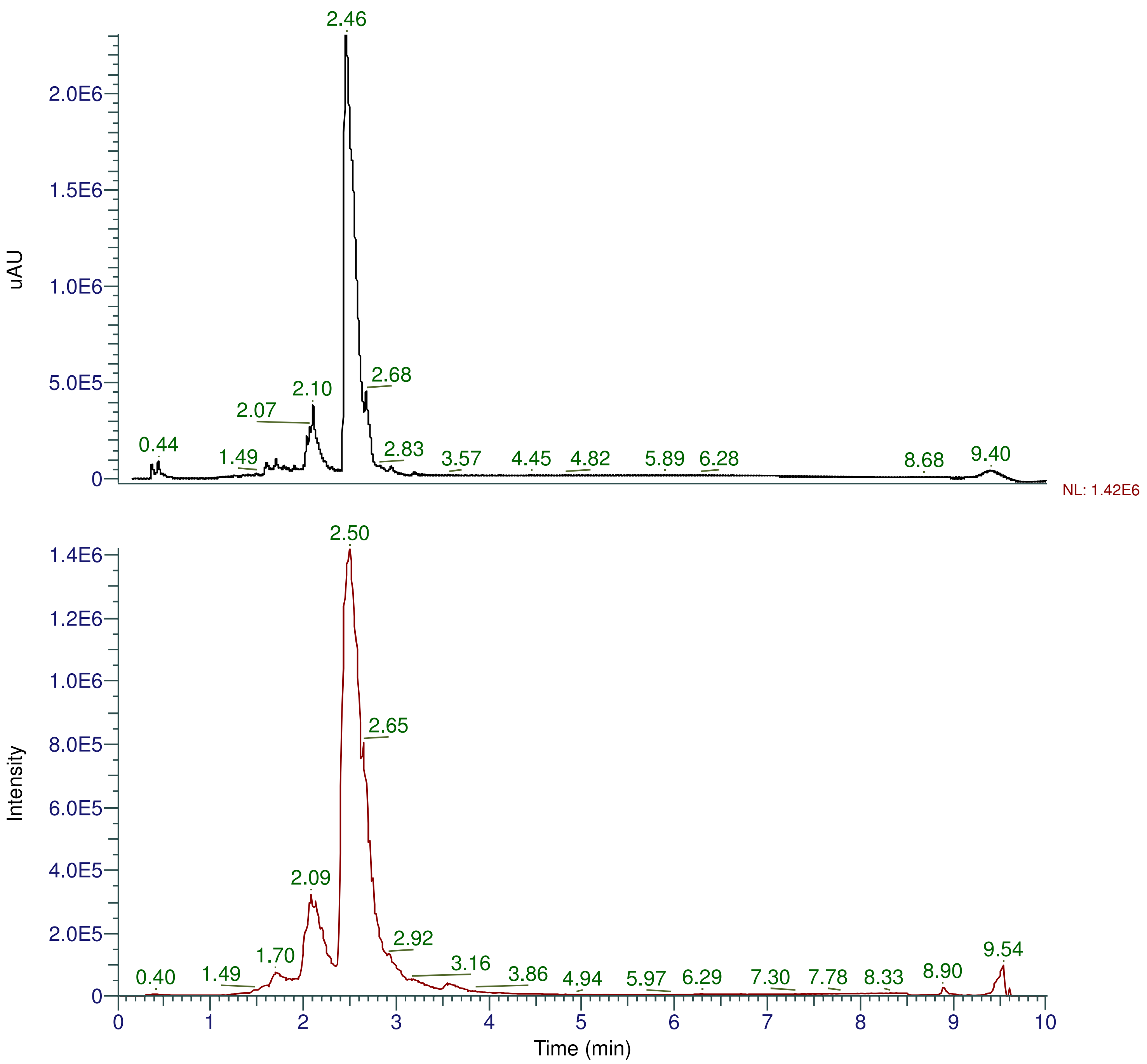

MP7aaM-apo_pure #157 RT: 2.49 AV: 1 NL: 7.91E+005
T: ITMS + c ESI Full ms [150.00-2000.00]

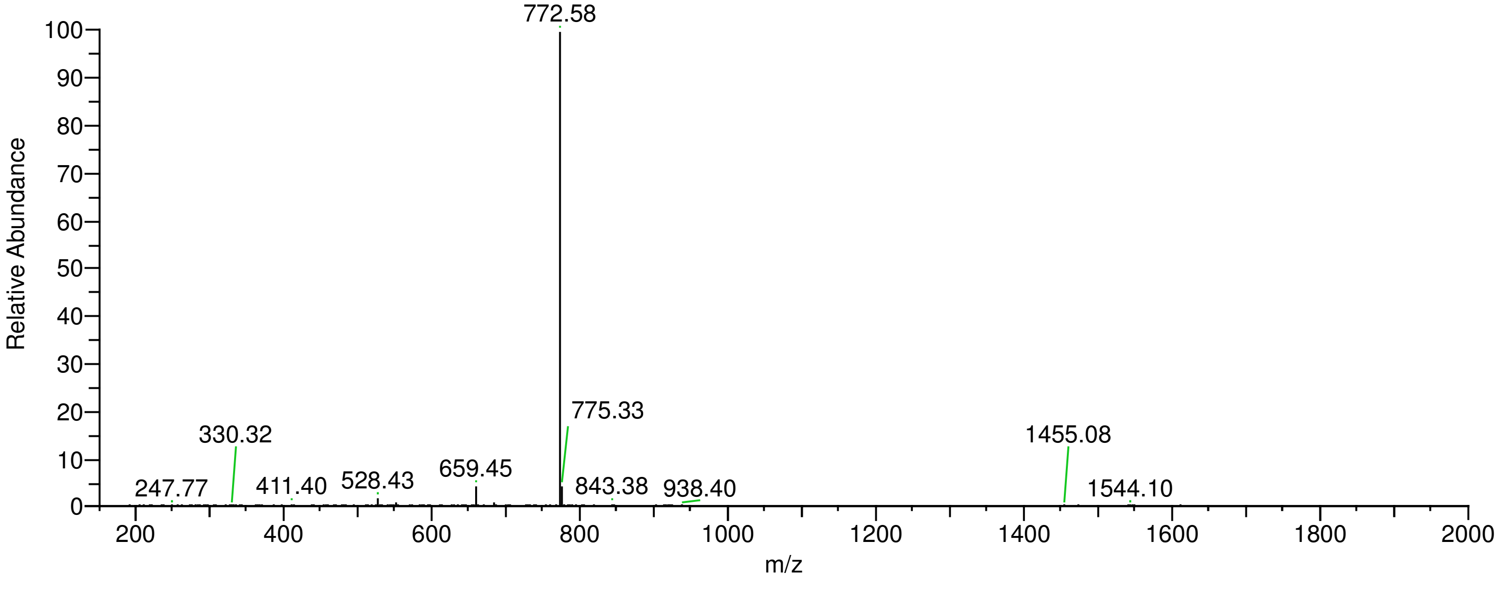

HR-MS 20-apo (AAAḦAAA)

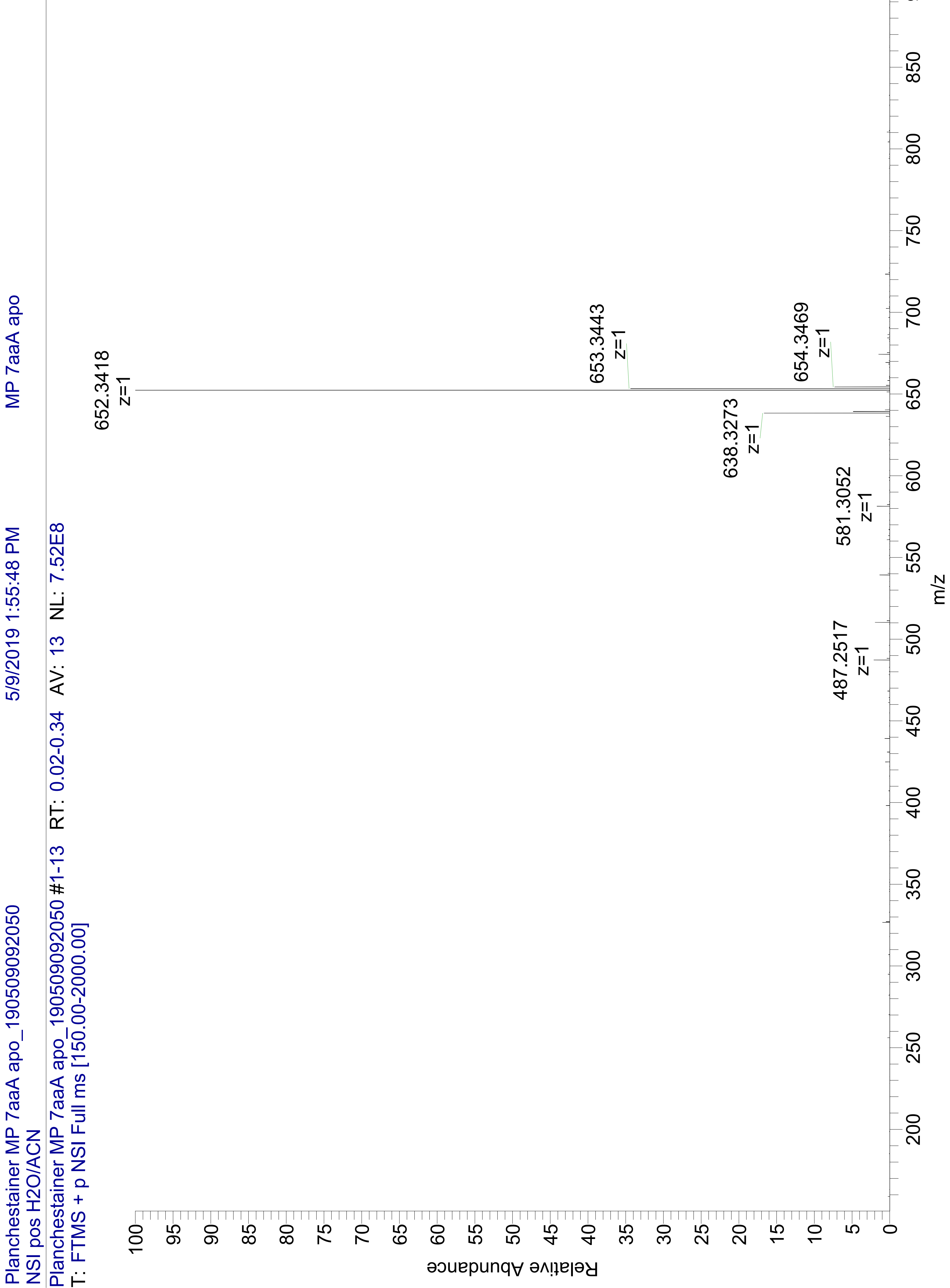

Planchestainer MP7aaA_Ir F2_190520163239 5/20/2019 4:37:19 PM MP7aaA_Ir F2
Velos NSI pos

Planchestainer MP7aaA_Ir F2_190520163239 #147-160 RT: 3.68-3.93 AV: 14 NL: 2.22E6
T: FTMS + p NSI Full ms [150.00-2000.00]

HR-MS 20-Ir (AAAḦAAA)

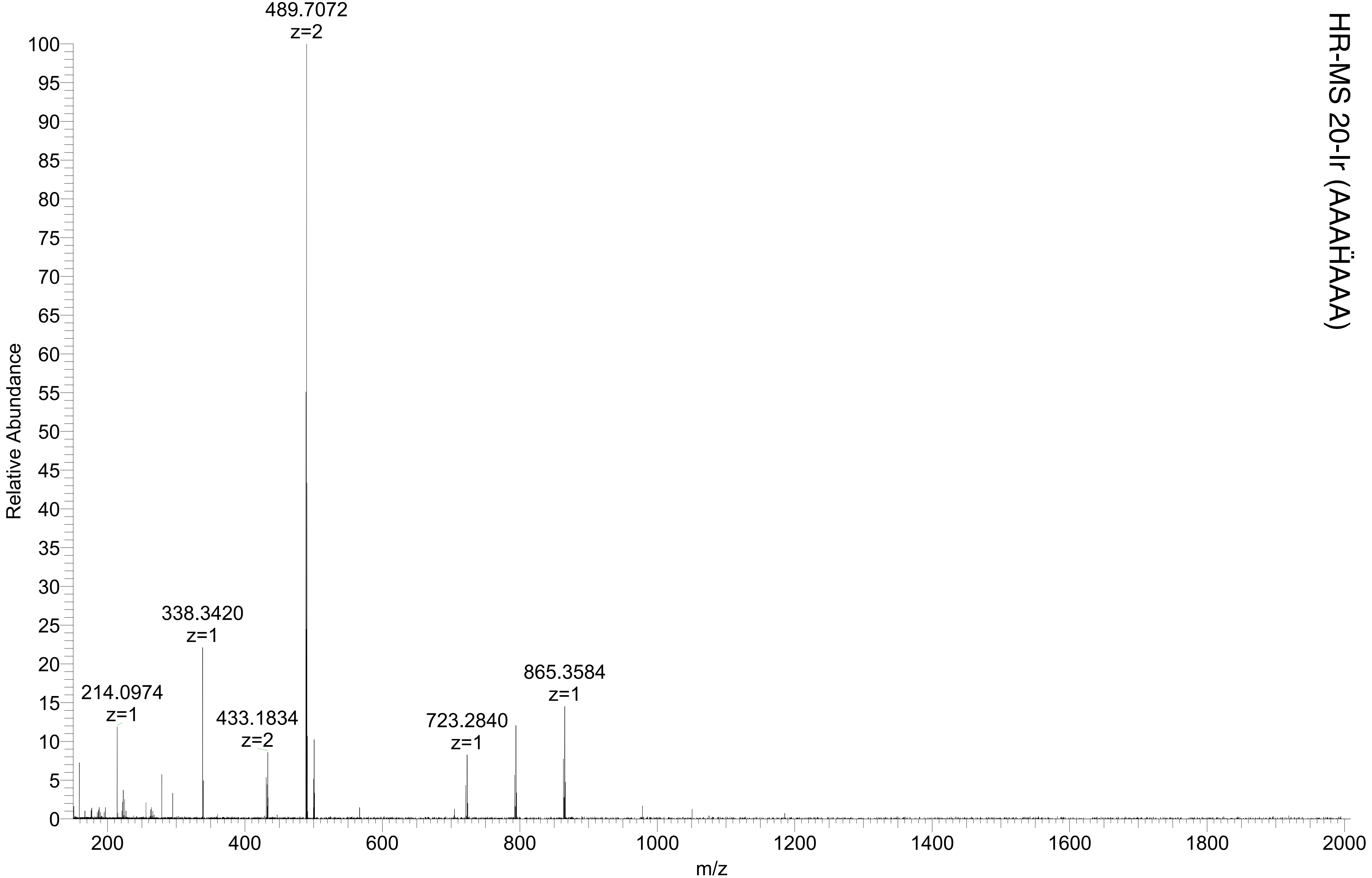

F:\Xcalibur\Data\Zamolo\MATTEO\MP_7aaA_apo.raw 29.05.2019 09:44:44

RT :0.00-10.00 GNL: 2.92E6 TIC MS MP_7aaA_apo

LC-MS 20-apo (AAAȞAAA)

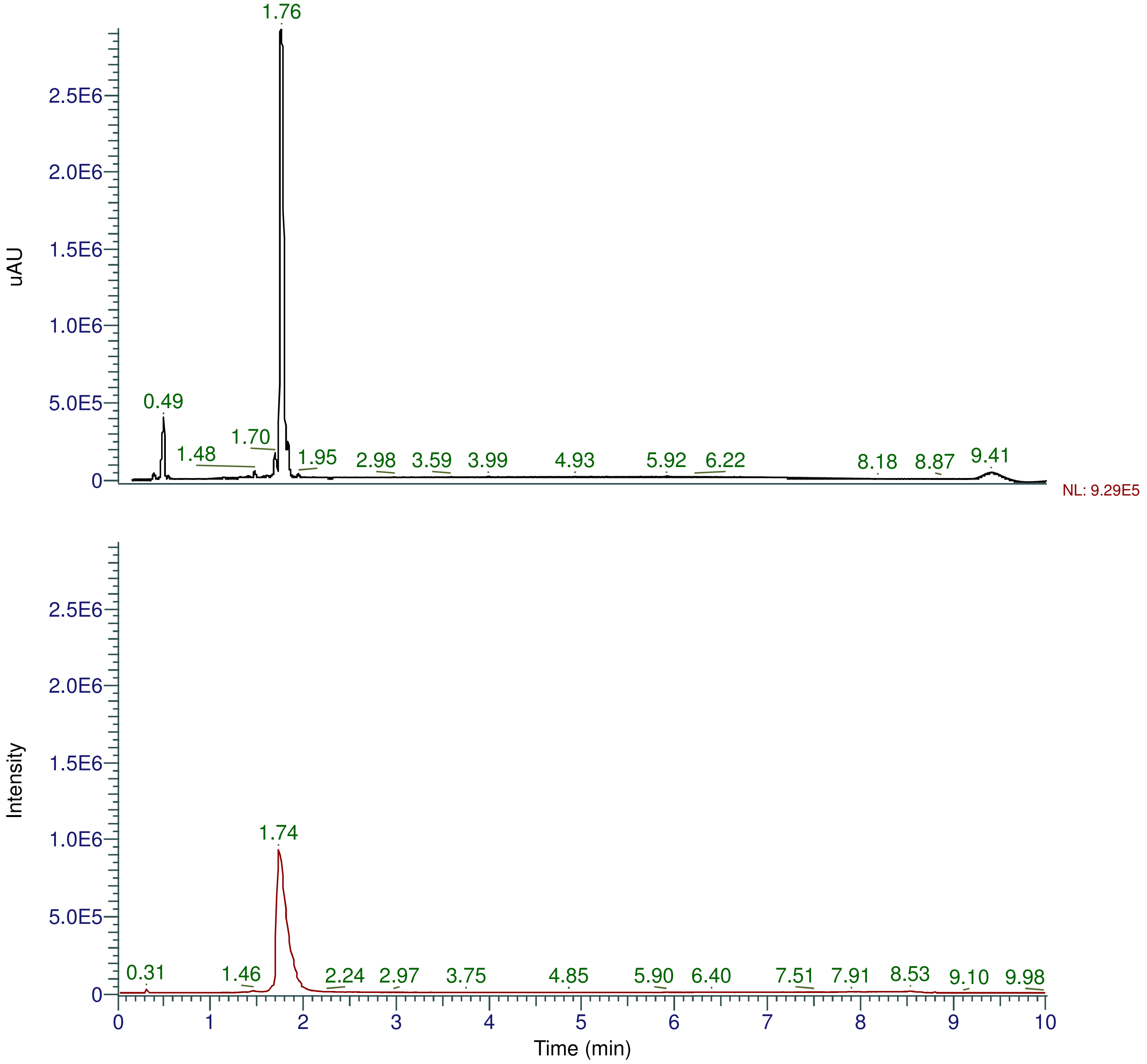

MP_7aaA_apo #96 RT: 1.75 AV: 1 NL: 5.86E+005
T: ITMS + c ESI Full ms [150.00-2000.00]

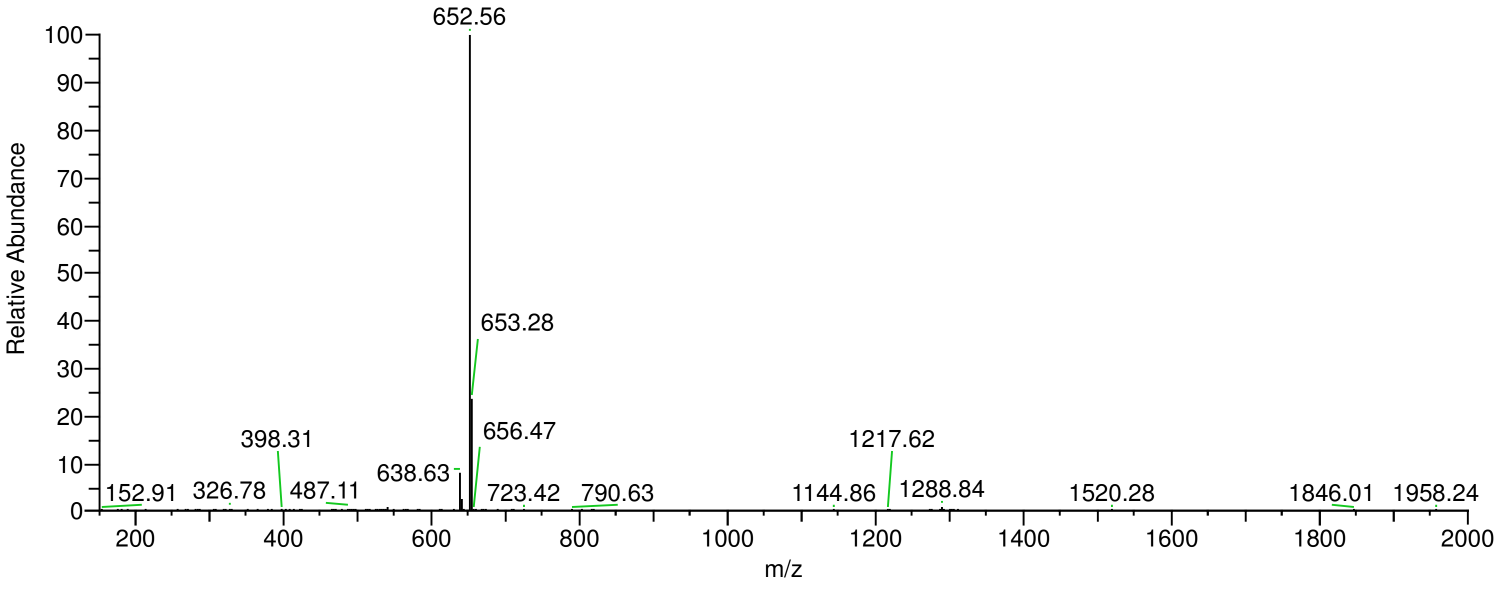

F:\Xcalibur\Data\Zamolo\MATTEO\MP_7aaA_Ir.raw 29.05.2019 09:43:37

RT :0.00-10.00 GNL: 2.24E6 TIC MS MP_7aaA_Ir

LC-MS 20-Ir (AAAḦAAA)

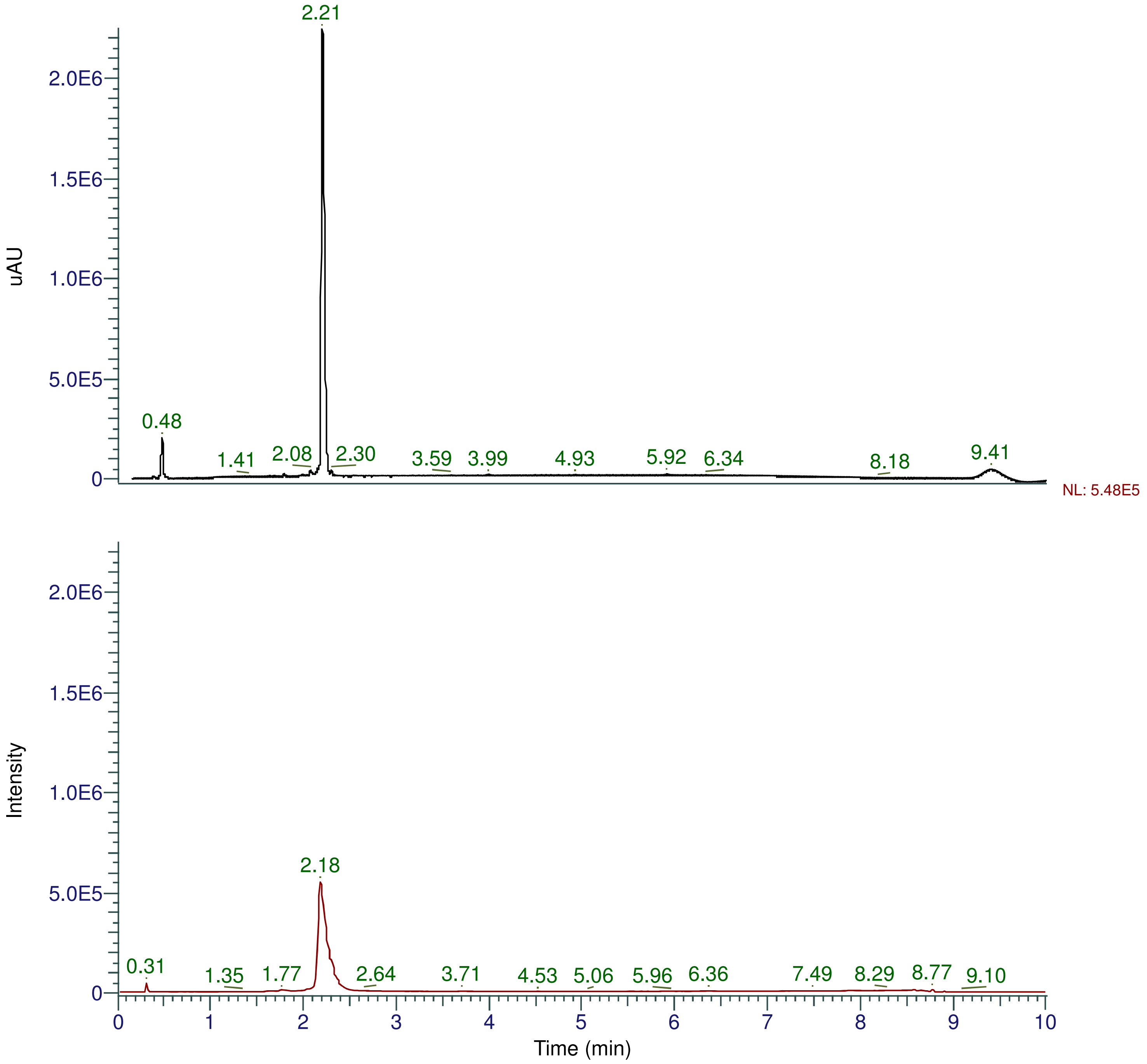

MP_7aaA_Ir #120 RT: 2.21 AV: 1 NL: 2.00E+005
T: ITMS + c ESI Full ms [150.00-2000.00]

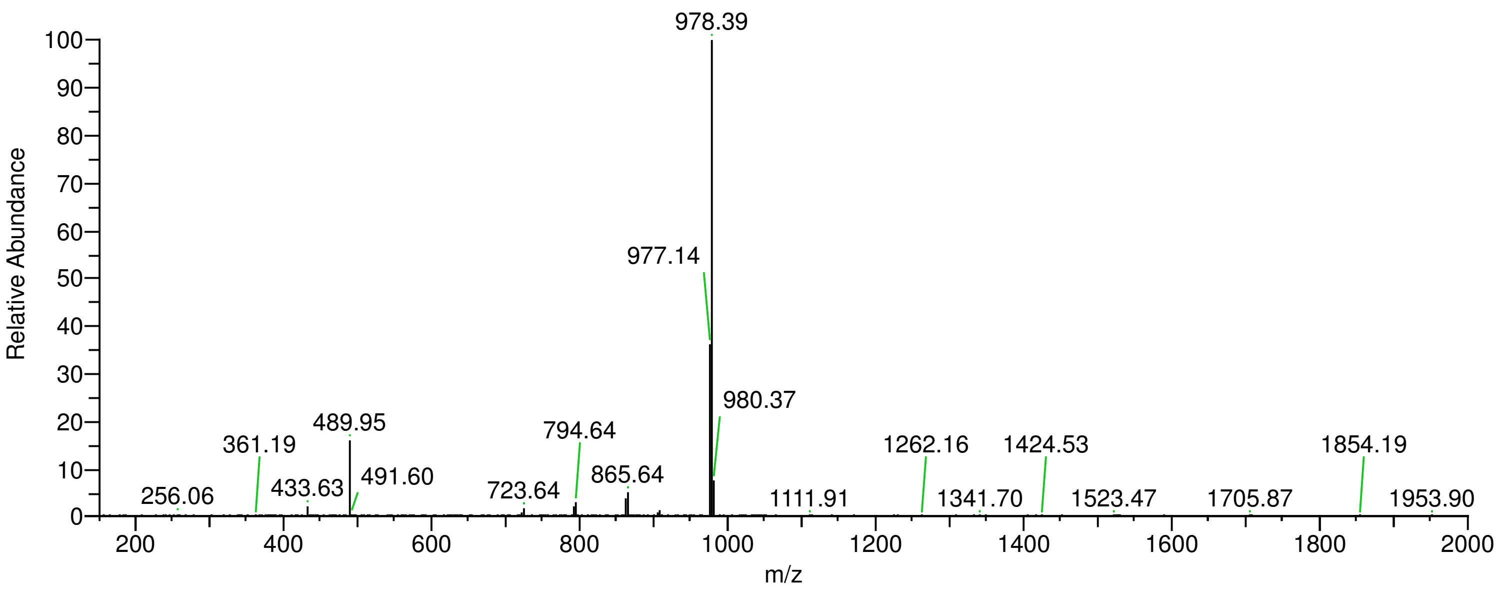

Matteo MP 7aaV_apo_pure_190111124157 1/11/2019 12:57:16 PM Matteo MP 7aaV_apo_pure
NSI pos ACN_H2O

Matteo MP 7aaV_apo_pure_190111124157 #1-4 RT: 0.02-0.10 AV: 4 NL: 8.71E7
T: FTMS + p NSI Full ms [150.00-2000.00]

HR-MS 21-apo (AVAḦAVA)

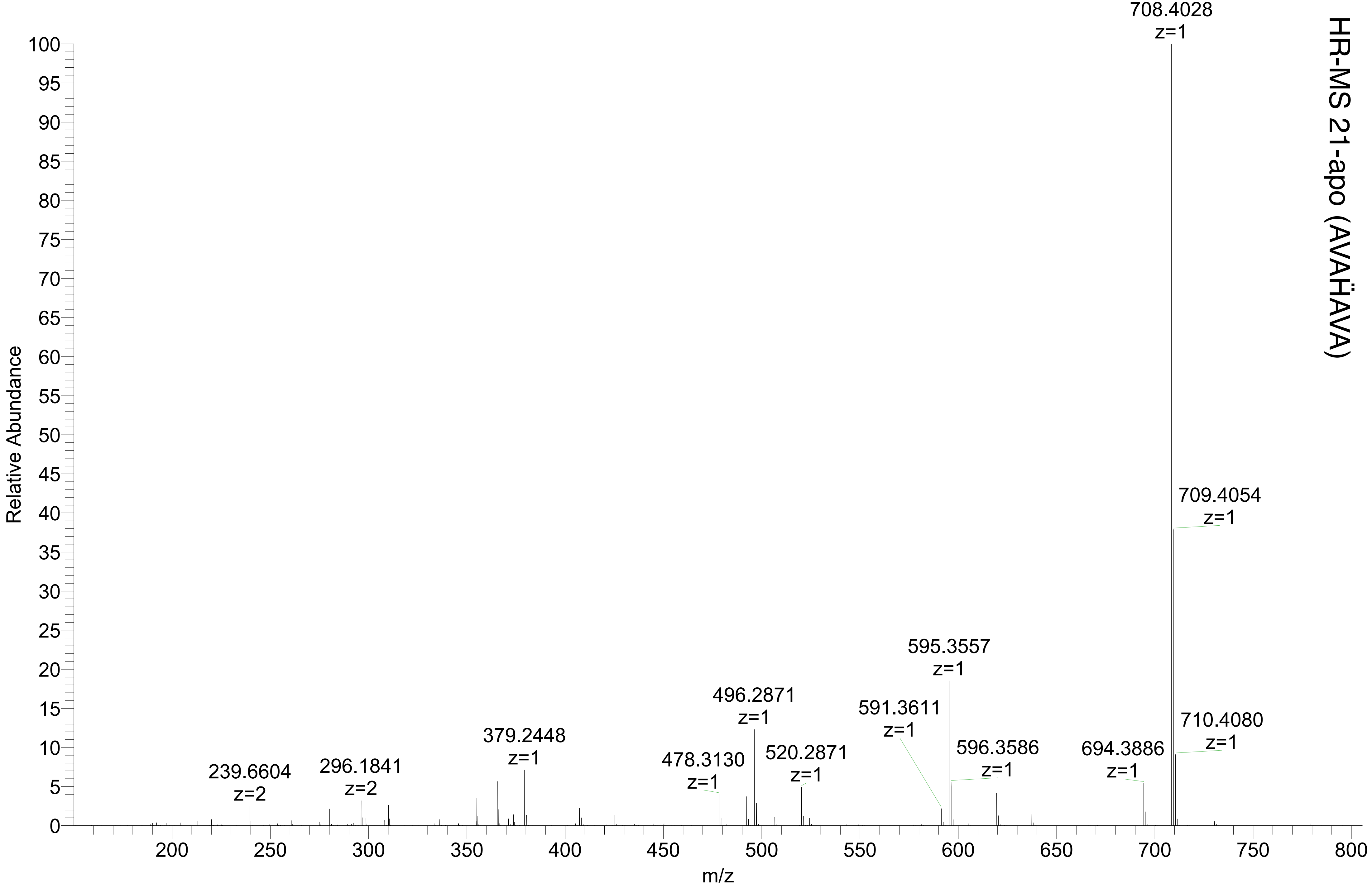

Planchestainer MP7aaV_Ir F2_190521090606 5/21/2019 9:23:35 AM MP7aaV_Ir F2
Velos NSI pos

Planchestainer MP7aaV_Ir F2_190521090606 #12-41 RT: 0.16-0.56 AV: 30 NL: 4.16E6
T: FTMS + p NSI Full ms [150.00-2000.00]

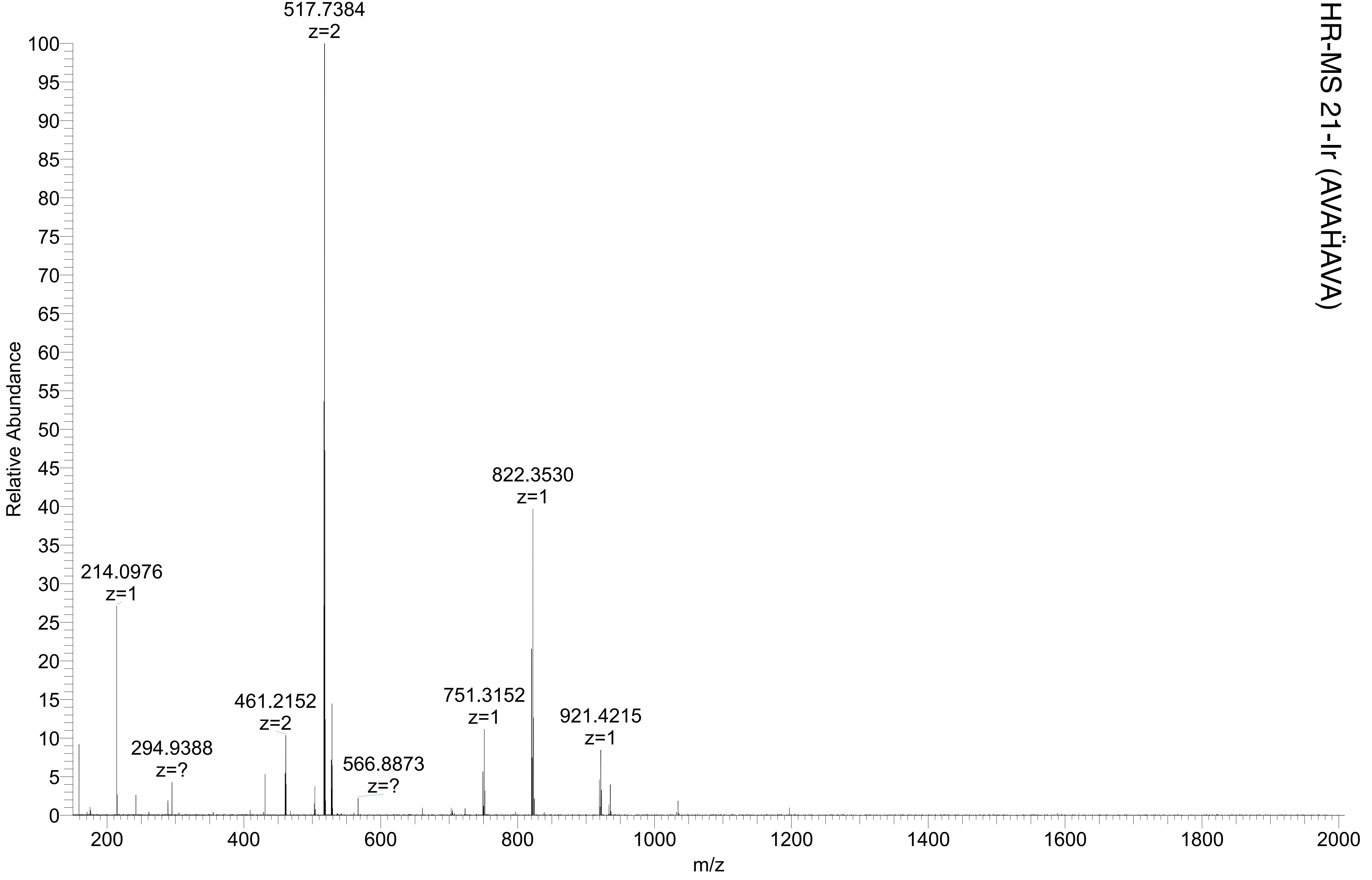

F:\Xcalibur\Data\Zamolo\MATTEO\MP_7aaV_apo.raw 29.05.2019 09:46:01

RT :0.00-10.00 GNL: 2.97E6 TIC MS MP_7aaV_apo

LC-MS 21-apo (AVAḦAVA)

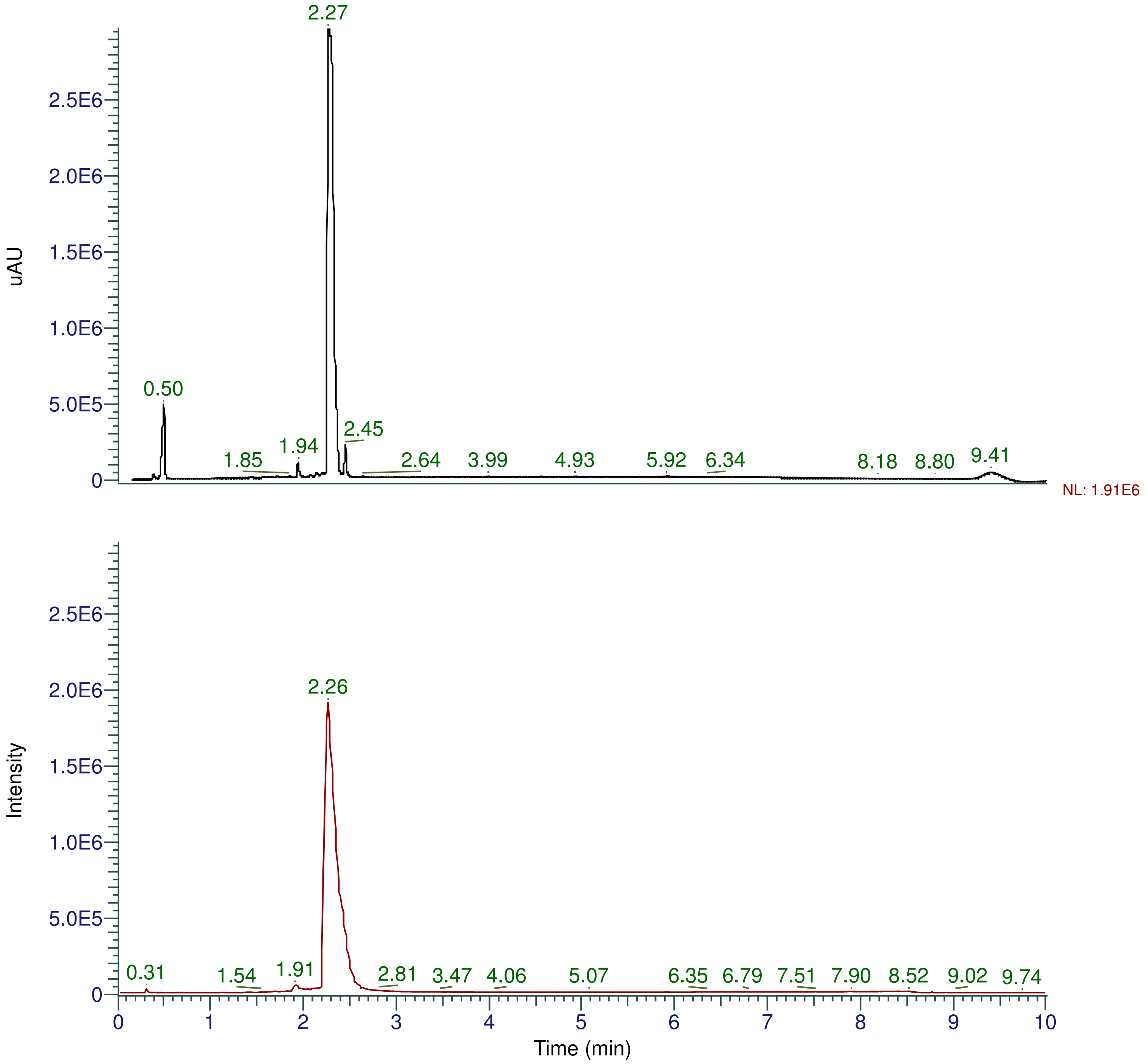

MP_7aaV_apo #130 RT: 2.30 AV: 1 NL: 9.64E+005

T: ITMS + c ESI Full ms [150.00-2000.00]

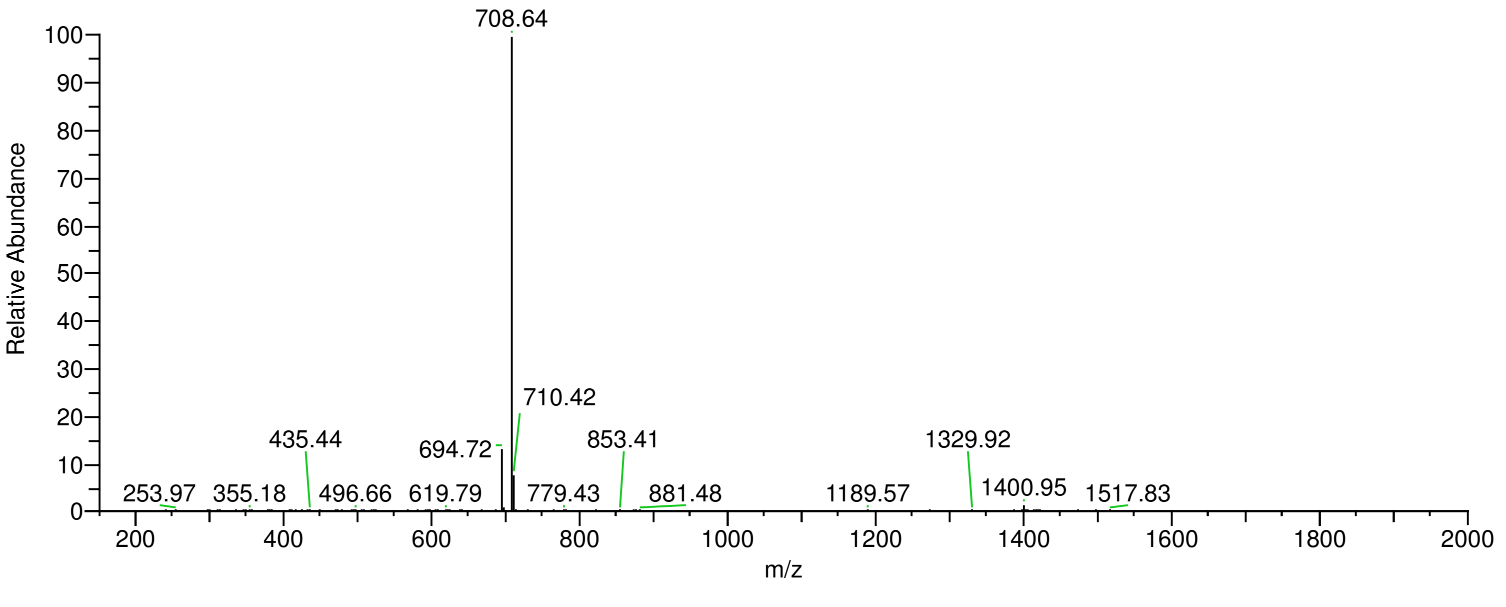

F:\Xcalibur\Data\Zamolo\MATTEO\MP_7aaV_Ir.raw 29.05.2019 09:45:29

RT :0.00-10.00 GNL: 1.66E6 TIC MS MP_7aaV_Ir

LC-MS 21-Ir (AVAḦAVA)

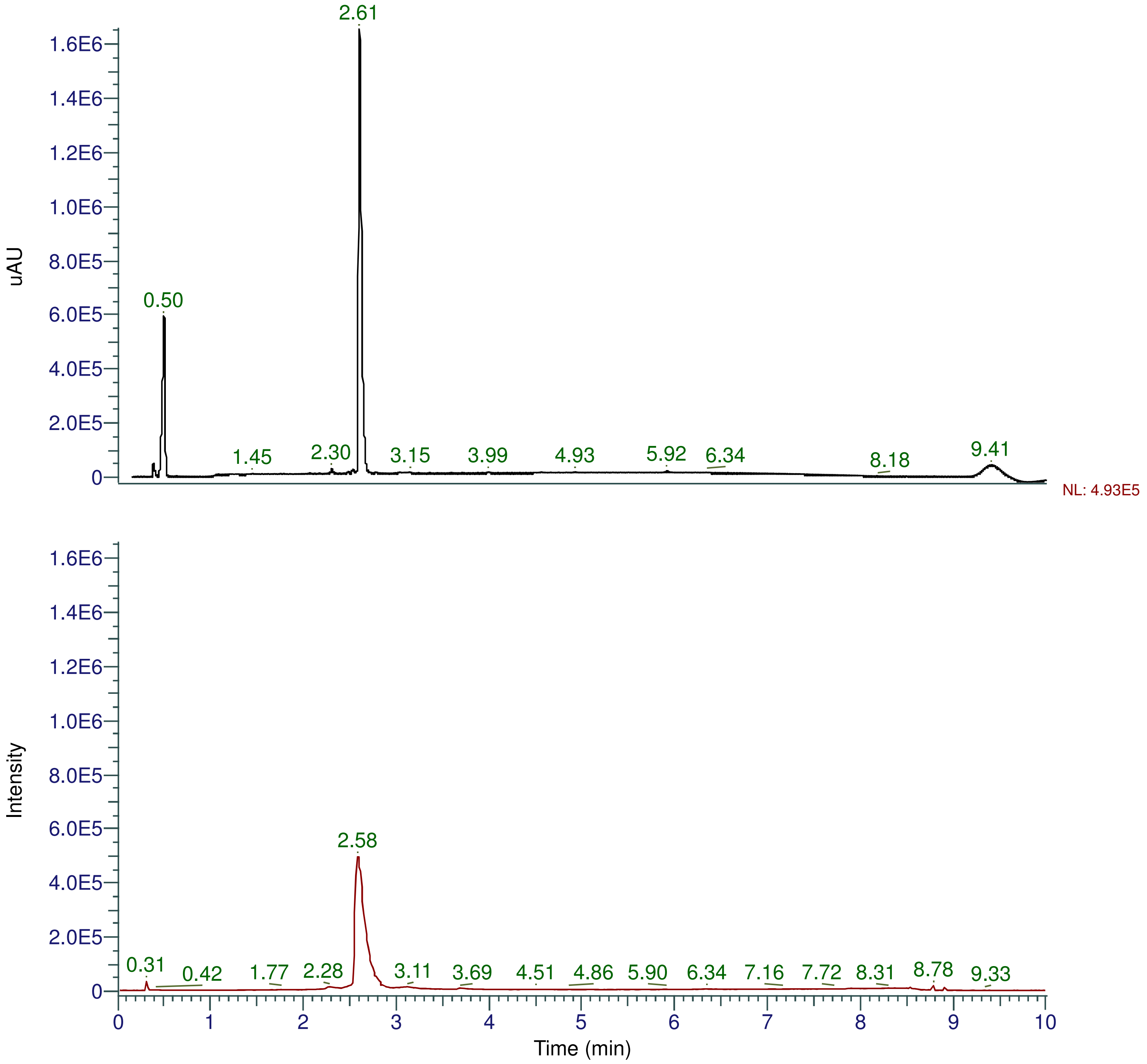

MP_7aaV_Ir #140 RT: 2.59 AV: 1 NL: 1.26E+005
T: ITMS + c ESI Full ms [150.00-2000.00]

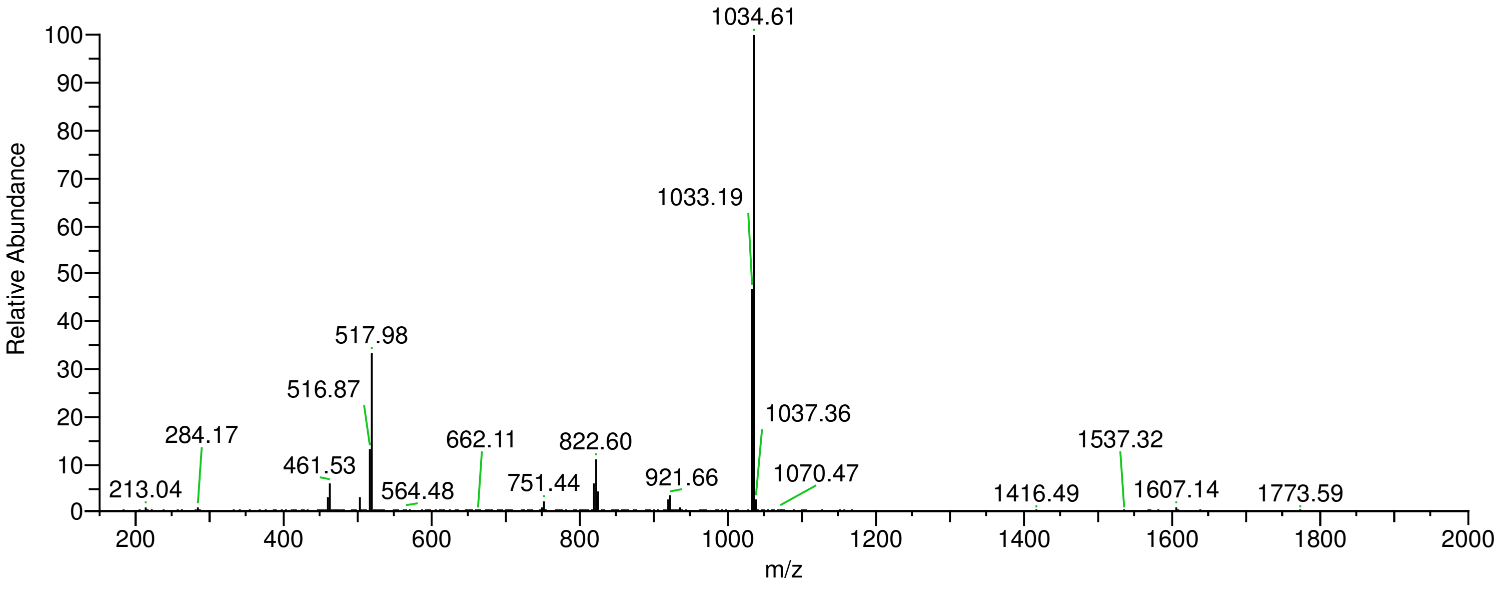

Feller IF 011 7aaP Apo_190408170200 4/9/2019 9:09:58 AM Feller IF 011 7aaP Apo
NSI pos H2O_MeOH

Feller IF 011 7aaP Apo_190408170200 #1-3 RT: 0.02-0.09 AV: 3 NL: 2.13E5
T: FTMS + p NSI Full ms [200.00-2000.00]

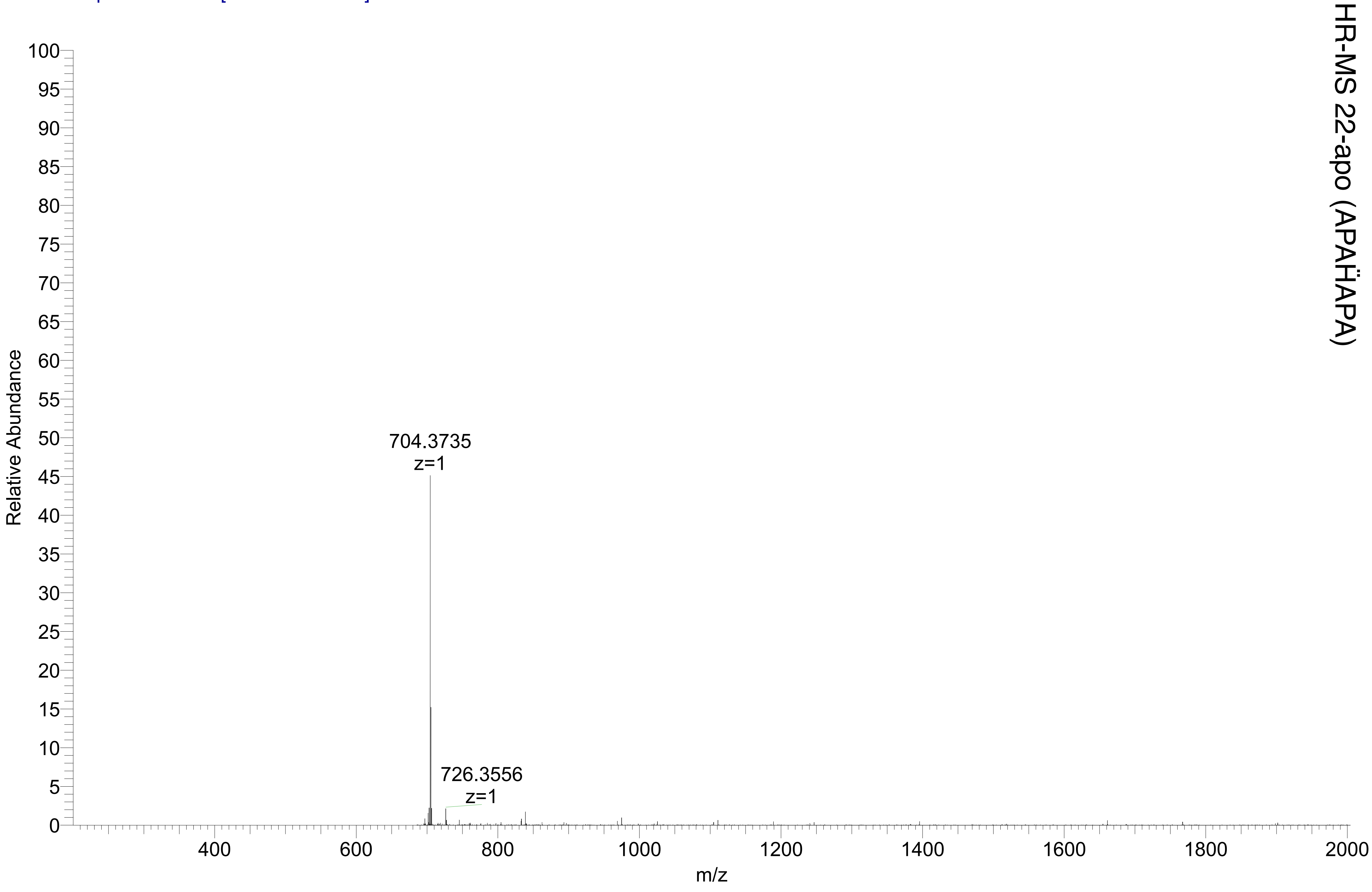

Feller IF 011 7aaP Ir_190408170200 4/9/2019 10:24:38 AM Feller IF 011 7aaP Ir
NSI pos H2O_MeOH

Feller IF 011 7aaP Ir_190408170200 #15-20 RT: 0.49-0.63 AV: 6 NL: 8.19E7
T: FTMS + p NSI Full ms [200.00-2000.00]

HR-MS 22-Ir (APAḦAPA)

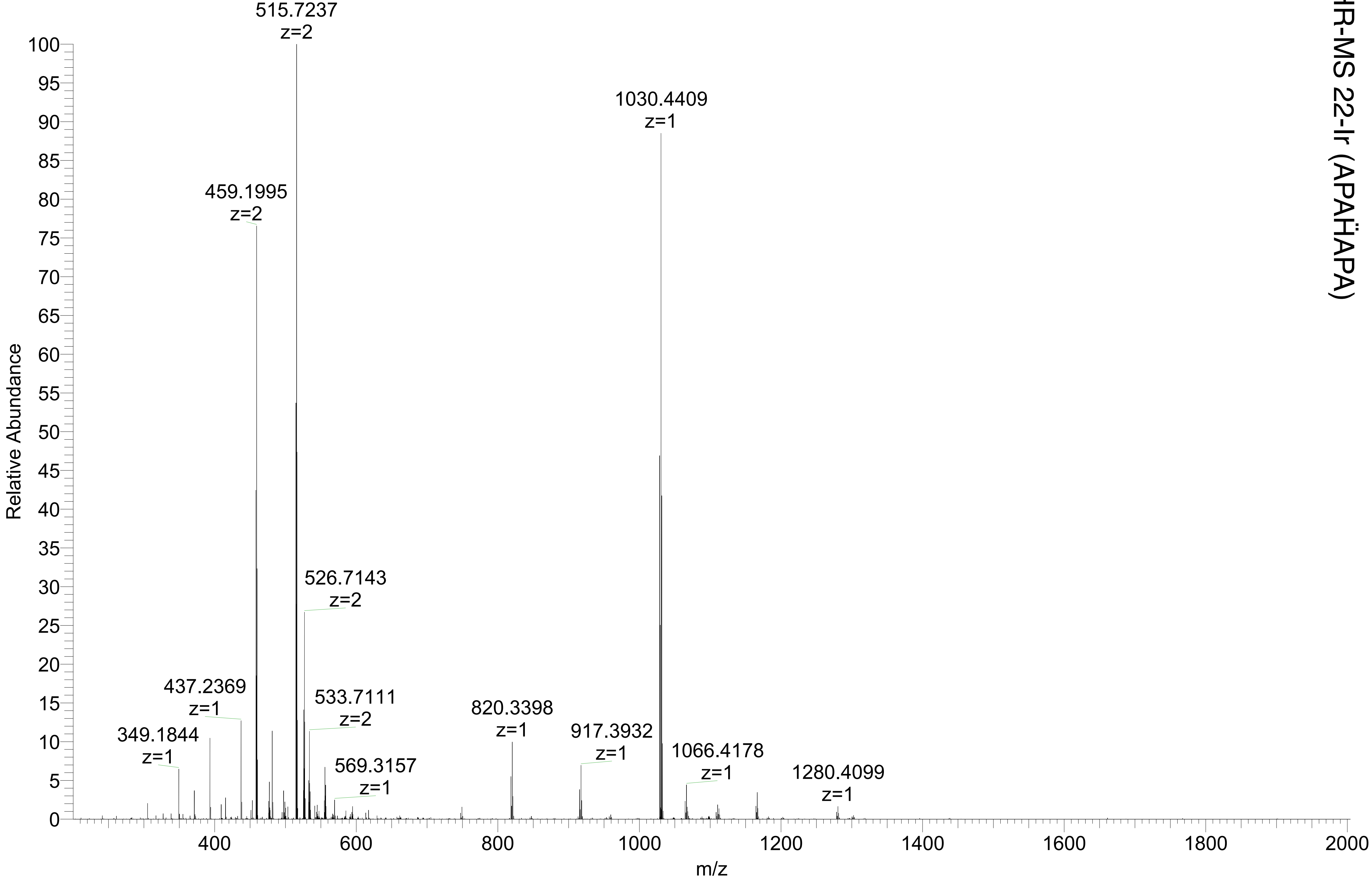

RT :0.00-10.00 GNL: 5.12E5 TIC MS MP_7aaP-apo

LC-MS 22-apo (APAḦAPA)

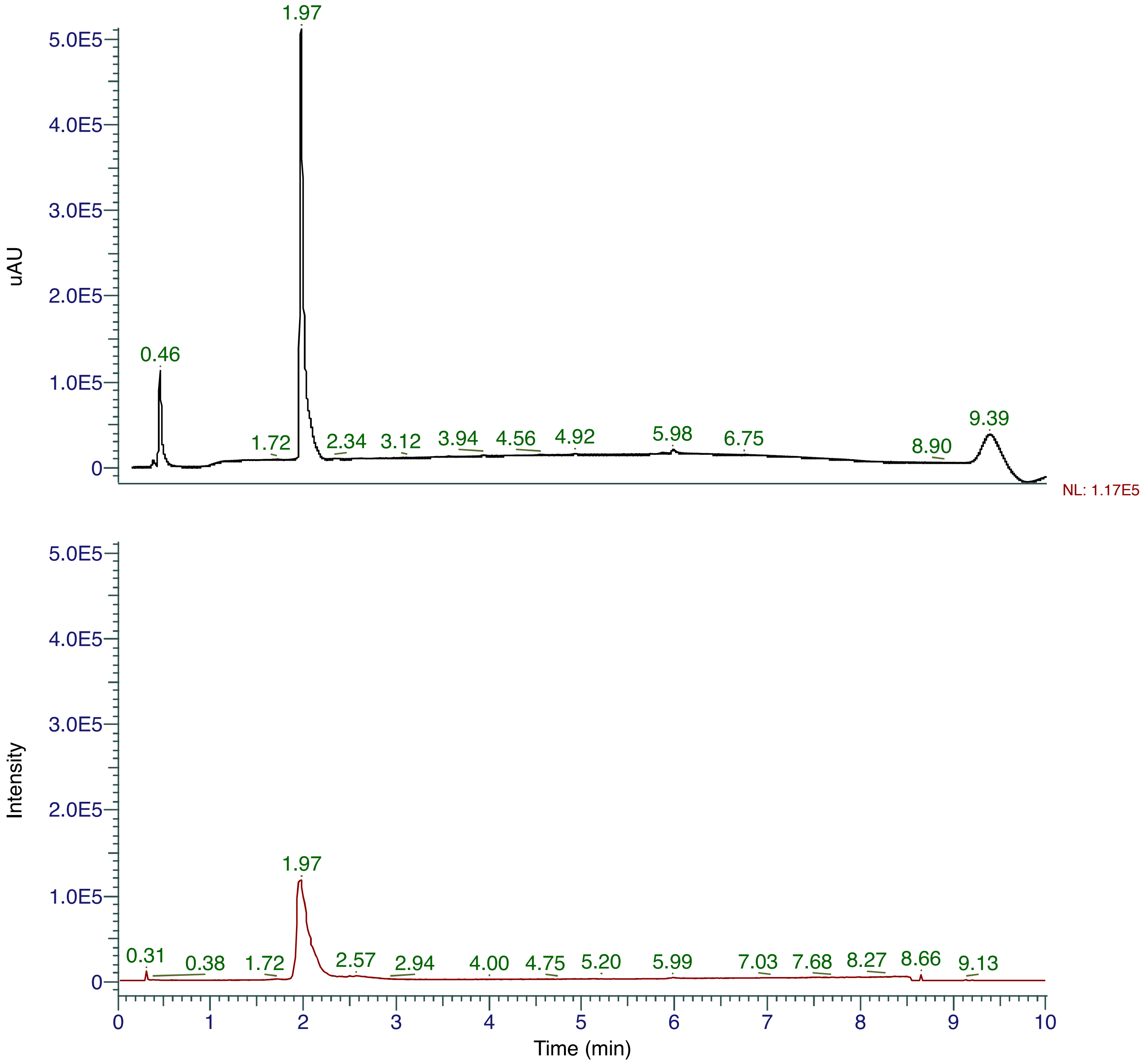

MP_7aaP-apo #101 RT: 1.91 AV: 1 NL: 5.47E+003
T: ITMS + c ESI Full ms [150.00-2000.00]

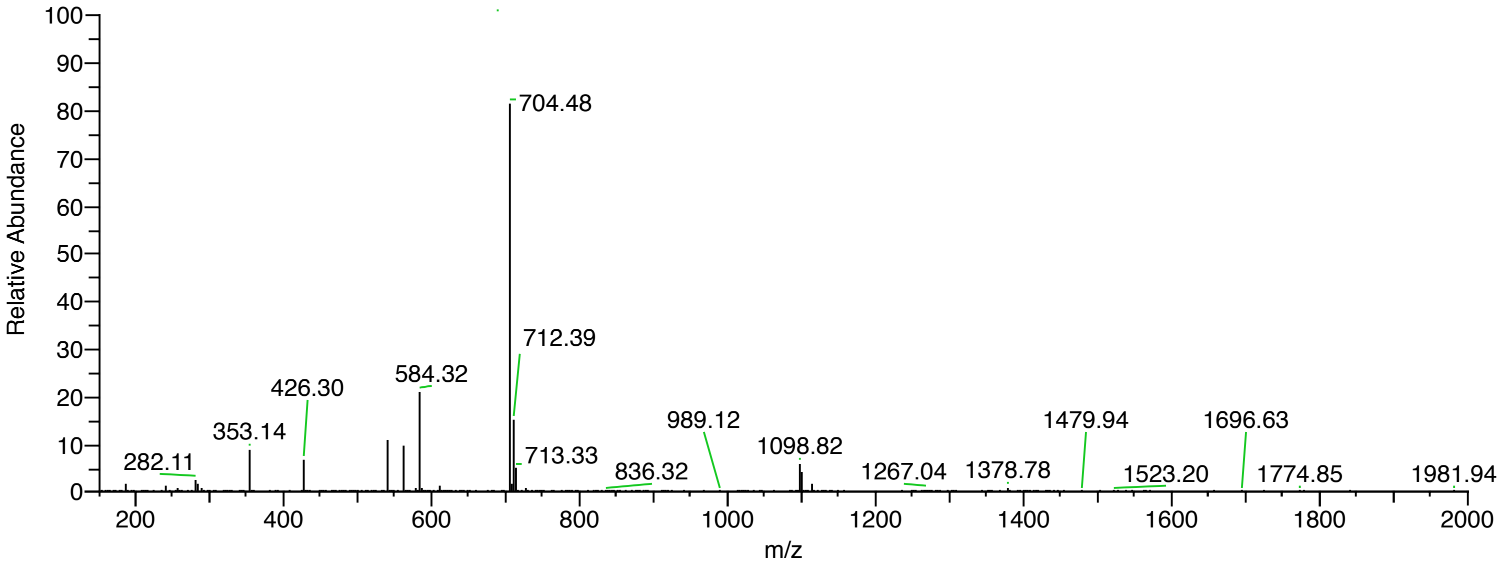

RT :0.00-10.00 GNL: 1.44E6 TIC MS MP_7aaP-Ir

LC-MS 22-Ir (APAḦAPA)

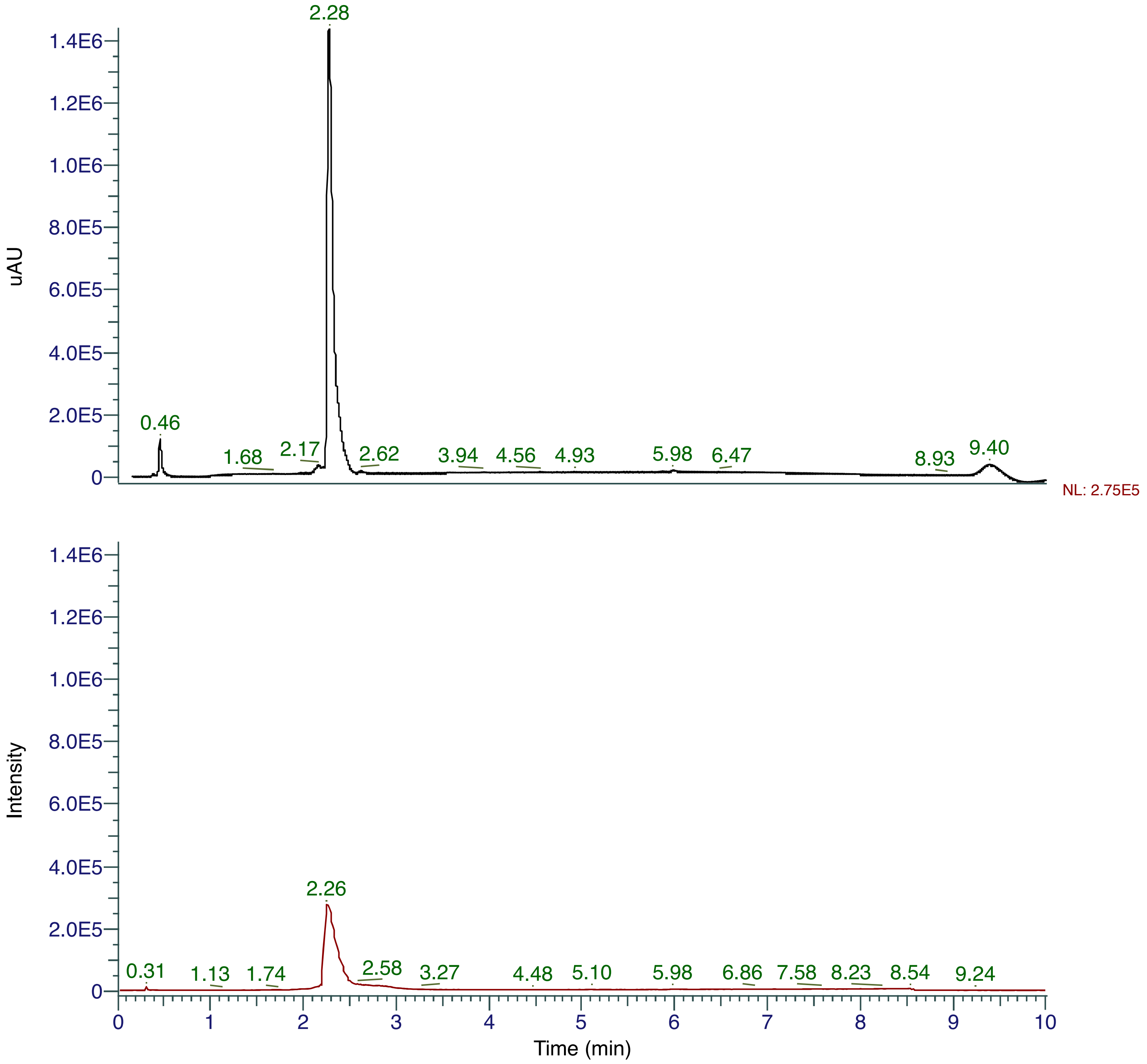

MP_7aaP-Ir #121 RT: 2.26 AV: 1 NL: 8.39E+004
T: ITMS + c ESI Full ms [150.00-2000.00]

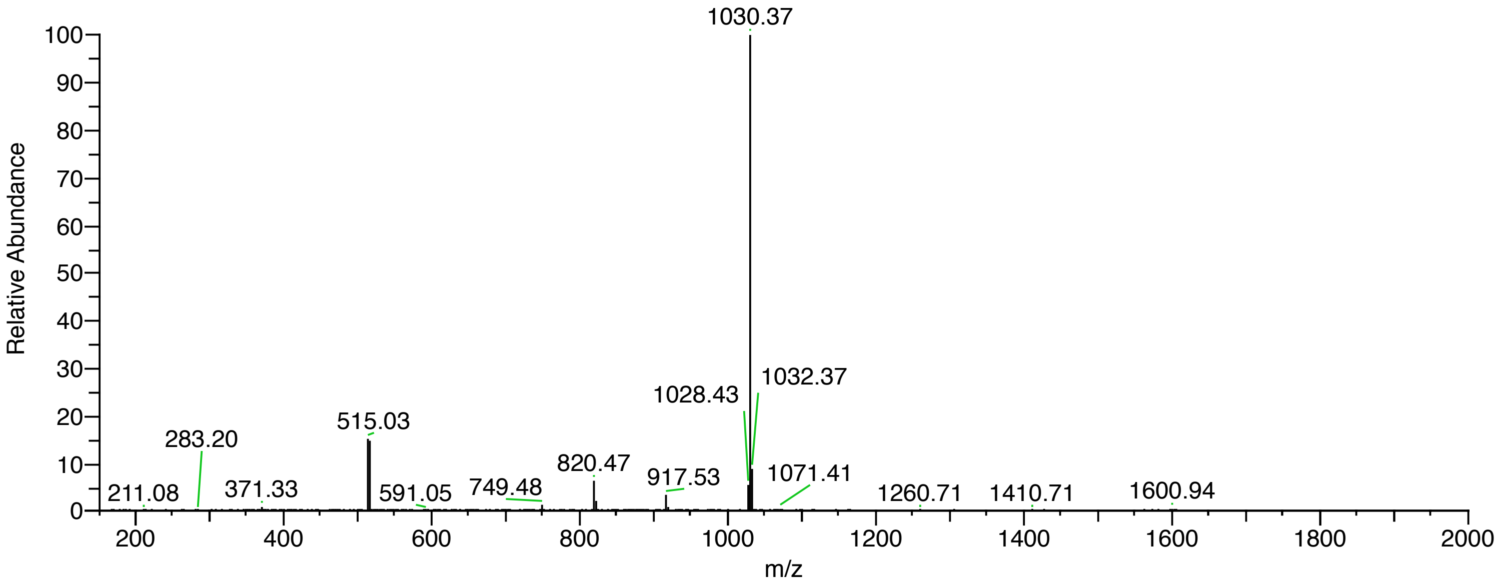

HR-MS 23-apo (ALAḦALA)

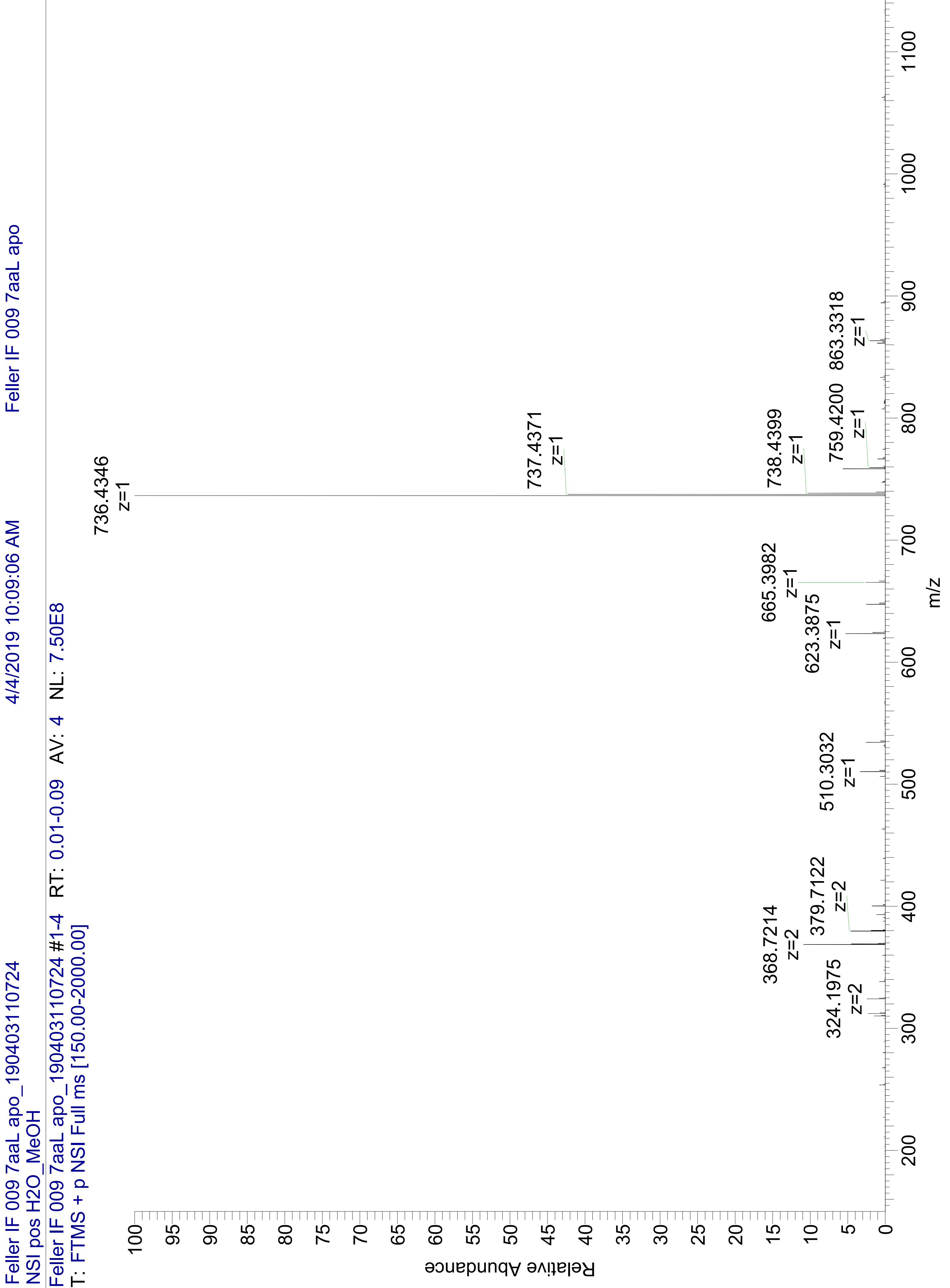

HR-MS 23-Ir (ALAḦALA)

Feller IF 009 7aaL Ir_190403110724 4/4/2019 10:11:14 AM Feller IF 009 7aaL Ir
NSI pos H2O_MeOH

Feller IF 009 7aaL Ir_190403110724 #1-3 RT: 0.00-0.05 AV: 3 NL: 3.01E8
T: FTMS + p NSI Full ms [150.00-2000.00]

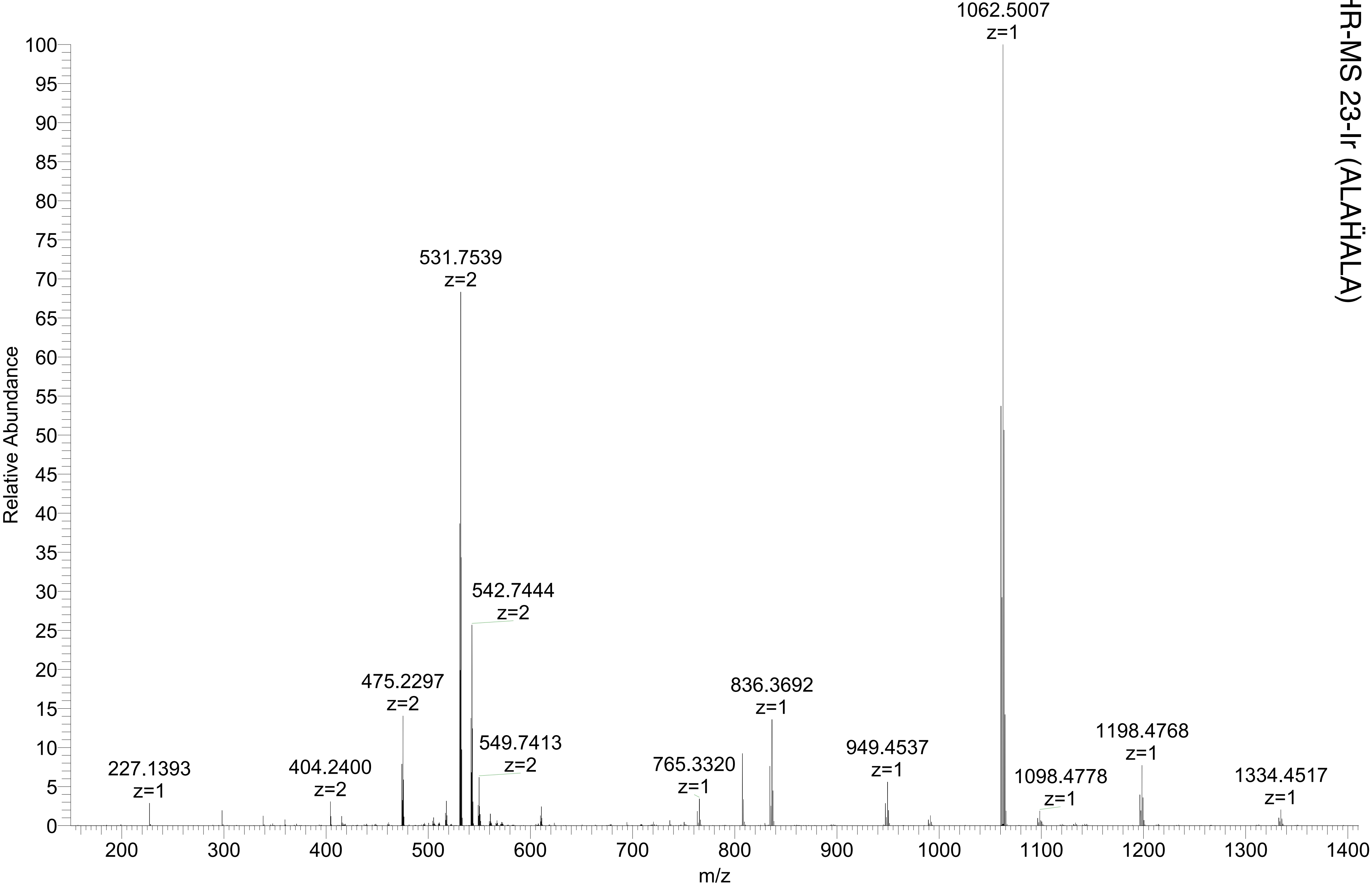

F:\Xcalibur\Data\Zamolo\MATTEO\MP_7aaL-apo.raw 25.04.2019 17:35:51

RT :0.00-10.00 GNL: 2.72E6 TIC MS MP_7aaL-apo

LC-MS 23-apo (ALAḦALA)

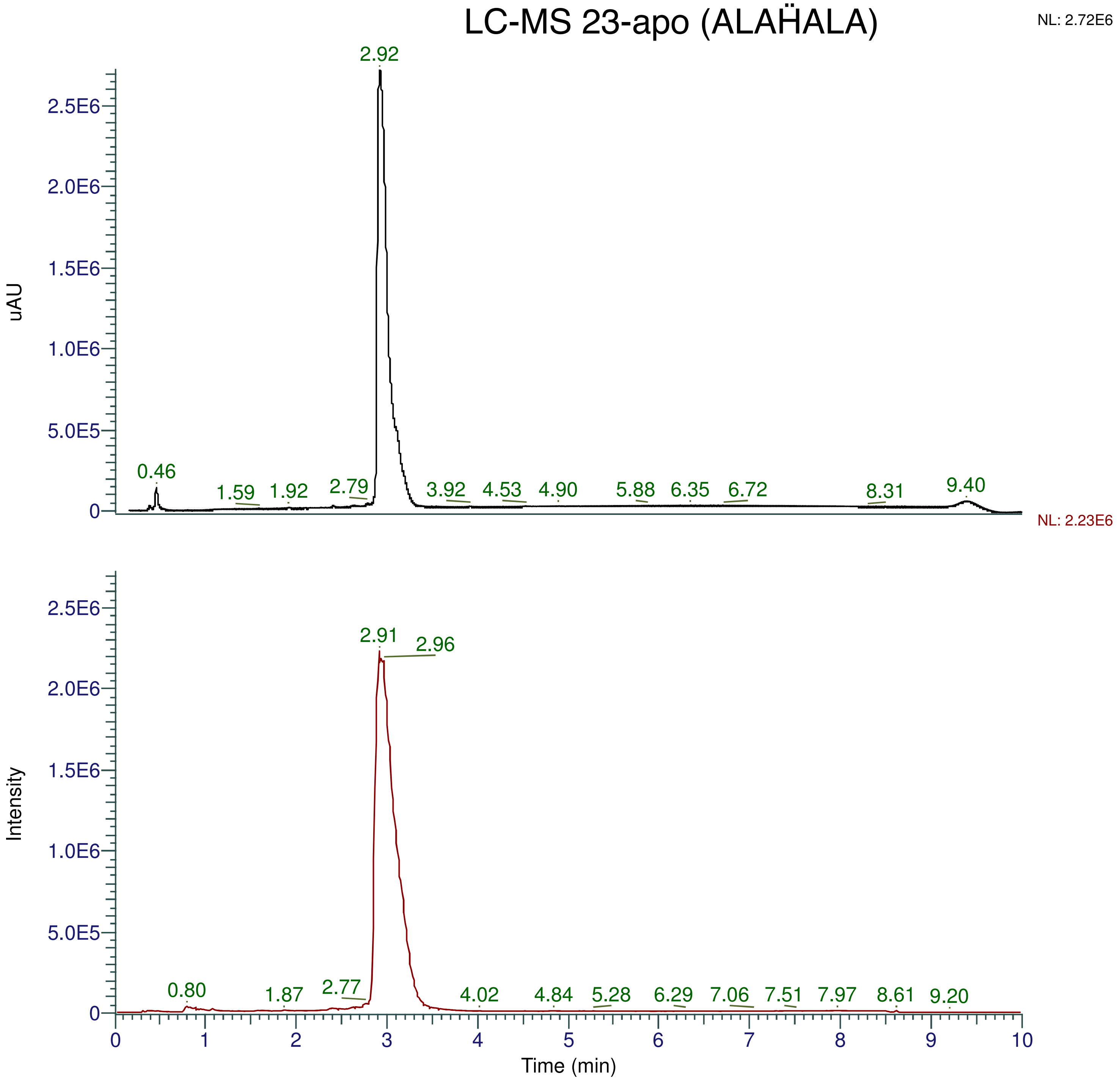

MP_7aaL-apo #171 RT: 2.95 AV: 1 NL: 1.42E+006
T: ITMS + c ESI Full ms [150.00-2000.00]

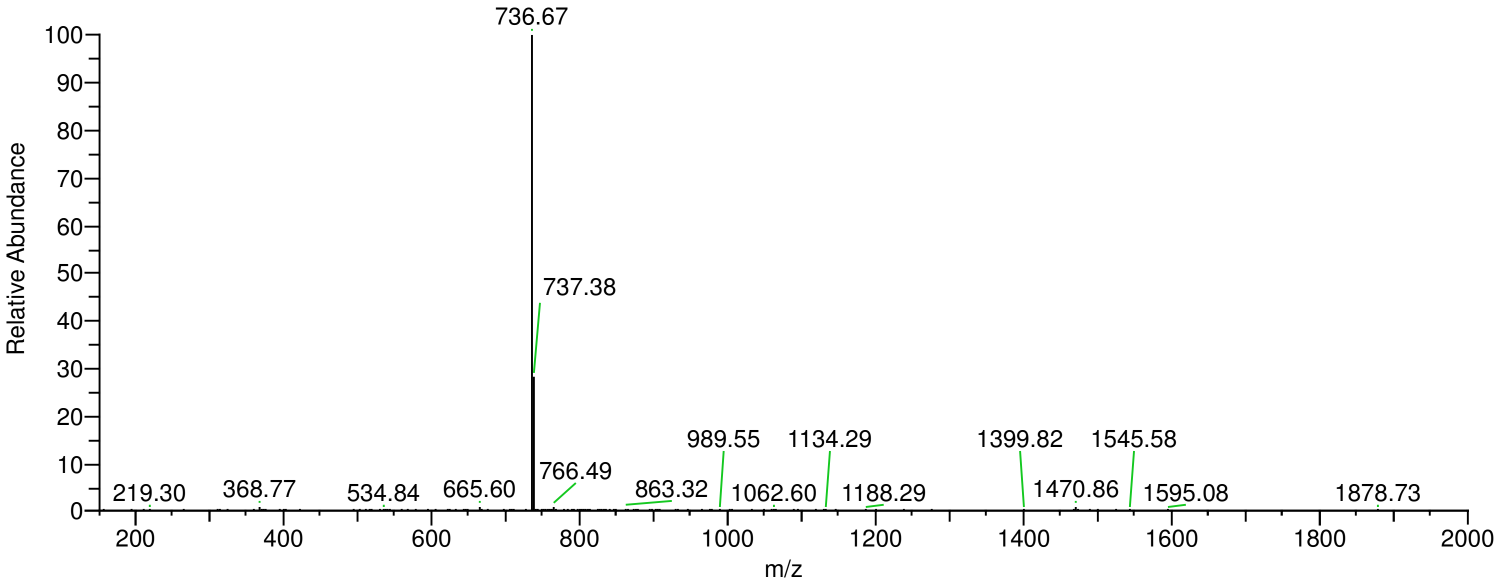

F:\Xcalibur\Data\Zamolo\MATTEO\MP_7aaL-Ir.raw 25.04.2019 17:36:59

RT :0.00-10.00 GNL: 1.06E6 TIC MS MP_7aaL-Ir

LC-MS 23-Ir (ALAḦALA)

NL: 1.06E6

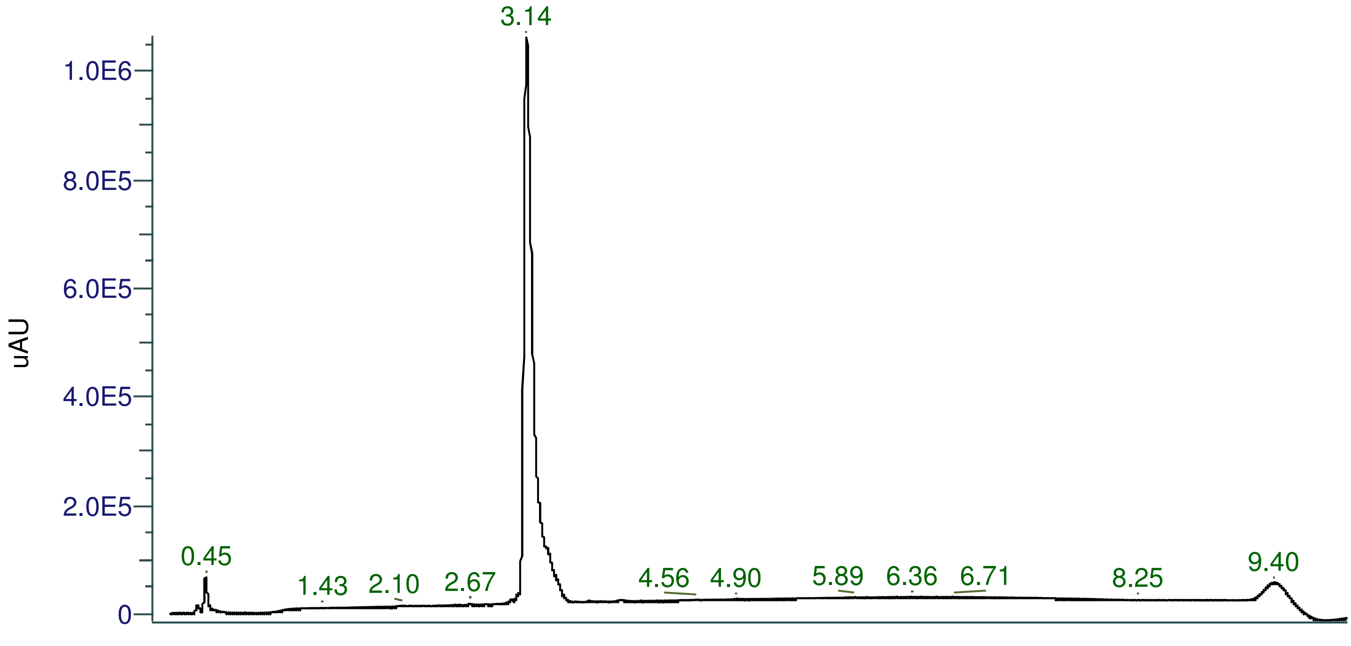

NL: 6.85E5

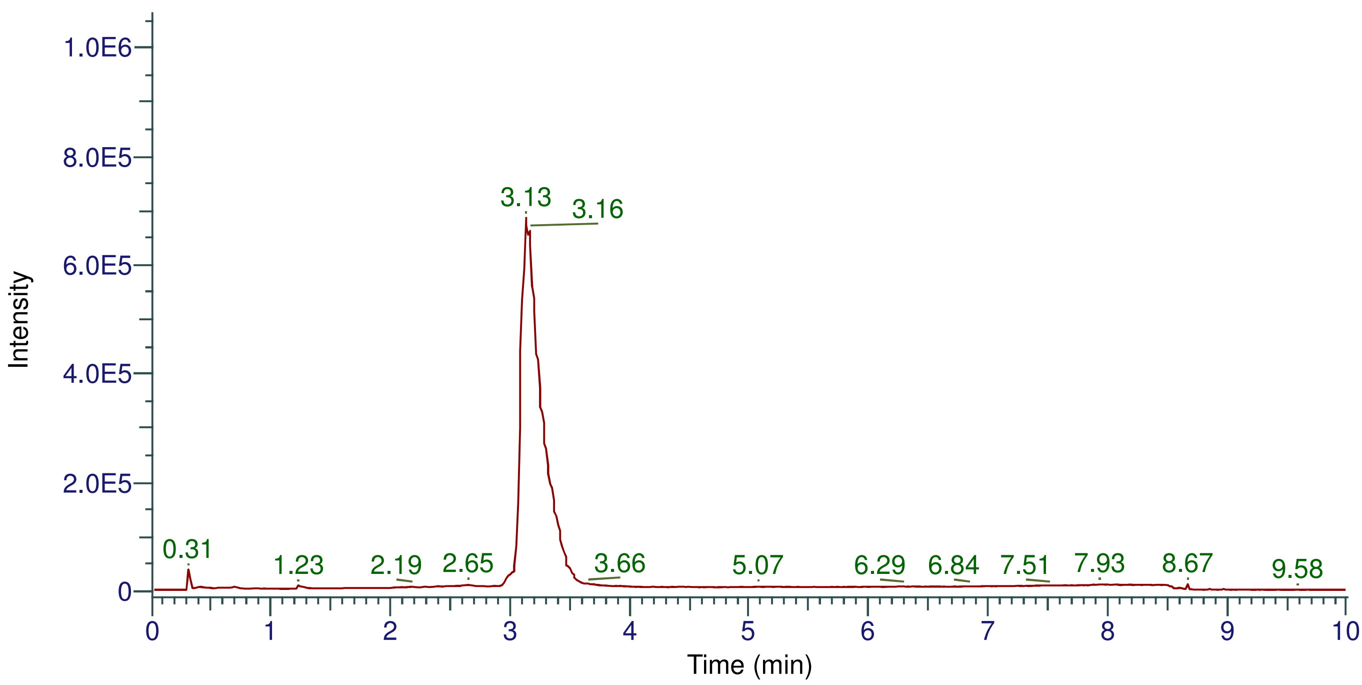

MP_7aaL-Ir #170 RT: 3.14 AV: 1 NL: 2.05E+005
T: ITMS + c ESI Full ms [150.00-2000.00]

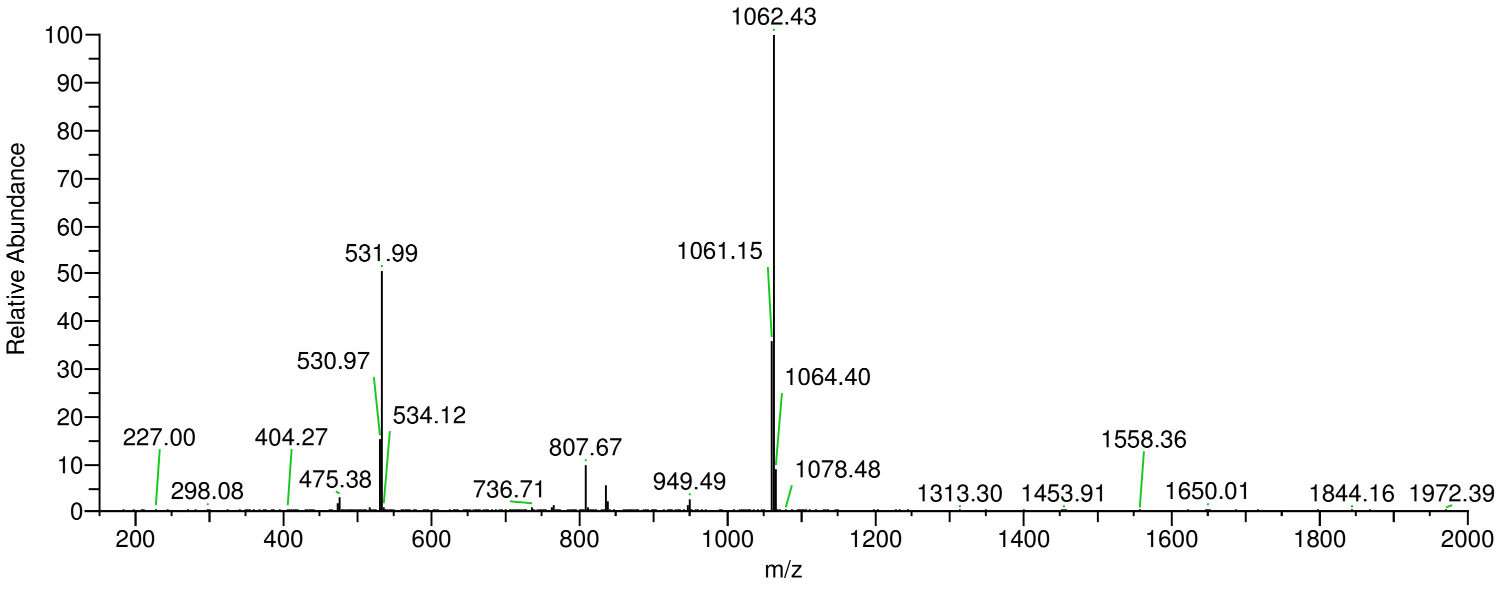

Feller IF 010 7aal apo_190403110724 4/4/2019 10:13:37 AM Feller IF 010 7aal apo
NSI pos H2O_MeOH

Feller IF 010 7aal apo_190403110724 #1 RT: 0.01 AV: 1 NL: 7.84E8
T: FTMS + p NSI Full ms [150.00-2000.00]

HR-MS 24-apo (AIAḦAIA)

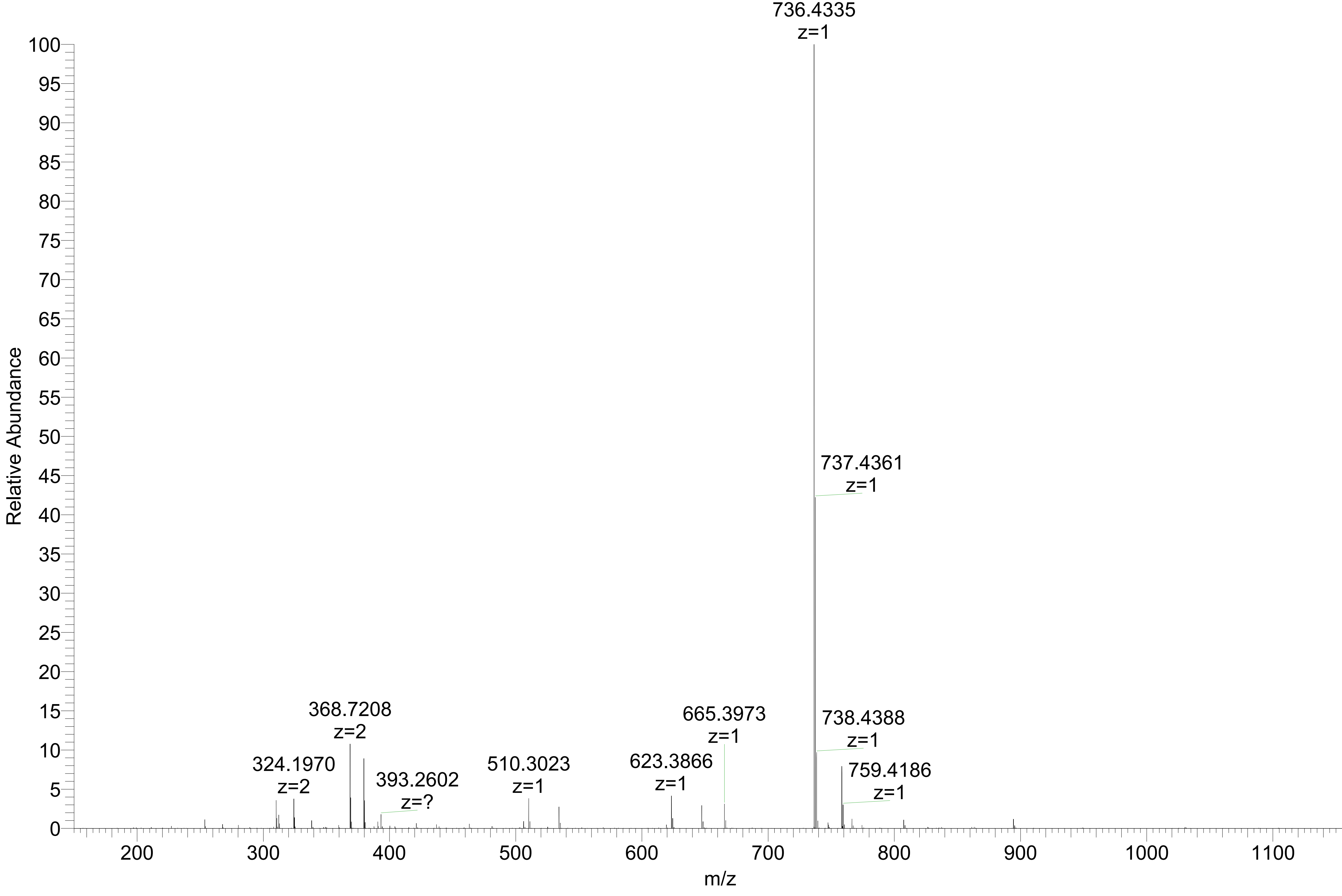

Feller IF 010 7aal Ir_190403110724 4/4/2019 10:15:36 AM Feller IF 010 7aal Ir
NSI pos H2O_MeOH

Feller IF 010 7aal Ir_190403110724 #1-3 RT: 0.01-0.07 AV: 3 NL: 2.85E8
T: FTMS + p NSI Full ms [150.00-2000.00]

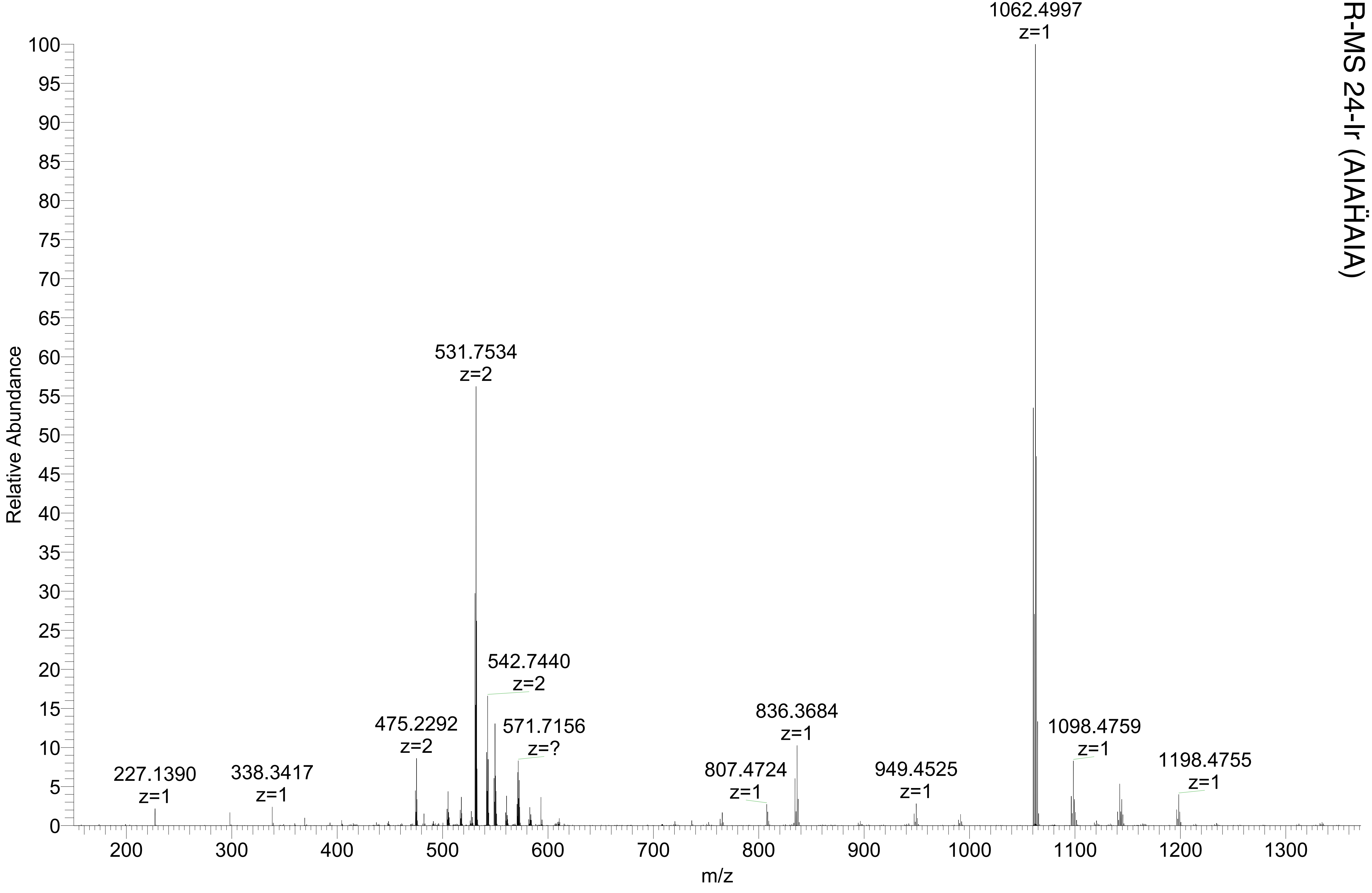

F:\Xcalibur\Data\Zamolo\MATTEO\MP_7aaI-apo.raw 25.04.2019 17:40:06

RT :0.00-10.00 GNL: 1.15E6 TIC MS MP_7aaI-apo

LC-MS 24-apo (AIAḦAIA)

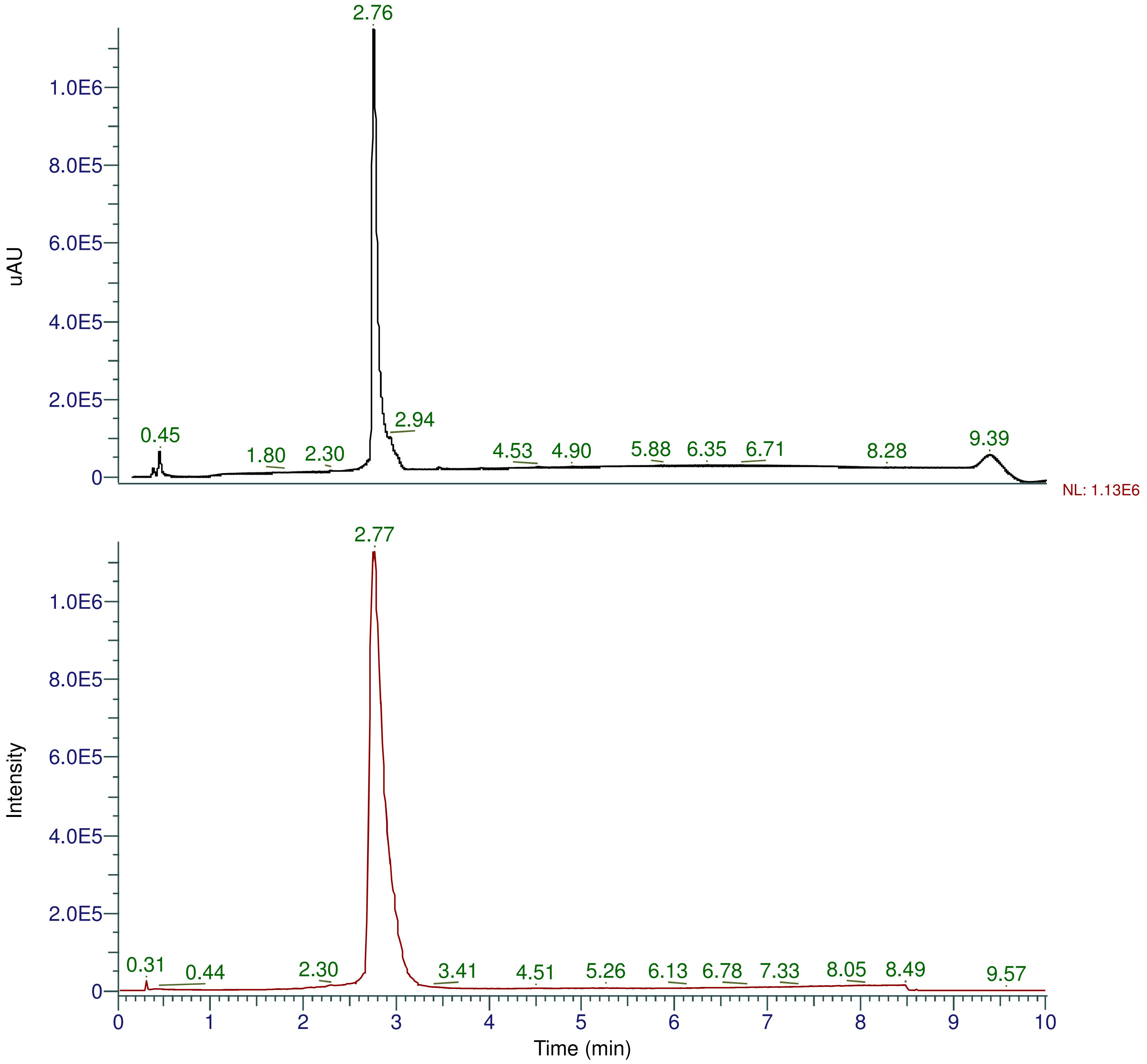

MP_7aaI-apo #152 RT: 2.76 AV: 1 NL: 9.22E+005

T: ITMS + c ESI Full ms [150.00-2000.00]

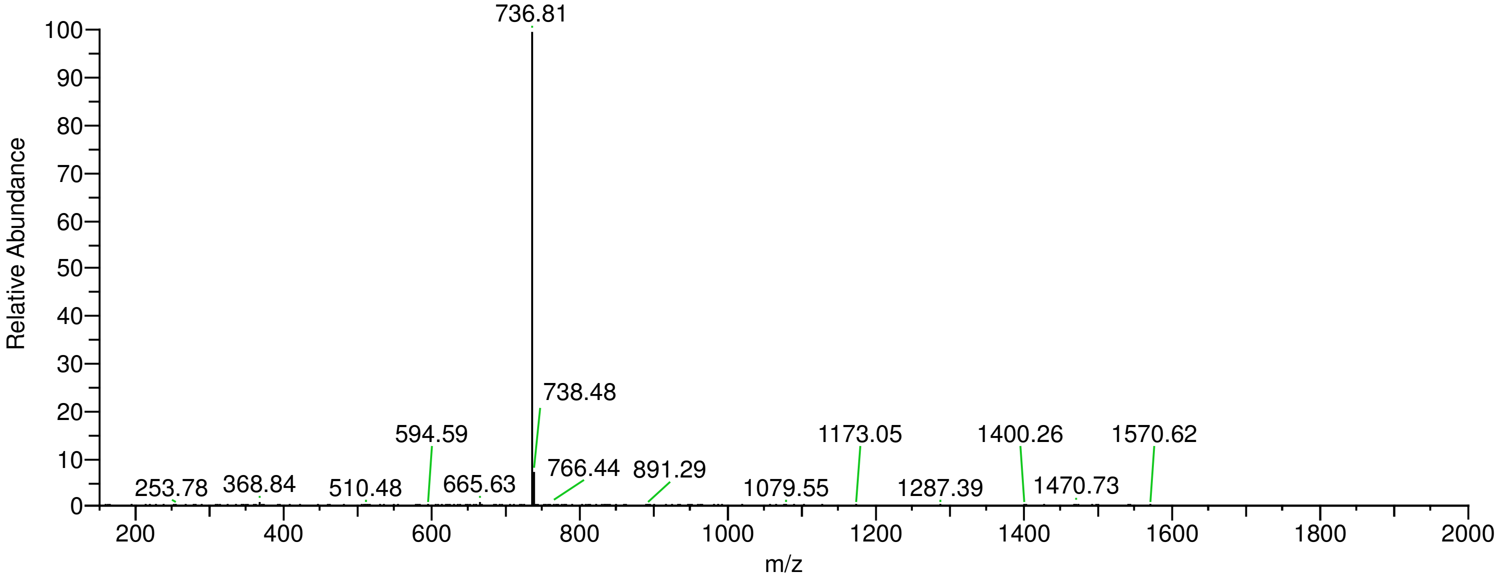

F:\Xcalibur\Data\Zamolo\MATTEO\MP_7aaI-Ir.raw 25.04.2019 17:39:25

RT :0.00-10.00 GNL: 1.98E6 TIC MS MP_7aaI-Ir

LC-MS 24-Ir (AIAḦAIA)

NL: 1.98E6

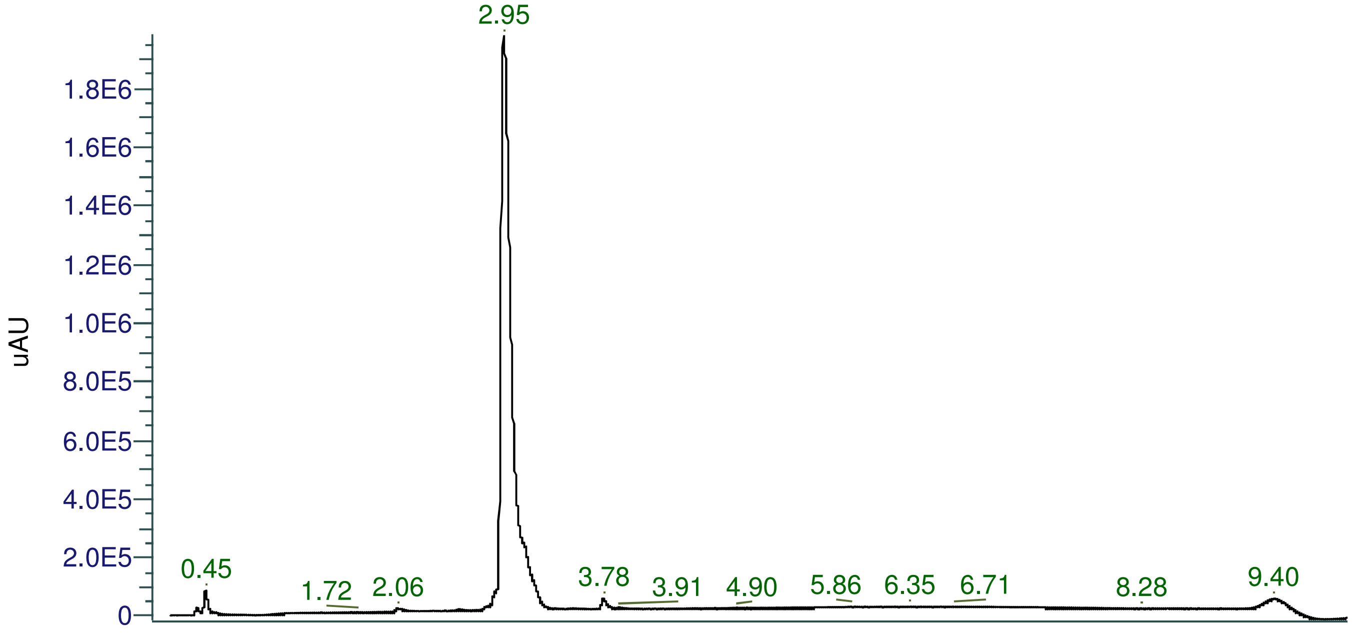

NL: 9.33E5

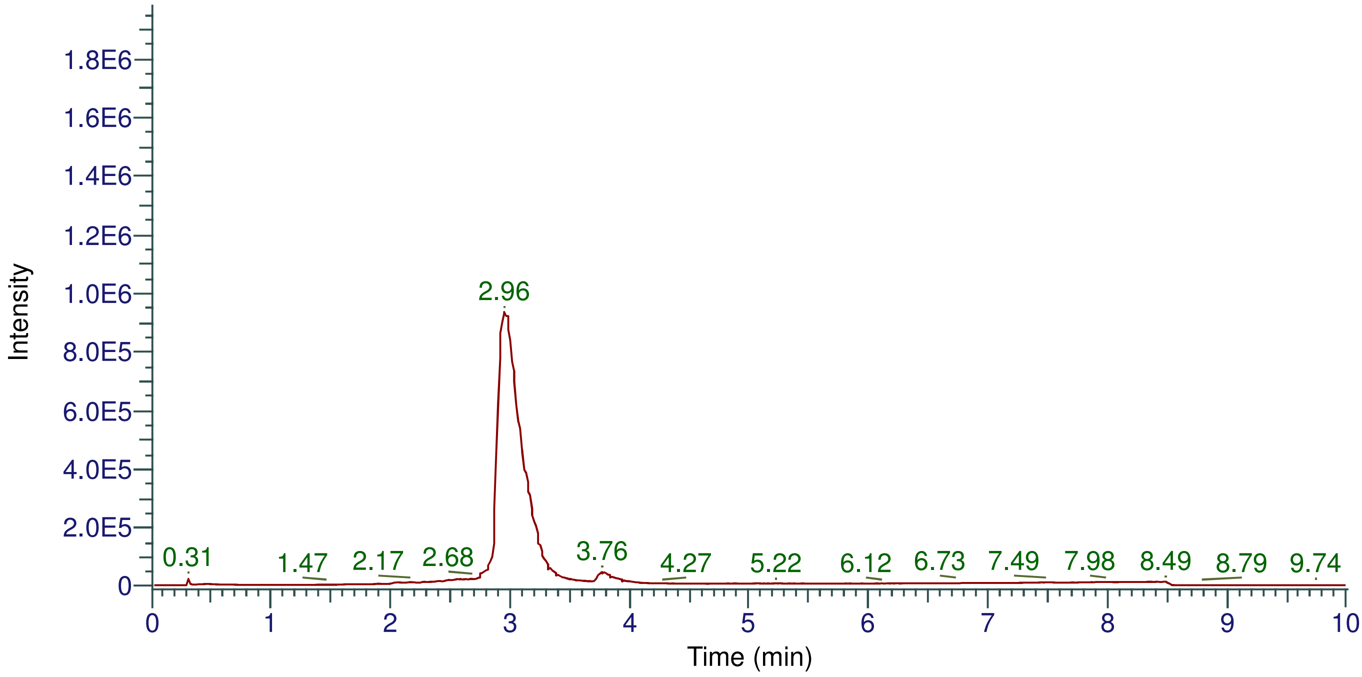

MP_7aaI-Ir #167 RT: 2.97 AV: 1 NL: 3.05E+005

T: ITMS + c ESI Full ms [150.00-2000.00]

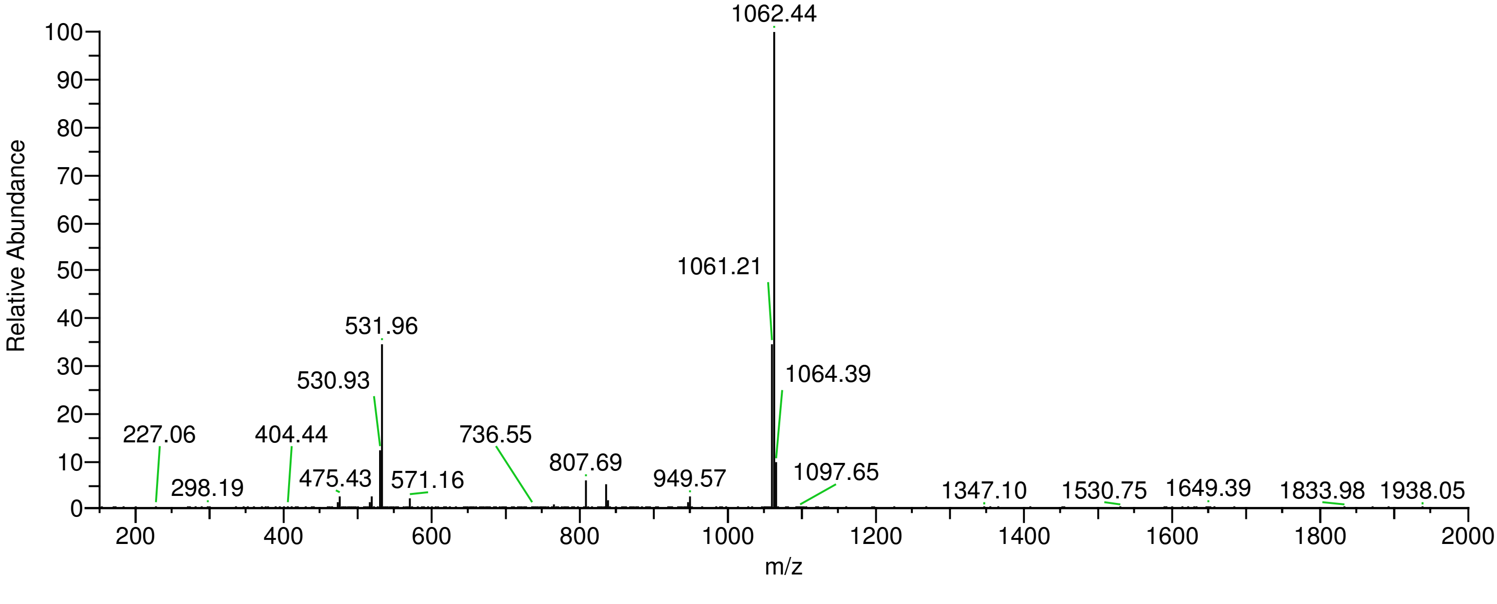

Feller IF 012-7aaF-Apo_190410110135 4/10/2019 3:59:49 PM IF 012-7aaF-Apo
NSI pos H2O/ACN + 1% HFo

Feller IF 012-7aaF-Apo_190410110135 #1-9 RT: 0.02-0.24 AV: 9 NL: 6.76E7
T: FTMS + p NSI Full ms [150.00-2000.00]

HR-MS 25-apo (AFAḦAFA)

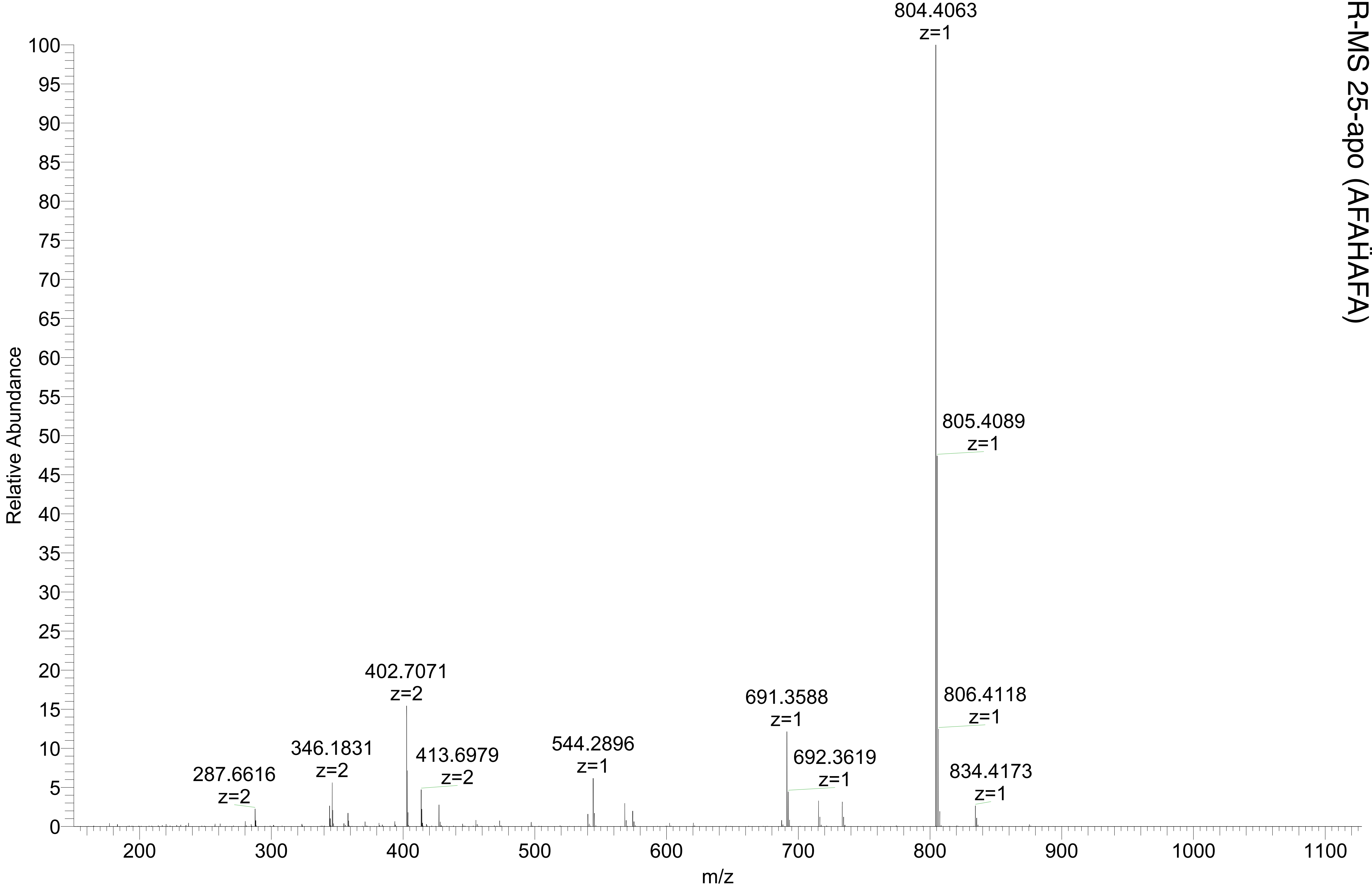

Feller IF 012 7aaF Ir_190410110135 4/12/2019 10:32:46 AM Feller IF 012 7aaF Ir
NSI pos H2O_MeOH

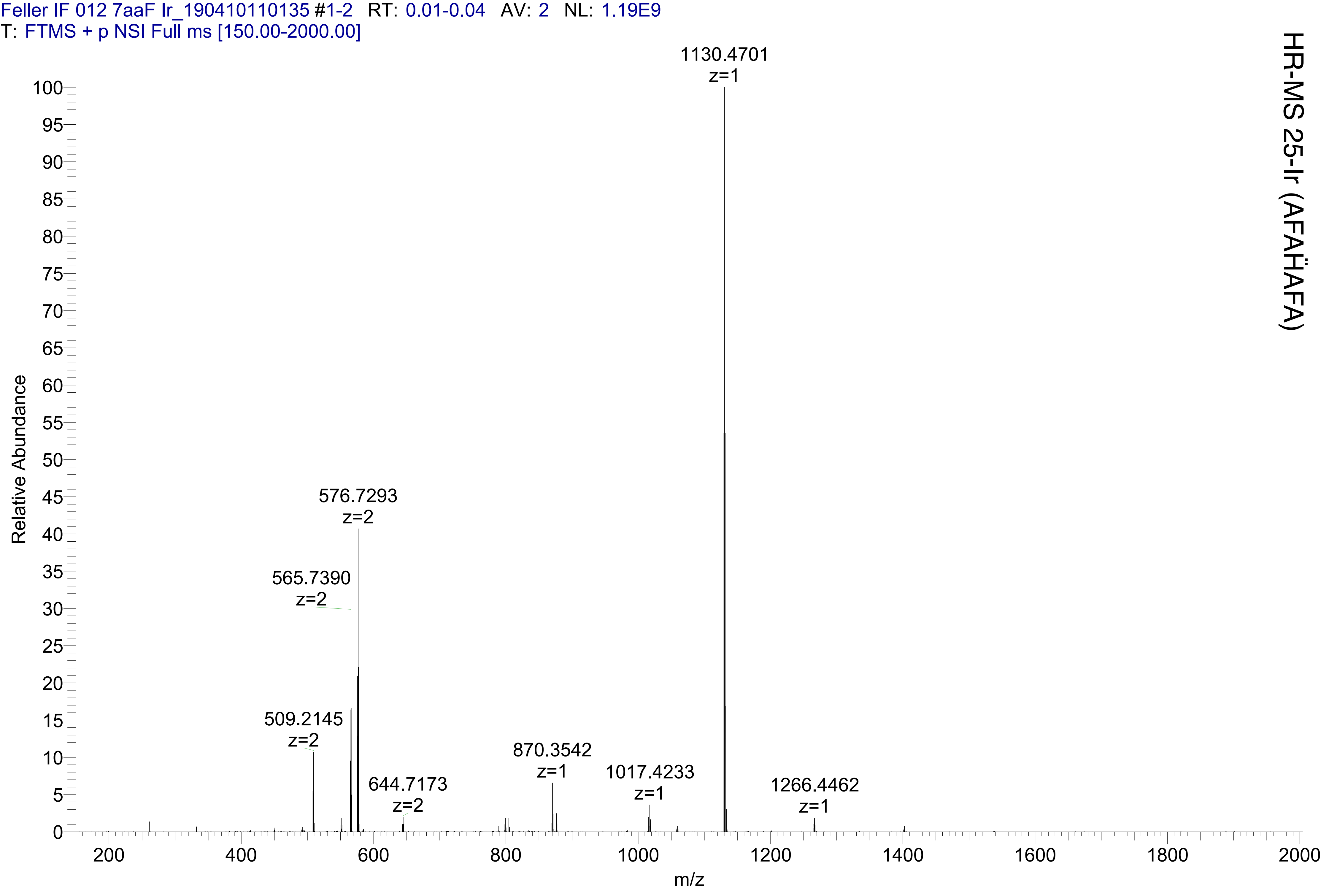

F:\Xcalibur\Data\Zamolo\MATTEO\MP_7aaF-apo.raw 25.04.2019 17:38:35

RT :0.00-10.00 GNL: 2.17E6 TIC MS MP_7aaF-apo

LC-MS 25-apo (AFAḦAFA)

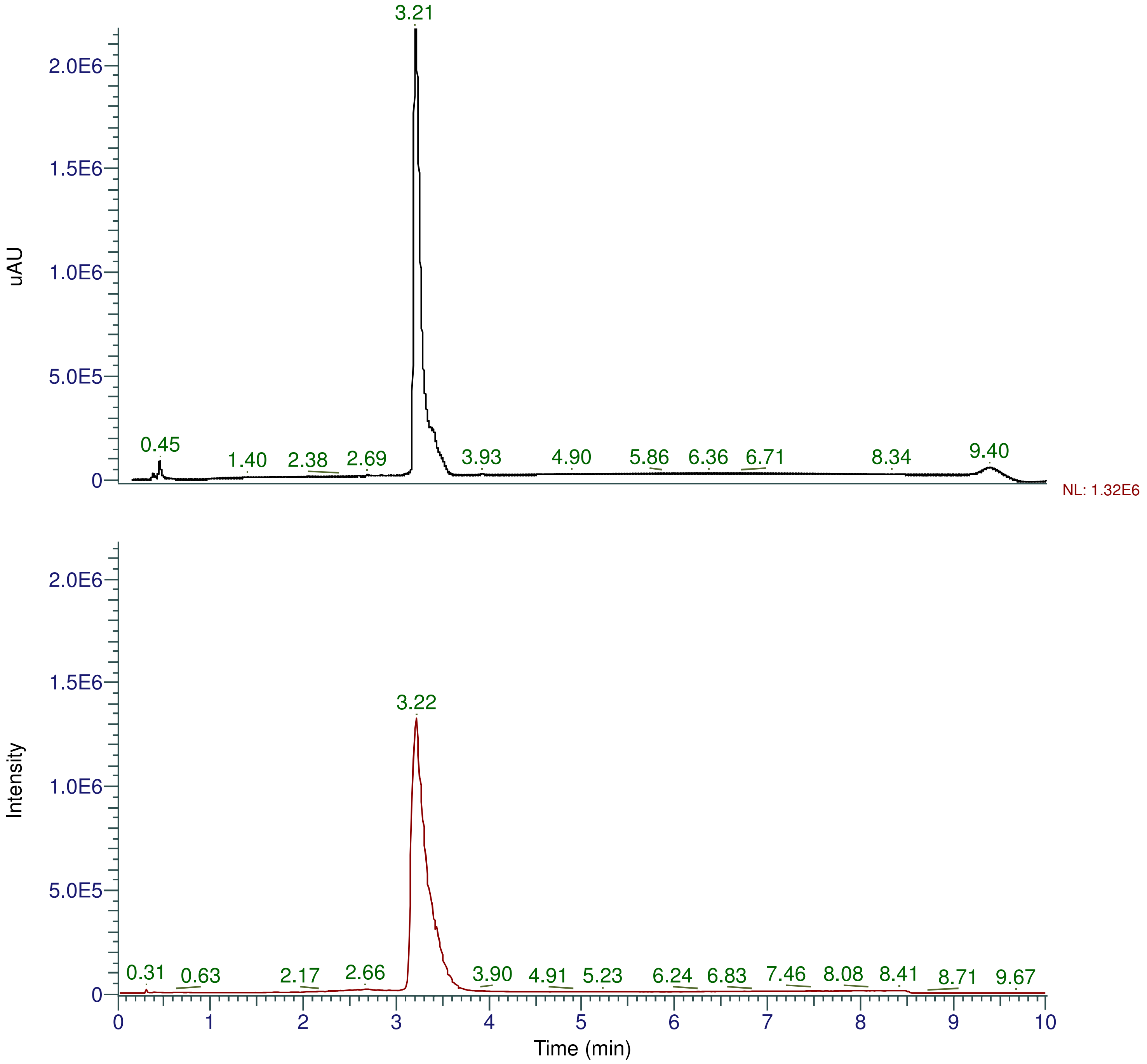

MP_7aaF-apo #178 RT: 3.23 AV: 1 NL: 8.18E+005
T: ITMS + c ESI Full ms [150.00-2000.00]

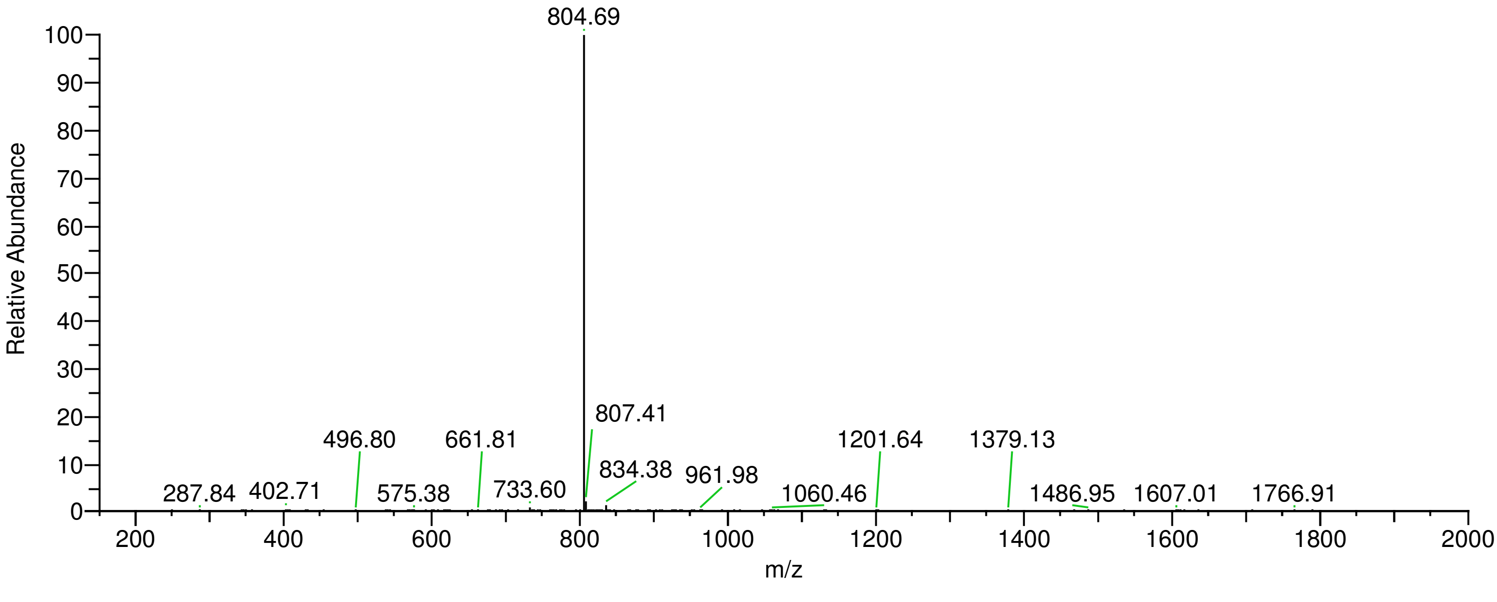

F:\Xcalibur\Data\Zamolo\MATTEO\MP_7aaF-Ir.raw 25.04.2019 17:58:05

RT :0.00-10.00 GNL: 8.08E5 TIC MS MP_7aaF-Ir

LC-MS 25-Ir (AFAḦAFA)

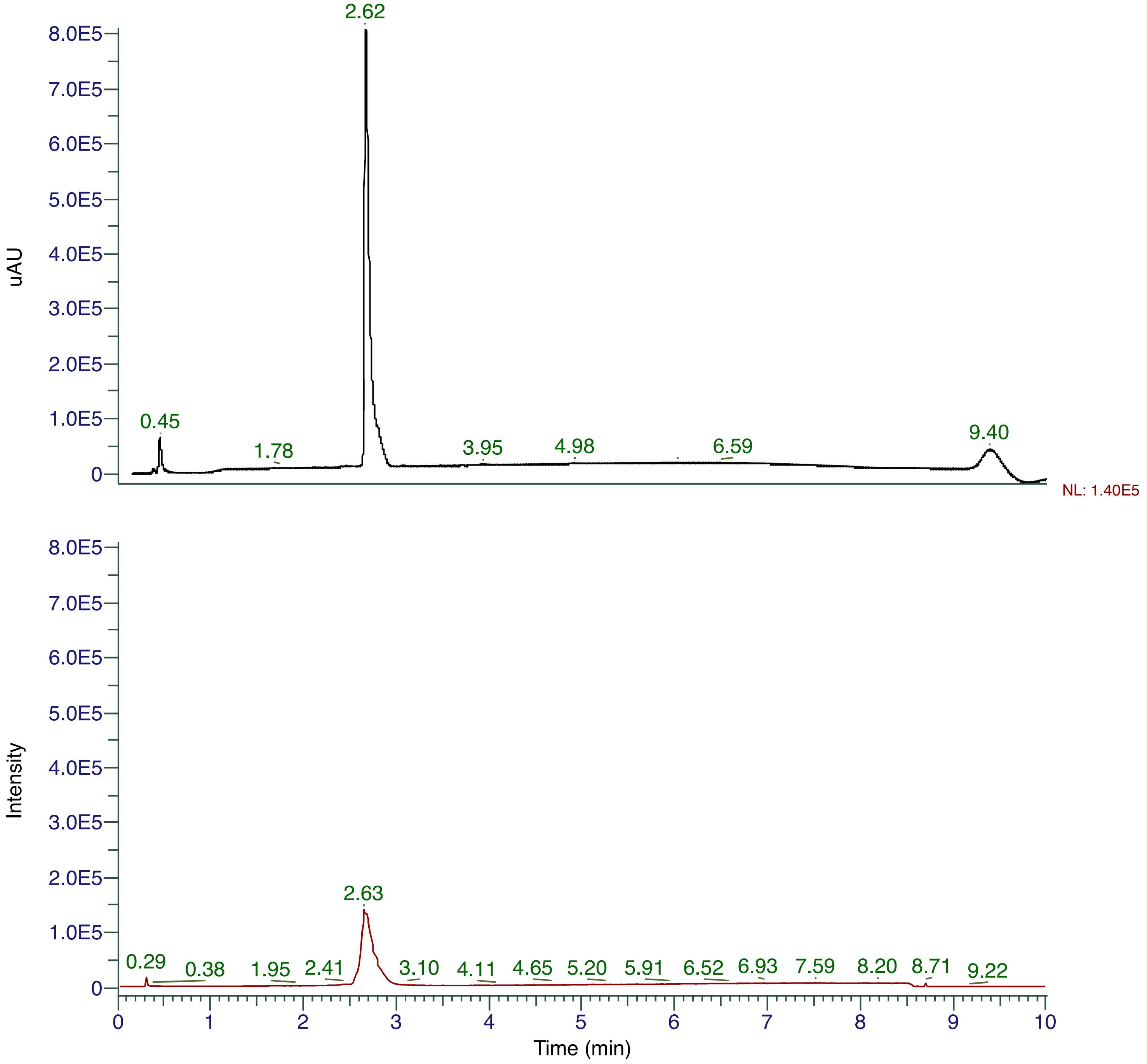

MP_7aaY-Ir #146 RT: 2.71 AV: 1 NL: 2.25E+004
T: ITMS + c ESI Full ms [150.00-2000.00]

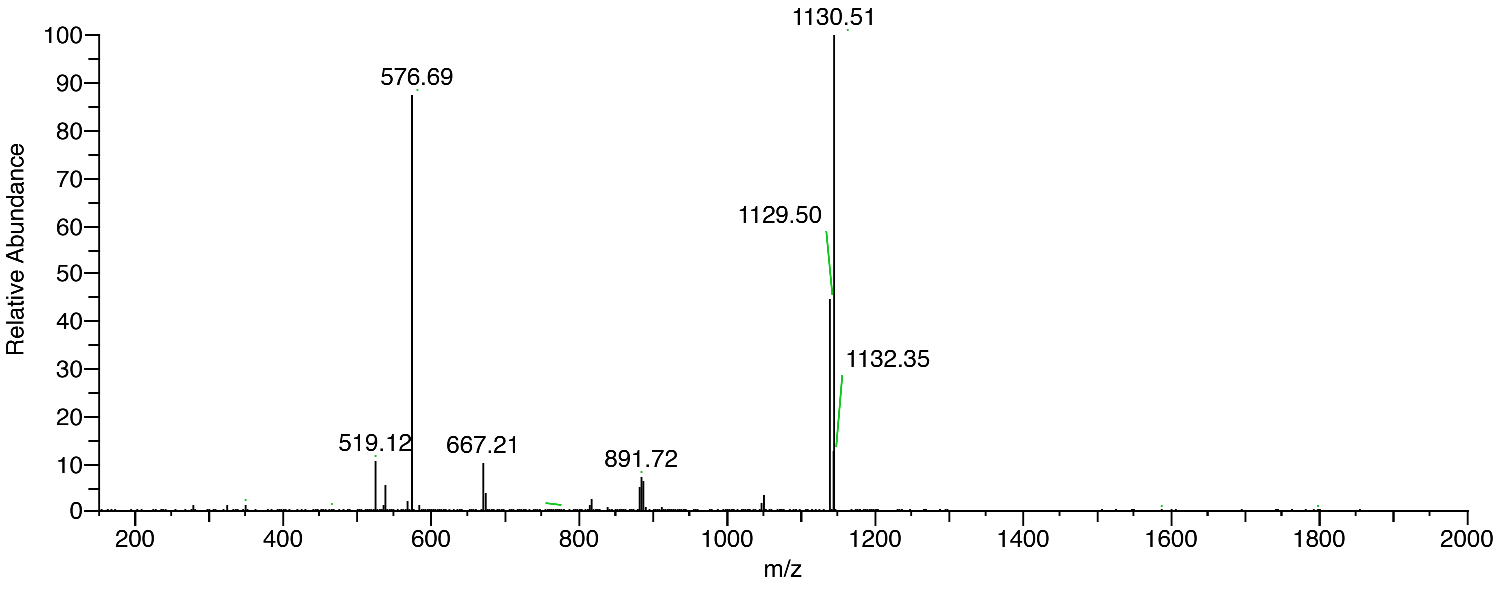

HR-MS 26-apo (AWAḦAWA)

Feller IF 008_7aaW_F1_190401084734 4/1/2019 10:10:49 AM Feller IF 008_7aaW_F1
NSI pos H2O_MeOH

Feller IF 008_7aaW_F1_190401084734 #1-3 RT: 0.01-0.06 AV: 3 NL: 2.71E8
T: FTMS + p NSI Full ms [150.00-2000.00]

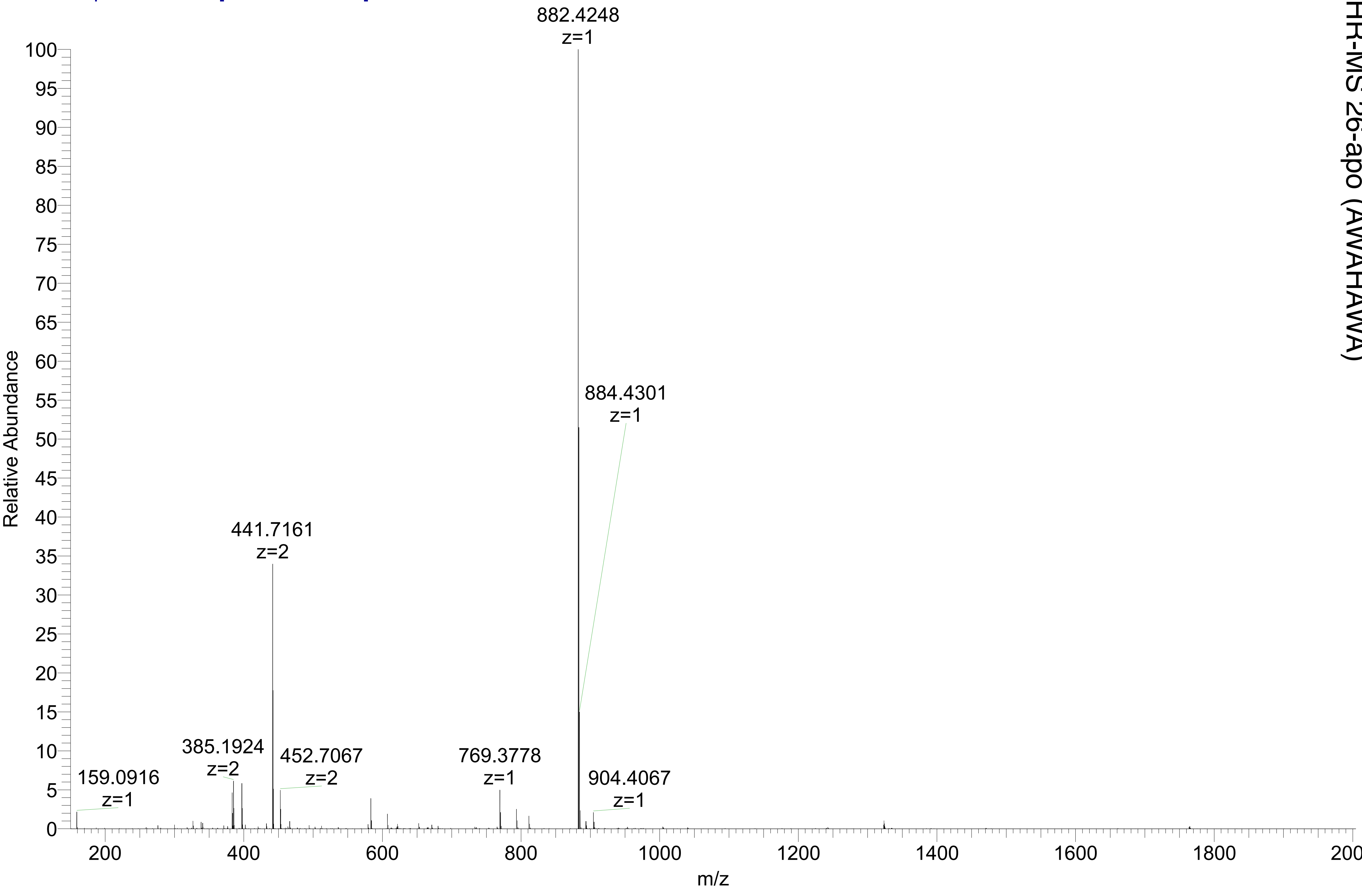

Feller IF 008_7aaW_F4_190405161935 4/8/2019 9:23:03 AM Feller IF 008_7aaW_F4
NSI pos H2O_ACN

Feller IF 008_7aaW_F4_190405161935 #1-4 RT: 0.01-0.09 AV: 4 NL: 5.91E7
T: FTMS + p NSI Full ms [150.00-2000.00]

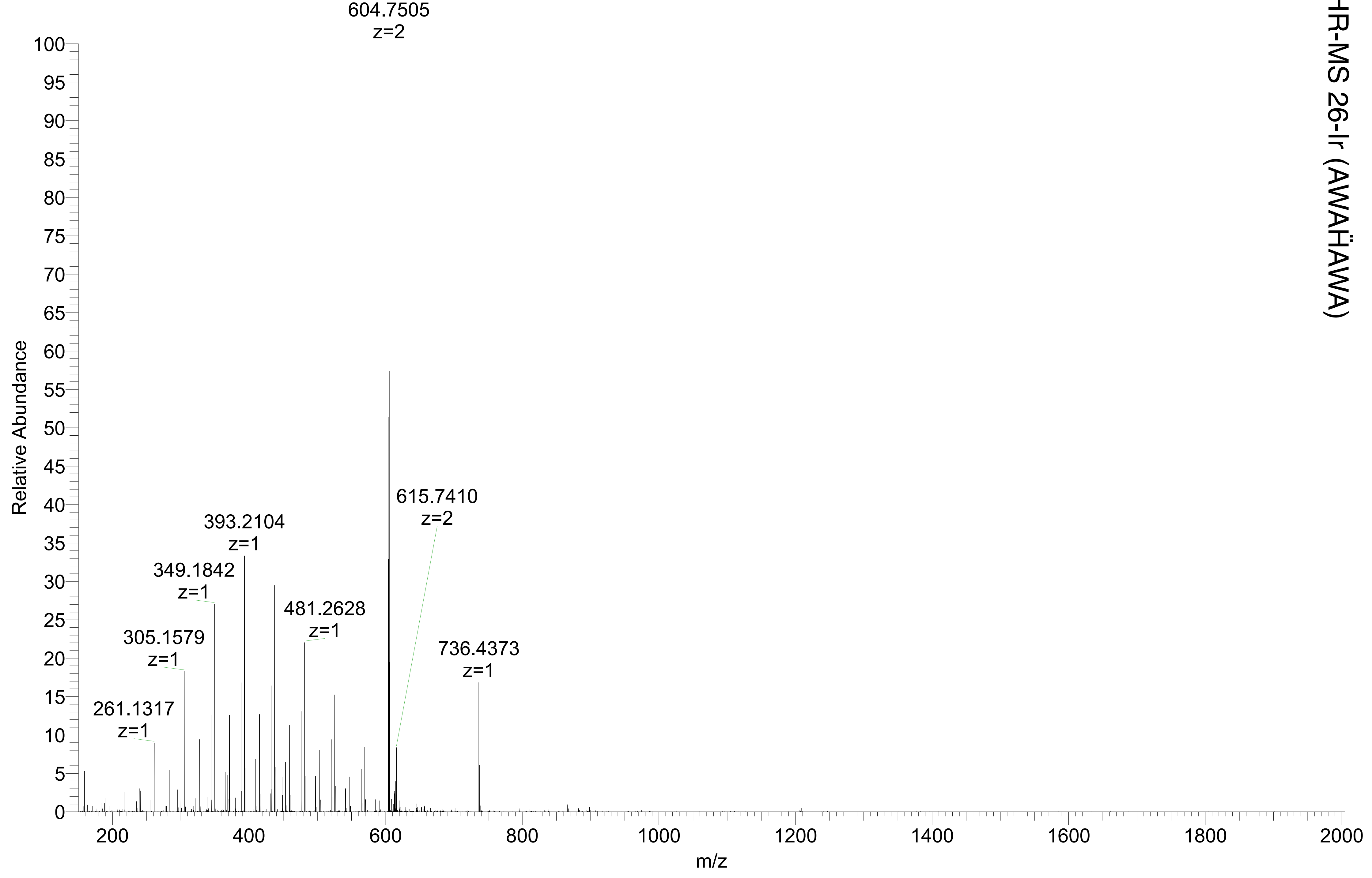

HR-MS 26-Ir (AWAḦAWA)

RT :0.00-10.00 GNL: 4.43E5 TIC MS MP_7aaW-apo

LC-MS 26-apo (AWAḦAWA)

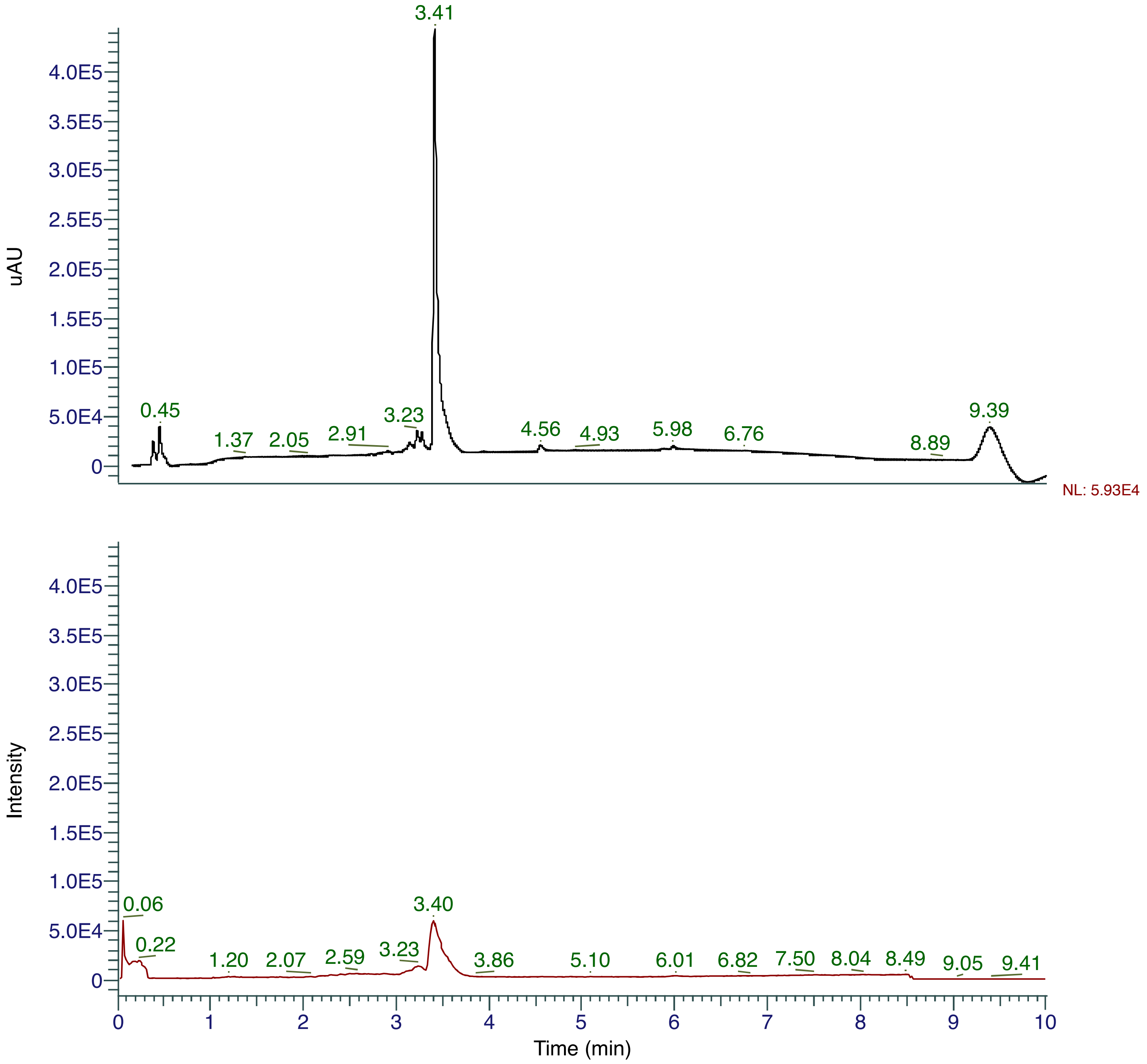

MP_7aaW-apo #178-195 RT: 3.33-3.57 AV: 18 NL: 1.52E4
T: ITMS + c ESI Full ms [150.00-2000.00]

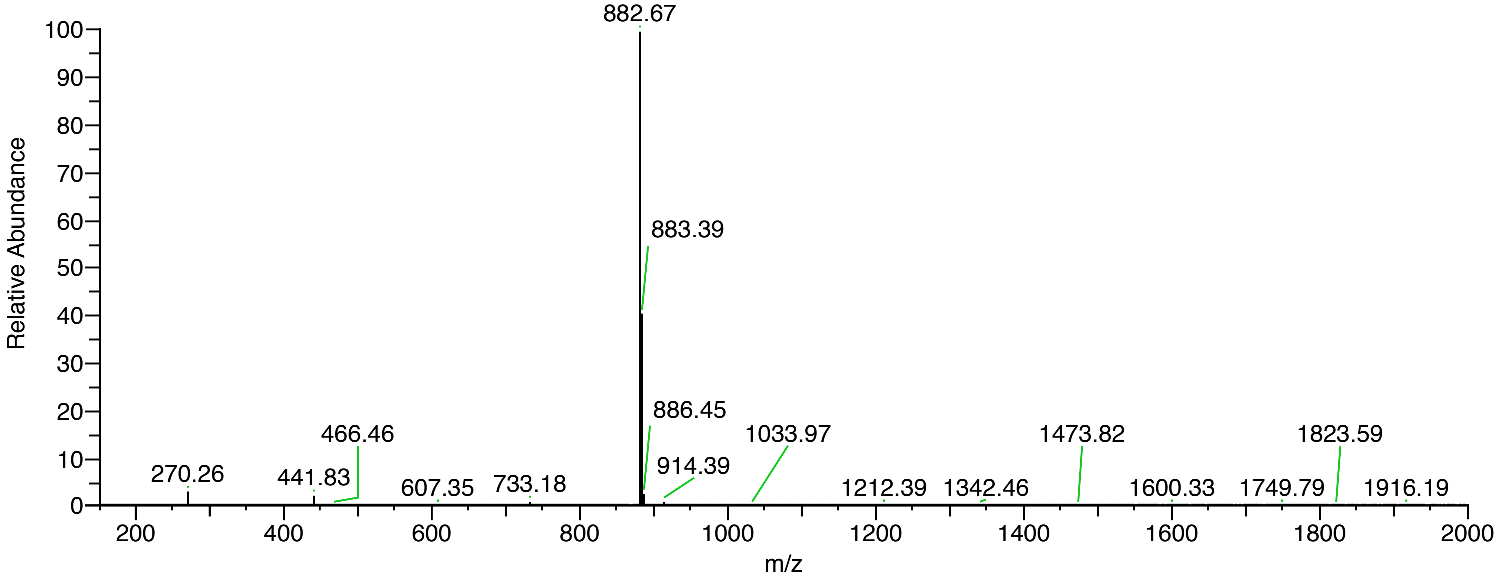

F:\Xcalibur\Data\Zamolo\MATTEO\MP_7aaW-Ir.raw 02.05.2019 08:25:33

RT :0.00-10.00 GNL: 4.72E5 TIC MS MP_7aaW-Ir

LC-MS 26-Ir (AWAḦAWA)

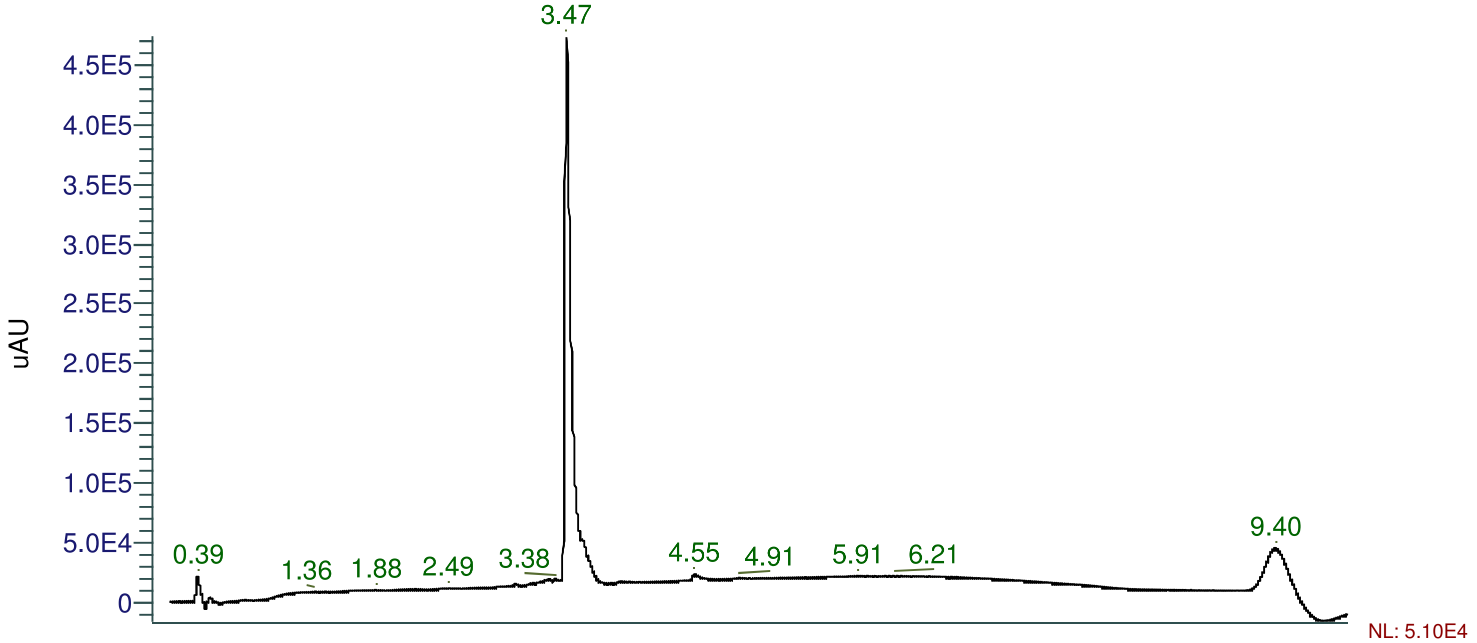

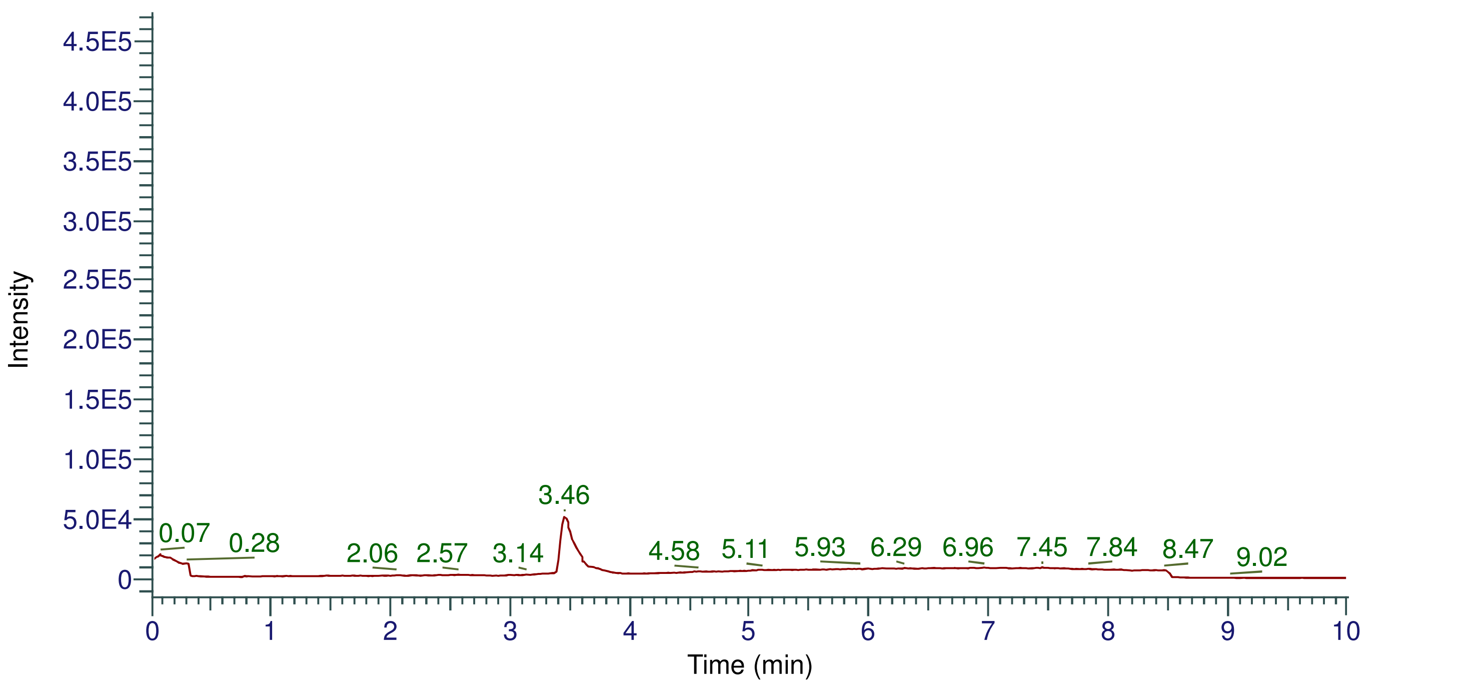

MP_7aaW-Ir #186 RT: 3.47 AV: 1 NL: 1.11E+004
T: ITMS + c ESI Full ms [150.00-2000.00]

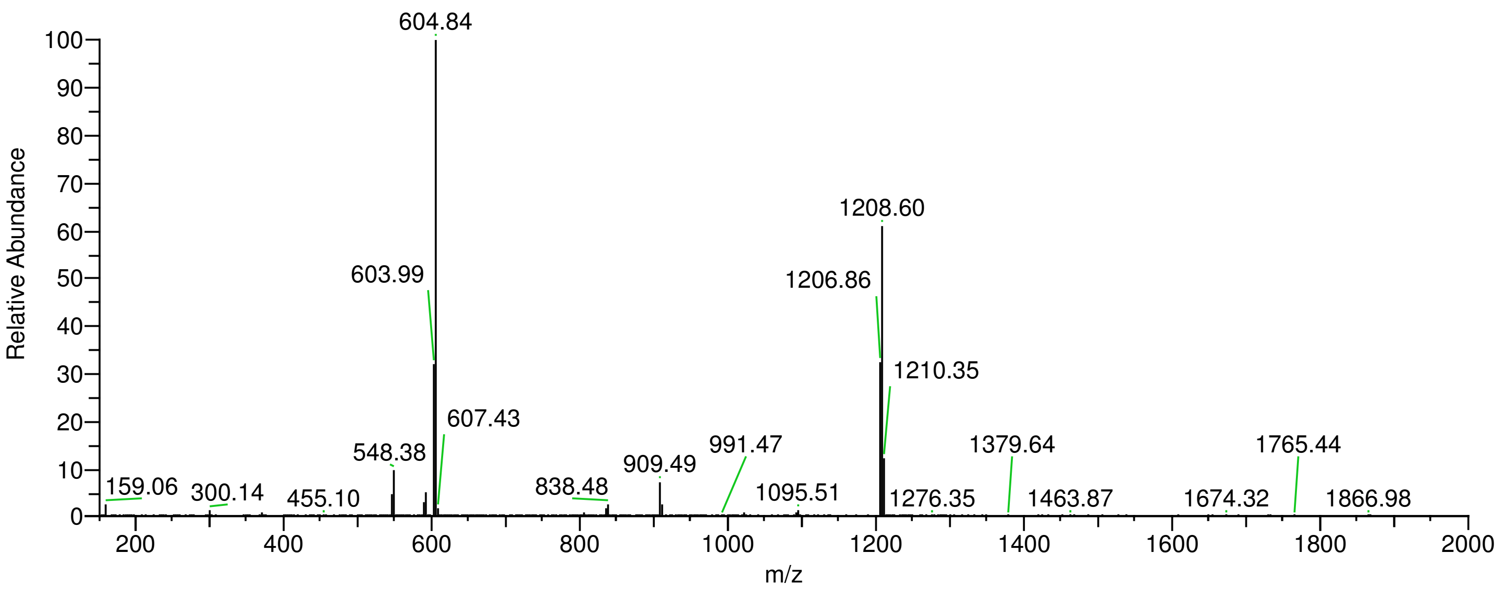

D:\Xcalibur\...\Feller IF 007_7aaT F2 3/27/2019 11:07:45 AM Feller IF 007_7aaT F2
NSI pos H2O_MeOH

Feller IF 007_7aaT F2 #1-3 RT: 0.00-0.06 AV: 3 NL: 3.23E8
T: FTMS + p NSI Full ms [150.00-2000.00]

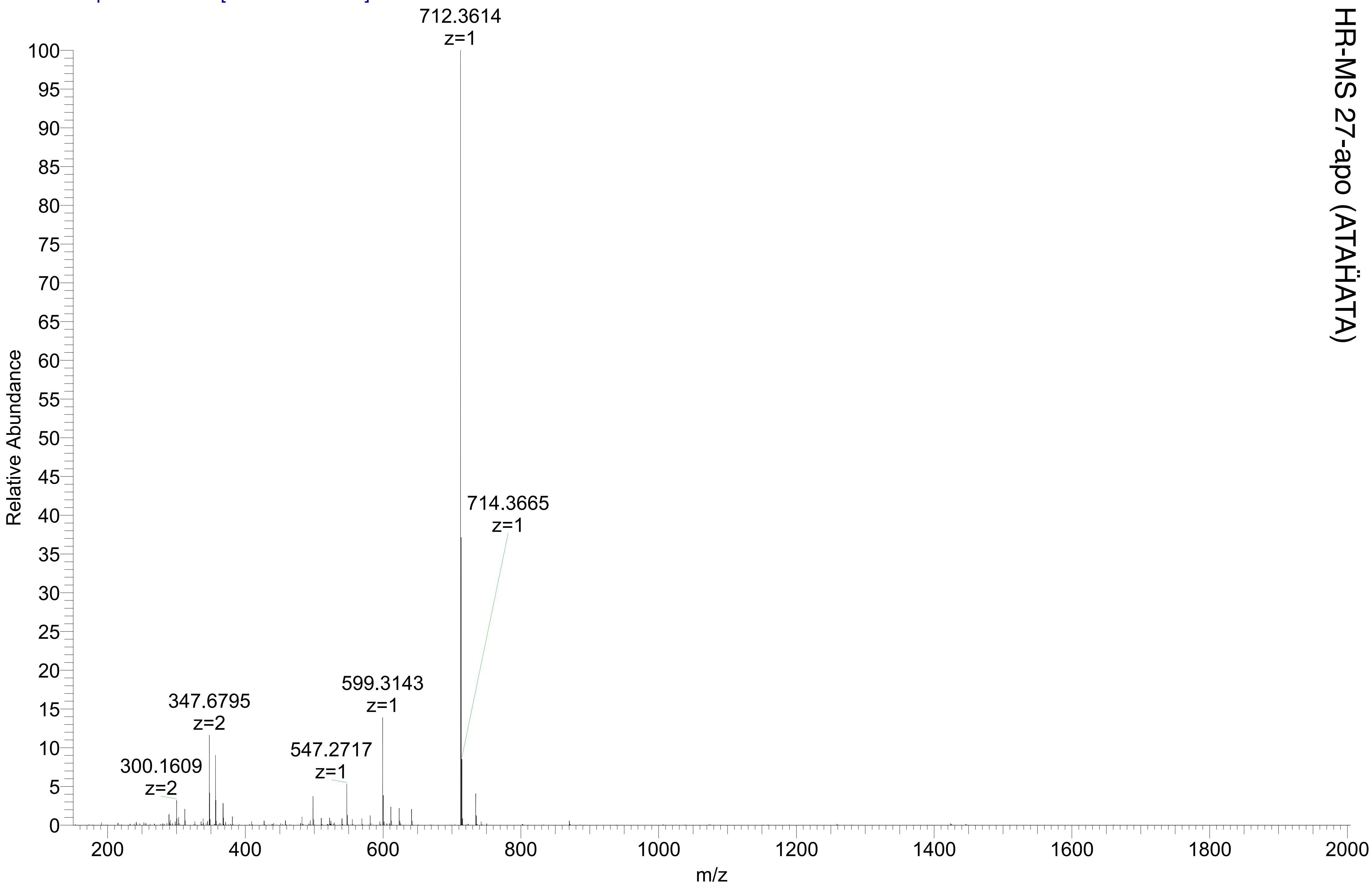

HR-MS 27-apo (ATAӒATA)

D:\Xcalibur\...\Feller IF 007_7aaT F3 3/27/2019 11:09:49 AM Feller IF 007_7aaT F3
NSI pos H2O_MeOH

Feller IF 007_7aaT F3 #1-2 RT: 0.00-0.03 AV: 2 NL: 1.39E8
T: FTMS + p NSI Full ms [150.00-2000.00]

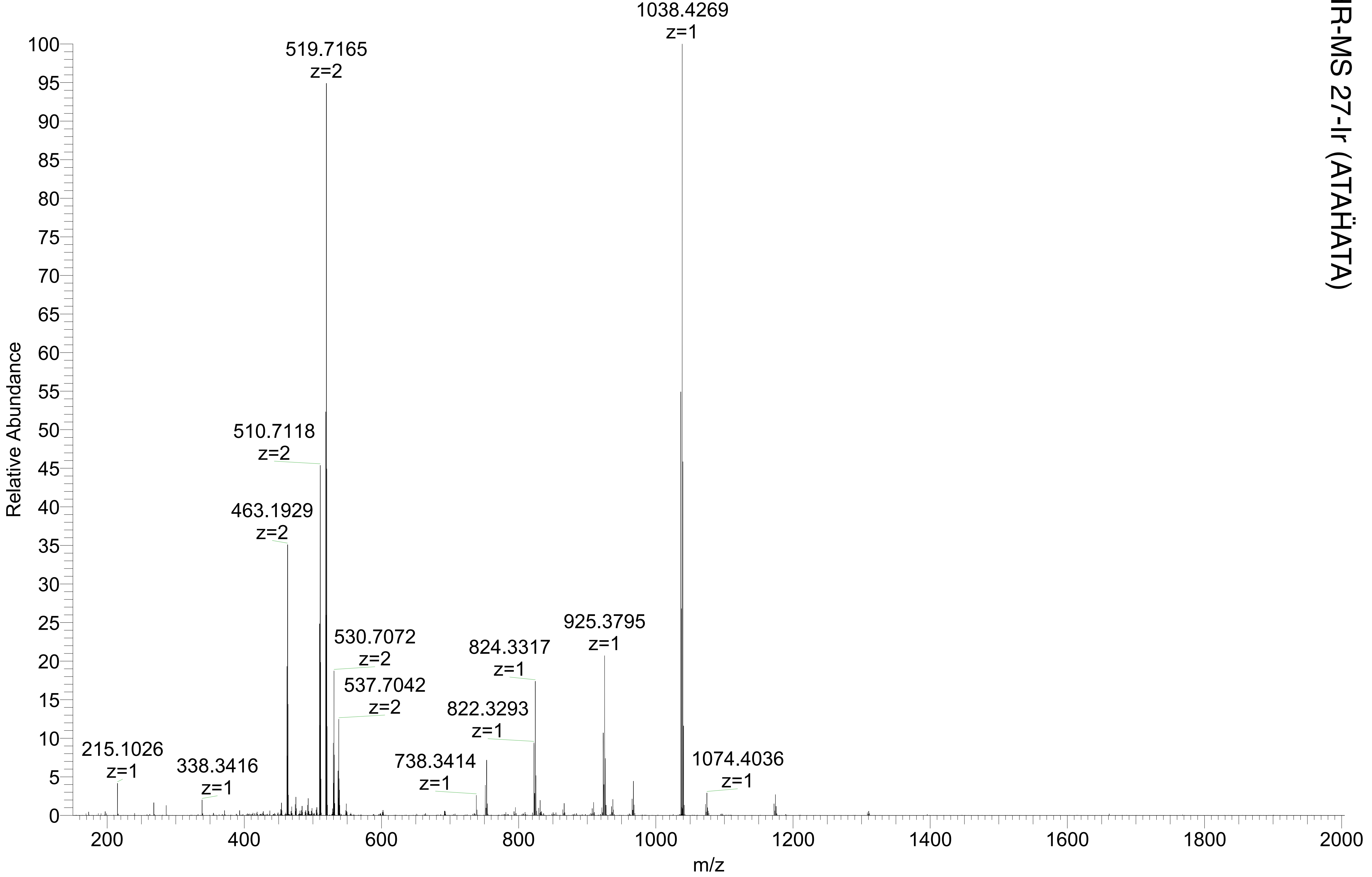

F:\Xcalibur\Data\Zamolo\MATTEO\MP_7aaT-apo.raw 25.04.2019 17:43:38

RT :0.00-10.00 GNL: 2.17E6 TIC MS MP_7aaT-apo

LC-MS 27-apo (ATAḦATA)

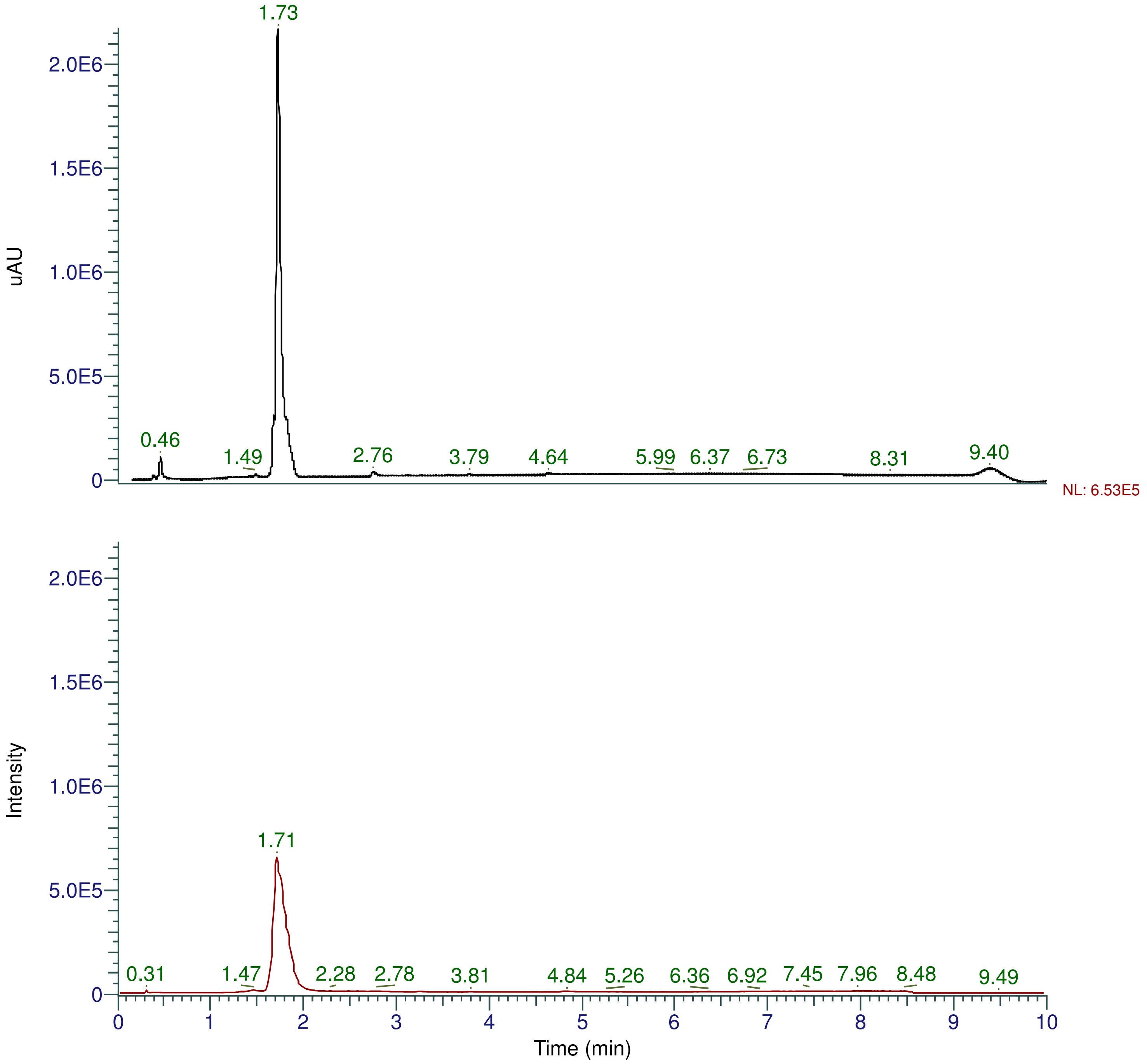

MP_7aaT-apo #95 RT: 1.73 AV: 1 NL: 5.41E+005

T: ITMS + c ESI Full ms [150.00-2000.00]

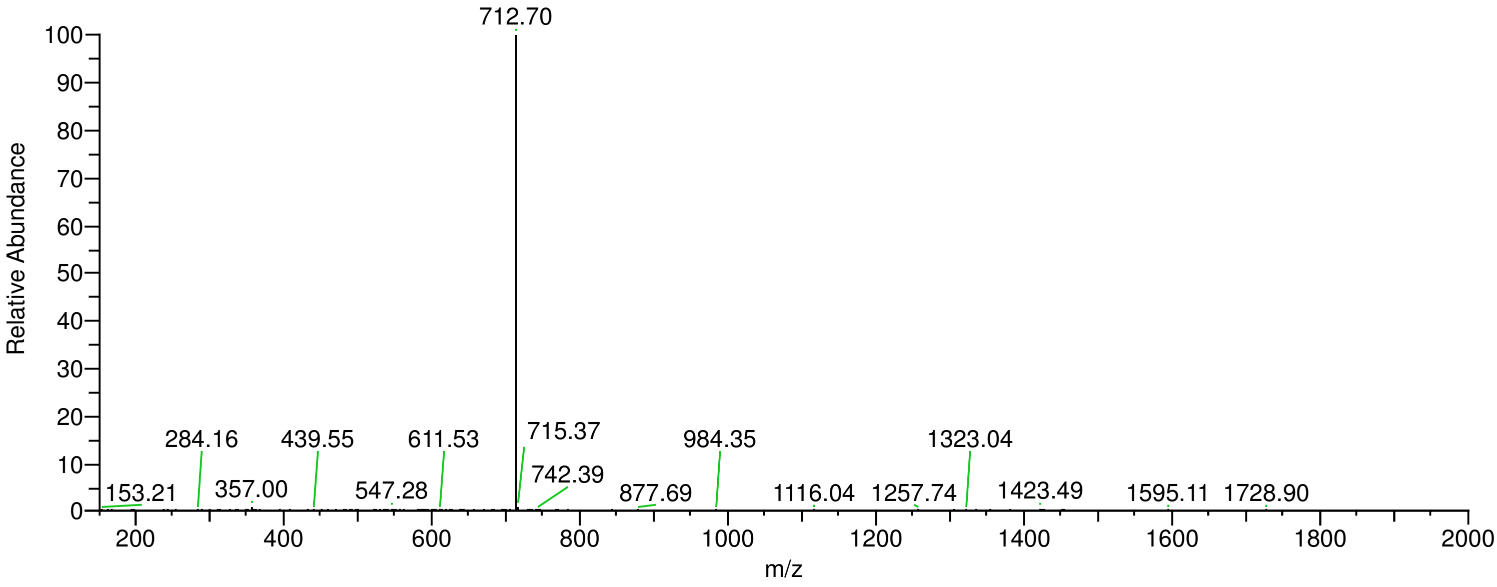

F:\Xcalibur\Data\Zamolo\MATTEO\MP_7aaT-Ir.raw 26.04.2019 17:40:04

RT :0.00-10.00 GNL: 1.70E6 TIC MS MP_7aaT-Ir

LC-MS 27-Ir (ATAḦATA)

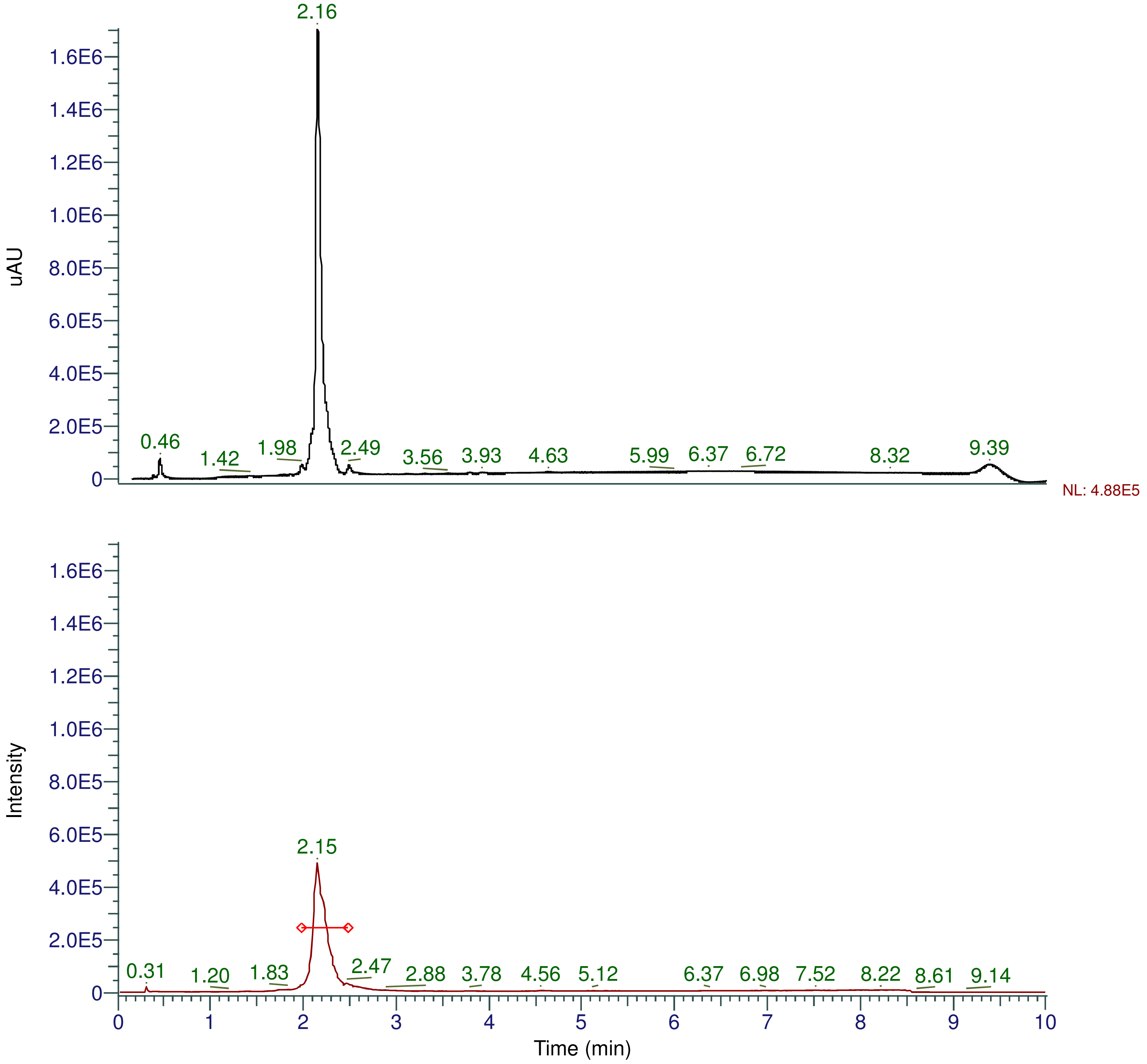

MP_7aaT-Ir #105-146 RT: 1.98-2.49 AV: 42 NL: 4.00E4
T: ITMS + c ESI Full ms [150.00-2000.00]

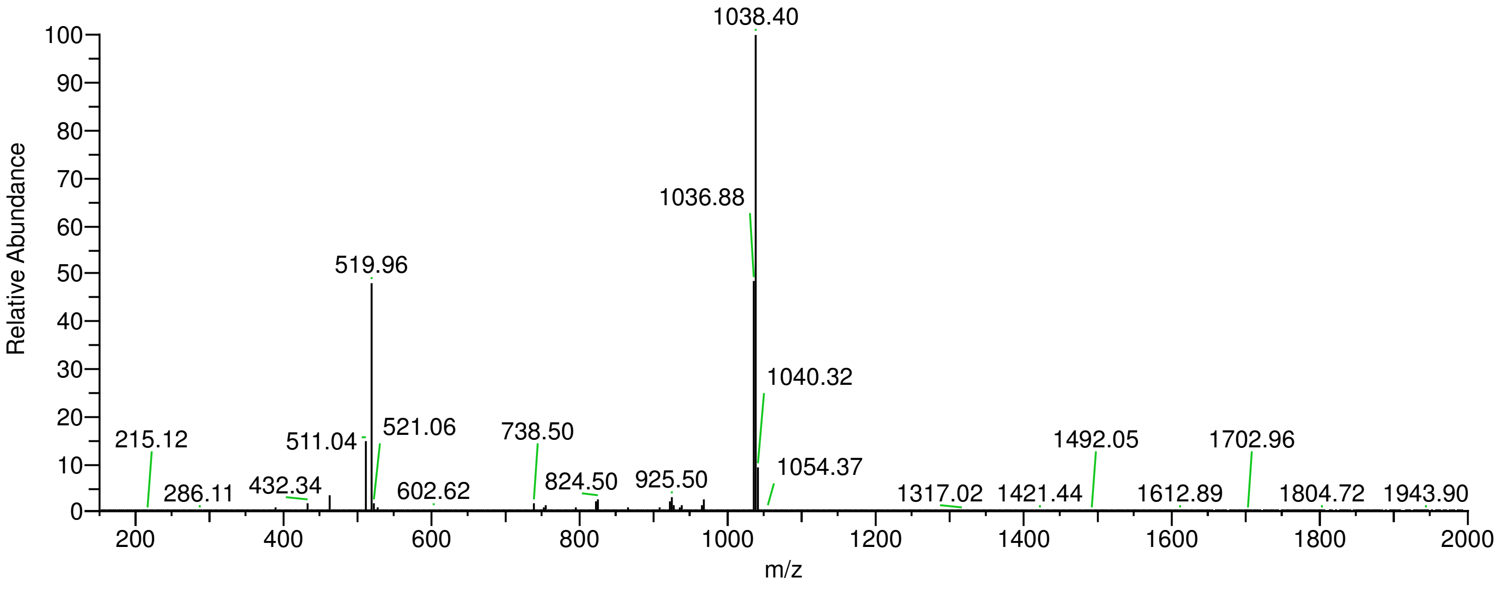

Matteo MP 7aaN_apo_pure_190111124157 1/11/2019 12:51:07 PM Matteo MP 7aaN_apo_pure
NSI pos ACN_H2O

Matteo MP 7aaN_apo_pure_190111124157 #1-6 RT: 0.01-0.14 AV: 6 NL: 2.10E8
T: FTMS + p NSI Full ms [150.00-2000.00]

HR-MS 28-apo (ANAḦANA)

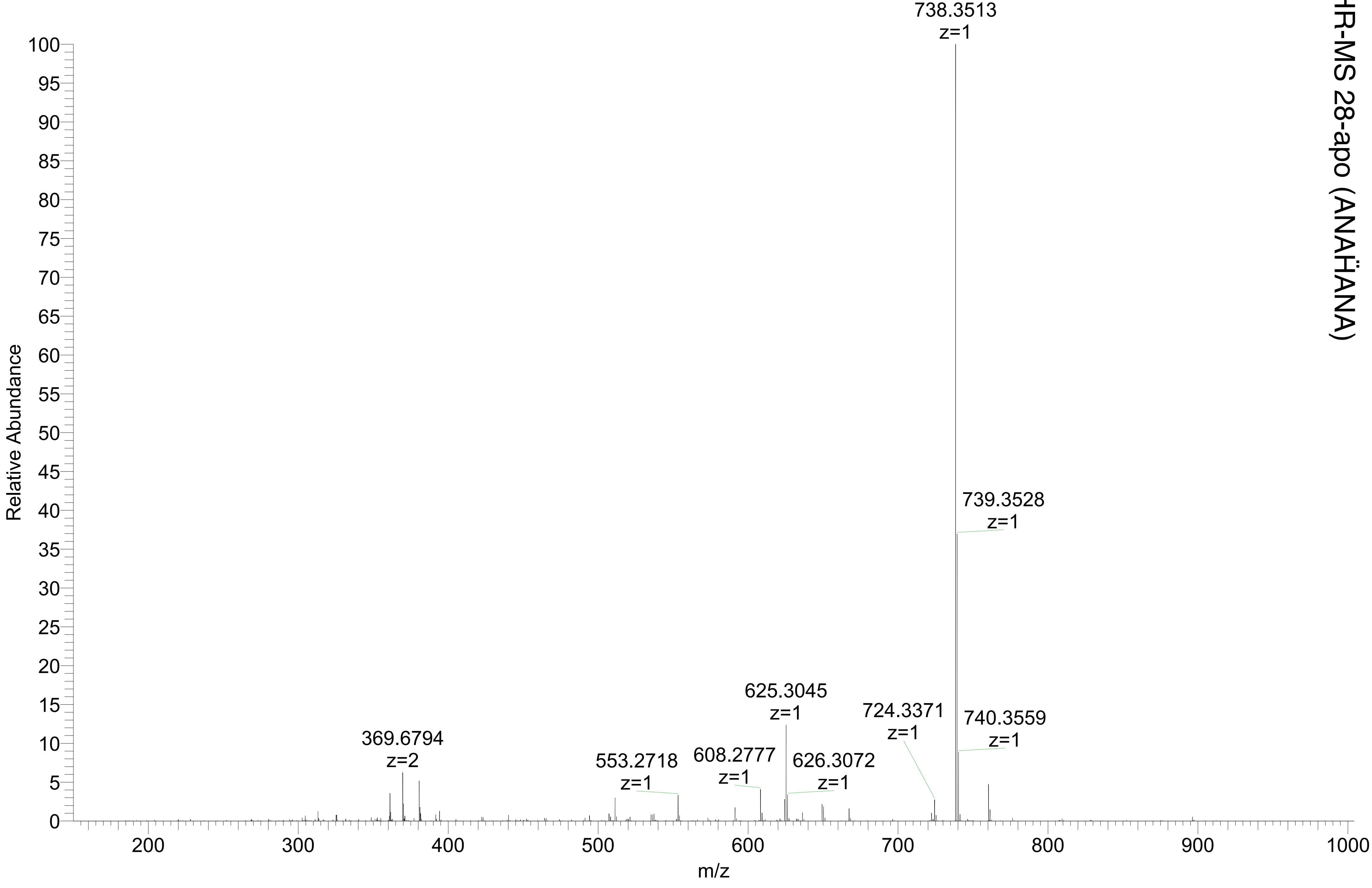

Feller IF 004_7aaN_FIr_190311094147 3/11/2019 10:50:05 AM Feller IF 004_7aaN_FIr
NSI pos MeOH_H2O

Feller IF 004_7aaN_FIr_190311094147 #1-4 RT: 0.02-0.10 AV: 4 NL: 5.21E7
T: FTMS + p NSI Full ms [150.00-2000.00]

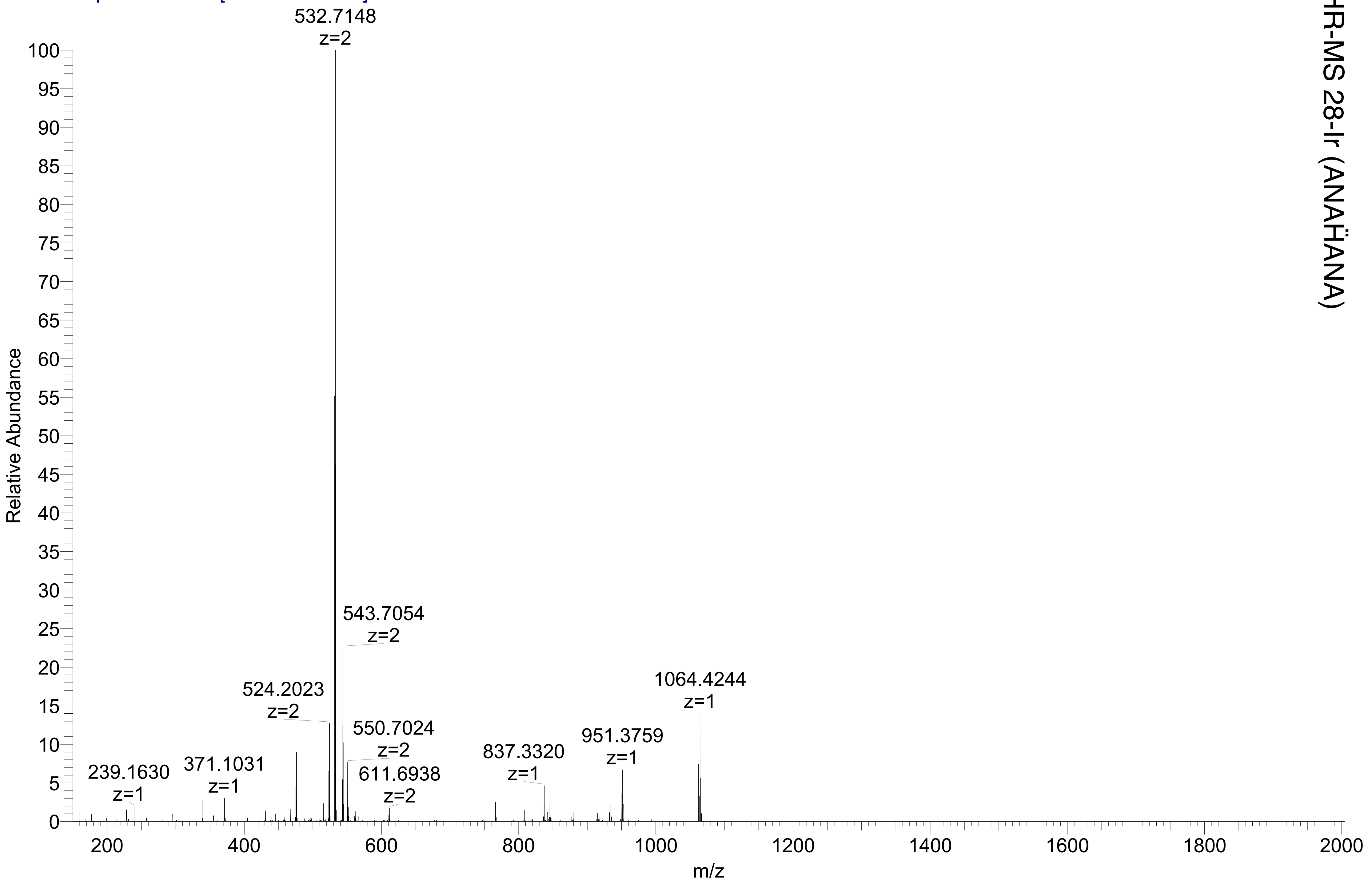

F:\Xcalibur\Data\Zamolo\MATTEO\MP7aaN-apo.raw 21.12.2018 12:19:24

RT :0.00-10.00 TIC MS MP7aaN-apo

LC-MS 28-apo (ANAḦANA)

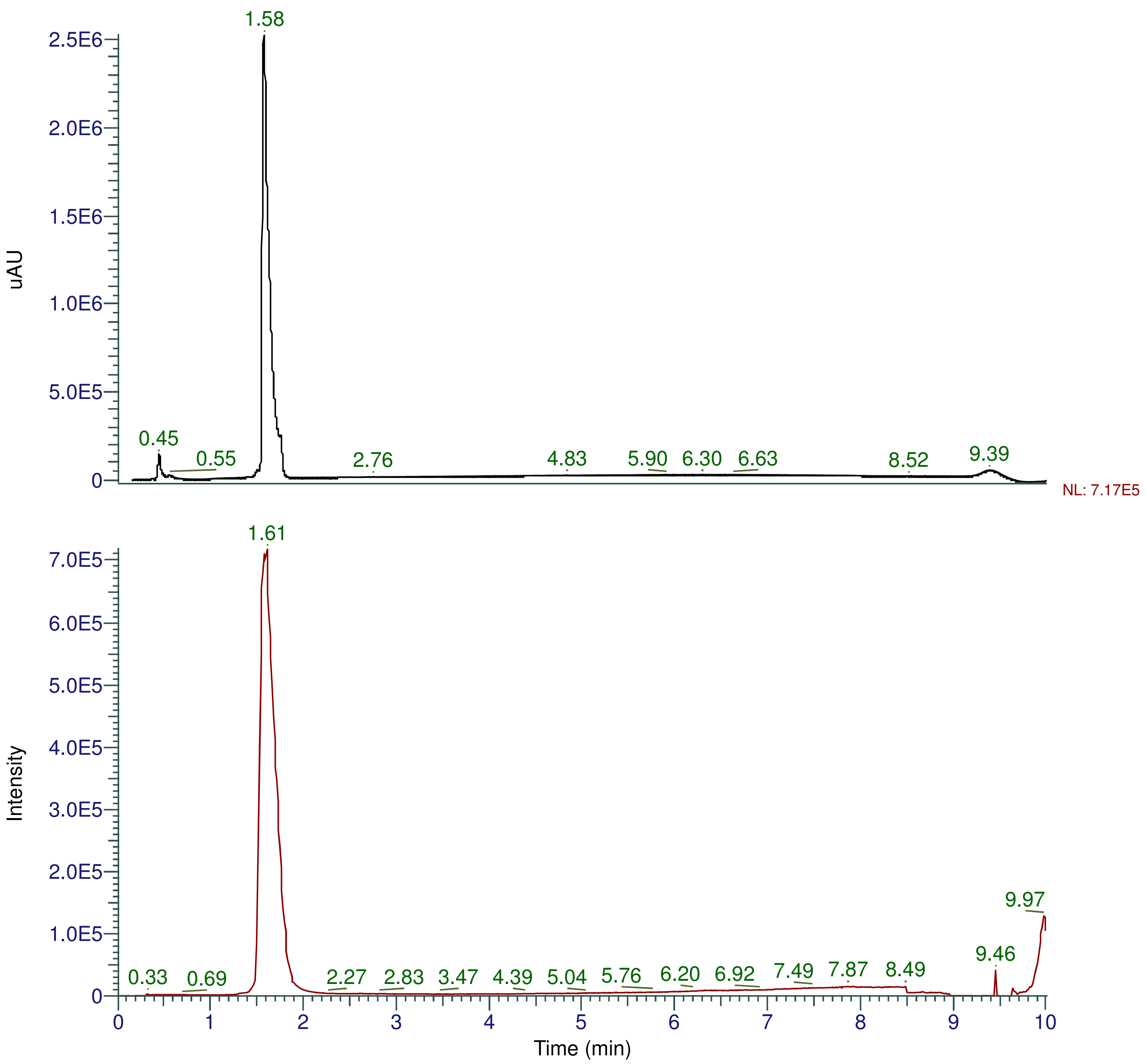

MP7aaN-apo #87 RT: 1.59 AV: 1 NL: 4.46E+005

T: ITMS + c ESI Full ms [150.00-2000.00]

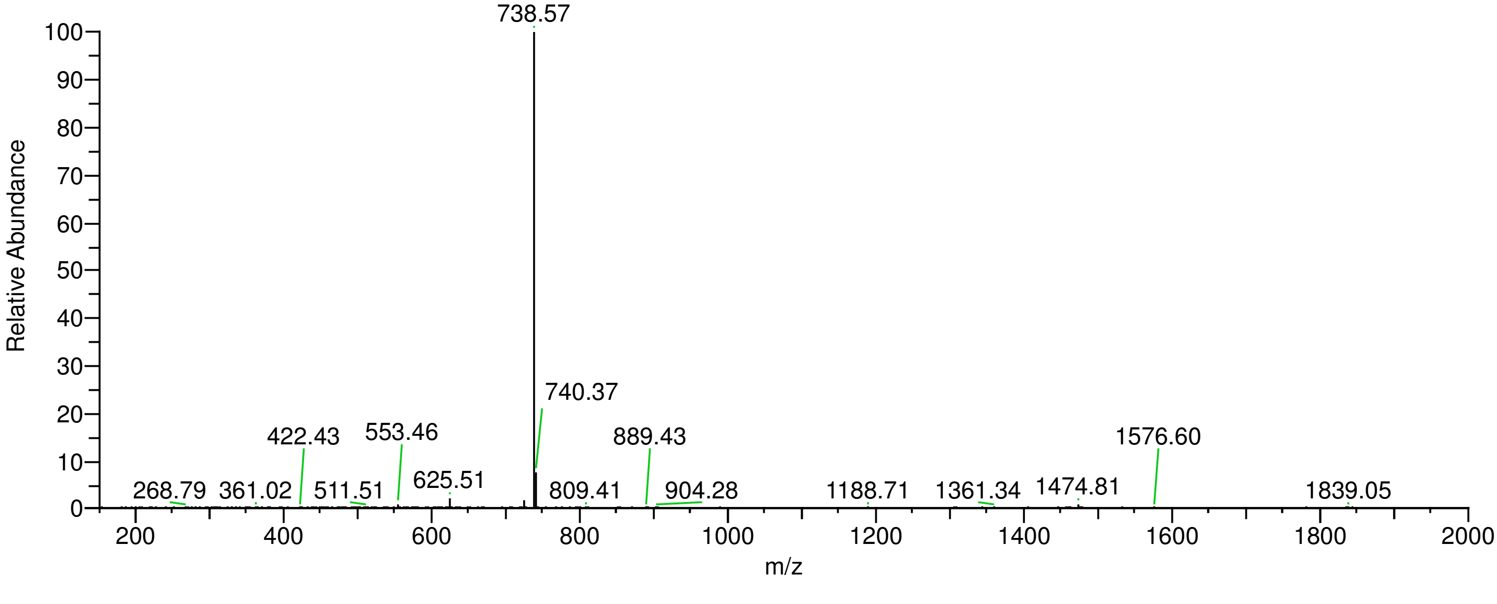

RT :0.00-10.00 GNL: 5.71E5 TIC MS MP_7aaN-Ir

LC-MS 28-Ir (ANAḦANA)

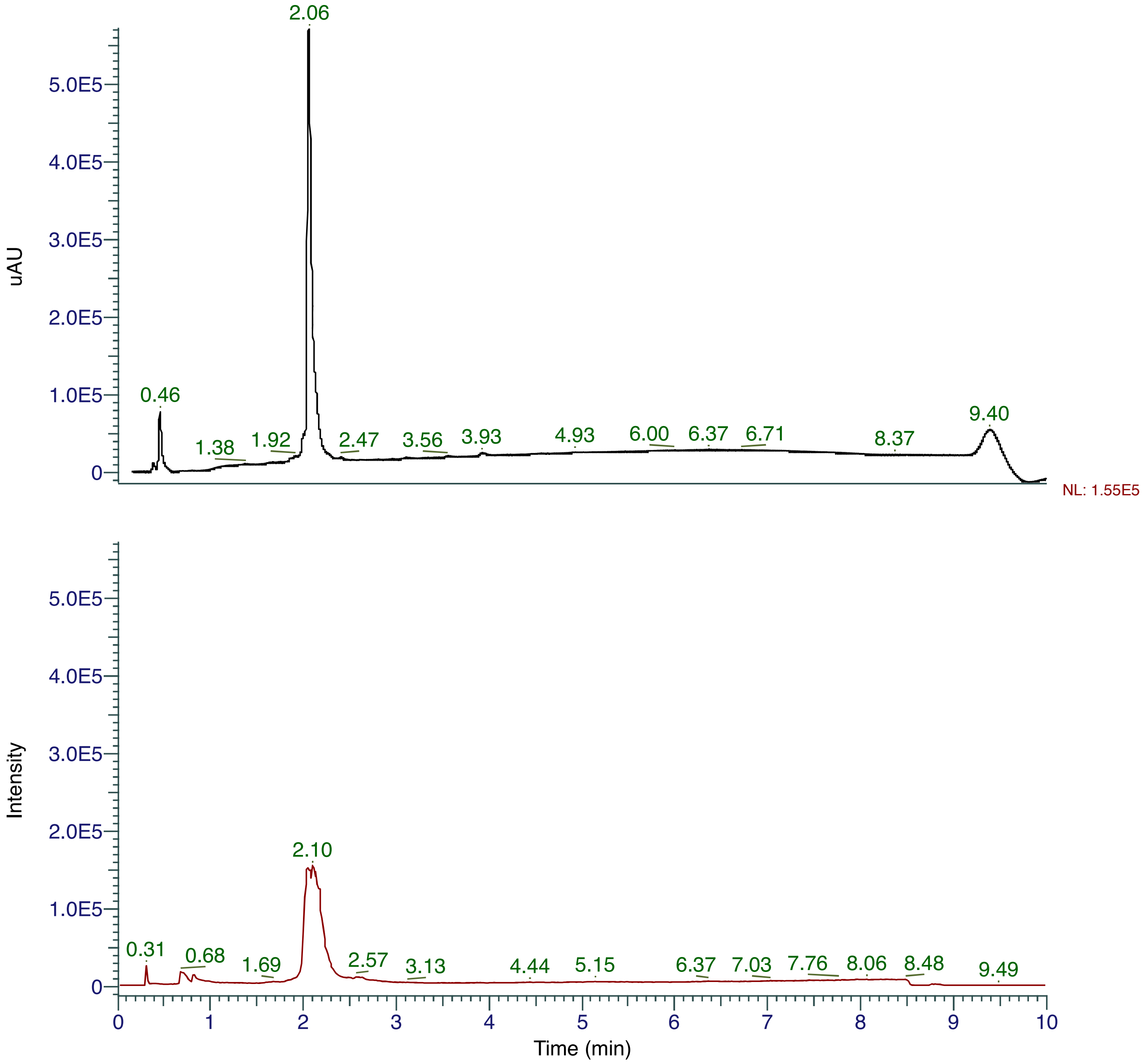

MP_7aaN-Ir #112 RT: 2.06 AV: 1 NL: 2.87E+004
T: ITMS + c ESI Full ms [150.00-2000.00]

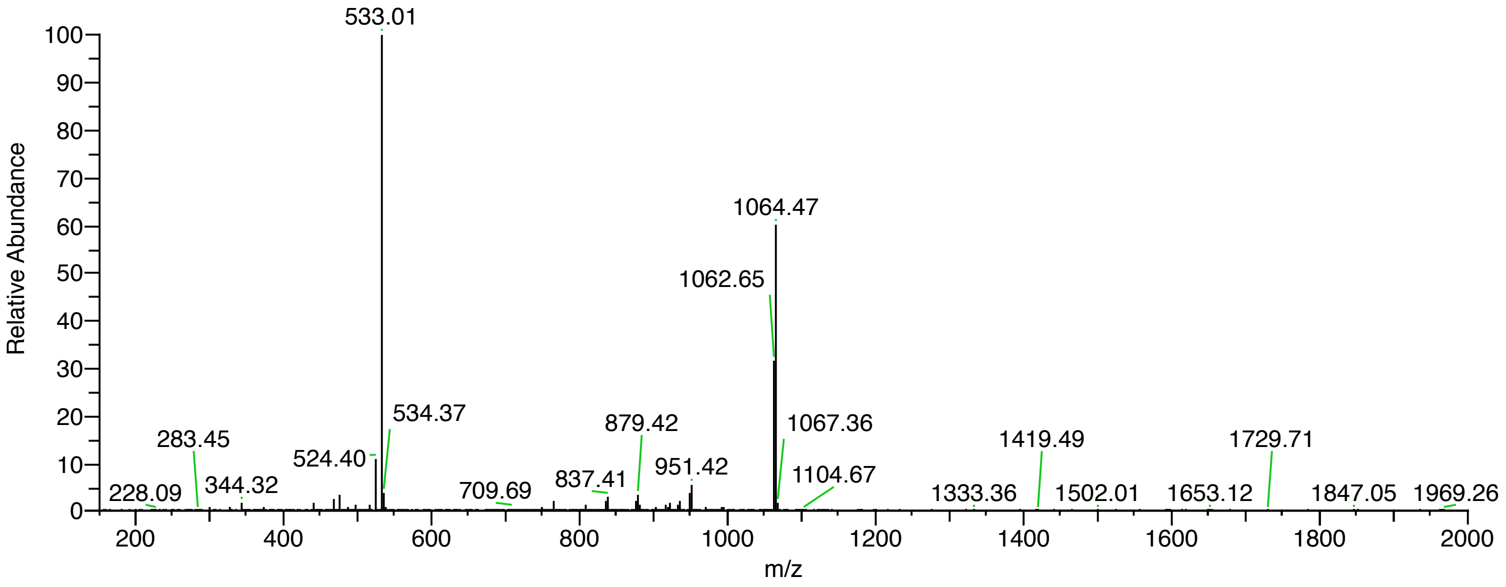

Matteo MP 7aaQ_apo_pure_190111124157 1/11/2019 12:53:28 PM Matteo MP 7aaQ_apo_pure
NSI pos ACN_H2O

Matteo MP 7aaQ_apo_pure_190111124157 #24 RT: 0.78 AV: 1 NL: 1.60E7
T: FTMS + p NSI Full ms [150.00-2000.00]

HR-MS 29-apo (AQAḦAQA)

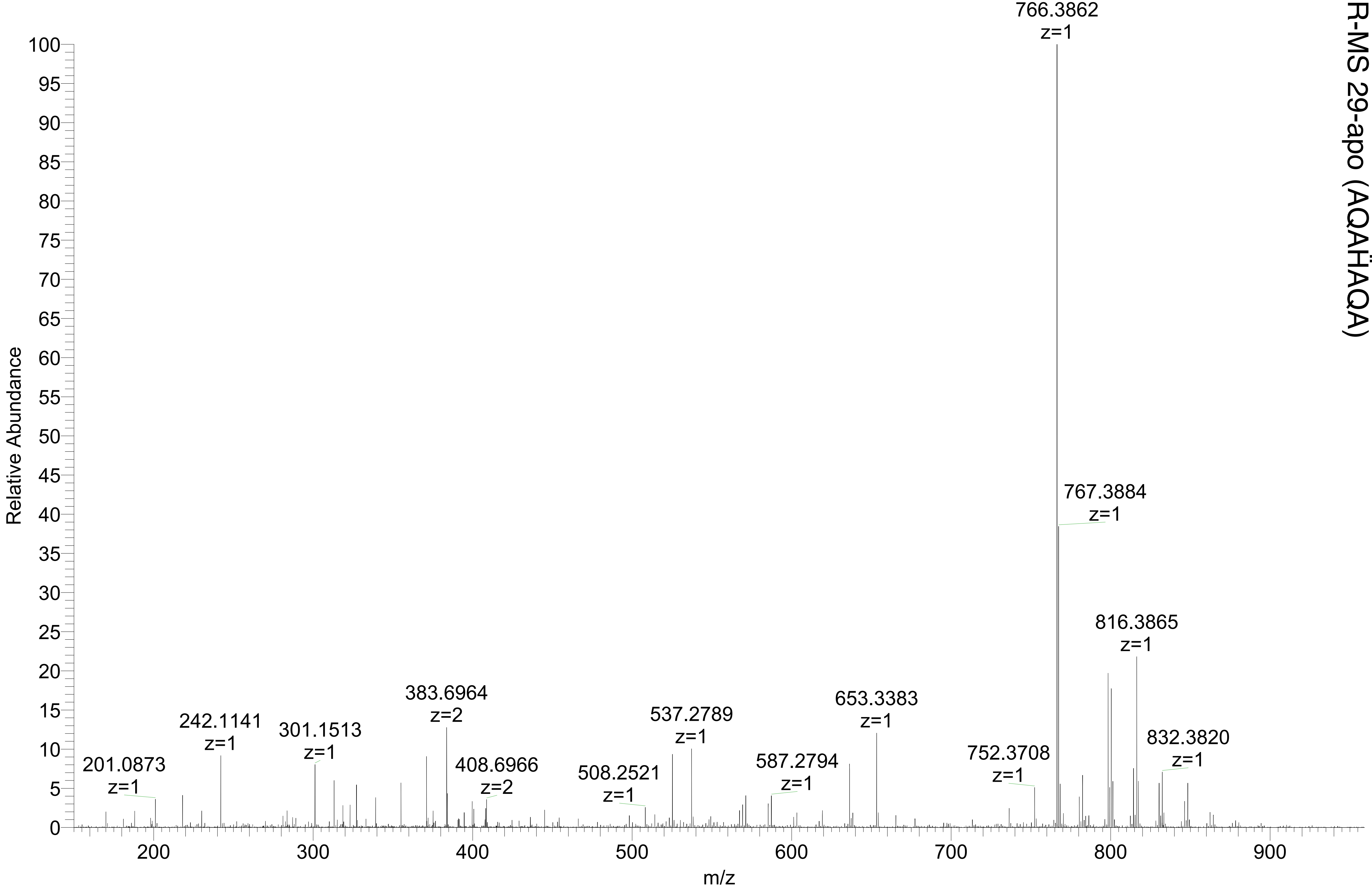

HR-MS 29-Ir (AQAḦAQA)

Feller IF 006_7aaQ_F3_190321141043 3/22/2019 11:20:43 AM Feller IF 006_7aaQ_F3
NSI pos ACN_H2O

Feller IF 006_7aaQ_F3_190321141043 #1-5 RT: 0.01-0.12 AV: 5 NL: 1.01E8
T: FTMS + p NSI Full ms [200.00-2000.00]

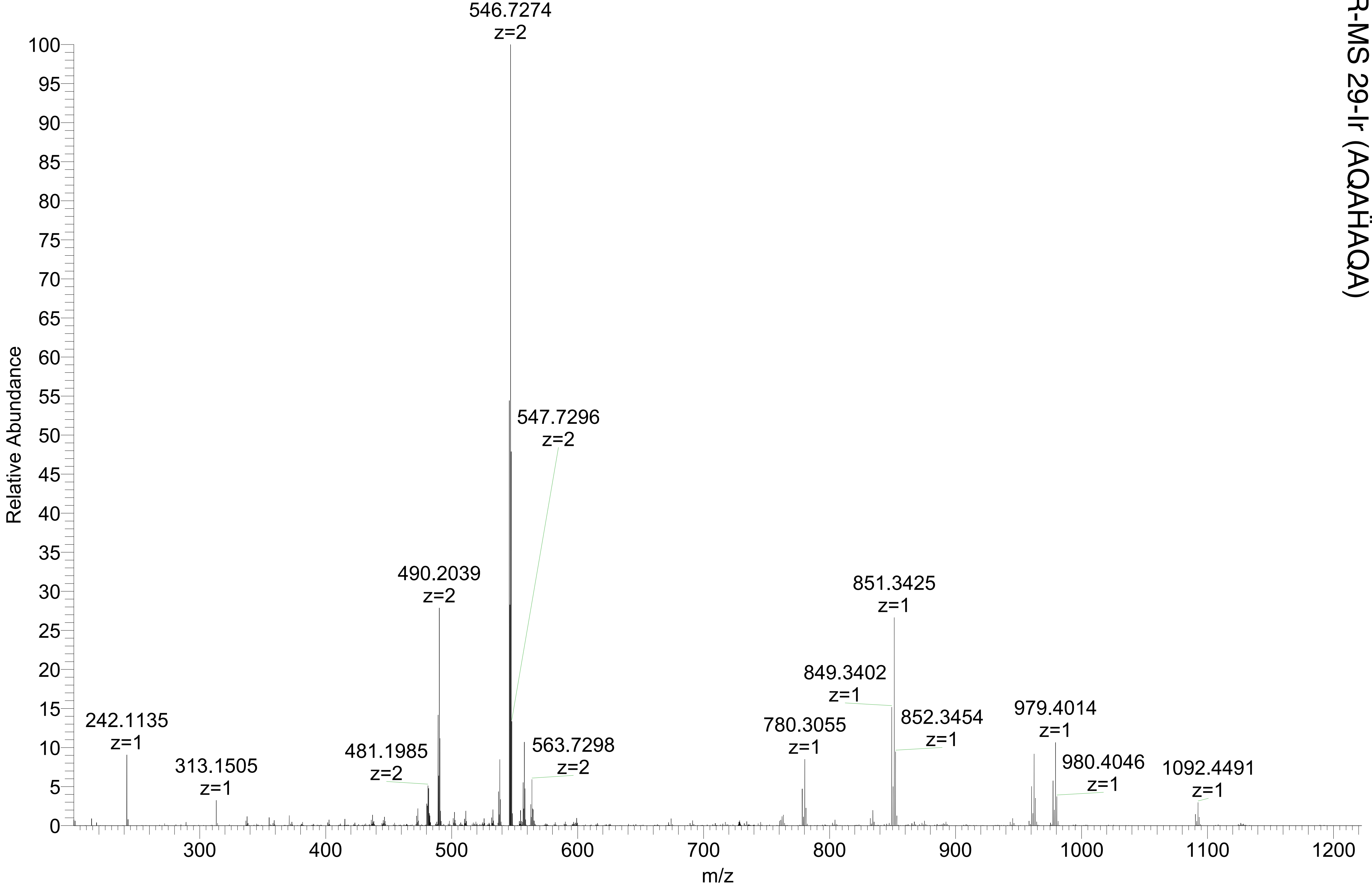

F:\Xcalibur\Data\Zamolo\MATTEO\MP7aaQ-apo.raw 21.12.2018 12:22:43

RT :0.00-10.00 TIC MS MP7aaQ-apo

LC-MS 29-apo (AQAḦAQA)

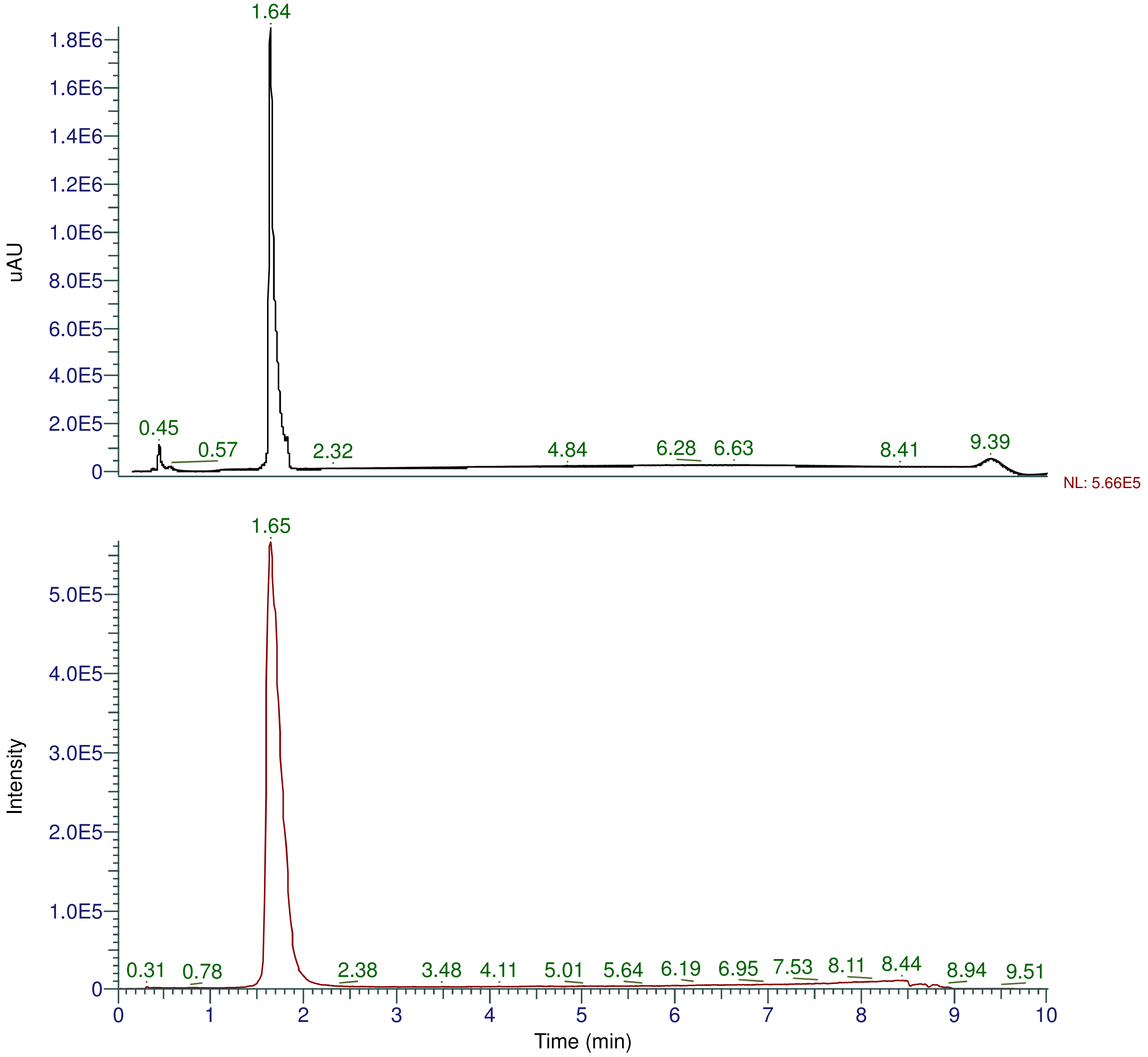

MP7aaQ-apo #89 RT: 1.64 AV: 1 NL: 3.27E+005
T: ITMS + c ESI Full ms [150.00-2000.00]

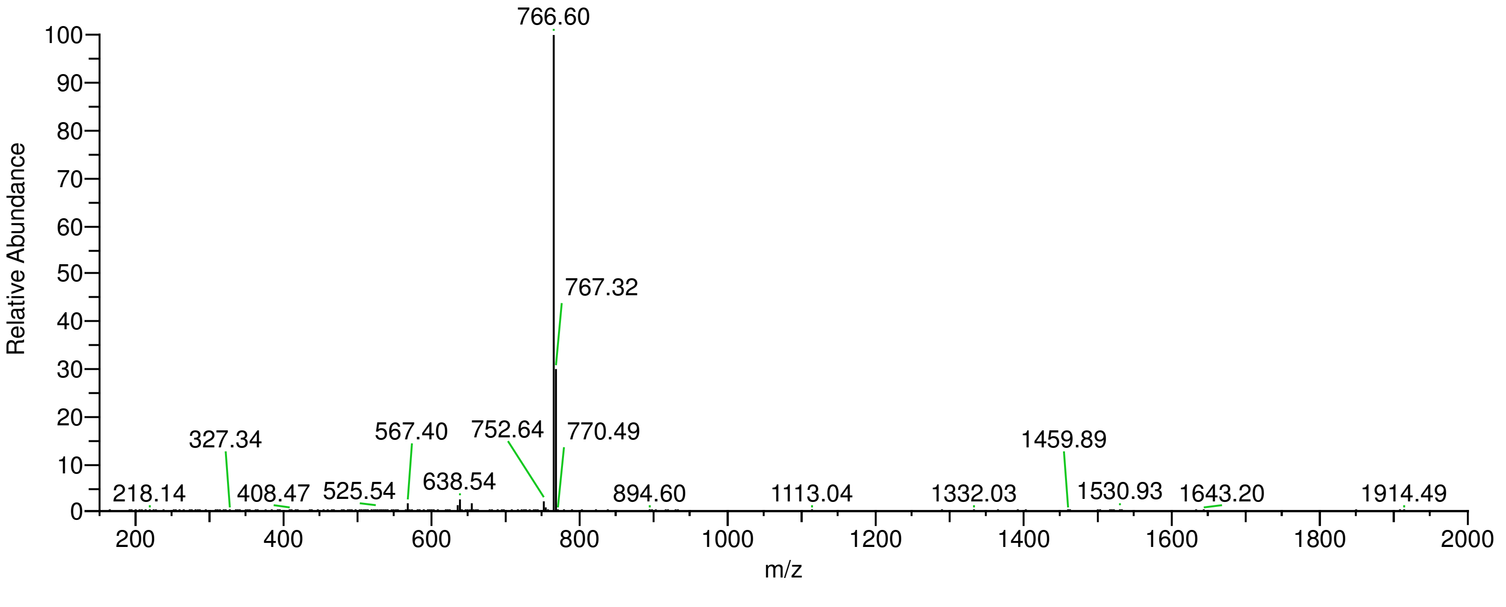

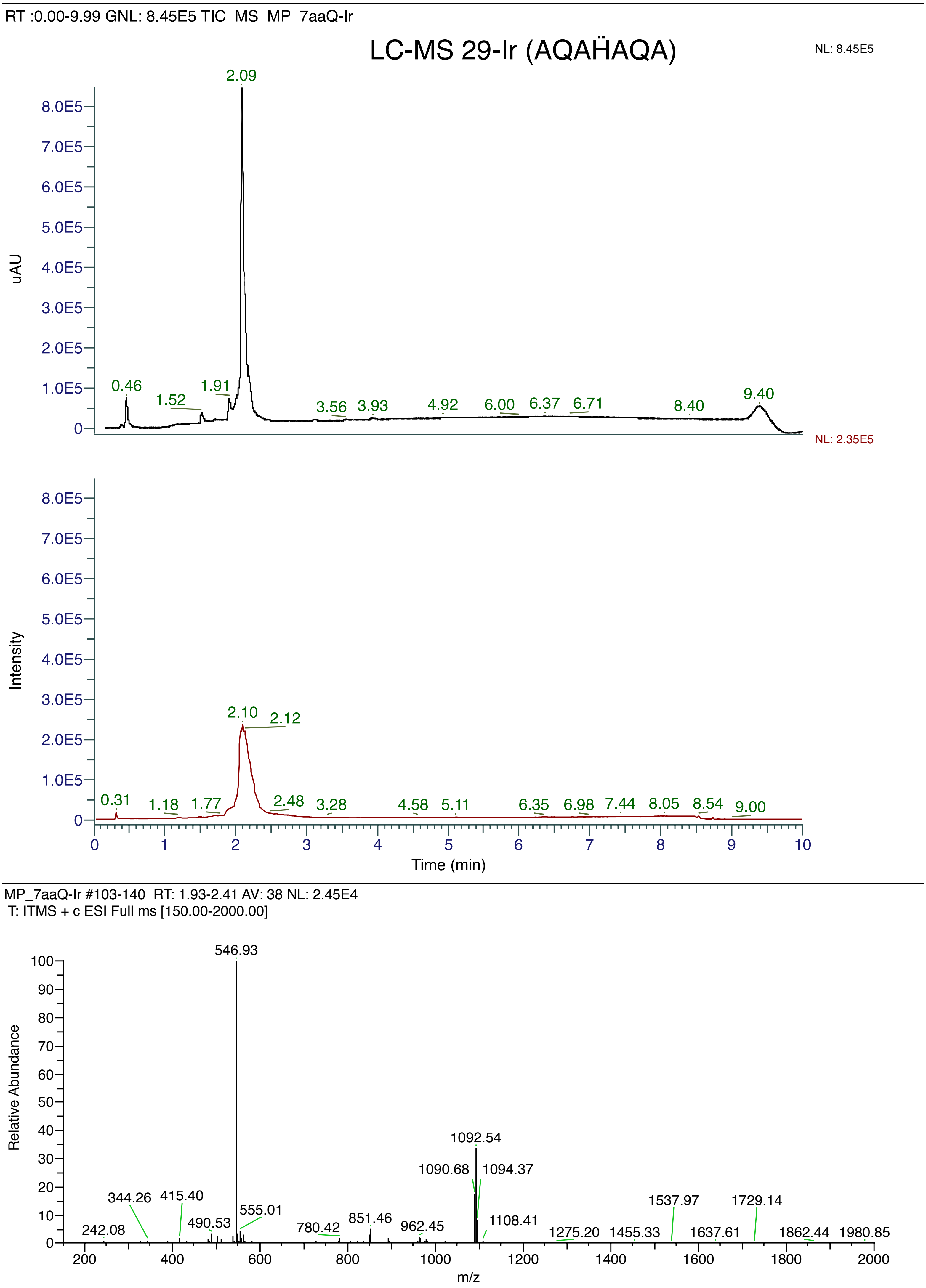
RT :0.00-9.99 GNL: 8.45E5 TIC MS MP_7aaQ-Ir
LC-MS 29-Ir (AQAḦAQA)
NL: 8.45E5
uAU
2.09
0.46
1.52
1.91
3.56
3.93
4.92
6.00
6.37
6.71
8.40
9.40
NL: 2.35E5
Intensity
2.10
2.12
0.31
1.18
1.77
2.48
3.28
4.58
5.11
6.35
6.98
7.44
8.05
8.54
9.00
Time (min)
MP_7aaQ-Ir #103-140 RT: 1.93-2.41 AV: 38 NL: 2.45E4
T: ITMS + c ESI Full ms [150.00-2000.00]
Relative Abundance
546.93
1092.54
1090.68
1094.37
344.26
415.40
555.01
490.53
242.08
780.42
851.46
962.45
1108.41
1537.97
1729.14
1275.20
1455.33
1637.61
1862.44
1980.85
m/z

HR-MS 30-apo (ARAḦARA)

Matteo MP 7aaR_apo_pure_190111124157 1/11/2019 12:59:47 PM Matteo MP 7aaR_apo_pure
NSI pos ACN_H2O

Matteo MP 7aaR_apo_pure_190111124157 #1-5 RT: 0.02-0.14 AV: 5 NL: 8.00E7
T: FTMS + p NSI Full ms [150.00-2000.00]

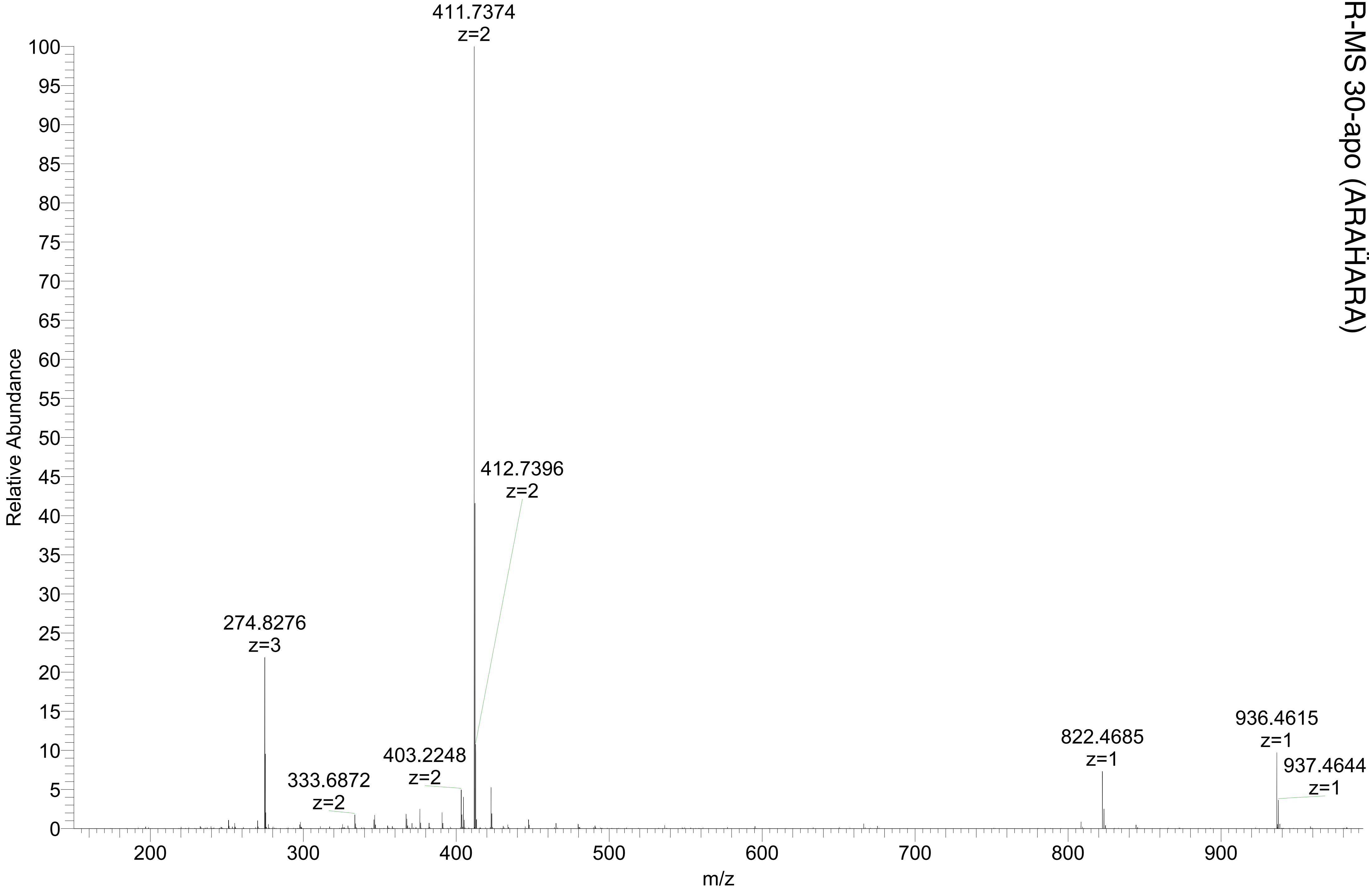

Matteo MP 7aaaR_Ir 1pure_190108112013 1/9/2019 9:04:44 AM Matteo MP 7aaaR_Ir 1pure
NSI pos ACN_H2O

Matteo MP 7aaaR_Ir 1pure_190108112013 #1-4 RT: 0.02-0.12 AV: 4 NL: 1.35E6
T: FTMS + p NSI Full ms [150.00-2000.00]

HR-MS 30-Ir (ARAḦARA)

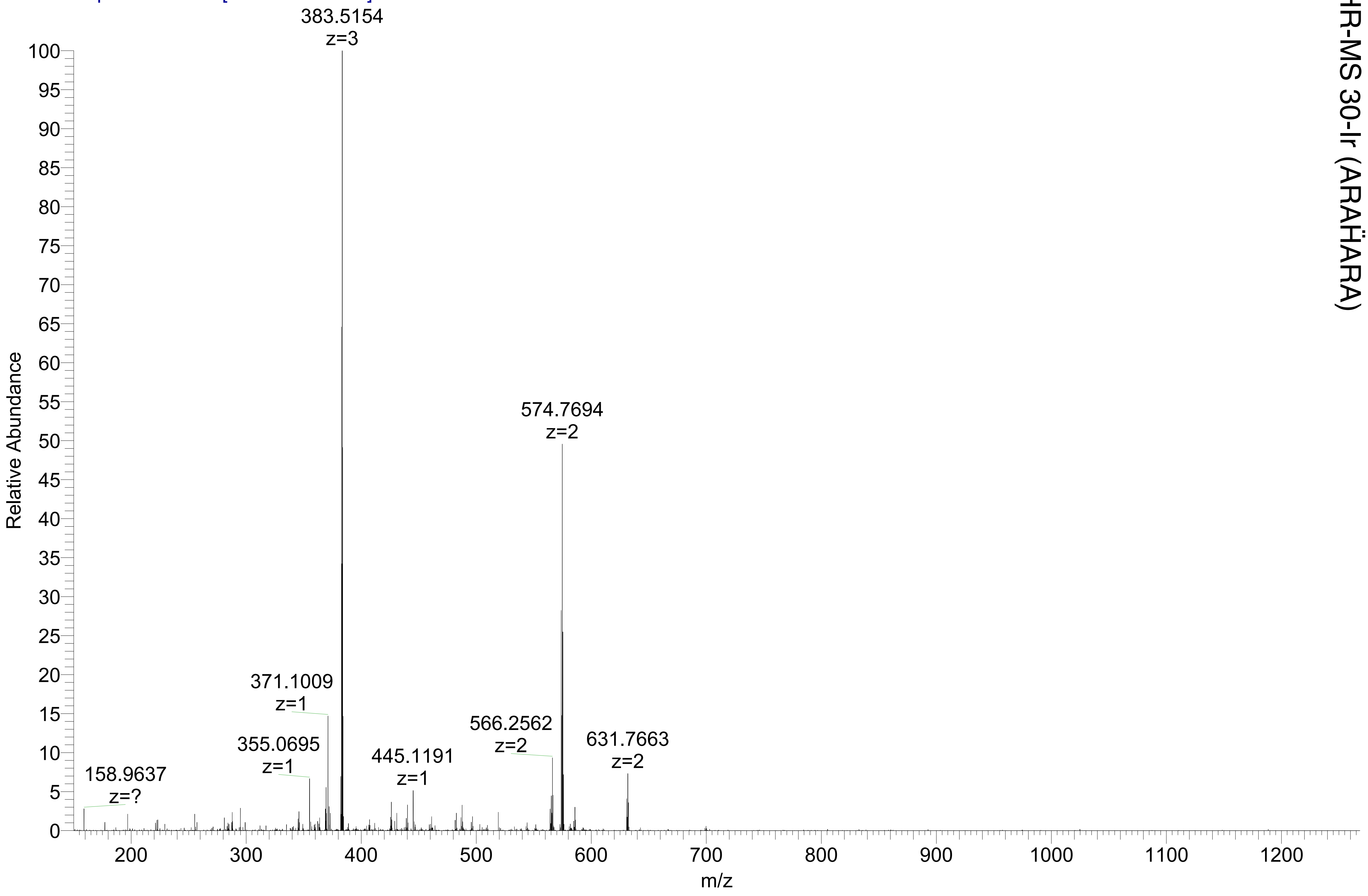

RT :0.00-9.99 GNL: 9.01E5 TIC MS MP_7aaR-apo

LC-MS 30-apo (ARAḦARA)

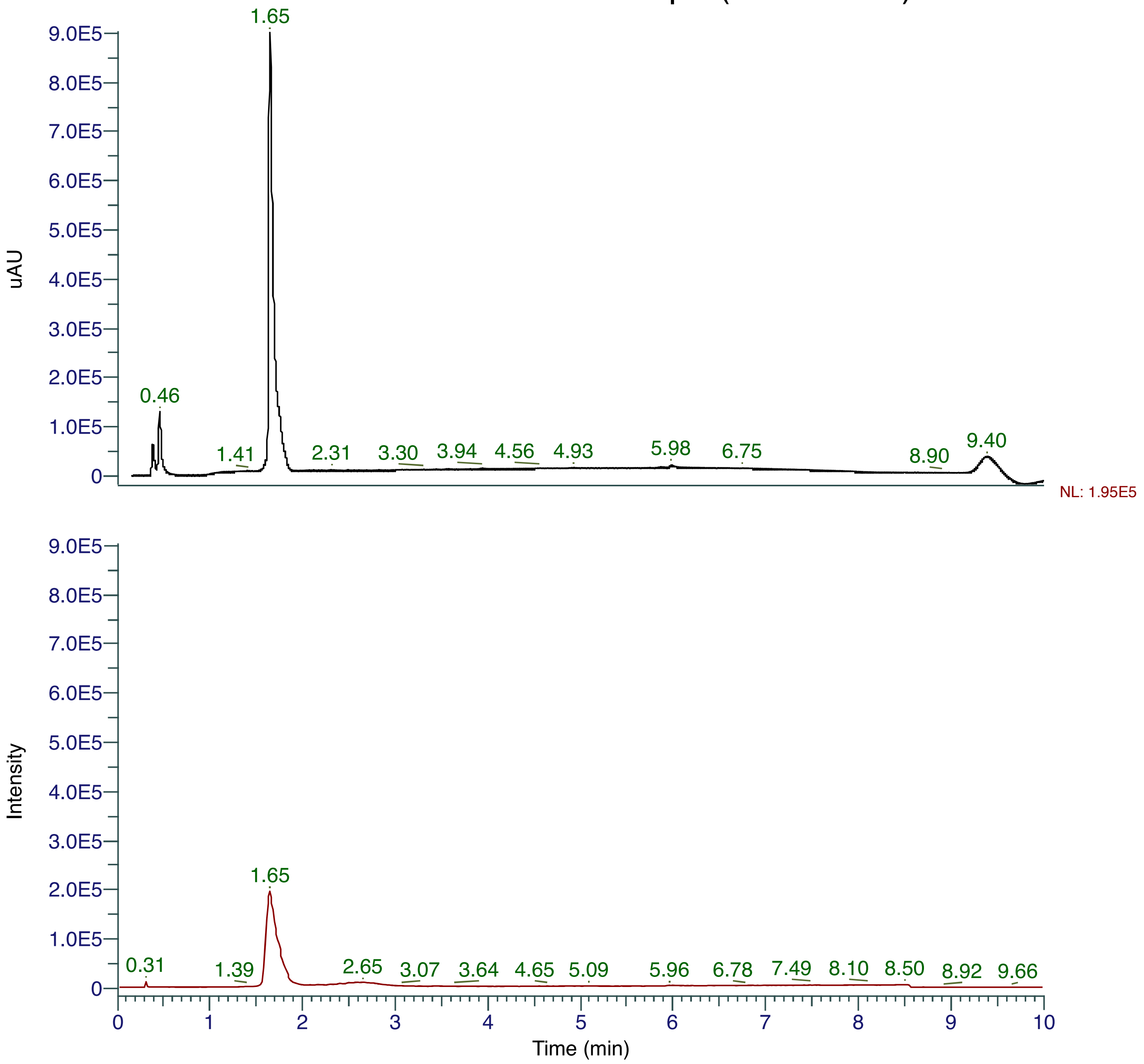

MP_7aaR-apo #83-112 RT: 1.57-1.99 AV: 30 NL: 3.08E4
T: ITMS + c ESI Full ms [150.00-2000.00]

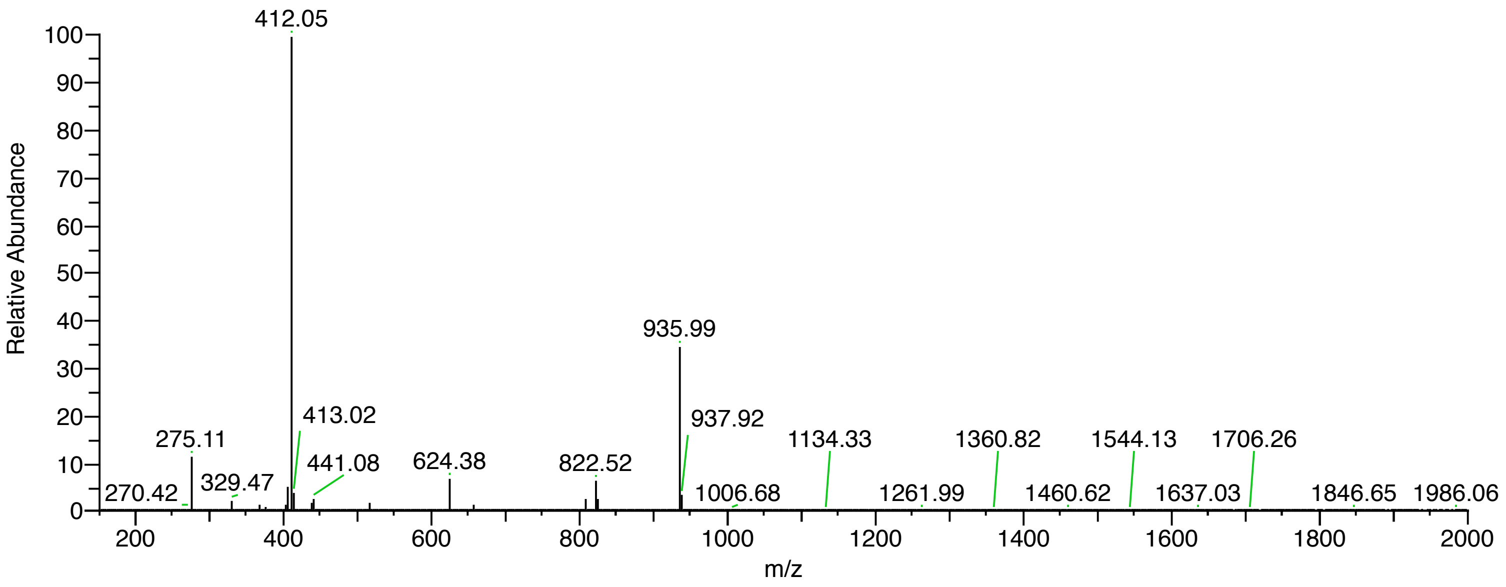

RT :0.00-9.99 GNL: 7.16E5 TIC MS MP_7aaR-Ir

LC-MS 30-Ir (ARAḦARA)

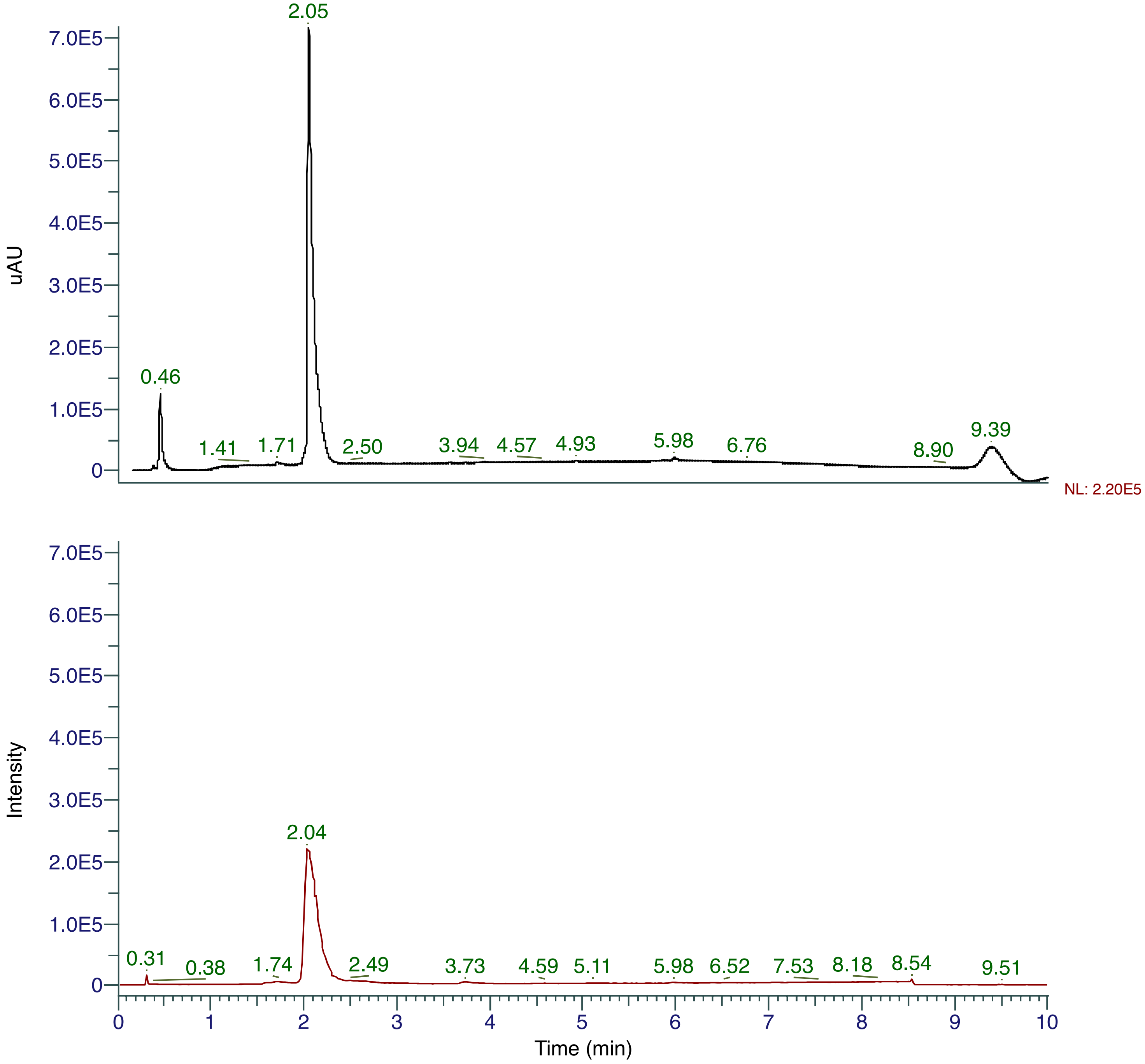

MP_7aaR-Ir #103-123 RT: 1.95-2.21 AV: 21 NL: 4.40E4
T: ITMS + c ESI Full ms [150.00-2000.00]

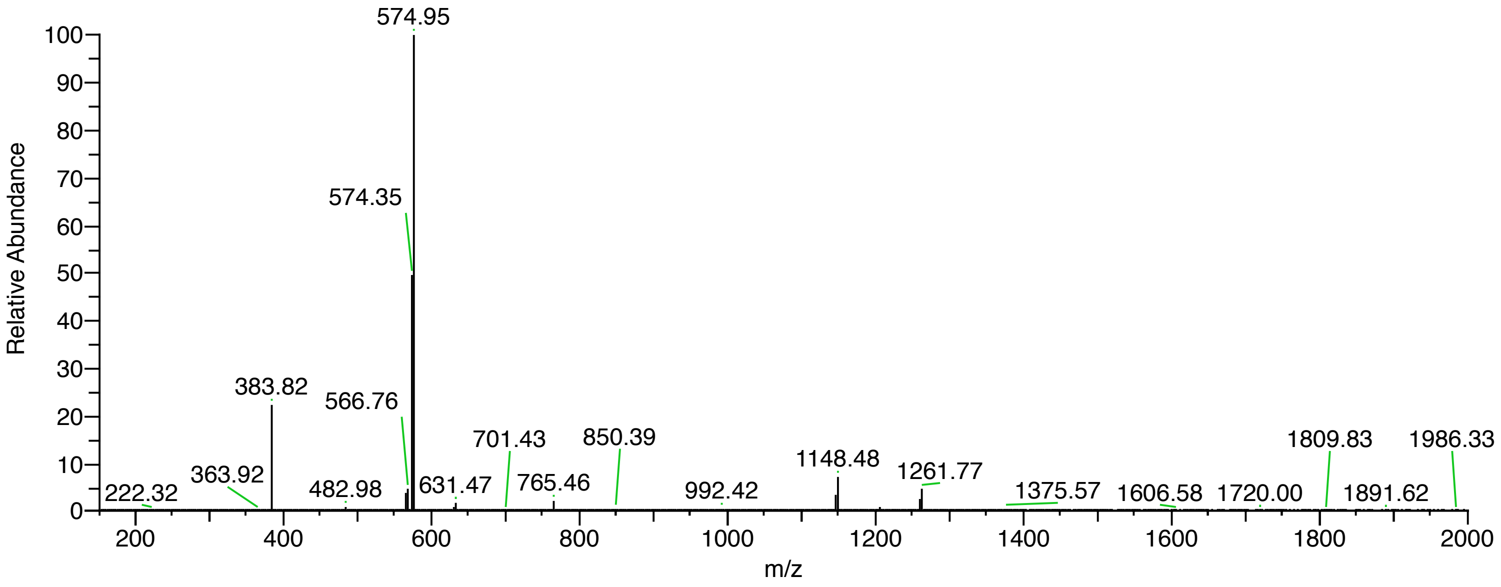

Supplement: CC-057-D1CC03158A-s001 [file CC-057-D1CC03158A-s001.pdf]
